# Photoredox‐Catalyzed Decarboxylative Bromination, Chlorination and Thiocyanation Using Inorganic Salts

**DOI:** 10.1002/anie.202309684

**Published:** 2023-08-10

**Authors:** Jingjing Wu, Chao Shu, Zhihang Li, Adam Noble, Varinder K. Aggarwal

**Affiliations:** ^1^ School of Chemistry University of Bristol Cantock's Close BS8 1TS Bristol UK; ^2^ Current address: Frontiers Science Center for Transformative Molecules School of Chemistry and Chemical Engineering Zhangjiang Institute for Advanced Study Shanghai Jiao Tong University No. 429, Zhangheng Road 200213 Shanghai China; ^3^ Current address: National Key Laboratory of Green Pesticide College of Chemistry Central China Normal University (CCNU) 152 Luoyu Road 430079 Wuhan Hubei China

**Keywords:** Copper Catalysis, Decarboxylation, Halogenation, Photoredox Catalysis, Thiocyanation

## Abstract

Decarboxylative halogenation reactions of alkyl carboxylic acids are highly valuable reactions for the synthesis of structurally diverse alkyl halides. However, many reported protocols rely on stoichiometric strong oxidants or highly electrophilic halogenating agents. Herein, we describe visible‐light photoredox‐catalyzed decarboxylative halogenation reactions of *N*‐hydroxyphthalimide‐activated carboxylic acids that avoid stoichiometric oxidants and use inexpensive inorganic halide salts as the halogenating agents. Bromination with lithium bromide proceeds under simple, transition‐metal‐free conditions using an organic photoredox catalyst and no other additives, whereas dual photoredox‐copper catalysis is required for chlorination with lithium chloride. The mild conditions display excellent functional‐group tolerance, which is demonstrated through the transformation of a diverse range of structurally complex carboxylic acid containing natural products into the corresponding alkyl bromides and chlorides. In addition, we show the generality of the dual photoredox‐copper‐catalyzed decarboxylative functionalization with inorganic salts by extension to thiocyanation with potassium thiocyanide, which was applied to the synthesis of complex alkyl thiocyanates.

## Graphical abstract

Decarboxylative halogenation typically requires stoichiometric strong oxidants or electrophilic halogenating agents. It is now shown that simple inorganic halide salts can be used for halogenation of *N*‐hydroxyphthalimide‐activated carboxylic acids under visible‐light photoredox catalysis. Halodecarboxylation proceeded with LiBr and LiCl, and the generality of the conditions was demonstrated by extension to decarboxylative thiocyanation with KSCN.

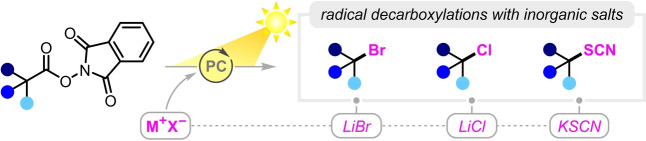

## Introduction

The carboxylic acid functional group is commonly found in feedstock chemicals and is ubiquitous in natural products and drug molecules. As a result, synthetic methods for the derivatization of carboxylic acids are highly useful for transforming these readily available substrates into valuable products. Radical‐mediated decarboxylative reactions of alkyl carboxylic acids have proved particularly attractive since they provide the opportunity to access structurally diverse and synthetically versatile building blocks through late‐stage functionalization of complex materials and medicines.^[1]^ Research in this area has led to the development of numerous decarboxylative processes for the installation of C(sp^3^)−C,^[2]^ C(sp^3^)−B,^[3]^ C(sp^3^)‐pnictogen,[^4^, ^5^] C(sp^3^)‐chalcogen,^[4]^ and C(sp^3^)‐halogen bonds.^[6]^

**Scheme 1:**
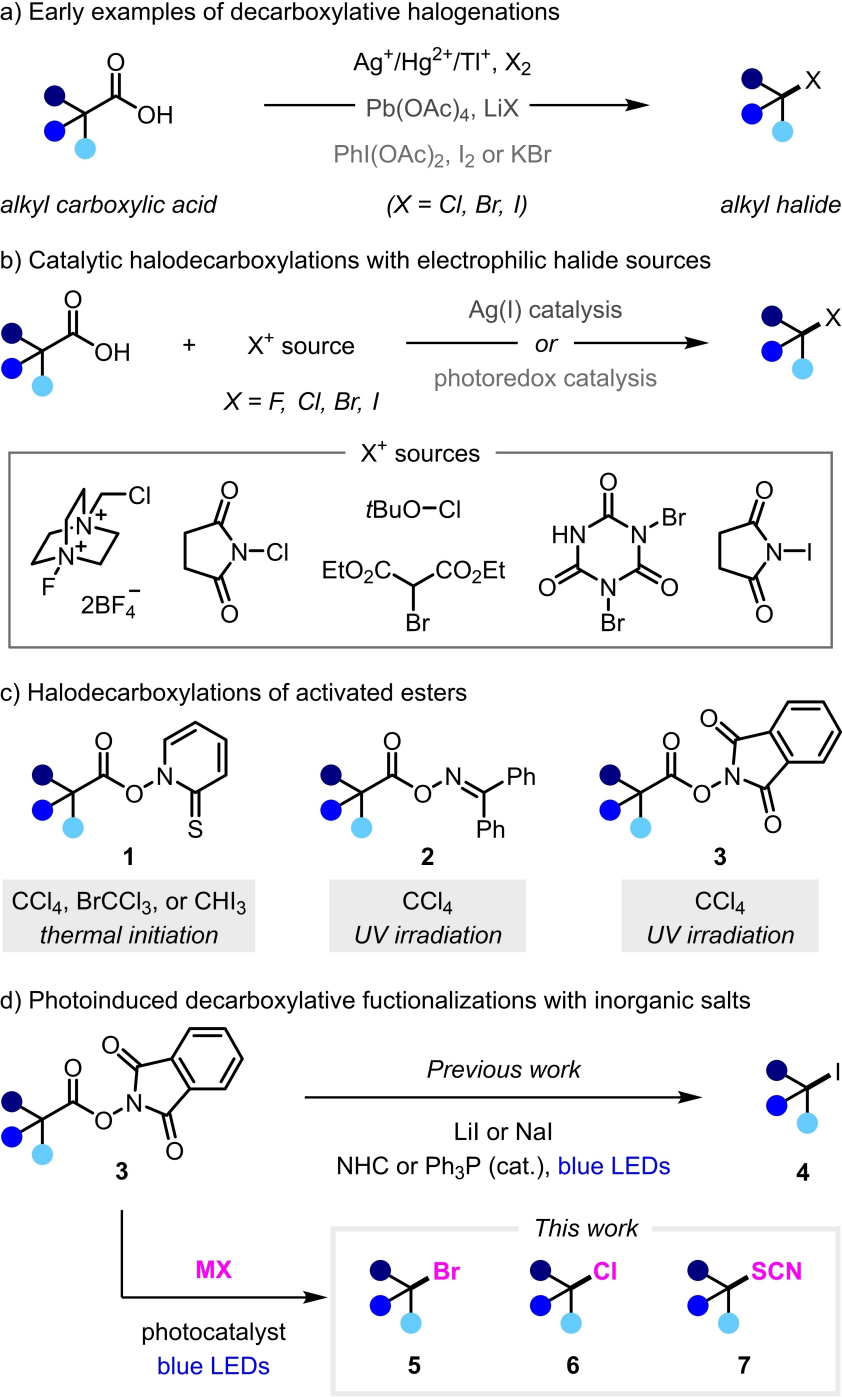
Decarboxylative halogenation reactions.

Decarboxylative halogenation, commonly referred to as the Hunsdiecker reaction, is one of the most well‐known decarboxylative transformations and an attractive method for synthesizing alkyl halides, which are highly versatile building blocks in synthetic chemistry (Scheme 1a).^[6]^ The generality of this reaction was first reported in 1942, where strictly anhydrous silver carboxylate salts were shown to undergo bromodecarboxylation in the presence of bromine.^[7]^ Due to the difficult preparation of dry Ag^I^ carboxylates, modified procedures that utilized Hg^II^,^[8]^ Pb^IV^,^[9]^ or Tl^I^ salts were later developed.^[10]^ In 1986, Suárez and co‐workers reported that these toxic heavy metals could be replaced by stoichiometric PhI(OAc)_2_ in iododecarboxylations with I_2_ under photo‐irradiation,^[11]^ and this method has since been extended to bromo‐ and chlorodecarboxylation.[^12^, ^13^, ^14^] More recent developments have focused on catalytic variations (Scheme 1b), including those that employ silver and photoredox catalysis.[^15^, ^16^] However, most of these methods rely on the use of a highly oxidizing electrophilic halogenating agents, which are incompatible with certain functional groups (e.g., electron‐rich aromatic rings and olefins). Whilst methods that use simple, inexpensive metal halides have been reported, these still require stoichiometric strong oxidants, such as Pb(OAc)_4_ ^[9a]^ or PhI(OAc)_2_,[^11^, ^12^] which also limit functional‐group tolerance. Therefore, there is a need for alternative halodecarboxylation methods that proceed under mild conditions and in the absence of strong oxidants.

In 1983, Barton reported a modified halodecarboxylation wherein carboxylic acids were first converted to thiohydroxamate esters **1**, which enabled halogenations with CCl_4_, CBrCl_3_ and CHI_3_ via radical chain mechanisms (Scheme 1c).^[17]^ Similar approaches were later reported by the groups Hasebe and Okada, who replaced the unstable thiohydroxamate esters **1** with benzophenone oxime esters **2** and *N*‐hydroxyphthalimide (NHP) esters **3**, respectively, both of which undergo chlorodecarboxylation with CCl_4_ upon UV irradiation.[^18^, ^19^] These methods successfully allow halodecarboxylation while avoiding strongly oxidizing or electrophilic reagents, but they still require the use of solvent quantities of toxic halogenating agents. This limitation was overcome in 2020, when the Chen and Shang groups independently reported visible‐light‐mediated iododecarboxylation reactions of NHP esters **3** with simple iodide salts in the presence of catalytic amounts of an *N*‐heterocyclic carbene (NHC) or PPh_3_ (Scheme 1d).^[20]^ These mild conditions proceed under transition‐metal‐ and photocatalyst‐free conditions due to the formation of photoactive electron donor‐acceptor complexes between the Lewis base catalyst, iodide salt, and ester **3**. Excellent functional‐group tolerance was demonstrated, but the reactions were limited to iodinations, since the addition of other halide salts (e.g., LiBr or LiCl) failed to provide the corresponding bromides and chlorides.^[20b]^

We questioned whether the halodecarboxylation of NHP esters **3** could be extended beyond iodination to enable a broad range of decarboxylative functionalization reactions with simple inorganic salts (Scheme 1d). Herein, we report the development of a transition‐metal‐free, visible‐light photoredox‐catalyzed decarboxylative bromination of NHP esters with LiBr. Furthermore, we demonstrate that extension to chlorinations with LiCl is possible through the use of dual photoredox‐copper catalysis. Finally, this dual catalytic process could also be used for other decarboxylative functionalization reactions with nucleophilic inorganic salts, as demonstrated by the development of a decarboxylative thiocyanation reaction.

## Results and Discussion

We began our studies by investigating the decarboxylative bromination of NHP ester **3 a** with LiBr in MeCN under blue‐light irradiation (Table 1). Without any photocatalyst or additive, alkyl bromide **5 a** was formed in only 5 % yield, with 95 % **3 a** remaining (entry 1). However, in the presence of the photoredox catalysts Ir(ppy)_3_, Ir[dF(CF_3_)ppy]_2_(dtbbpy)PF_6_ (**[Ir‐1]**), or 4CzIPN, full conversion of **3 a** was observed and **5 a** was generated in high yields (entries 2–4). Pleasingly, the inexpensive organic photocatalyst, 4CzIPN,^[22]^ provided **5 a** in identical yield to that obtained with the optimum iridium photocatalyst. Finally, a control experiment demonstrated that light was necessary for the reaction (entry 5).

**Table 1:** Optimization of the bromodecarboxylation.^[a]^

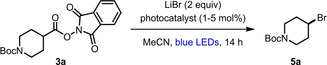			
Entry	Photocatalyst (mol %)	Conversion [%]	Yield [%]
1	none	5	5
2	Ir(ppy)_3_ (1)	100	74
3	**[Ir‐1]** (1)	100	93
4	4CzIPN (5)	100	93
5^[b]^	4CzIPN (5)	0	0

[a] The reactions were performed with **3 a** (0.10 mmol) and LiBr (2 equiv) in MeCN (1 mL). The blue LEDs are the integrated photoreactor described in Ref. [21]. Yields and conversions were determined by GC analysis using an internal standard. [b] Reaction performed in the dark. **[Ir‐1]**=Ir[dF(CF_3_)ppy]_2_(dtbbpy)PF_6_.

**Scheme 2:**
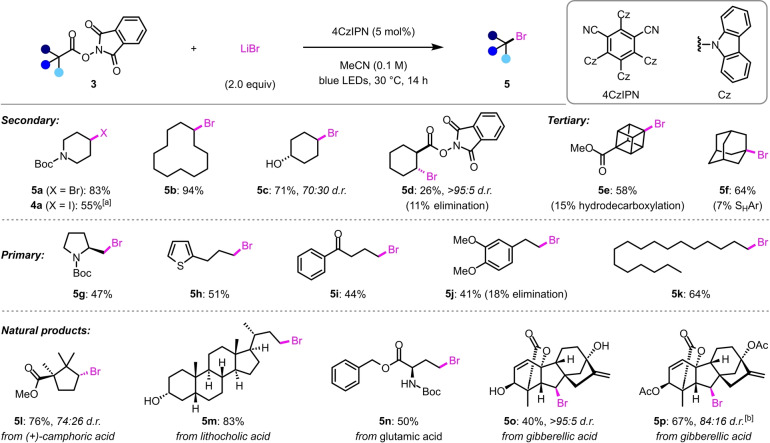
Scope of the bromodecarboxylation reaction. Reaction conditions: **3** (0.2 mmol, 1 equiv), 4CzIPN (5 mol %), and LiBr (2 equiv), in MeCN (2 mL), N_2_ atmosphere, blue LEDs, 28–30 °C, 14 h. Yields are of the isolated products after chromatographic purification. Diastereomeric ratios were determined by ^1^H NMR analysis of the purified products. [a] Using NaI (2 equiv) instead of LiBr. [b] The diastereomeric ratio was determined from the yields of the separated diastereomers.

With the optimized conditions in hand, we investigated the scope of the decarboxylative bromination (Scheme 2). Alkyl bromides **5** were accessed in good yields for a wide range of NHP esters, including secondary (**5 a**–**5 d**), tertiary (**5 e**, **5 f**), and primary substrates (**5 g**–**5 k**). Notably, substrates containing readily oxidizable alcohols (**5 c**) or electron‐rich aromatic rings (**5 h**, **5 j**) were tolerated, whereas, such functional groups are likely incompatible with previously reported bromodecarboxylations that employ strongly oxidizing/electrophilic brominating agents.[^12a^, ^15c^, ^16b^] The cyclohexane‐1,2‐dicarboxylate substrate **3 d** gave the mono‐bromination product **5 d** in only 26 % yield, together with 11 % of the alkene formed by subsequent elimination of HBr. No 1,2‐dibromocyclohexane was observed, likely due to elimination of the intermediate β‐bromo radical to form cyclohexene.^[23]^ Interestingly, **5 d** was formed as a single *trans* diastereomer, which we attribute to selective elimination of the *cis* isomer. Elimination was also observed as a competing reaction during the synthesis of homobenzylic bromide **5 j**. The bromination of the cubane NHP ester **3 e** was accompanied by the corresponding hydrodecarboxylation product, whereas adamantane substrate **3 f** underwent competitive homolytic aromatic substitution (S_H_Ar) with phthalimide (see Supporting Information for details); a pathway that was found to outcompete the desired iododecarboxylation under the conditions reported by Chen.^[19]^ The excellent functional‐group tolerance of these mild reaction conditions also enabled the preparation of alkyl bromides from a number of complex natural product derivatives, including camphoric acid (**5 l**), lithocholic acid (**5 m**), glutamic acid (**5 n**), and gibberellic acid (**5 o**, **5 p**). Notably, the gibberellic acid substrate **3 o**, with two free alcohols and two alkenes, afforded bromide **5 o** in 40 % yield as a single diastereomer.^[24]^ Finally, we tested the suitability of these conditions for iodination by using NaI in place of LiBr, which provided alkyl iodide **4 a** in 55 % yield. This result demonstrates the generality of these halogenation conditions; however, we did not investigate the iodination reaction further because it is comparable to the PPh_3_‐catalyzed process reported by Shang and co‐workers.^[20b]^

Next, we turned our attention to the corresponding decarboxylative chlorination of NHP ester **3 a** (Table 2). Our initial attempts to simply replace LiBr with LiCl under the conditions optimized for bromination demonstrated that chlorodecarboxylation was indeed possible, however, low yields of the corresponding alkyl chloride **6 a** were obtained due to the competitive formation of hydrodecarboxylation product **8 a** (entry 1), which was also observed for a range of different photocatalysts and chloride sources (see Table S3 in the Supporting Information for further details). Inspired by a recent report by the Zou group on Cu^I^‐catalyzed chlorination of alkyl radicals,^[25]^ we investigated the effect of catalytic copper additives in our decarboxylative process. Pleasingly, the yield of **6 a** increased to 54 % upon adding 20 mol % CuCl, and no hydrodecarboxylation product **8 a** was observed, however, a significant amount of dehydrodecarboxylation occurred to form alkene **9 a** (entry 2).^[26]^ The formation of **9 a** prompted us to investigate the addition of ligands to prevent the elimination pathway and promote chlorination.^[27]^ Fortunately, including 20 mol % of 4,4′‐di‐*tert*‐butyl‐2,2′‐bipyridyl (dtbbpy) prevented the undesired dehydrodecarboxylation and gave a dramatically enhanced yield of **6 a** of 90 % (entry 3). We found that replacing CuCl with air stable CuCl_2_ further improved the yield (entry 4). A slightly lower yield of **6 a** was obtained using 2,9‐dimethyl‐1,10‐phenanthroline (dmp) as the ligand (entry 5), however, these conditions were found to be optimal due to the unexpected sensitivity of the dtbbpy conditions to reaction scale. Finally, although **[Ir‐1]** was found to be the most effective photocatalyst for this transformation, **6 a** could also be obtained in high yield using 4CzIPN (see Table S2).

**Table 2:** Optimization of the chlorodecarboxylation.^[a]^

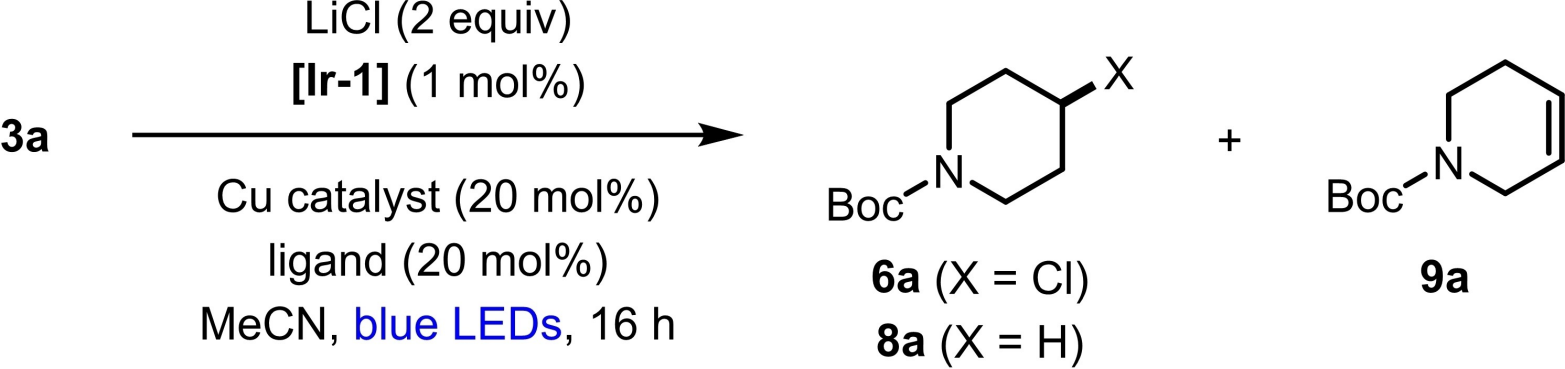					
Entry	Cu catalyst	Ligand	**6 a** [%]	**8 a** [%]	**9 a** [%]
1^[b]^	none	none	15	17	0
2^[b]^	CuCl	none	54	0	20
3	CuCl	dtbbpy	90	0	0
4	CuCl_2_	dtbbpy	96	0	0
5	CuCl_2_	dmp	92	0	0

[a] The reactions were performed with **3 a** (0.10 mmol) and LiCl (2 equiv) in MeCN (1 mL). The blue LEDs are the integrated photoreactor described in Ref. [21]. Yields and conversions were determined by ^1^H NMR analysis using an internal standard. [b] Using 3 equivalents of LiCl. **[Ir‐1]**=Ir[dF(CF_3_)ppy]_2_(dtbbpy)PF_6_; dtbbpy=4,4′‐di‐*tert*‐butyl‐2,2′‐bipyridyl; dmp=2,9‐dimethyl‐1,10‐phenanthroline.

**Scheme 3:**
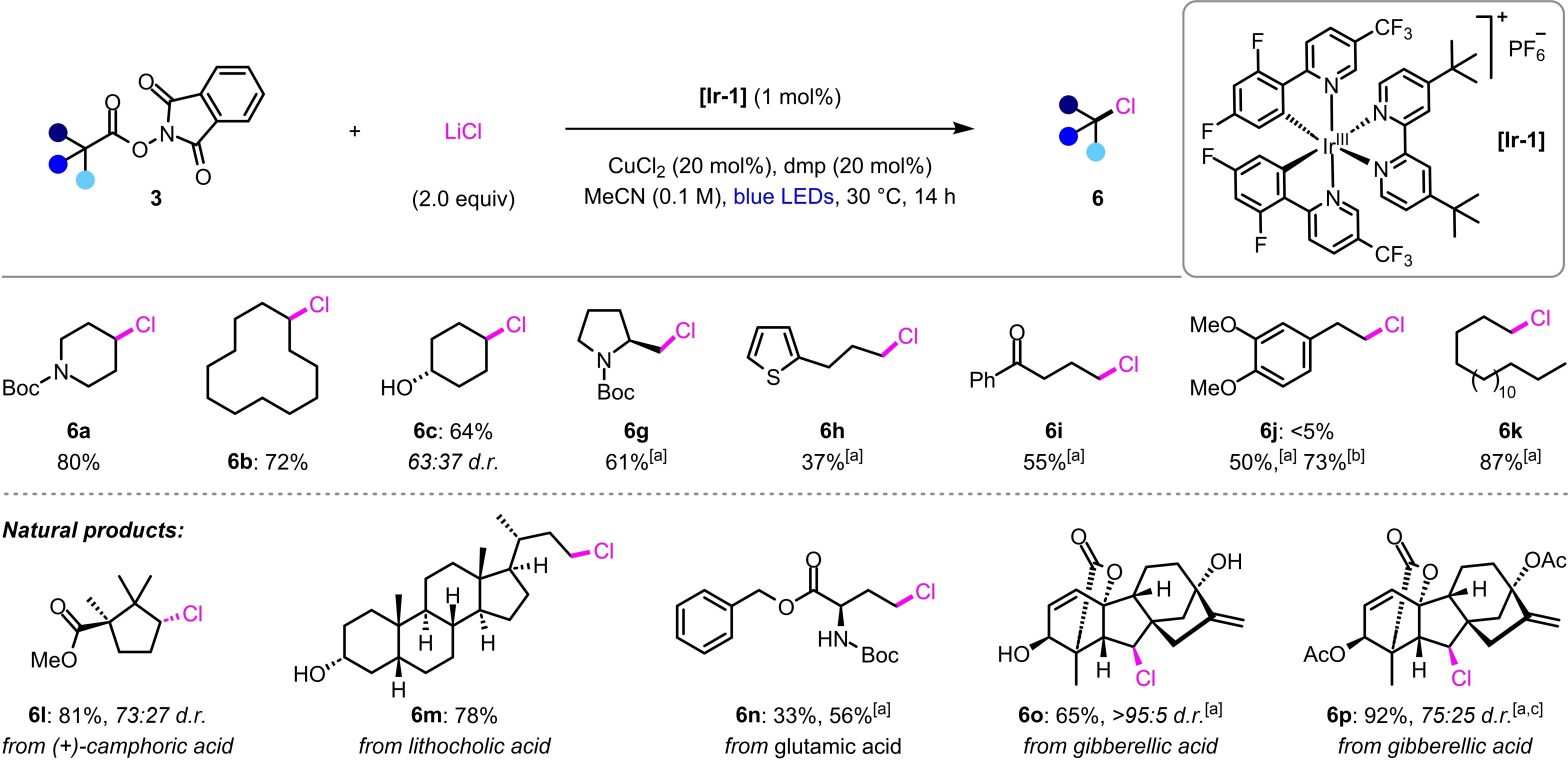
Scope of the chlorodecarboxylation reaction. Reaction conditions: **3** (0.2 mmol, 1 equiv), Ir[dF(CF_3_)ppy]_2_(dtbbpy)PF_6_, (1 mol %), LiCl (2 equiv), CuCl_2_ (20 mol %), and dmp (20 mol %) in MeCN (2 mL), N_2_ atmosphere, blue LEDs, 28–30 °C, 14 h. Yields are of the isolated products after chromatographic purification. Diastereomeric ratios were determined by ^1^H NMR analysis of the purified products. [a] Using MeCN/EtOAc (1 : 1, 0.1 M) as the solvent. [b] Using acetone (0.1 M) as the solvent. [c] The diastereomeric ratio was determined from the yields of the separated diastereomers.

Next, we explored the scope of this dual photoredox‐copper‐catalyzed chlorodecarboxylation (Scheme 3). Similar reaction efficiencies to the bromination protocol were observed for a range of secondary NHP esters (**3 a**–**3 c**, **3 l**, **3 o**–**3 p**). Unexpectedly, translation of these conditions to primary substrates gave poor yields of the corresponding alkyl chlorides (**6 g**–**6 k**, **6 n**), however, dramatic improvements in reaction efficiencies were obtained upon switching to a mixed solvent system of MeCN/EtOAc (1 : 1). Acetone was also found to be a competent solvent, providing a further enhancement in yield of chloride **6 j**. As with the bromination reaction, excellent functional group tolerance was observed, including free alcohols (**6 c**, **6 m**, **6 o**), electron‐rich aromatic rings (**6 h**, **6 j**), and alkenes (**6 o**–**6 p**).

Given the versatility of copper catalysis for introducing diverse functional groups to alkyl radicals,[^1d^, ^28^] we proceeded to investigate whether these dual photoredox‐copper‐catalyzed decarboxylations could be extended beyond halogenation reactions. Organic thiocyanates are useful functional groups that are widely used in synthesis and they are also found in bioactive natural products.[^29^, ^30^] Whilst there are numerous methods available for the synthesis of alkyl thiocyanates,^[31]^ including via radical pathways,^[32]^ decarboxylative thiocyanations are rare.[^33^, ^34^] In 1992, Barton and co‐workers reported the only example of a decarboxylative thiocyanation of alkyl carboxylic acids.^[33]^ They found that reaction of thiohydroxamate esters with mesyl or tosyl isothiocyanates under visible‐light irradiation generated the corresponding alkyl thiocyanates. However, this method relies on the use of unstable reagents and electrophilic thiocyanate sources. A more attractive approach would be to use simple inorganic thiocyanate salts, however, the only examples of their use in decarboxylative thiocyanations are limited to reactions of cinnamic acids, thus, they are unsuitable for alkyl carboxylic acids.^[34]^ Based on previous reports of the use of copper catalysis in thiocyanations of alkyl radicals with nucleophilic thiocyanate reagents,^[35]^ we reasoned that decarboxylative thiocyanations of NHP esters **3** would be readily accomplished using our dual photoredox‐copper‐catalyzed conditions. Gratifyingly, with only minor modifications to our chlorodecarboxylation conditions, including switching LiCl to KSCN and CuCl_2_ to CuSCN, decarboxylative thiocyanation of NHP ester **3 a** gave alkyl thiocyanate **7 a** in 74 % NMR yield (see Table S4). Furthermore, the yield could be improved upon changing the solvent from MeCN to acetone, which provided **7 a** in nearly quantitative yield. Importantly, this process was also effectively catalyzed by the inexpensive photocatalyst 4CzIPN, although **7 a** was obtained in a lower yield of 72 %.

**Scheme 4:**
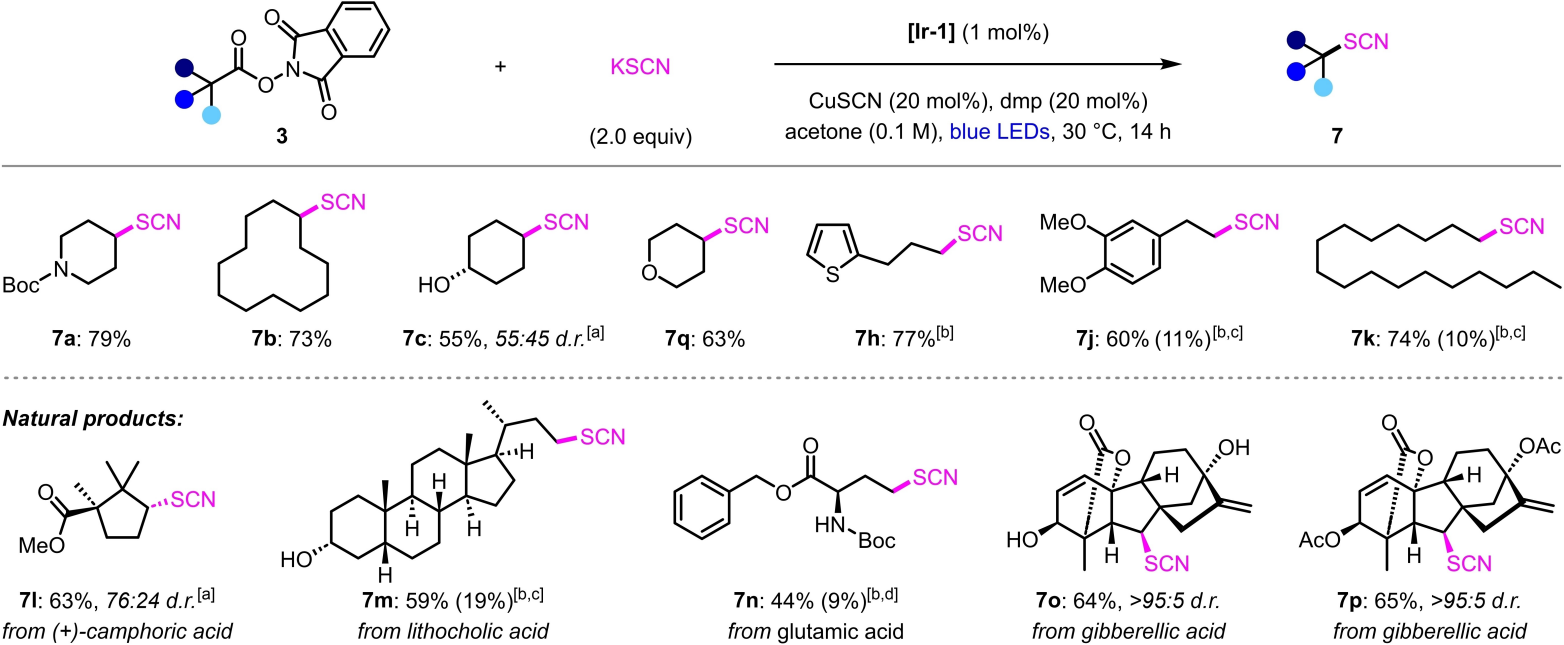
Scope of the decarboxylative thiocyanation reaction. Reaction conditions: **3** (0.2 mmol, 1 equiv), Ir[dF(CF_3_)ppy]_2_(dtbbpy)PF_6_ (1 mol %), KSCN (2 equiv), CuSCN (20 mol %), and dmp (20 mol %) in acetone (2 mL), N_2_ atmosphere, blue LEDs, 28–30 °C, 14 h. Yields are of the isolated products after chromatographic purification. Diastereomeric ratios were determined by ^1^H NMR analysis of the purified products. [a] The diastereomeric ratio was determined from the yields of the separated diastereomers. [b] Using TMSNCS (2 equiv) instead of KSCN. [c] The yield in parentheses is of the isothiocyanate product. [d] The yield in parentheses is of the hydrodecarboxylation product.

We subsequently investigated the scope of the decarboxylative thiocyanation (Scheme 4). A range of secondary NHP esters reacted efficiently to give the corresponding alkyl thiocyanates in moderate to good yields (**7 a**–**7 c**, **7 l**, **7 o**–**7 q**). However, low yields were obtained for primary substrates, with significant amounts of competing hydrolysis of the NHP esters **3** observed. We hypothesized that the contrasting results for primary and secondary substrates resulted from the faster rate of hydrolysis of the less hindered primary NHP esters by the nucleophilic thiocyanate anion, which outcompetes radical decarboxylation. To circumvent this problem, we exchanged KSCN for the less nucleophilic trimethylsilyl isothiocyanate (TMSNCS).^[35]^ With these modified conditions, primary thiocyanates **7 h**, **7 j**–**7 k**, and **7 m**–**7 n** were obtained in good yields. Once again, free alcohols (**7 c**, **7 m**, **7 o**), electron‐rich aromatic rings (**7 h**, **7 j**), and alkenes (**7 o**–**7 p**) were well tolerated. It should be noted that for primary substrates, the thiocyanate product was always accompanied by small amounts of the corresponding isothiocyanate, but this was formed in significant quantities for some substrates (**3 j**, **3 k**, and **3 m**). However, these isomeric products were readily separated by column chromatography, which allowed the isolation of the pure thiocyanates.

**Scheme 5:**
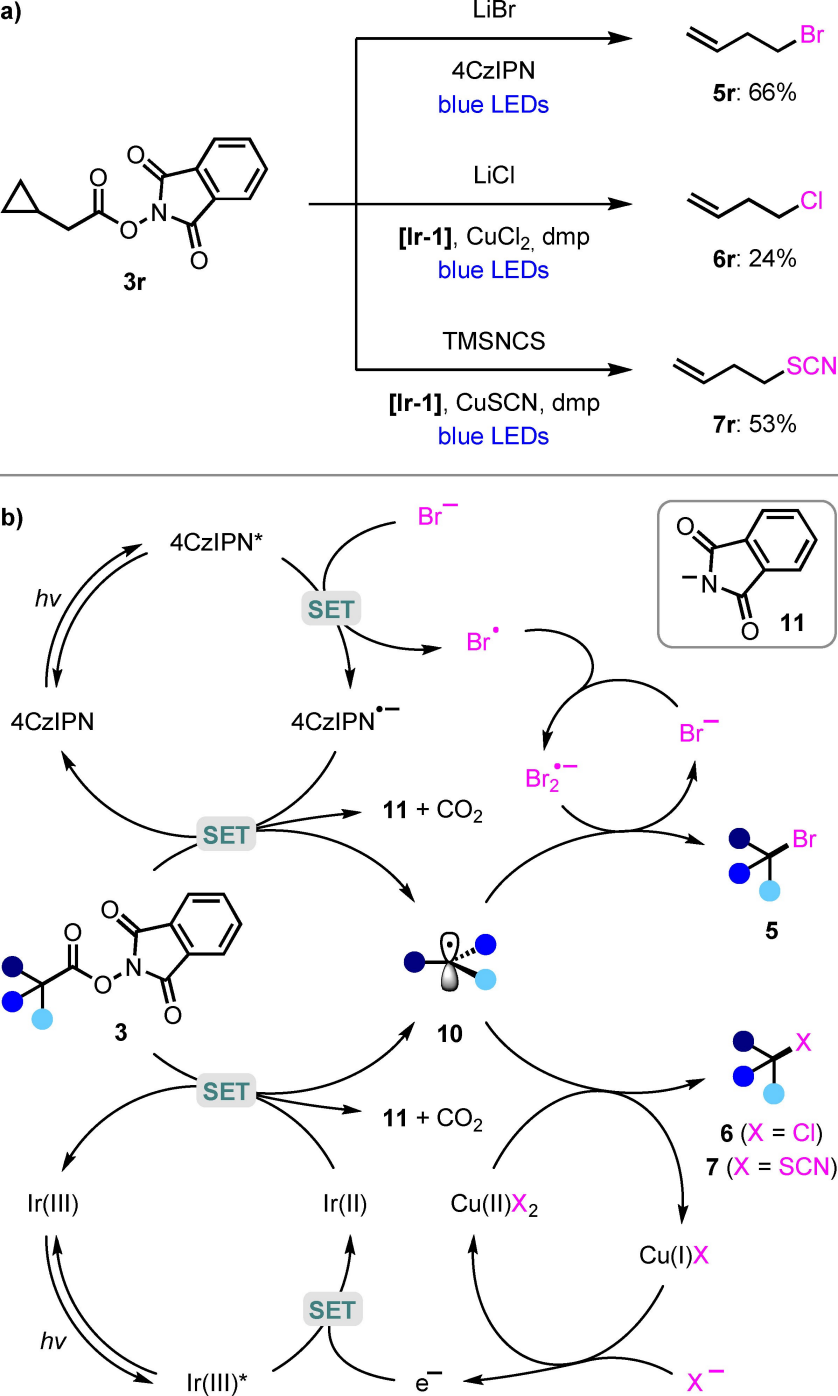
(a) Mechanistic studies and (b) proposed mechanisms.

To gain insight into the mechanism of the photoredox‐catalyzed decarboxylation reactions, radical clock experiments were carried out with cyclopropyl methyl NHP ester **3 r** (Scheme 5a). Butenyl derivatives **5 r**, **6 r**, and **7 r** were obtained as the only observed products for the bromination, chlorination, and thiocyanation reactions, respectively, thereby confirming the formation of an alkyl radical intermediate that undergoes rapid cyclopropane ring‐opening. For each decarboxylative functionalization reaction, we propose that single‐electron reduction of NHP ester **3** by the photocatalyst results in N−O bond cleavage and decarboxylation to generate alkyl radical **10** and the phthalimide anion **11** (Scheme 5b). Given the large negative reduction potential of **3** (*E*_p_ [**3 a**/**3** ***a*** ⋅^−^]=−1.2 V vs SCE in MeCN),^[36]^ oxidative quenching of the excited state photocatalysts by **3** is endergonic (*E*_1/2_ [Ir^IV^/Ir^III^*]=−0.89 V and *E*_1/2_ [4CzIPN⋅^**+**^/4CzIPN*]=−1.04 V, both vs SCE in MeCN),^[37]^ therefore, initial reductive quenching of the photocatalyst followed by single‐electron transfer (SET) between **3** and the reduced state photocatalysts (*E*_1/2_ [Ir^III^/Ir^II^]=−1.37 and *E*_1/2_ [4CzIPN/4CzIPN⋅^−^]=−1.21 V)^[37]^ is likely the favored pathway.

For the bromodecarboxylation, reductive quenching of excited state 4CzIPN (*E*_1/2_ [4CzIPN*/4CzIPN⋅^−^]=1.35 V vs SCE in MeCN)^[37]^ by a bromide anion (*E*_p_ [Br⋅/Br^−^]=0.80 V vs SCE in DME)^[38]^ to form a bromine radical is thermodynamically favored (Scheme 5b). The subsequent trapping of alkyl radical **10** to form the C−Br bond could occur via several different pathways. Firstly, direct radical‐radical coupling between **10** and the bromine radical (Br⋅) could occur.^[39]^ However, in the iododecarboxylation reactions reported by Chen and Shang,^[20]^ they proposed that Lewis base additives played a crucial role in stabilizing iodine radicals to enable subsequent reaction with alkyl radicals. In our protocol, given the absence of Lewis base additives, we propose that the bromine radical is stabilized by excess bromide through the generation of the dibromide radical anion (Br_2_⋅^−^), which has been reported to occur with a rate of 1.1×10^10^ M^−1^ s^−1^.^[40]^ Therefore, C−Br bond formation to generate alkyl bromide **5** likely occurs by reaction of **10** with Br_2_⋅^−^. A third possible pathway involves the dimerization of two bromine radicals to generate Br_2_ as the brominating agent,^[39b]^ although this pathway is unlikely given the successful bromodecarboxylation of substrates containing alkenes (**5 o**, **5 p**), which could react with the strongly oxidizing and electrophilic Br_2_.

For the dual photoredox‐copper catalyzed chlorination and thiocyanation reactions, reductive quenching of excited state **[Ir‐1]** (*E*_1/2_ [Ir^III^*/Ir^II^]=1.21 V vs SCE in MeCN)^[37]^ likely occurs by SET with the Cu^I^X catalyst (Scheme 5b), (since this should be a highly exergonic process (*E*_1/2_ [CuCl_2_/CuCl_2_^−^]=0.47 V vs SCE in MeCN).^[41]^ For the chlorination reaction, which uses Cu^II^Cl_2_ as the catalyst, reductive quenching by lithium chloride (*E*_p/2_ [Cl⋅/Cl^−^]=1.00 V vs SCE in MeCN for NEt_4_Cl)^[42]^ is also a possibility. After SET transfer between the reduced photocatalyst and **3**, C−X bond formation occurs upon reaction of alkyl radical **10** with Cu^II^X_2_ to give alkyl chloride **6** or thiocyanate **7** and regenerate Cu^I^X. This could proceed via both an inner‐sphere pathway, involving radical addition to Cu^II^X_2_ and reductive elimination of the resulting alkyl‐Cu^III^X_2_ species; or an outer‐sphere pathway, involving atom transfer.[^27a^, ^35^, ^43^]

## Conclusion

In conclusion, we have developed visible‐light photoredox‐catalyzed protocols for the decarboxylative bromination, chlorination, and thiocyanation of NHP esters. These redox neutral transformations proceed efficiently with inexpensive inorganic salts (LiBr, LiCl, and KSCN), thus avoiding the requirement for stoichiometric oxidants or strongly electrophilic reagents. Simple, transition‐metal‐ and additive‐free bromination conditions were identified, wherein C−Br bond formation occurs through the reaction of alkyl radicals with a dibromide radical anion. Alternatively, the construction of C−Cl and C−SCN bonds was accomplished using dual photoredox‐copper catalysis.^[44]^ The mild conditions displayed excellent functional‐group tolerance, allowing the conversion of a diverse range of structurally complex carboxylic acids into the corresponding alkyl bromides, chlorides and thiocyanates.

## Conflict of interest

The authors declare no conflict of interest.

1

## Supporting information

As a service to our authors and readers, this journal provides supporting information supplied by the authors. Such materials are peer reviewed and may be re‐organized for online delivery, but are not copy‐edited or typeset. Technical support issues arising from supporting information (other than missing files) should be addressed to the authors.

Supporting Information

## Data Availability

The data that support the findings of this study are available in the supplementary material of this article.
