# Supplementary material for: Photoredox‐Catalyzed Decarboxylative Bromination, Chlorination and Thiocyanation Using Inorganic Salts
Source: Angew Chem Int Ed Engl. 2023 Aug 10;62(38):e202309684. doi: 10.1002/anie.202309684 (PMC10952529; doi:10.1002/anie.202309684)
Supplement: Supplementary file 1 — Supporting Information [file ANIE-62-0-s001.pdf]

Angewandte Chemie
Eine Zeitschrift der Gesellschaft Deutscher Chemiker

Supporting Information

Photoredox-Catalyzed Decarboxylative Bromination, Chlorination and Thiocyanation Using Inorganic Salts

J. Wu, C. Shu, Z. Li, A. Noble, V. K. Aggarwal**

TABLE OF CONTENTS

LIST OF SUPPLEMENTARY SCHEMES, FIGURES AND TABLES 2

LIST OF CHARACTERISED PRODUCTS 2

1. MATERIALS AND GENERAL METHODS 4
   - 1.1. Glassware, Solvents and Reagents 4
   - 1.2. Chromatography and Instrumentation 4
   - 1.3. Naming of Compounds 4
   - 1.4. Photochemical Equipment 5
2. EXPERIMENTAL DATA 6
   - 2.1. General Procedures 6
     - 2.1.1. General Procedure 1 (GP1): Synthesis of redox active esters 6
     - 2.1.2. General Procedure 2 (GP2): Decarboxylative bromination 6
     - 2.1.3. General Procedure 3 (GP3): Decarboxylative chlorination 6
     - 2.1.4. General Procedure 4 (GP4): Decarboxylative thiocyanation 7
   - 2.2. Synthesis of Redox Active Esters 8
     - 2.2.1. Previously reported substrates 8
     - 2.2.2. Synthesised substrates 9
   - 2.3. Optimisation Studies 14
     - 2.3.1. Decarboxylative Bromination 14
     - 2.3.2. Decarboxylative Chlorination 15
     - 2.2.3. Decarboxylative Thiocyanation 16
   - 2.4. Decarboxylative Iodination 17
   - 2.5. Decarboxylative Bromination 18
   - 2.6. Decarboxylative Chlorination 29
   - 2.7. Decarboxylative Thiocyanation 38
   - 2.8. Unsuccessful Decarboxylative Functionalisations 48
     - 2.8.1. Fluorination 48
     - 2.8.2. Azidation 48
3. MECHANISTIC STUDIES 49
   - 3.1. Radical Clock Experiments 49
     - 3.1.1. Decarboxylative Bromination 49
     - 3.1.2. Decarboxylative Chlorination 50
     - 3.1.3. Decarboxylative Thiocyanation 51
   - 3.2. Response to a Reviewer’s Comment on C(sp^3)–H Bromination 52
4. SPECTROSCOPIC DATA 53
5. REFERENCES 128

LIST OF SUPPLEMENTARY SCHEMES, FIGURES AND TABLES

Figure S1. Photochemical reaction setup 5
Table S1: Redox active esters prepared according to our previous report 8
Table S2: Optimisation of the decarboxylative bromination 14
Table S3: Optimisation of the decarboxylative chlorination 15
Table S4: Optimisation of the decarboxylative thiocyanation 16

LIST OF CHARACTERISED PRODUCTS

1,3-Dioxoisoindolin-2-yl (1*r*,4*r*)-4-hydroxycyclohexane-1-carboxylate (**3c**) 9
1,3-Dioxoisoindolin-2-yl 4-(thiophen-2-yl)butanoate (**3h**) 9
1,3-Dioxoisoindolin-2-yl 5-oxo-5-phenylpentanoate (**3i**) 10
1,3-Dioxoisoindolin-2-yl 3-(3,4-dimethoxyphenyl)propanoate (**3j**) 10
3-(1,3-Dioxoisoindolin-2-yl) 1-methyl (1*R*,3*S*)-1,2,2-trimethylcyclopentane-1,3-dicarboxylate (**3l**) 11
1-Benzyl 5-(1,3-dioxoisoindolin-2-yl) (*tert*-butoxycarbonyl)-*D*-glutamate (**3n**) 12
Gibberellic acid redox active ester (**3o**) 12
tert-Butyl 4-iodopiperidine-1-carboxylate (**4a**) 17
tert-Butyl 4-bromopiperidine-1-carboxylate (**5a**) 18
Bromocyclododecane (**5b**) 18
(1*r*,4*r*)-4-Bromocyclohexan-1-ol (**5c**) 19
1,3-Dioxoisoindolin-2-yl (1*S**,2*R**)-2-bromocyclohexane-1-carboxylate (**5d**) 19
Methyl (1*r*,2*R*,3*r*,4*s*,8*S*)-4-bromocubane-1-carboxylate (**5e**) 20
(3*s*,5*s*,7*s*)-1-Bromoadamantane (**5f**) 21
tert-Butyl (*S*)-2-(bromomethyl)pyrrolidine-1-carboxylate (**5g**) 22
2-(3-Bromopropyl)thiophene (**5h**) 22
4-Bromo-1-phenylbutan-1-one (**5i**) 23
4-(2-Bromoethyl)-1,2-dimethoxybenzene (**5j**) 23
1-Bromoheptadecane (**5k**) 24
Methyl (1*R*)-3-bromo-1,2,2-trimethylcyclopentane-1-carboxylate (**5l**) 24
((3*R*,5*R*,8*R*,9*S*,10*S*,13*R*,14*S*,17*R*)-17-((*R*)-4-Bromobutan-2-yl)-10,13-dimethylhexadecahydro-1*H*-cyclopenta[*a*]phenanthren-3-ol (**5m**) 25
Benzyl (*R*)-4-bromo-2-((*tert*-butoxycarbonyl)amino)butanoate (**5n**) 26
(1*S*,2*S*,4a*R*,4b*R*,7*S*,9a*S*,10*S*,10a*R*)-10-Bromo-2,7-dihydroxy-1-methyl-8-methylene-1,2,4b,5,6,7,8,9,10,10a-decahydro-4a,1-(epoxymethano)-7,9a-methanobenzo[*a*]azulen-13-one (**5o**) 26
(1*S*,2*S*,4a*R*,4b*R*,7*S*,9a*S*,10*S*,10a*R*)-10-Bromo-1-methyl-8-methylene-13-oxo-1,2,5,6,8,9,10,10a-octahydro-4a,1-(epoxymethano)-7,9a-methanobenzo[a]azulene-2,7(4b*H*)-diyl diacetate (**5p**) 27
tert-Butyl 4-chloropiperidine-1-carboxylate (**6a**) 29
Chlorocyclododecane (**6b**) 29
(1*r*,4*r*)-4-Chlorocyclohexan-1-ol (**6c**) 30
tert-Butyl (*S*)-2-(chloromethyl)pyrrolidine-1-carboxylate (**6g**) 30
2-(3-Chloropropyl)thiophenecarboxylate (**6h**) 31
4-Chloro-1-phenylbutan-1-one (**6i**) 31
4-(2-Chloroethyl)-1,2-dimethoxybenzene (**6j**) 32
1-Chloroheptadecane (**6k**) 33

Methyl (1*R*)-3-chloro-1,2,2-trimethylcyclopentane-1-carboxylate (**6l**) 33
((3*R*,5*R*,8*R*,9*S*,10*S*,13*R*,14*S*,17*R*)-17-((*R*)-4-Chlorobutan-2-yl)-10,13-dimethylhexadecahydro-1*H*-cyclopenta[*a*]phenanthren-3-ol (**6m**) 34
Benzyl (*R*)-2-((*tert*-butoxycarbonyl)amino)-4-chlorobutanoate (**6n**) 35
(1*S*,2*S*,4a*R*,4b*R*,7*S*,9a*S*,10*S*,10a*R*)-10-Chloro-2,7-dihydroxy-1-methyl-8-methylene-1,2,4b,5,6,7,8,9,10,10a-decahydro-4a,1-(epoxymethano)-7,9a-methanobenzo[*a*]azulen-13-one (**6o**) 35
(1*S*,2*S*,4a*R*,4b*R*,7*S*,9a*S*,10*S*,10a*R*)-10-Chloro-1-methyl-8-methylene-13-oxo-1,2,5,6,8,9,10,10a-octahydro-4a,1-(epoxymethano)-7,9a-methanobenzo[*a*]azulene-2,7(4b*H*)-diyl diacetate (**6p**) 36
tert-Butyl 4-thiocyanatopiperidine-1-carboxylate (**7a**) 38
Thiocyanatocyclododecane (**7b**) 38
4-Thiocyanatocyclohexan-1-ol (**7c**) 39
2-(3-Chloropropyl)thiophene (**7h**) 40
1,2-Dimethoxy-4-(2-thiocyanatoethyl)benzene (**7j**) 40
1-Thiocyanatoheptadecane (**7k**) 41
Methyl (1*R*,3*R*)-1,2,2-trimethyl-3-thiocyanatocyclopentane-1-carboxylate (**7l**) 42
(3*R*,5*R*,8*R*,9*S*,10*S*,13*R*,14*S*,17*R*)-10,13-Dimethyl-17-((*R*)-4-thiocyanatobutan-2-yl)hexadecahydro-1*H*-cyclopenta[*a*]phenanthren-3-ol (**7m**) 43
Benzyl *N*-(*tert*-butoxycarbonyl)-*S*-cyano-*D*-homocysteinate (**7n**) 44
(1*S*,2*S*,4a*R*,4b*R*,7*S*,9a*S*,10*S*,10a*R*)-2,7-Dihydroxy-1-methyl-8-methylene-10-thiocyanato-1,2,4b,5,6,7,8,9,10,10a-decahydro-4a,1-(epoxymethano)-7,9a-methanobenzo[*a*]azulen-13-one (**7o**) 45
(1*S*,2*S*,4a*R*,4b*R*,7*S*,9a*S*,10*S*,10a*R*)-1-Methyl-8-methylene-13-oxo-10-thiocyanato-1,2,5,6,8,9,10,10a-octahydro-4a,1-(epoxymethano)-7,9a-methanobenzo[*a*]azulene-2,7(4b*H*)-diyl diacetate (**7p**) 46
4-Thiocyanatotetrahydro-2*H*-pyran (**7q**) 46

1. MATERIALS AND GENERAL METHODS

1.1. Glassware, Solvents and Reagents

All manipulations were performed with oven-dried (130 °C for a minimum of 12 h) glassware using standard Schlenk techniques under an atmosphere of nitrogen, unless otherwise stated.

All anhydrous solvents were commercially supplied from Sigma-Aldrich (acetone, methanol) or dried using an Anhydrous Engineering alumina column drying system [DCM (dichloromethane), acetonitrile (MeCN), toluene]. Water is deionised and brine refers to a saturated aqueous solution of NaCl. Reagents were purchased from commercial sources and used as received.

1.2. Chromatography and Instrumentation

Thin layer chromatography (TLC) was performed using Merck Kieselgel 60 F254 fluorescent treated silica, which was visualised under UV light, or by staining with aqueous basic potassium permanganate followed by heating, an ethanolic solution of phosphomolybdic acid followed by heating, or an ethanolic solution of ninhydrin followed by heating.

Flash column chromatography (FCC) was carried out using Sigma-Aldrich silica gel (60 Å, 230-400 mesh, 40-63 μm) or a Biotage Isolera™ flash purification system. In cases where automated column chromatography was employed the solvent gradient and flow rate are indicated.

NMR spectra were recorded at various field strengths, as indicated, using Bruker 400 MHz, Varian VNMR 400 MHz, Varian VNMR 500 MHz, or Bruker Cryo 500 MHz for ^{1}H and ^{13}C acquisitions. All NMR spectra were recorder at 25 °C unless otherwise stated. Chemical shifts (δ) are reported in parts per million (ppm) and referenced to $CDCl_3$ (^{1}H: 7.26 ppm; ^{13}C: 77.0 ppm). Coupling constants (*J*) are given in Hertz (Hz) and refer to apparent multiplicities (s = singlet, d = doublet, t = triplet, q = quartet, quin = quintet, hex = hextet, h = heptet, m = multiplet, br = broad signal, dd = doublet of doublets, etc.). The ^{1}H NMR spectra are reported as follows: chemical shift (multiplicity, coupling constants, number of protons)

High resolution mass spectra (HRMS) were recorded on a Bruker Daltonics MicrOTOF II by Electrospray Ionisation (ESI); a Thermo Scientific QExactive by Electron Ionisation (EI); or a Thermo Scientific Orbitrap Elite by ESI or Atmospheric Pressure Chemical Ionisation (APCI).

IR spectra were recorded neat as a thin film on a Perkin Elmer Spectrum One FT-IR equipped with an ATR sampling accessory. Selected absorption maxima (ν_{max}) are reported in wavenumbers (cm^{-1}).

1.3. Naming of Compounds

Compound names are those generated by ChemDraw Professional 20.0 software (PerkinElmer), following the IUPAC nomenclature.

1.4. Photochemical Equipment

The blue LEDs were either the Penn OC Photoreactor M1 (purchased from www.pennoc.com, discontinued) or the Penn PhD Photoreactor M2 (purchased from www.sigmaaldrich.com) equipped with 450 nm LEDs.

Reaction mixtures were prepared under a nitrogen atmosphere in 7 mL vials before sealing with parafilm and placing in the photoreactor (Figure S1). The LED intensity was set to 100% and the stirring speed set to 500 rpm. The fan speed was set to 4500 rpm to maintain a temperature of 30–35 °C.

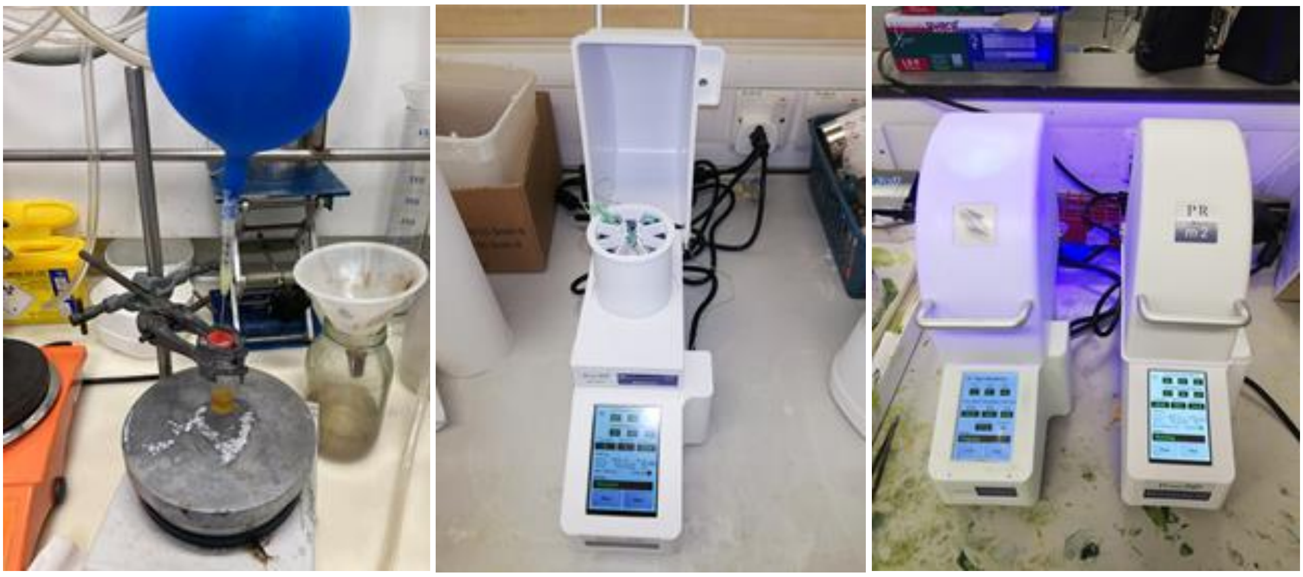

Figure S1. Photochemical reaction setup

2. EXPERIMENTAL DATA

2.1. General Procedures

2.1.1. General Procedure 1 (GP1): Synthesis of redox active esters

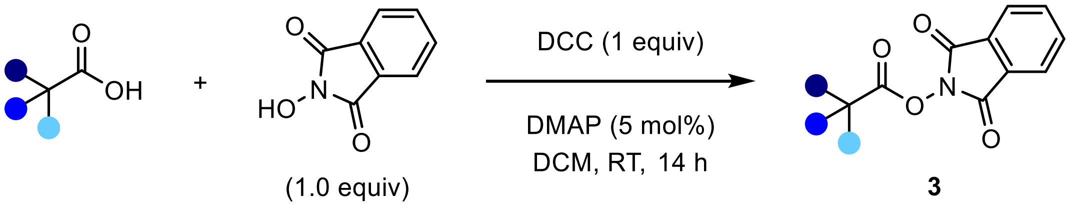

To a solution of carboxylic acid **1** (2.0 mmol) and *N*-hydroxyphthalimide (2.0 mmol, 1.0 equiv.) in DCM (10 mL) was added 4-(dimethylamino)pyridine (DMAP) (0.10 mmol, 5.0 mol%) followed by *N*,*N'*-dicyclohexylcarbodiimide (DCC) (2.0 mmol, 1.0 equiv.). The reaction was stirred at room temperature for 14 h, after which TLC analysis showed complete conversion. The reaction was concentrated under reduced pressure and purified by flash column chromatography to give redox active ester **3**.

2.1.2. General Procedure 2 (GP2): Decarboxylative bromination

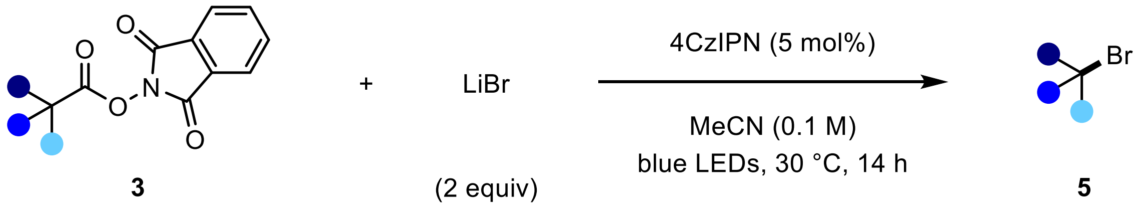

An oven-dried vial (7 mL) was charged with redox active ester **3** (0.20 mmol, 1.0 equiv.), 4CzIPN (7.9 mg, 0.010 mmol, 5 mol%), and LiBr (35 mg, 0.40 mmol, 2.0 equiv.). The vial was sealed with a septum and placed under an N_2 atmosphere before adding anhydrous MeCN (2.0 mL). The mixture was subsequently degassed by sparging with N_2 for 1 min. The reaction was stirred under blue LED irradiation in the photoreactor for 14 h, after which TLC analysis showed complete conversion. The reaction was concentrated under reduced pressure and purified by flash column chromatography to give alkyl bromide **5**.

2.1.3. General Procedure 3 (GP3): Decarboxylative chlorination

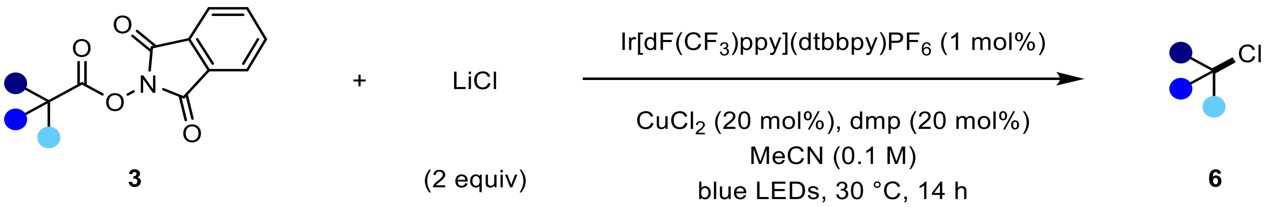

An oven-dried vial (7 mL) was charged with redox active ester **3** (0.20 mmol, 1.0 equiv.), $Ir[dF(CF_3)ppy)_2(dtbbpy)PF_6$ (2.2 mg, 0.0020 mmol, 1.0 mol%), $CuCl_2$ (5.4 mg, 0.040 mmol, 20 mol%), 2,9-

dimethyl-1,10-phenanthroline (dmp) (8.3 mg, 0.040 mmol, 20 mol%), and LiCl (17 mg, 0.40 mmol, 2.0 equiv.). The vial was sealed with a septum and placed under an N_2 atmosphere before adding anhydrous MeCN (1.0 mL). The mixture was subsequently degassed by sparging with N_2 for 1 min. The reaction was stirred under blue LED irradiation in the photoreactor for 14 h, after which TLC analysis showed complete conversion. The reaction was concentrated under reduced pressure and purified by flash column chromatography to give alkyl chloride **6**.

2.1.4. General Procedure 4 (GP4): Decarboxylative thiocyanation

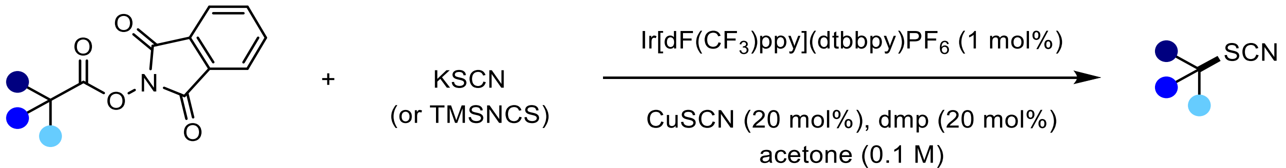

3 **7**

An oven-dried vial (7 mL) was charged with redox active ester **3** (0.20 mmol, 1.0 equiv.), $Ir[dF(CF_3)ppy)_2(dtbbpy)PF_6$ (2.2 mg, 0.0020 mmol, 1.0 mol%), CuSCN (4.9 mg, 0.040 mmol, 20 mol%), 2,9-dimethyl-1,10-phenanthroline (dmp) (8.3 mg, 0.040 mmol, 20 mol%) and KSCN (39 mg, 0.40 mmol, 2.0 equiv.) or trimethylsilyl isothiocyanate (TMSNCS, 56 µL, 0.40 mmol, 2.0 equiv.). The vial was sealed with a septum and placed under an N_2 atmosphere before adding acetone (2.0 mL). The mixture was subsequently degassed by sparging with N_2 for 1 min. The reaction was stirred under blue LED irradiation in the photoreactor for 14 h, after which TLC analysis showed complete conversion. The reaction was concentrated under reduced pressure and purified by flash column chromatography to give alkyl thiocyanate **7**.

Note: KSCN was used for secondary alkyl redox active esters and TMSNCS was used for primary alkyl redox active esters.

2.2. Synthesis of Redox Active Esters

2.2.1. Previously reported substrates

The redox active esters (RAEs) listed in Table S1 were prepared according to our previous report.[1]

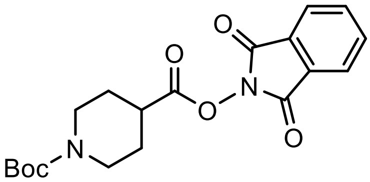

3a

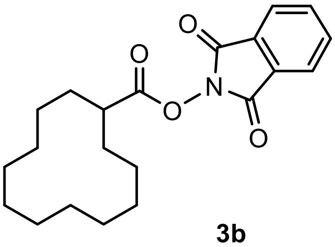

3b

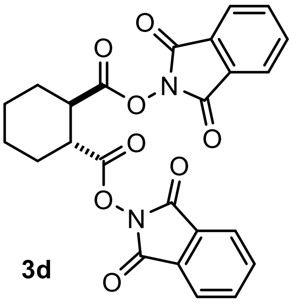

3d

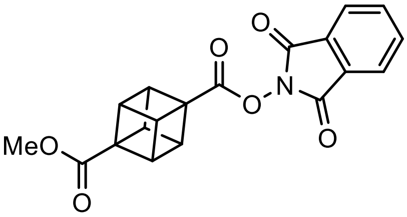

3e

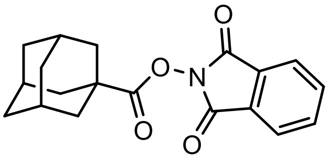

3f

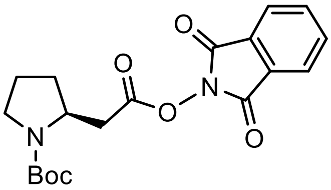

3g

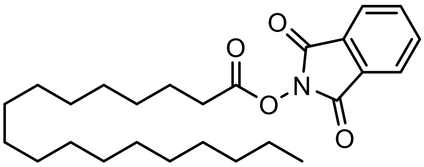

3k

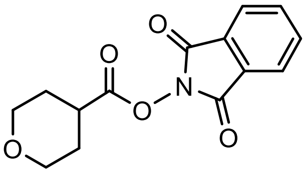

3q

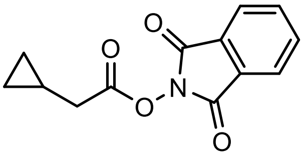

3r

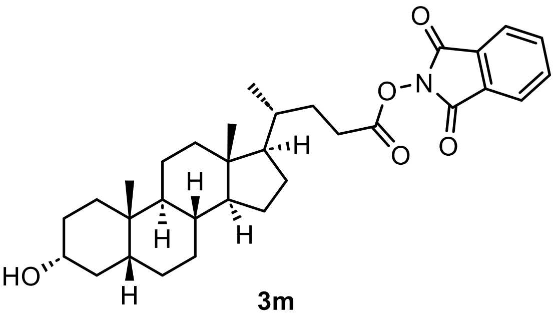

3m

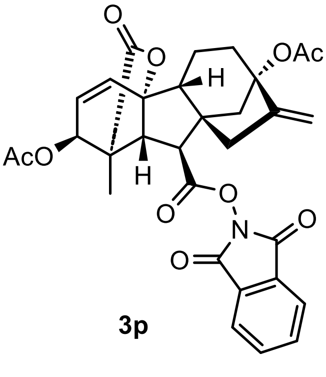

3p

Table S1: Redox active esters prepared according to our previous report

2.2.2. Synthesised substrates

1,3-Dioxoisoindolin-2-yl (1*r*,4*r*)-4-hydroxycyclohexane-1-carboxylate (3c)

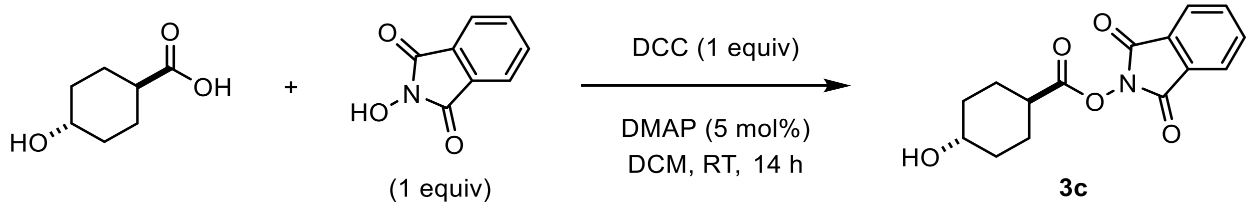

3c

Prepared following **GP1**, using *trans*-4-hydroxycyclohexane-1-carboxylic acid (432 mg, 3.00 mmol), *N*-hydroxyphthalimide (504 mg, 3.09 mmol, 1.02 equiv.), DCC (619 mg, 3.00 mmol, 1.00 equiv.) and DMAP (18 mg, 0.015 mmol, 5 mol%) in DCM (15 mL). Purification by flash column chromatography (Biotage, Sfär Silica 25 g, petroleum ether/Et_2O = 100:0 to 50:50) gave **3c** (272 mg, 31%) as a white solid.

NMR Spectroscopy (see spectra):

^{1}H NMR (500 MHz, $CDCl_3$): δ_H 7.88 (dd, *J* = 5.4, 3.2 Hz, 2H), 7.79 (dd, *J* = 5.5, 3.2 Hz, 2H), 3.69 (tt, *J* = 10.0, 4.3 Hz, 1H), 2.75 – 2.63 (m, 1H), 2.24 (dd, *J* = 14.2, 4.0 Hz, 2H), 2.11 (dd, *J* = 13.1, 4.1 Hz, 2H), 1.77 – 1.66 (m, 2H), 1.58 (br s, 1H), 1.45 – 1.34 (m, 2H) ppm;

^{13}C NMR (125 MHz, $CDCl_3$): δ_C 171.6, 162.1, 134.9, 129.1, 124.1, 69.4, 39.7, 34.1, 26.9 ppm.

IR (film): ν_{max} 3518 (br), 2939, 2861, 1812, 1786, 1741, 1454, 1371, 1186, 1060 cm^{-1}.

HRMS (ESI$^+$): m/z calc'd for $C_{15}H_{15}NO_5Na$ $[M+Na]^+$ 312.0842; found 312.0856.

1,3-Dioxoisoindolin-2-yl 4-(thiophen-2-yl)butanoate (3h)

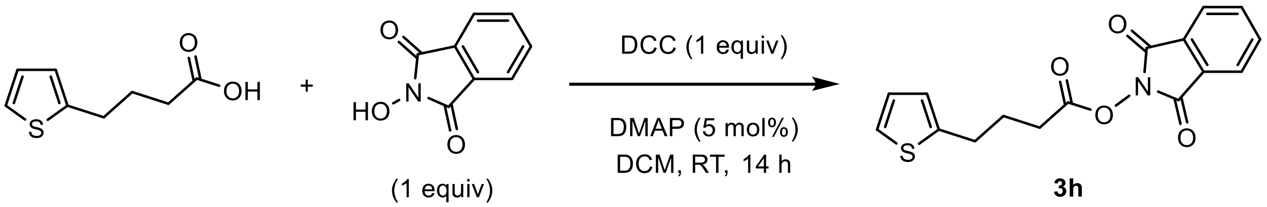

3h

Prepared following **GP1**, using 4-(thiophen-2-yl)butanoic acid (340 mg, 2.00 mmol), *N*-hydroxyphthalimide (336 mg, 2.06 mmol, 1.03 equiv.), DCC (412 mg, 2.00 mmol, 1.00 equiv.) and DMAP (12 mg, 0.010 mmol, 5 mol%) in DCM (10 mL). Purification by flash column chromatography (Biotage, Sfär Silica 25 g, petroleum ether/EtOAc = 100:0 to 60:40) gave **3h** (441 mg, 70%) as a white solid.

NMR Spectroscopy (see spectra):

^{1}H NMR (400 MHz, $CDCl_3$): δ_H 7.91 – 7.86 (m, 2H), 7.81 – 7.76 (m, 2H), 7.15 (d, *J* = 5.1 Hz, 1H), 6.96 – 6.91 (m, 1H), 6.86 (d, *J* = 3.3 Hz, 1H), 3.00 (t, *J* = 7.4 Hz, 2H), 2.72 (t, *J* = 7.3 Hz, 2H), 2.21 – 2.11 (m, 2H) ppm;

^{13}C NMR (100 MHz, $CDCl_3$): δ_C 169.4, 162.1, 143.2, 134.9, 129.0, 127.0, 125.2, 124.1, 123.7, 30.0, 28.7, 26.7 ppm.

All recorded spectroscopic data matched those previously reported in the literature.[2]

1,3-Dioxoisoindolin-2-yl 5-oxo-5-phenylpentanoate (3i)

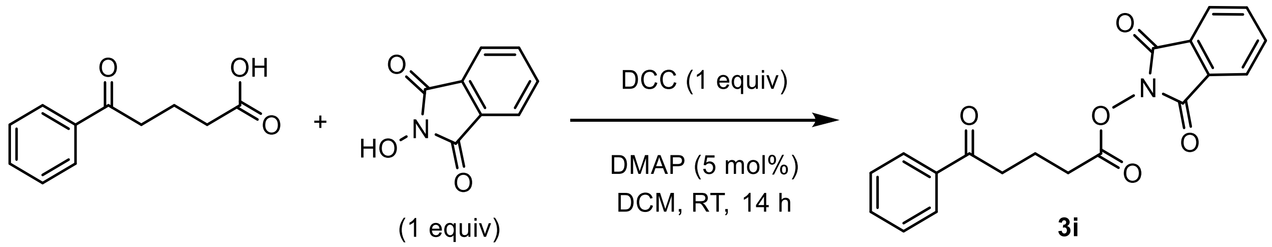

Prepared following **GP1**, using 5-oxo-5-phenylpentanoic acid (380 mg, 2.00 mmol), *N*-hydroxyphthalimide (336 mg, 2.06 mmol, 1.03 equiv.), DCC (412 mg, 2.00 mmol, 1.00 equiv.) and DMAP (12 mg, 0.010 mmol, 5 mol%) in DCM (10 mL). Purification by flash column chromatography (Biotage, Sfär Silica 25 g, petroleum ether/EtOAc = 100:0 to 50:50) gave **3i** (438 mg, 65%) as a white solid.

NMR Spectroscopy (*see spectra*):

^{1}**H NMR** (400 MHz, $CDCl_3$): δ_H 8.01 – 7.97 (m, 2H), 7.90 – 7.85 (m, 2H), 7.80 – 7.75 (m, 2H), 7.58 – 7.53 (m, 1H), 7.49 – 7.43 (m, 2H), 3.19 (t, *J* = 7.1 Hz, 2H), 2.83 (t, *J* = 7.0 Hz, 2H), 2.28 – 2.19 (m, 2H) ppm;

^{13}**C NMR** (100 MHz, $CDCl_3$): δ_C 199.0, 169.5, 162.1, 136.7, 134.9, 133.3, 129.0, 128.8, 128.2, 124.1, 36.8, 30.3, 19.2 ppm.

All recorded spectroscopic data matched those previously reported in the literature.[3]

1,3-Dioxoisoindolin-2-yl 3-(3,4-dimethoxyphenyl)propanoate (3j)

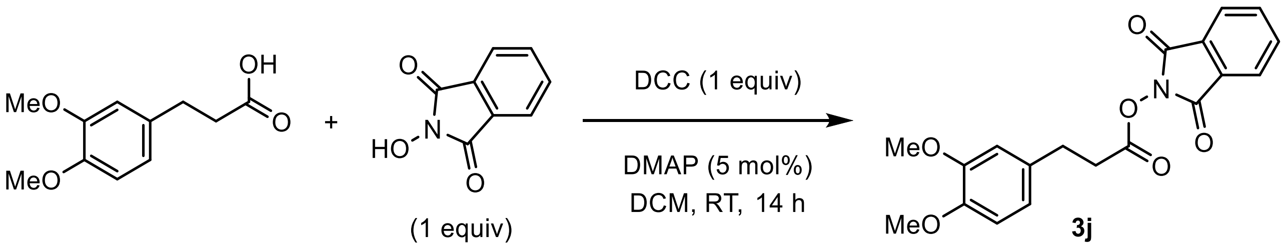

Prepared following **GP1**, using 3-(3,4-dimethoxyphenyl)propanoic acid (420 mg, 2.00 mmol), *N*-hydroxyphthalimide (336 mg, 2.06 mmol, 1.03 equiv.), DCC (412 mg, 2.00 mmol, 1.00 equiv.) and DMAP (12 mg, 0.010 mmol, 5 mol%) in DCM (10 mL). Purification by flash column chromatography (Biotage, Sfär Silica 25 g, petroleum ether/EtOAc = 100:0 to 60:40) gave **3j** (610 mg, 86%) as a white solid.

NMR Spectroscopy (*see spectra*):

^{1}**H NMR** (500 MHz, $CDCl_3$): δ_H 7.90 (dd, *J* = 5.4, 3.1 Hz, 2H), 7.80 (dd, *J* = 5.5, 3.1 Hz, 2H), 6.86 – 6.75 (m, 3H), 3.90 (s, 3H), 3.87 (s, 3H), 3.06 (t, *J* = 7.7 Hz, 2H), 2.97 (t, *J* = 7.7 Hz, 2H) ppm;

^{13}**C NMR** (125 MHz, $CDCl_3$): δ_C 169.1, 162.1, 149.2, 148.0, 134.9, 132.0, 129.1, 124.1, 120.3, 111.7, 111.6,

56.1, 56.0, 33.2, 30.4 ppm.

IR (film): v_{max} 2998, 2934, 2834, 1814, 1786, 1741, 1516, 1466, 1261, 1238, 1080 cm^{-1}.

HRMS (ESI^+): m/z calc'd for $C_{19}H_{17}NO_6Na$ $[M+Na]^+$ 378.0948; found 378.0943.

3-(1,3-Dioxoisoindolin-2-yl) 1-methyl (1*R*,3*S*)-1,2,2-trimethylcyclopentane-1,3-dicarboxylate (3l)

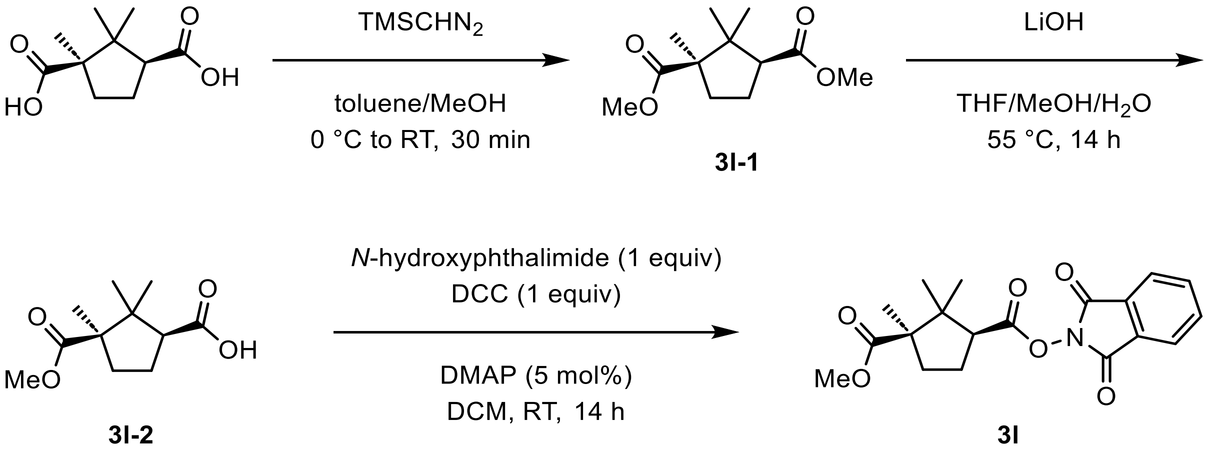

Step 1: A solution of *D*-camphoric acid (400 mg, 2.00 mmol) in toluene (4 mL) and MeOH (1 mL) was cooled to 0 °C before trimethylsilyldiazomethane ($TMSCHN_2$) (2 M in Et_2O, 2.5 mL, 5.0 mmol, 2.5 equiv.) was added. The reaction was removed for the cooling bath and stirred at room temperature for 30 min before concentrating under reduced pressure to give crude **3l-1**, which was used in the next step without further purification.

Step 2: To a solution crude **3l-1** in THF (8 mL), MeOH (2 mL) and H_2O (1 mL), was added lithium hydroxide monohydrate (84 mg, 1 equiv.). The reaction was heated to 55 °C and stirred for 14 h before allowing to cool to room temperature. The mixture was acidified with aqueous HCl (1 M, 4 mL) and extracted with EtOAc (50 + 20 mL). The combined organic extracts were washed with brine, dried ($MgSO_4$), and filtered before being concentrated under reduced pressure to give crude **3l-2**, which was used in the next step without further purification.

Step 3: Prepared following **GP1**, using crude carboxylic acid **3l-2** (2.00 mmol), *N*-hydroxyphthalimide (336 mg, 2.06 mmol, 1.03 equiv.), DCC (412 mg, 2.00 mmol, 1.00 equiv.) and DMAP (12 mg, 0.010 mmol, 5 mol%) in DCM (8 mL). Purification by flash column chromatography (Biotage, Sfär Silica 25 g, hexane/acetone = 100:0 to 80:20) gave **3l** (513 mg, 71% over 3 steps) as a white solid.

NMR Spectroscopy (*see spectra*):

^{1}H NMR (500 MHz, $CDCl_3$): δ_H 7.88 (dd, *J* = 5.5, 3.1 Hz, 2H), 7.79 (dd, *J* = 5.5, 3.1 Hz, 2H), 3.70 (s, 3H), 3.17 (t, *J* = 9.4 Hz, 1H), 2.71 – 2.62 (m, 1H), 2.29 – 2.20 (m, 1H), 2.06 – 1.96 (m, 1H), 1.64 – 1.56 (m, 1H), 1.45 (s, 3H), 1.26 (s, 3H), 0.98 (s, 3H) ppm;

^{13}C NMR (125 MHz, $CDCl_3$): δ_C 175.8, 170.6, 162.2, 134.9, 129.1, 124.1, 56.6, 51.8, 50.6, 47.9, 32.7, 23.1, 22.7, 21.7, 21.0 ppm.

IR (film): ν_{max} 2974, 2951, 2885, 1809, 1784, 1742, 1713, 1467, 1366, 1261, 1186, 1117, 1082 cm^{-1}.

HRMS (ESI$^+$): m/z calc'd for $C_{19}H_{21}NO_6Na$ [M+Na]$^+$ 382.1261; found 382.1258.

1-Benzyl 5-(1,3-dioxoisoindolin-2-yl) (*tert*-butoxycarbonyl)-*D*-glutamate (3n)

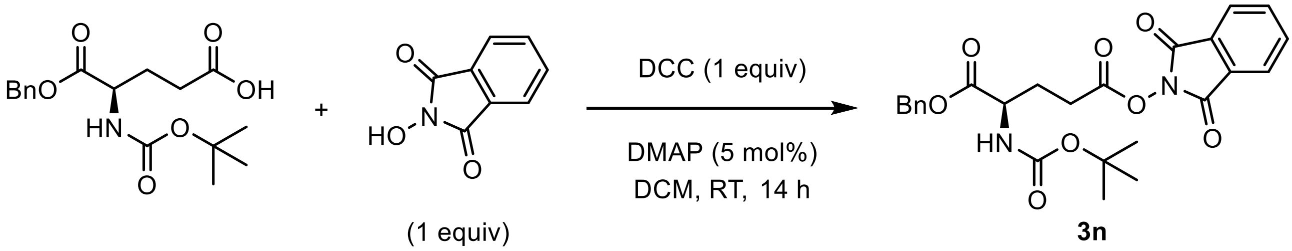

Prepared following **GP1**, using *N*-Boc-*D*-glutamic acid 5-benzyl ester (338 mg, 1.00 mmol), *N*-hydroxyphthalimide (168 mg, 1.03 mmol, 1.03 equiv.), DCC (206 mg, 1.00 mmol, 1.00 equiv.) and DMAP (6 mg, 0.05 mmol, 5 mol%) in DCM (6 mL). Purification by flash column chromatography (Biotage, Sfär Silica 25 g, petroleum ether/EtOAc = 100:0 to 60:40) gave **3n** (466 mg, 97%) as a colourless oil.

NMR Spectroscopy (*see spectra*):

^{1}H NMR (500 MHz, $CDCl_3$): δ_H 7.89 (dd, *J* = 5.5, 3.1 Hz, 2H), 7.79 (dd, *J* = 5.5, 3.1 Hz, 2H), 7.41 – 7.31 (m, 5H), 5.27 – 5.10 (br m, 1H), 5.20 (s, 2H), 4.50 – 4.22 (br m, 1H), 2.79 (ddd, *J* = 17.0, 9.4, 6.3 Hz, 1H), 2.70 (ddd, *J* = 16.9, 9.4, 5.9 Hz, 1H), 2.41 – 2.30 (m, 1H), 2.17 – 2.07 (m, 1H), 1.44 (s, 9H) ppm;

^{13}C NMR (125 MHz, $CDCl_3$): δ_C 171.7, 169.0, 161.9, 155.5, 135.2, 134.9, 129.0, 128.8, 128.7, 128.5, 124.1, 80.4, 67.6, 52.8, 28.4, 27.8, 27.5 ppm.

IR (film): ν_{max} 3383 (br), 3065, 3033, 2978, 2932, 1816, 1789, 1740, 1713, 1499, 1366, 1185, 1158, 1081, 1066 cm^{-1}.

HRMS (ESI$^+$): m/z calc'd for $C_{25}H_{26}N_2O_8Na$ [M+Na]$^+$ 505.1581; found 505.1594.

Gibberellic acid redox active ester (3o)

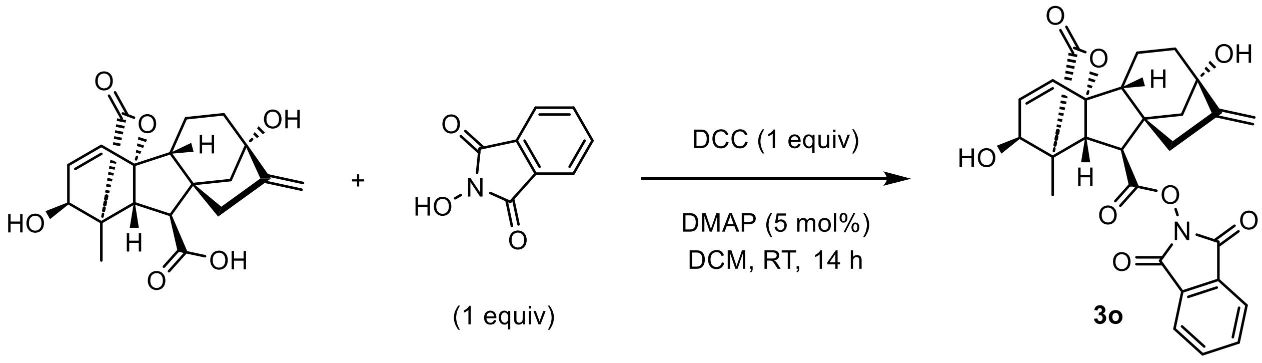

Prepared following **GP1**, using gibberellic acid (692 mg, 2.00 mmol), *N*-hydroxyphthalimide (336 mg, 2.06 mmol, 1.03 equiv.), DCC (412 mg, 2.00 mmol, 1.00 equiv.) and DMAP (12 mg, 0.010 mmol, 5 mol%) in DCM (10 mL).

Purification by flash column chromatography (Biotage, Sfär Silica 25 g, petroleum ether/EtOAc = 100:0 to 30:70) gave **3o** (446 mg, 45%) as a colourless oil.

NMR Spectroscopy (*see spectra*):

> **^{1}H NMR** (500 MHz, $CDCl_3$): δ_H 7.88 (dd, *J* = 5.5, 3.1 Hz, 2H), 7.80 (dd, *J* = 5.5, 3.1 Hz, 2H), 6.33 (d, *J* = 9.3 Hz, 1H), 5.92 (dd, *J* = 9.3, 3.8 Hz, 1H), 5.34 (dd, *J* = 3.2, 1.7 Hz, 1H), 5.06 (s, 1H), 4.21 (d, *J* = 3.7 Hz, 1H), 3.30 (d, *J* = 10.9 Hz, 1H), 3.14 (d, *J* = 10.9 Hz, 1H), 2.79 (dt, *J* = 15.7, 3.0 Hz, 1H), 2.31 – 2.23 (m, 1H), 2.16 – 2.10 (m, 2H), 2.04 – 1.91 (m, 3H), 1.87 – 1.79 (m, 1H), 1.75 – 1.68 (m, 1H), 1.40 (s, 3H) ppm;

> **^{13}C NMR** (125 MHz, $CDCl_3$): δ_C 178.0, 168.9, 161.9, 156.8, 135.1, 132.8, 132.7, 128.9, 124.2, 108.1, 90.3, 78.3, 69.8, 53.7, 53.6, 51.6, 50.9, 48.4, 44.8, 42.9, 38.0, 17.2, 14.6 ppm.

IR (film): ν_{max} 3407 (br), 3061, 3049, 2975, 2937, 2881, 1811, 1777, 1740, 1374, 1186, 1158, 1100, 1070, 1045, 1017 cm^{-1}.

HRMS (ESI^+): m/z calc'd for $C_{27}H_{25}NO_8Na$ $[M+Na]^+$ 514.1472; found 514.1475.

2.3. Optimisation Studies

2.3.1. Decarboxylative Bromination

An oven-dried vial (7 mL) was charged with redox active ester **3a** (0.10 mmol, 1.0 equiv.), photocatalyst (1-5 mol%), and LiBr (0.20 mmol, 2.0 equiv.). The vial was sealed with a septum and placed under a N_2 atmosphere before adding solvent (1.0 mL). The mixture was subsequently degassed by sparging with N_2 for 1 min. The reaction was stirred under blue LED irradiation in the photoreactor for 14 h. The reaction was concentrated under reduced pressure before adding 1,3,5-trimethoxybenzene (5.6 mg, 0.033 mmol) as an internal standard. The yield was subsequently determined by GC analysis.

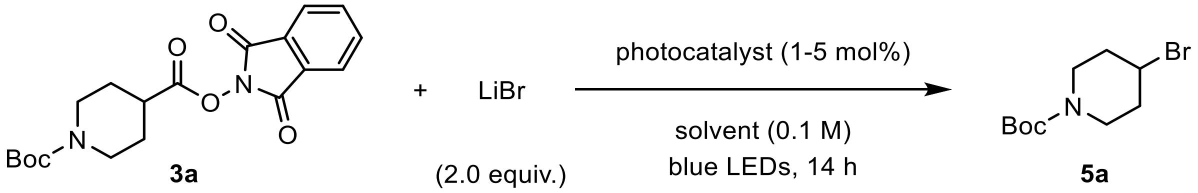

entry	Photocatalyst (mol%)	solvent	conversion	yield[a]
1	none	MeCN	5%	5%
2	$Ir(ppy)_3$ (1)	MeCN	100%	74%
3	$Ir[dF(CF_3)ppy]_2(dtbbpy)PF_6$ (1)	MeCN	100%	93%
4	4CzIPN (5)	MeCN	100%	93%
5	4CzIPN (5)	acetone	100%	86%
6[b]	4CzIPN (5)	MeCN	0%	0%

Table S2: Optimisation of the decarboxylative bromination

[a] Yield of **6a** determined by GC analysis using 1,3,5-trimethoxybenzene as an internal standard. [b] Reaction performed in the dark.

2.3.2. Decarboxylative Chlorination

An oven-dried vial (7 mL) was charged with redox active ester **3a** (0.10 mmol, 1.0 equiv.), photocatalyst (1-5 mol%), copper catalyst (20 mol%), ligand (20 mol%), and LiCl (0.20 mmol, 2.0 equiv.). The vial was sealed with a septum and placed under a N_2 atmosphere before adding anhydrous MeCN (1.0 mL). The mixture was subsequently degassed by sparging with N_2 for 1 min. The reaction was stirred under blue LED irradiation in the photoreactor for 16 h. The reaction was concentrated under reduced pressure before adding 1,3,5-trimethoxybenzene (5.6 mg, 0.033 mmol) as an internal standard. The yield was determined by ^{1}H NMR analysis.

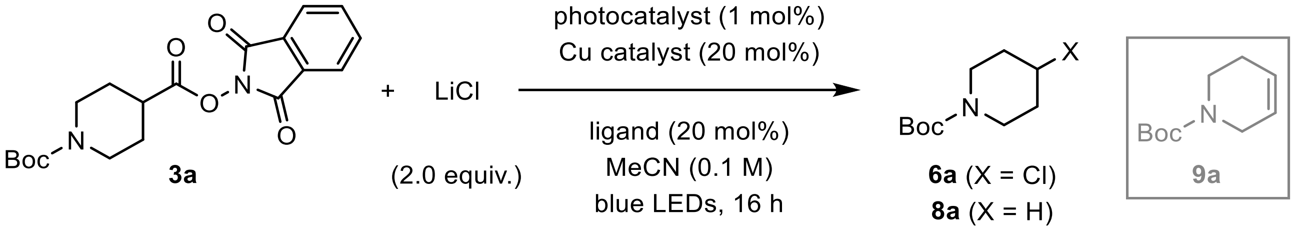

entry	photocatalyst	Cu catalyst	ligand	solvent	conversion	yield[a]
1[b]	Ir(ppy)$_3$	none	none	MeCN	41%	20% (21%)[c]
2[b]	**[Ir-1]**	none	none	MeCN	32%	15% (17%)[c]
3[d]	**[Ir-1]**	none	none	MeCN	50%	<1% (48%)[c]
4	**[Ir-1]**	CuCl	none	MeCN	100%	54% (20%)[e]
5	none	CuCl	none	MeCN	13%	11%
6[f]	none	CuCl	none	MeCN	0%	0%
7	**[Ir-1]**	CuCl	bpy	MeCN	70%	68%
8	**[Ir-1]**	CuCl	dtbbpy	MeCN	100%	90%
9	**[Ir-1]**	CuCl	phen	MeCN	100%	92%
10	**[Ir-1]**	Cu(MeCN)$_4$PF$_6$	dtbbpy	MeCN	100%	91%
11	**[Ir-1]**	CuCl$_2$	dtbbpy	MeCN	100%	96%
12[g]	**[Ir-1]**	CuCl$_2$	dtbbpy	MeCN	46%	42%
13	**[Ir-1]**	CuCl$_2$	dmp	MeCN	100%	92%
14[g]	**[Ir-1]**	CuCl$_2$	dmp	MeCN	100%	87%
15	**[Ir-1]**	CuCl$_2$	dmp	1:1 MeCN/EtOAc	100%	77%
16	**[Ir-1]**	CuCl$_2$	dmp	acetone	100%	62%
17[h]	**[Ir-1]**	CuCl$_2$	dmp	MeCN	100%	86%
18[i]	4CzIPN	CuCl$_2$	dmp	MeCN	100%	86%

Table S3: Optimisation of the decarboxylative chlorination

[a] Yield of **6a** determined by ^{1}H NMR analysis using 1,3,5-trimethoxybenzene as an internal standard. [b] Using 3 equivalents of LiCl. [c] Number in parentheses is the yield of the hydrodecarboxylation product **8a**. [d] Using NH_4Cl (3 equiv) instead of LiCl. [e] Number in parentheses is the yield of alkene **9a**. [f] Reaction run in the dark. [g] Performed on a 0.2 mmol scale. [h] Using 10 mol% $CuCl_2$ and 10 mol% dmp. [i] Using 5 mol% 4CzIPN. **[Ir-1]** = Ir[dF(CF$_3$)ppy]$_2$(dtbbpy)PF$_6$; dtbbpy = 4,4'-di-*tert*-butyl-2,2'-bipyridyl; phen = 1,10-phenanthroline; dmp = 2,9-dimethyl-1,10-phenanthroline.

2.2.3. Decarboxylative Thiocyanation

An oven-dried vial (7 mL) was charged with redox active ester **3a** (0.10 mmol, 1.0 equiv.), photocatalyst (1-5 mol%), CuSCN (20 mol%), ligand (20 mol%), and KSCN (0.20 mmol, 2.0 equiv.). The vial was sealed with a septum and placed under a N_2 atmosphere before adding anhydrous MeCN (1.0 mL). The mixture was subsequently degassed by sparging with N_2 for 1 min. The reaction was stirred under blue LED irradiation in the photoreactor for 16 h. The reaction was concentrated under reduced pressure before adding 1,3,5-trimethoxybenzene (5.6 mg, 0.033 mmol) as an internal standard. The yield was determined by ^{1}H NMR analysis.

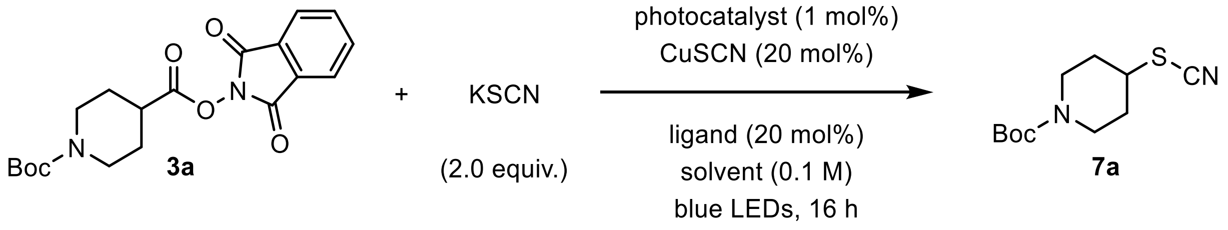

entry	photocatalyst	ligand	solvent	conversion	yield[a]
1[b]	Ir(ppy)$_3$	none	MeCN	100%	0%
2	Ir[dF(CF$_3$)ppy]$_2$(dtbbpy)PF$_6$	dmp	MeCN	100%	74%
3	Ir[dF(CF$_3$)ppy]$_2$(dtbbpy)PF$_6$	dtbbpy	MeCN	50%	31%
4	Ir[dF(CF$_3$)ppy]$_2$(dtbbpy)PF$_6$	dtbbpy	acetone	100%	73%
5	Ir[dF(CF$_3$)ppy]$_2$(dtbbpy)PF$_6$	dmp	acetone	100%	98%
6	Ir[dF(CF$_3$)ppy]$_2$(dtbbpy)PF$_6$	phen	acetone	100%	99%
7[c]	4CzIPN	dmp	acetone	100%	72%

Table S4: Optimisation of the decarboxylative thiocyanation

[a] Yield determined by ^{1}H NMR analysis using 1,3,5-trimethoxybenzene as an internal standard. [b] Reaction performed without CuSCN. [c] Using 5 mol% 4CzIPN. dtbbpy = 4,4'-di-*tert*-butyl-2,2'-bipyridyl; phen = 1,10-phenanthroline; dmp = 2,9-dimethyl-1,10-phenanthroline.

2.4. Decarboxylative Iodination

tert-Butyl 4-iodopiperidine-1-carboxylate (4a)

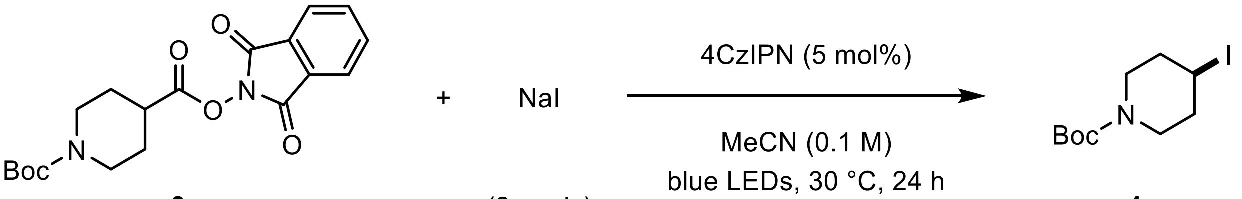

An oven-dried vial (7 mL) was charged with redox active ester **3a** (0.20 mmol, 1.0 equiv.), 4CzIPN (7.9 mg, 0.010 mmol, 5.0 mol%), and NaI (60 mg, 0.40 mmol, 2.0 equiv.). The vial was sealed with a septum and placed under an N_2 atmosphere before adding anhydrous MeCN (2.0 mL). The mixture was subsequently degassed by sparging with N_2 for 1 min. The reaction was stirred under blue LED irradiation in the photoreactor for 24 h, after which TLC analysis showed complete conversion. The reaction was concentrated under reduced pressure and purified by flash column chromatography (Biotage, Sfär Silica 25 g, petroleum ether/Et_2O = 100:0 to 85:15) to give alkyl iodide **4a** (34 mg, 55% yield) as a colourless oil.

Note: The same yield was obtained when using Ir[dF(CF_3)ppy$)_2$(dtbbpy)PF_6 (2.3 mg, 0.0020 mmol, 1.0 mol%) as the photocatalyst.

NMR Spectroscopy (*see spectra*):

^{1}**H NMR** (500 MHz, $CDCl_3$): δ_H 4.43 (p, *J* = 6.0 Hz, 1H), 3.58 (dt, *J* = 13.6, 5.1 Hz, 2H), 3.27 (dt, *J* = 13.7, 5.8 Hz, 2H), 2.01 (q, *J* = 5.7 Hz, 4H), 1.45 (s, 9H) ppm;

^{13}**C NMR** (125 MHz, $CDCl_3$): δ_C 154.8, 79.9, 43.9 (br), 37.5, 28.5, 27.8 ppm.

All recorded spectroscopic data matched those previously reported in the literature.[4]

2.5. Decarboxylative Bromination

tert-Butyl 4-bromopiperidine-1-carboxylate (5a)

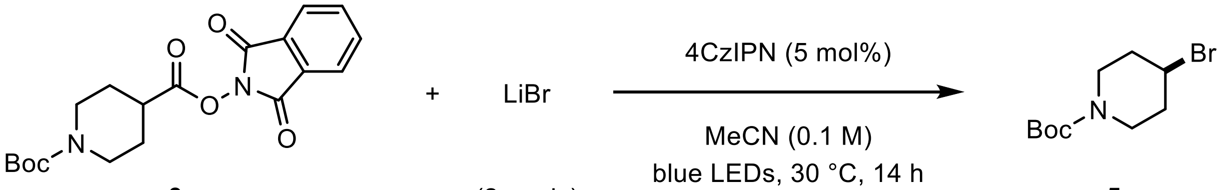

3a (2 equiv) **5a**

Prepared following **GP2**, using redox active ester **3a** (75 mg, 0.20 mmol), 4CzIPN (7.9 mg, 0.010 mmol, 5.0 mol%), and LiBr (35 mg, 0.40 mmol, 2.0 equiv.) in MeCN (2 mL). Purification by flash column chromatography (Biotage, Sfär Silica 10 g, petroleum ether/EtOAc = 100:0 to 85:15) gave **5a** (44 mg, 83%) as a colourless oil.

NMR Spectroscopy (*see spectra*):

^{1}H NMR (400 MHz, $CDCl_3$): δ_H 4.34 (tt, *J* = 7.8, 3.8 Hz, 1H), 3.68 (ddd, *J* = 13.6, 6.8, 3.7 Hz, 2H), 3.36 – 3.26 (m, 2H), 2.08 (ddt, *J* = 14.3, 7.3, 3.7 Hz, 2H), 1.98 – 1.87 (m, 2H), 1.46 (s, 9H) ppm;

All recorded spectroscopic data matched those previously reported in the literature and those of a commercially available sample.[5]

Bromocyclododecane (5b)

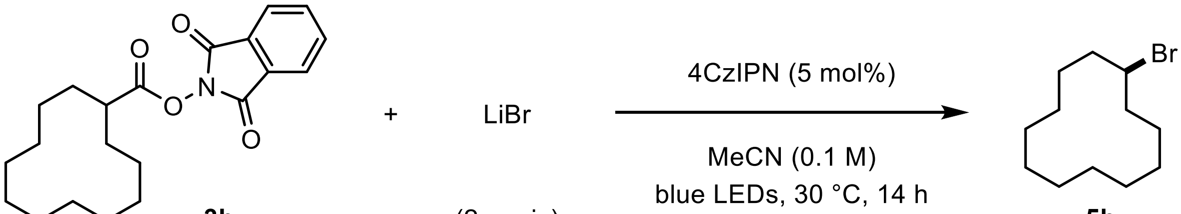

3b (2 equiv) **5b**

Prepared following **GP2**, using redox active ester **3b** (72 mg, 0.20 mmol), 4CzIPN (7.9 mg, 0.010 mmol, 5.0 mol%), and LiBr (35 mg, 0.40 mmol, 2.0 equiv.) in MeCN (2 mL). Purification by flash column chromatography (Biotage, Sfär Silica 10 g, petroleum ether/Et_2O = 100:0 to 90:10) gave **5b** (46 mg, 94%) as a colourless oil.

NMR Spectroscopy (*see spectra*):

^{1}H NMR (500 MHz, $CDCl_3$): δ_H 4.26 (p, *J* = 6.3 Hz, 1H), 2.05 (dq, *J* = 14.0, 6.9 Hz, 2H), 1.89 (dq, *J* = 13.1, 6.1 Hz, 2H), 1.57 – 1.48 (m, 2H), 1.47 – 1.27 (m, 16H) ppm;

^{13}C NMR (125 MHz, $CDCl_3$): δ_C 54.2, 34.8, 23.8, 23.8, 23.6, 22.9 ppm.

All recorded spectroscopic data matched those previously reported in the literature.[6]

(1*r*,4*r*)-4-Bromocyclohexan-1-ol (5c)

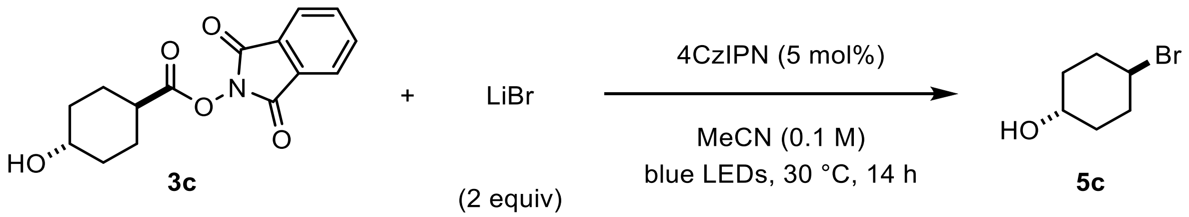

Prepared following **GP2**, using redox active ester **3c** (43 mg, 0.15 mmol), 4CzIPN (5.9 mg, 0.0075 mmol, 5.0 mol%), and LiBr (27 mg, 0.30 mmol, 2.0 equiv.) in MeCN (1.5 mL). Purification by flash column chromatography (Biotage, Sfär Silica 10 g, petroleum ether/Et_2O = 100:0 to 60:40) gave **5c** (19 mg, 71%) as a colourless oil. The d.r. was determined to be 70:30 (*trans*:*cis*) by ^{1}H NMR analysis of the purified product.

NMR Spectroscopy (*see spectra*):

^{1}H NMR (500 MHz, $CDCl_3$, mixture of diastereomers): δ_H 4.36 (tt, *J* = 6.7, 3.5 Hz, 0.70H), 4.13 (tt, *J* = 9.6, 4.0 Hz, 0.30H), 3.84 – 3.72 (m, 1H), 2.28 (dq, *J* = 13.9, 4.5 Hz, 0.70H), 2.20 – 2.10 (m, 1.3H), 2.04 – 1.97 (m, 0.7 H), 1.96 – 1.88 (m, 1.3 H), 1.87 – 1.78 (m, 2H), 1.78 – 1.70 (m, 1.3H), 1.58 (br bs, 1H), 1.42 (dtd, *J* = 13.4, 9.6, 3.7 Hz, 0.7) ppm;

^{13}C NMR (125 MHz, $CDCl_3$, mixture of diastereomers): δ_C 68.2 (minor), 67.9 (major), 52.0 (major), 51.3 (minor), 34.4 (minor), 34.2 (minor), 32.9 (major), 31.7 (major) ppm.

All recorded spectroscopic data matched those previously reported in the literature.[7]

1,3-Dioxoisoindolin-2-yl (1*S,2*R**)-2-bromocyclohexane-1-carboxylate (5d)**

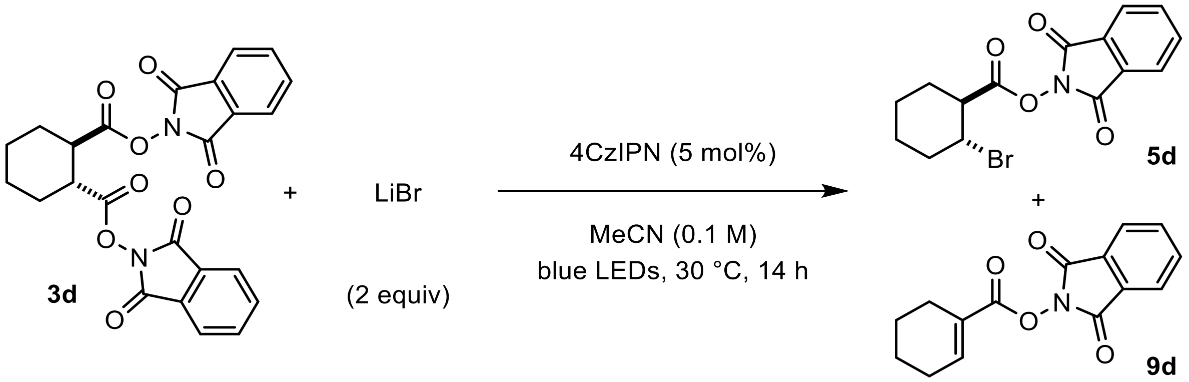

Prepared following **GP2**, using redox active ester **3d** (93 mg, 0.20 mmol), 4CzIPN (7.9 mg, 0.010 mmol, 5.0 mol%), and LiBr (35 mg, 0.40 mmol, 2.0 equiv.) in MeCN (2 mL). Purification by flash column chromatography (Biotage, Sfär Silica 10 g, petroleum ether/Et_2O = 100:0 to 60:40) gave **5d** (18 mg, 26%) as a white solid, and **9d** (6 mg, 11%) as a white solid. The d.r. of **5d** was determined to be >95:5 (*trans*:*cis*) by ^{1}H NMR analysis of the purified product.

For **5d**:

NMR Spectroscopy (*see spectra*):

^{1}H NMR (500 MHz, $CDCl_3$): δ_H 7.88 (dt, *J* = 7.7, 3.8 Hz, 2H), 7.79 (dd, *J* = 5.5, 3.1 Hz, 2H), 4.28 (td, *J* = 10.6, 4.3 Hz, 1H), 3.13 (td, *J* = 10.7, 3.9 Hz, 1H), 2.47 – 2.40 (m, 1H), 2.38 – 2.30 (m, 1H), 1.95 – 1.79 (m, 3H), 1.74 (qd, *J* = 11.7, 3.5 Hz, 1H), 1.50 – 1.39 (m, 2H) ppm;

^{13}C NMR (125 MHz, $CDCl_3$): δ_C 169.8, 161.9, 134.9, 129.1, 124.1, 50.7, 48.8, 36.5, 30.8, 26.1, 24.1 ppm.

IR (film): ν_{max} 2940, 2861, 1815, 1786, 1742, 1467, 1448, 1371, 1185, 1082, 990, 877 cm^{-1}.

HRMS (ESI^+): m/z calc'd for $C_{15}H_{14}BrNO_4Na$ $[M+Na]^+$ 373.9998; found 374.0013.

For **9d**:

NMR Spectroscopy (*see spectra*):

^{1}H NMR (500 MHz, $CDCl_3$): δ_H 7.92 – 7.87 (m, 2H), 7.81 – 7.77 (m, 2H), 7.39 (tt, *J* = 3.9, 1.7 Hz, 1H), 2.42 – 2.37 (m, 2H), 2.34 – 2.28 (m, 2H), 1.76 – 1.70 (m, 2H), 1.70 – 1.64 (m, 2H) ppm;

^{13}C NMR (125 MHz, $CDCl_3$): δ_C 163.1, 162.5, 146.0, 134.8, 129.2, 126.3, 124.1, 26.4, 24.2, 21.8, 21.2 ppm.

All recorded spectroscopic data matched those previously reported in the literature.[8]

Methyl (1*r*,2*R*,3*r*,4*s*,8*S*)-4-bromocubane-1-carboxylate (5e)

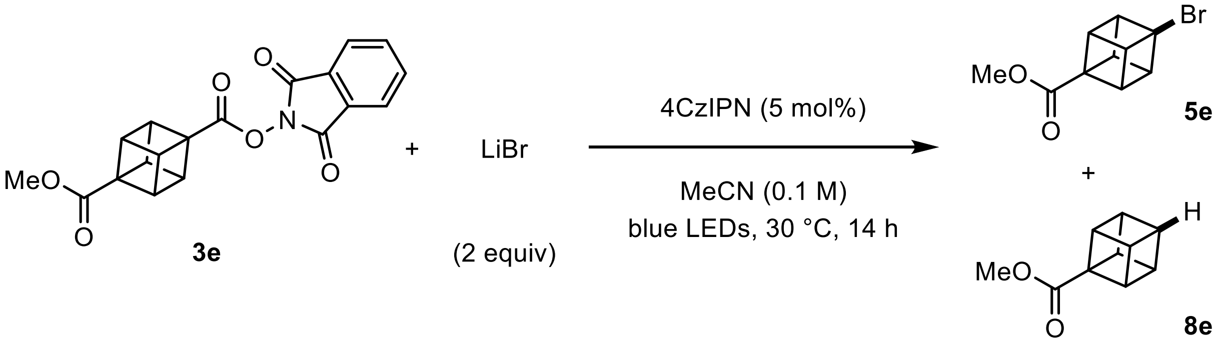

Prepared following **GP2**, using redox active ester **3e** (70 mg, 0.20 mmol), 4CzIPN (7.9 mg, 0.010 mmol, 5.0 mol%), and LiBr (35 mg, 0.40 mmol, 2.0 equiv.) in MeCN (2 mL). Purification by flash column chromatography (Biotage, Sfär Silica 10 g, petroleum ether/Et_2O = 100:0 to 85:15) gave **5e** (28 mg, 58%) as a white solid, and **8e** (5 mg, 15%) as a white solid.

For **5e**:

NMR Spectroscopy (*see spectra*):

^{1}H NMR (500 MHz, $CDCl_3$): δ_H 4.35 – 4.29 (m, 3H), 4.28 – 4.23 (m, 3H), 3.71 (s, 3H) ppm;

^{13}C NMR (125 MHz, $CDCl_3$): δ_C 172.0, 63.3, 56.4, 54.7, 51.8, 47.9 ppm.

All recorded spectroscopic data matched those previously reported in the literature.[9]

For **8e**:

NMR Spectroscopy (*see spectra*):

^{1}H NMR (500 MHz, $CDCl_3$): δ_H 4.25 (ddd, *J* = 6.4, 3.9, 2.2 Hz, 3H), 4.05 – 3.96 (m, 4H), 3.70 (s, 3H) ppm;

^{13}C NMR (125 MHz, $CDCl_3$): δ_C 173.0, 55.8, 51.6, 49.6, 48.0, 45.3 ppm.

All recorded spectroscopic data matched those previously reported in the literature.[10]

(3*s*,5*s*,7*s*)-1-Bromoadamantane (5f)

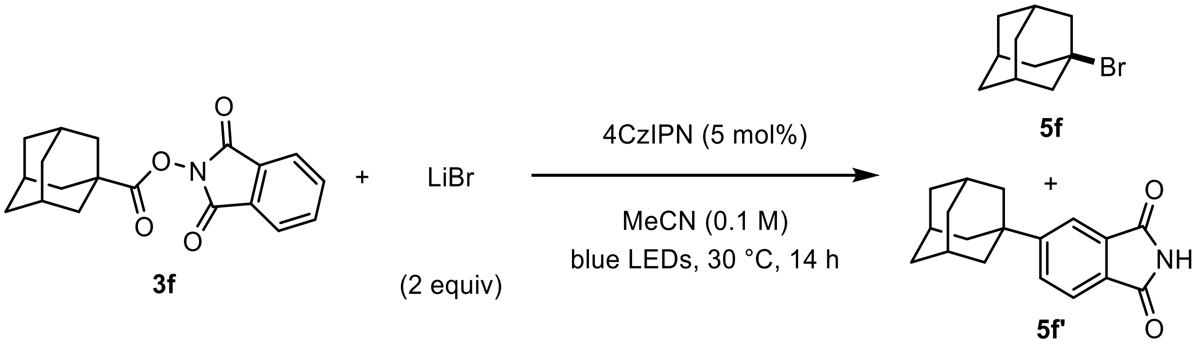

Prepared following **GP2**, using redox active ester **3f** (65 mg, 0.20 mmol), 4CzIPN (7.9 mg, 0.010 mmol, 5.0 mol%), and LiBr (35 mg, 0.40 mmol, 2.0 equiv.) in MeCN (2 mL). Purification by flash column chromatography (Biotage, Sfär Silica 10 g, petroleum ether/Et_2O = 100:0 to 70:30) gave **5f** (29 mg, 64%) as a white solid, and **7f'** (4 mg, 7%) as a white solid.

For **5f**:

NMR Spectroscopy (*see spectra*):

^{1}H NMR (500 MHz, $CDCl_3$): δ_H 2.36 (d, *J* = 2.9 Hz, 6H), 2.10 (t, *J* = 3.3 Hz, 3H), 1.73 (d, *J* = 3.0 Hz, 6H) ppm;

^{13}C NMR (125 MHz, $CDCl_3$): δ_C 66.9, 49.5, 35.7, 32.7 ppm.

All recorded spectroscopic data matched those previously reported in the literature.[11]

For **5f'**:

NMR Spectroscopy (*see spectra*):

^{1}H NMR (500 MHz, $CDCl_3$): δ_H 7.88 (d, *J* = 1.5 Hz, 1H), 7.80 (br s, 1H), 7.79 (d, *J* = 7.9 Hz, 1H), 7.74 (dd, *J* = 7.9, 1.6 Hz, 1H), 2.17 – 2.10 (m, 3H), 1.95 (d, *J* = 2.8 Hz, 6H), 1.85 – 1.73 (m, 6H) ppm;

^{13}C NMR (125 MHz, $CDCl_3$): δ_C 168.7, 168.3, 159.3, 133.0, 131.2, 130.1, 123.6, 120.7, 43.1, 37.5, 36.6, 28.8 ppm.

All recorded spectroscopic data matched those previously reported in the literature.[12]

tert-Butyl (S)-2-(bromomethyl)pyrrolidine-1-carboxylate (5g)

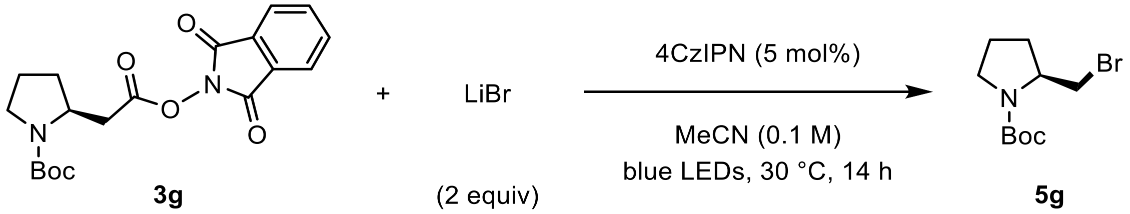

3g (2 equiv) **5g**

Prepared following **GP2**, using redox active ester **3g** (75 mg, 0.20 mmol), 4CzIPN (7.9 mg, 0.010 mmol, 5.0 mol%), and LiBr (35 mg, 0.40 mmol, 2.0 equiv.) in MeCN (2 mL). Purification by flash column chromatography (Biotage, Sfär Silica 10 g, petroleum ether/EtOAc = 100:0 to 80:20) gave **5g** (25 mg, 47%) as a yellow oil.

NMR Spectroscopy (*see spectra*):

^{1}H NMR (400 MHz, $CDCl_3$): δ_H 4.10 – 3.91 (m, 1H), 3.70 – 3.50 (m, 1H), 3.47 – 3.21 (m, 3H), 2.08 – 1.76 (m, 4H), 1.46 (s, 9H) ppm;

^{13}C NMR (100 MHz, $CDCl_3$): δ_C 154.6 + 154.1 (rotameric peaks), 80.1 + 79.7 (rotameric peaks), 58.0 + 57.9 (rotameric peaks), 47.5 + 47.0 (rotameric peaks), 35.0, 30.2 + 29.5 (rotameric peaks), 28.6, 23.7 + 22.9 (rotameric peaks) ppm.

All recorded spectroscopic data matched those previously reported in the literature.[13]

2-(3-Bromopropyl)thiophene (5h)

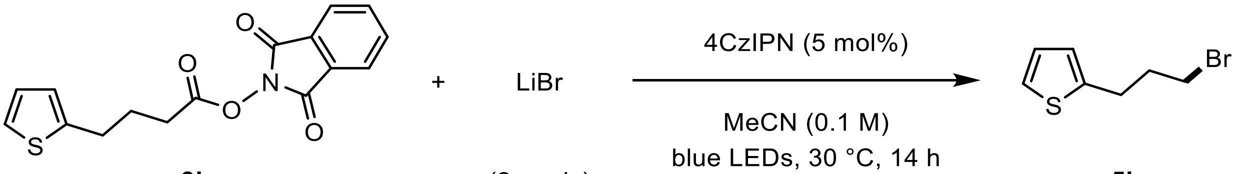

3h (2 equiv) **5h**

Prepared following **GP2**, using redox active ester **3h** (61 mg, 0.20 mmol), 4CzIPN (7.9 mg, 0.010 mmol, 5.0 mol%), and LiBr (35 mg, 0.40 mmol, 2.0 equiv.) in MeCN (2 mL). Purification by flash column chromatography (Biotage, Sfär Silica 10 g, petroleum ether/Et_2O = 100:0 to 90:10) gave **5h** (21 mg, 51%) as a yellow oil.

NMR Spectroscopy (*see spectra*):

^{1}H NMR (400 MHz, $CDCl_3$): δ_H 7.14 (dd, J = 5.1, 1.2 Hz, 1H), 6.93 (dd, J = 5.1, 3.4 Hz, 1H), 6.85 – 6.83 (m, 1H), 3.44 (t, J = 6.5 Hz, 2H), 3.02 (t, J = 7.2 Hz, 2H), 2.26 – 2.16 (m, 2H) ppm;

^{13}C NMR (100 MHz, $CDCl_3$): δ_C 143.1, 127.0, 125.1, 123.6, 34.4, 32.8, 28.2 ppm.

All recorded spectroscopic data matched those previously reported in the literature.[14]

4-Bromo-1-phenylbutan-1-one (5i)

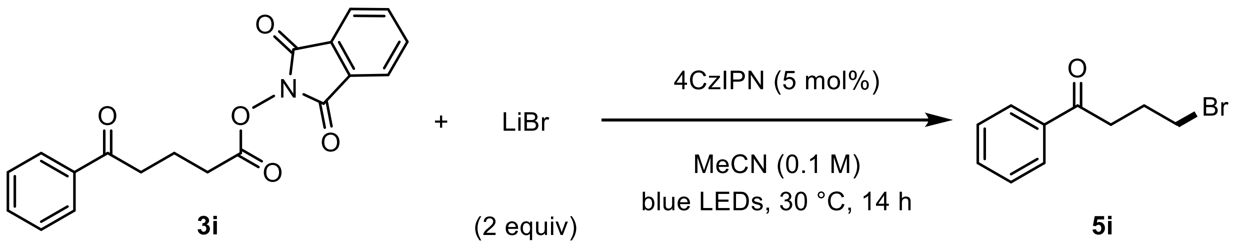

Prepared following **GP2**, using redox active ester **3i** (68 mg, 0.20 mmol), 4CzIPN (7.9 mg, 0.010 mmol, 5.0 mol%), and LiBr (35 mg, 0.40 mmol, 2.0 equiv.) in MeCN (2 mL). Purification by flash column chromatography (Biotage, Sfär Silica 10 g, petroleum ether/EtOAc = 100:0 to 75:25) gave **5i** (20 mg, 44%) as a yellow oil.

NMR Spectroscopy (*see spectra*):

^{1}H NMR (400 MHz, $CDCl_3$): δ_H 8.01 – 7.96 (m, 2H), 7.61 – 7.55 (m, 1H), 7.50 – 7.45 (m, 2H), 3.56 (t, *J* = 6.3 Hz, 2H), 3.19 (t, *J* = 6.9 Hz, 2H), 2.36 – 2.28 (m, 2H) ppm;

^{13}C NMR (100 MHz, $CDCl_3$): δ_C 199.0, 136.9, 133.4, 128.8, 128.2, 36.7, 33.8, 27.0 ppm.

All recorded spectroscopic data matched those previously reported in the literature.[15]

4-(2-Bromoethyl)-1,2-dimethoxybenzene (5j)

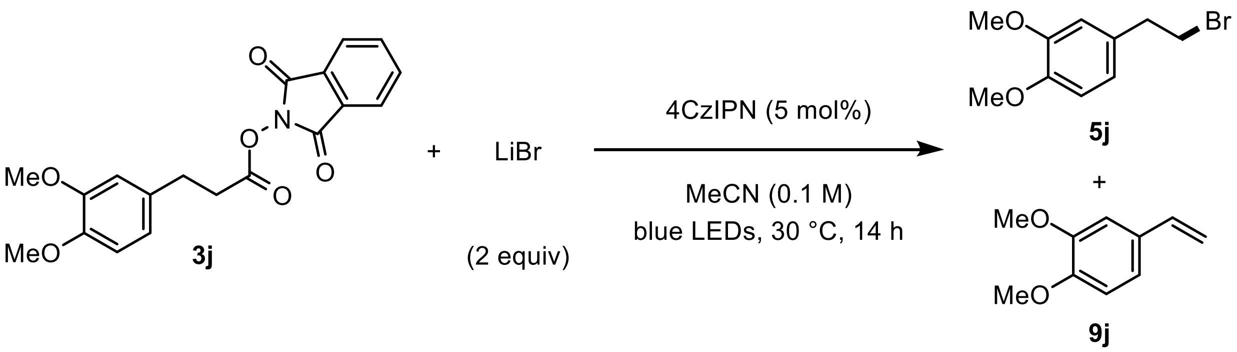

Prepared following **GP2**, using redox active ester **3j** (71 mg, 0.20 mmol), 4CzIPN (7.9 mg, 0.010 mmol, 5.0 mol%), and LiBr (35 mg, 0.40 mmol, 2.0 equiv.) in MeCN (2 mL). Purification by flash column chromatography (Biotage, Sfär Silica 10 g, petroleum ether/Et_2O = 100:0 to 85:15) gave **5j** (20 mg, 41%) as a colourless oil, and **9j** (6 mg, 18%).

For **5j**:

NMR Spectroscopy (*see spectra*):

^{1}H NMR (500 MHz, $CDCl_3$): δ_H 6.82 (d, *J* = 8.1 Hz, 1H), 6.78 – 6.71 (m, 2H), 3.88 (s, 3H), 3.87 (s, 3H), 3.55 (t, *J* = 7.7 Hz, 2H), 3.11 (t, *J* = 7.7 Hz, 2H) ppm;

^{13}C NMR (125 MHz, $CDCl_3$): δ_C 149.1, 148.1, 131.7, 120.8, 112.0, 111.4, 56.04, 56.01, 39.2, 33.4 ppm.

All recorded spectroscopic data matched those previously reported in the literature.[16]

For **9j**:

NMR Spectroscopy (*see spectra*):

^{1}H NMR (500 MHz, $CDCl_3$): δ_H 6.97 (d, *J* = 2.0 Hz, 1H), 6.95 (dd, *J* = 8.2, 2.0 Hz, 1H), 6.83 (d, *J* = 8.2 Hz, 1H), 6.66 (dd, *J* = 17.6, 10.8 Hz, 1H), 5.62 (dd, *J* = 17.5, 0.8 Hz, 1H), 5.15 (dd, *J* = 10.9, 0.8 Hz, 1H), 3.91 (s, 3H), 3.89 (s, 3H) ppm;

^{13}C NMR (125 MHz, $CDCl_3$): δ_C 149.2, 149.1, 136.6, 130.9, 119.6, 112.0, 111.2, 108.7, 56.1, 56.0 ppm.

All recorded spectroscopic data matched those previously reported in the literature.[17]

1-Bromoheptadecane (5k)

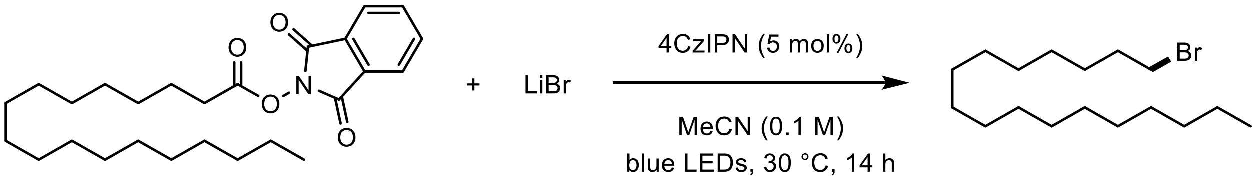

3k (2 equiv) **5k**

Prepared following **GP2**, using redox active ester **3k** (86 mg, 0.20 mmol), 4CzIPN (7.9 mg, 0.010 mmol, 5.0 mol%), and LiBr (35 mg, 0.40 mmol, 2.0 equiv.) in MeCN (2 mL). Purification by flash column chromatography (Biotage, Sfär Silica 25 g, pentane/Et_2O = 100:0 to 95:5) gave **5k** (41 mg, 64%) as a colourless oil.

NMR Spectroscopy (*see spectra*):

^{1}H NMR (500 MHz, $CDCl_3$): δ_H 3.41 (t, *J* = 6.9 Hz, 2H), 1.85 (p, *J* = 7.0 Hz, 2H), 1.46 – 1.38 (m, 2H), 1.33 – 1.21 (m, 26H), 0.88 (t, *J* = 6.8 Hz, 3H) ppm;

^{13}C NMR (125 MHz, $CDCl_3$): δ_C 34.2, 33.0, 32.1, 29.85 (5C), 29.81, 29.78, 29.7, 29.6, 29.5, 28.9, 28.4, 22.9, 14.3 ppm.

All recorded spectroscopic data matched those previously reported in the literature.[18]

Methyl (1*R*)-3-bromo-1,2,2-trimethylcyclopentane-1-carboxylate (5l)

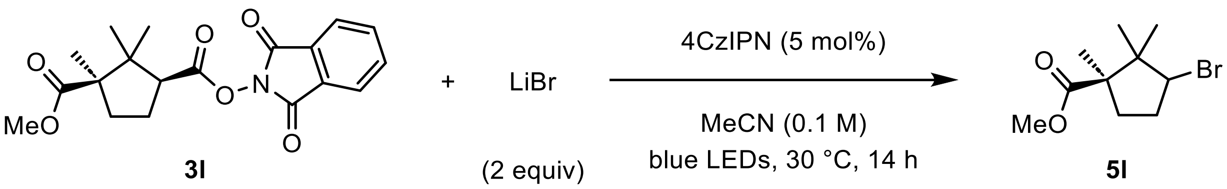

3l (2 equiv) **5l**

Prepared following **GP2**, using redox active ester **3l** (72 mg, 0.20 mmol), 4CzIPN (7.9 mg, 0.010 mmol, 5.0 mol%), and LiBr (35 mg, 0.40 mmol, 2.0 equiv.) in MeCN (2 mL). Purification by flash column chromatography (Biotage, Sfär Silica 10 g, petroleum ether/Et_2O = 100:0 to 90:10) gave **5l** (38 mg, 76%) as a colourless oil. The d.r. was determined to be 74:26 by ^{1}H NMR analysis of the purified product.

NMR Spectroscopy (*see spectra*):

^{1}H NMR (500 MHz, $CDCl_3$, major diastereomer): δ_H 4.38 (dd, *J* = 9.8, 8.4 Hz, 1H), 3.65 (s, 3H), 2.43 (dddd, *J* = 13.3, 9.8, 8.5, 4.7 Hz, 1H), 2.21 (ddd, *J* = 13.5, 9.8, 5.7 Hz, 1H), 2.09 – 2.00 (m, 1H), 1.61 (ddd, *J* = 13.6, 12.0, 4.7 Hz, 1H), 1.25 (s, 3H), 1.01 (s, 3H), 0.89 (s, 3H) ppm;

^{1}H NMR (500 MHz, $CDCl_3$, minor diastereomer): δ_H 4.17 (dd, *J* = 10.6, 8.6 Hz, 1H), 3.69 (s, 3H), 2.69 (td, *J* = 13.0, 4.7 Hz, 1H), 2.30 (dddd, *J* = 13.6, 9.7, 8.7, 4.7 Hz, 1H), 2.10 – 1.99 (m, 1H), 1.46 (ddd, *J* = 13.9, 9.8, 6.0 Hz, 1H), 1.19 (s, 3H), 1.13 (s, 3H), 0.90 (s, 3H) ppm;

^{13}C NMR (125 MHz, $CDCl_3$, major diastereomer): δ_C 177.7, 61.4, 53.3, 51.8, 47.8, 33.9, 32.8, 22.3, 20.9, 20.8 ppm.

^{13}C NMR (125 MHz, $CDCl_3$, minor diastereomer): δ_C 176.1, 61.3, 52.3, 51.9, 46.9, 32.1, 31.4, 22.9, 21.2, 21.0 ppm.

IR (film): ν_{max} 2974, 2947, 2877, 1725, 1460, 1433, 1289, 1197, 1114, 1095, 839 cm^{-1}.

HRMS (APCI$^+$): m/z calc'd for $C_{10}H_{17}O_2Br$ $[M+H]^+$ 249.0485; found 249.0479.

((3*R*,5*R*,8*R*,9*S*,10*S*,13*R*,14*S*,17*R*)-17-((*R*)-4-Bromobutan-2-yl)-10,13-dimethylhexadecahydro-1*H*-cyclopenta[*a*]phenanthren-3-ol (5m)

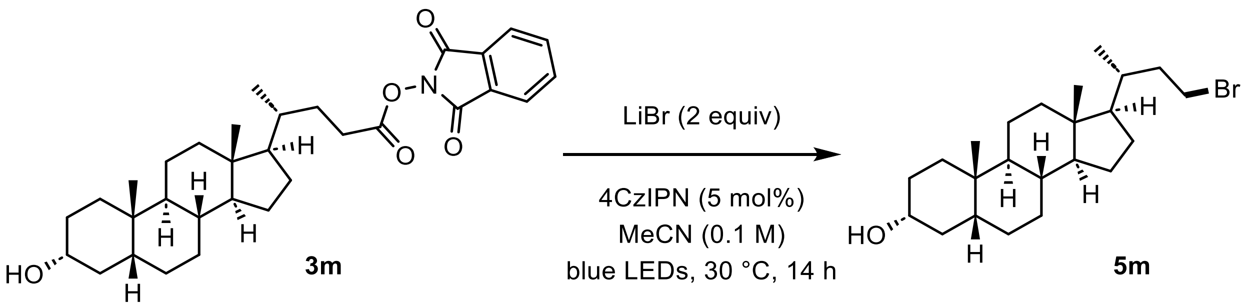

Prepared following **GP2**, using redox active ester **3m** (52 mg, 0.10 mmol), 4CzIPN (4 mg, 0.005 mmol, 5 mol%), and LiBr (18 mg, 0.20 mmol, 2.0 equiv.) in MeCN (1 mL). Purification by flash column chromatography (Biotage, Sfär Silica 10 g, hexane/acetone = 100:0 to 85:15) gave **5m** (34 mg, 83%) as a white solid.

NMR Spectroscopy (*see spectra*):

^{1}H NMR (500 MHz, $CDCl_3$): δ_H 3.63 (tt, *J* = 10.7, 4.6 Hz, 1H), 3.50 (td, *J* = 9.3, 3.7 Hz, 1H), 3.36 (q, *J* = 8.3 Hz, 1H), 2.05 – 1.93 (m, 2H), 1.89 – 1.70 (m, 4H), 1.66 (d, *J* = 11.4 Hz, 1H), 1.61 – 1.54 (m, 3H), 1.53 – 1.47 (m, 2H), 1.44 – 1.35 (m, 5H), 1.34 – 1.30 (m, 1H), 1.29 – 1.21 (m, 2H), 1.17 – 1.03 (m, 5H), 0.98 (dd, *J* = 14.3, 3.4 Hz, 1H), 0.93 (d, *J* = 6.2 Hz, 3H), 0.92 (s, 3H), 0.66 (s, 3H) ppm;

^{13}C NMR (125 MHz, $CDCl_3$): δ_C 72.0, 56.7, 56.2, 43.0, 42.2, 40.6, 40.3, 39.5, 36.6, 36.0, 35.5, 35.2, 34.7, 32.3, 30.7, 28.4, 27.3, 26.6, 24.3, 23.5, 21.0, 18.2, 12.2 ppm.

IR (film): ν_{max} 3286 (br), 2927, 2864, 1466, 1376, 1356, 1247, 1066, 1040, 1014, 740 cm^{-1}.

HRMS (APCI$^+$): m/z calc'd for $C_{23}H_{39}BrO$ $[M+H^+-H_2O]^+$ 393.2151; found 393.2141.

Benzyl (*R*)-4-bromo-2-((*tert*-butoxycarbonyl)amino)butanoate (5n)

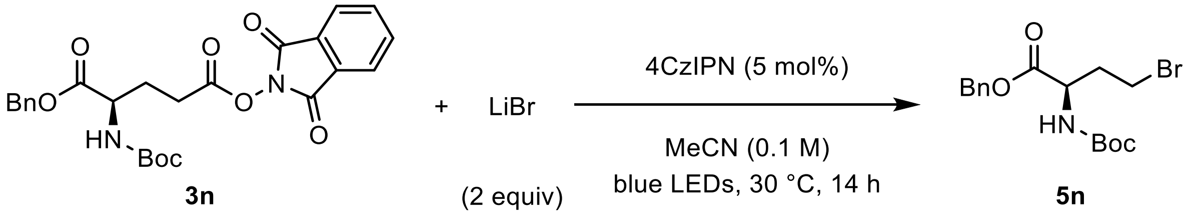

Prepared following **GP2**, using redox active ester **3n** (72 mg, 0.15 mmol), 4CzIPN (6 mg, 0.0075 mmol, 5 mol%), and LiBr (27 mg, 0.30 mmol, 2.0 equiv.) in MeCN (1.5 mL). Purification by flash column chromatography (Biotage, Sfär Silica 10 g, hexane/EtOAc = 100:0 to 50:50) gave **5n** (28 mg, 50%) as a colourless oil.

NMR Spectroscopy (*see spectra*):

^{1}H NMR (500 MHz, $CDCl_3$): δ_H 7.41 – 7.32 (m, 5H), 5.20 (d, J = 12.2 Hz, 1H), 5.17 (d, J = 12.2 Hz, 1H), 5.13 (br d, J = 7.7 Hz, 1H), 4.51 – 4.31 (br m, 1H), 3.35 – 3.44 (m, 2H), 2.48 – 2.36 (m, 1H), 2.26 – 2.17 (m, 1H), 1.44 (s, 9H) ppm;

^{13}C NMR (125 MHz, $CDCl_3$): δ_C 171.7, 155.4, 135.2, 128.8, 128.7, 128.5, 80.4, 67.6, 52.7, 35.9, 28.4, 28.3 ppm.

IR (film): ν_{max} 3361 (br), 3065, 3033, 2976, 2932, 1710, 1499, 1366, 1246, 1213, 1157, 1048, 1023, 749 cm^{-1}.

HRMS (ESI$^+$): m/z calc'd for $C_{16}H_{22}BrNO_4Na$ $[M+Na]^+$ 394.0624; found 394.0620.

(1*S*,2*S*,4a*R*,4b*R*,7*S*,9a*S*,10*S*,10a*R*)-10-Bromo-2,7-dihydroxy-1-methyl-8-methylene-1,2,4b,5,6,7,8,9,10,10a-decahydro-4a,1-(epoxymethano)-7,9a-methanobenzo[*a*]azulen-13-one (5o)

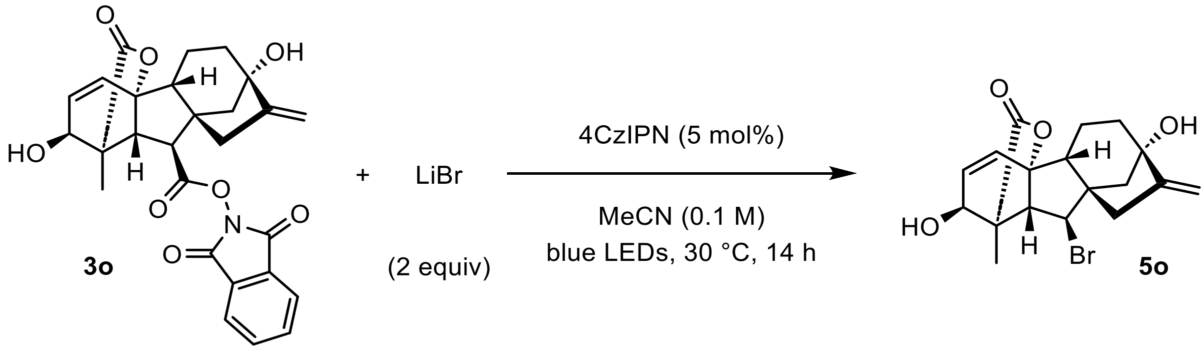

Prepared following **GP2**, using redox active ester **3o** (49 mg, 0.10 mmol), 4CzIPN (4 mg, 0.005 mmol, 5 mol%), and LiBr (18 mg, 0.20 mmol, 2.0 equiv.) in MeCN (1 mL). Purification by flash column chromatography (Biotage, Sfär Silica 10 g, hexane/EtOAc = 100:0 to 40:60) gave **5o** (15.3 mg, 40%) as a white solid. The d.r. was determined to be >95:5 by ^{1}H NMR analysis of the purified product. A small amount (<3 mg) of the minor diastereomer was also isolated, however, it was obtained as a mixture with an unidentified side product, which prevented accurate determination of the yield.

NMR Spectroscopy (*see spectra*):

^{1}H NMR (500 MHz, CD_3CN): δ_H 6.26 (dd, *J* = 9.3, 0.9 Hz, 1H), 5.79 (dd, *J* = 9.3, 3.7 Hz, 1H), 5.14 (ddd, *J* = 2.9, 1.8, 0.7 Hz, 1H), 4.91 (td, *J* = 1.7, 0.7 Hz, 1H), 4.13 (d, *J* = 10.1 Hz, 1H), 3.98 (dd, *J* = 6.6, 3.7 Hz, 1H), 3.56 (d, *J* = 6.7 Hz, 1H), 3.04 (d, *J* = 10.2 Hz, 1H), 2.92 (s, 1H), 2.71 (dt, *J* = 15.7, 3.0 Hz, 1H), 2.26 – 2.20 (m, 1H), 2.03 (dd, *J* = 11.3, 5.4 Hz, 1H), 1.93 – 1.90 (m, 1H), 1.76 – 1.62 (m, 4H), 1.51 (d, *J* = 11.3 Hz, 1H), 1.34 (s, 3H) ppm;

^{13}C NMR (125 MHz, CD_3CN): δ_C 179.2, 158.8, 133.7, 133.0, 107.1, 90.3, 78.7, 70.6, 59.8, 55.5, 54.3, 52.1, 50.4, 46.8, 43.6, 39.6, 18.0, 15.2 ppm.

IR (film): v_{max} 3401 (br), 3077, 3041, 2971, 2936, 2877, 1763, 1707, 1453, 1380, 1249, 1159, 1100, 1045, 1010, 967, 889 cm^{-1}.

HRMS (ESI$^+$): m/z calc'd for $C_{18}H_{21}BrO_4Na$ $[M+Na]^+$ 403.0515; found 403.0519.

(1*S*,2*S*,4a*R*,4b*R*,7*S*,9a*S*,10*S*,10a*R*)-10-Bromo-1-methyl-8-methylene-13-oxo-1,2,5,6,8,9,10,10a-octahydro-4a,1-(epoxymethano)-7,9a-methanobenzo[a]azulene-2,7(4b*H*)-diyl diacetate (5p)

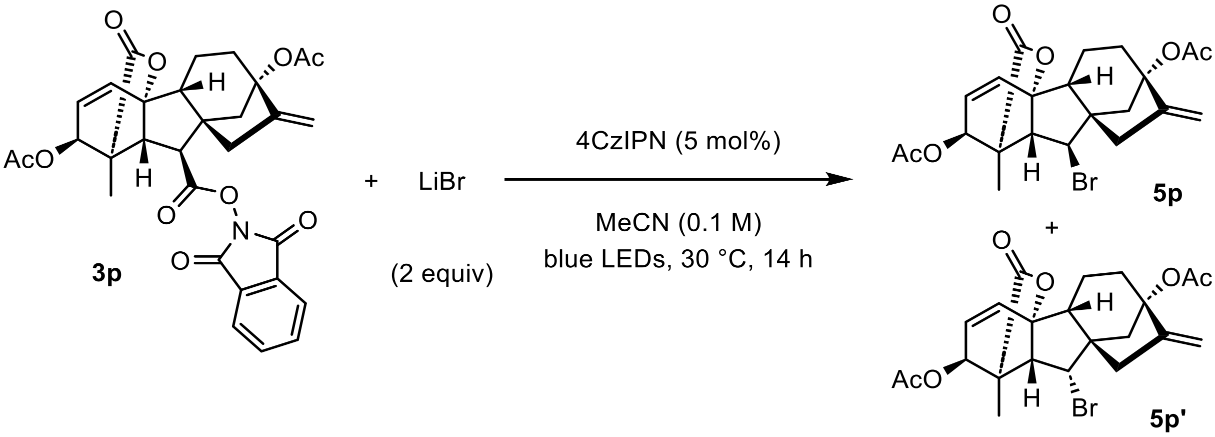

Prepared following **GP2**, using redox active ester **3p** (58 mg, 0.10 mmol), 4CzIPN (4 mg, 0.005 mmol, 5 mol%), and LiBr (18 mg, 0.20 mmol, 2.0 equiv.) in MeCN (1 mL). Purification by flash column chromatography (Biotage, Sfär Silica 10 g, hexane/acetone = 100:0 to 86:14) gave **5p** (26 mg, 56%) as a white solid, and **5p'** (5 mg, 11%) as a white solid.

For **5p**:

NMR Spectroscopy (*see spectra*):

^{1}H NMR (500 MHz, $CDCl_3$): δ_H 6.34 (d, *J* = 9.3 Hz, 1H), 5.86 (dd, *J* = 9.3, 3.8 Hz, 1H), 5.34 (d, *J* = 3.8 Hz, 1H), 5.19 (d, *J* = 2.6 Hz, 1H), 5.04 (d, *J* = 2.6 Hz, 1H), 4.00 (d, *J* = 10.4 Hz, 1H), 3.11 (d, *J* = 10.4 Hz, 1H), 2.93 (dt, *J* = 15.6, 3.1 Hz, 1H), 2.42 – 2.36 (m, 1H), 2.21 – 2.15 (m, 3H), 2.13 (s, 3H), 2.02 (s, 3H), 2.01 – 1.92 (m, 2H), 1.85 – 1.77 (m, 1H), 1.74 – 1.67 (m, 1H), 1.40 (s, 3H) ppm;

^{13}C NMR (125 MHz, $CDCl_3$): δ_C 176.5, 169.9, 169.8, 153.5, 134.2, 129.2, 108.1, 88.7, 84.2, 70.6, 59.4, 52.4, 52.2, 52.0, 49.9, 45.8, 38.2, 36.3, 22.0, 20.8, 17.1, 14.6 ppm.

IR (film): v_{max} 3073, 3045, 2979, 2938, 2881, 1778, 1735, 1452, 1369, 1224, 1155, 1086, 1016, 970, 897 cm^{-1}.

HRMS (ESI^+): m/z calc'd for $C_{22}H_{25}BrO_6Na$ $[M+Na]^+$ 487.0727; found 487.0750.

For **5p'**:

NMR Spectroscopy (*see spectra*):

^{1}H NMR (500 MHz, $CDCl_3$): δ_H 6.35 (d, *J* = 9.2 Hz, 1H), 5.85 (dd, *J* = 9.2, 3.9 Hz, 1H), 5.30 (dd, *J* = 3.9, 0.8 Hz, 1H), 5.15 (dd, *J* = 3.1, 1.3 Hz, 1H), 5.02 – 4.98 (m, 1H), 4.59 (d, *J* = 6.8 Hz, 1H), 3.14 (d, *J* = 6.8 Hz, 1H), 2.71 (dt, *J* = 14.2, 2.9 Hz, 1H), 2.49 – 2.34 (m, 3H), 2.24 (d, *J* = 14.1 Hz, 1H), 2.11 (s, 3H), 2.06 – 1.97 (m, 1H), 2.02 (s, 3H), 1.87 – 1.81 (m, 2H), 1.76 – 1.71 (m, 1H), 1.58 (s, 3H) ppm;

^{13}C NMR (125 MHz, $CDCl_3$): δ_C 177.4, 170.0, 169.9, 154.3, 135.7, 128.7, 108.9, 90.6, 84.4, 72.5, 56.7, 55.2, 55.0, 53.2, 51.6, 47.1, 41.2, 37.4, 22.2, 21.0, 17.3, 15.8 ppm.

IR (film): ν_{max} 3077, 3049, 2929, 2853, 1780, 1738, 1370, 1232, 1150, 1017, 971 cm^{-1}.

HRMS (ESI^+): m/z calc'd for $C_{22}H_{25}BrO_6Na$ $[M+Na]^+$ 487.0727; found 487.0720.

2.6. Decarboxylative Chlorination

tert-Butyl 4-chloropiperidine-1-carboxylate (6a)

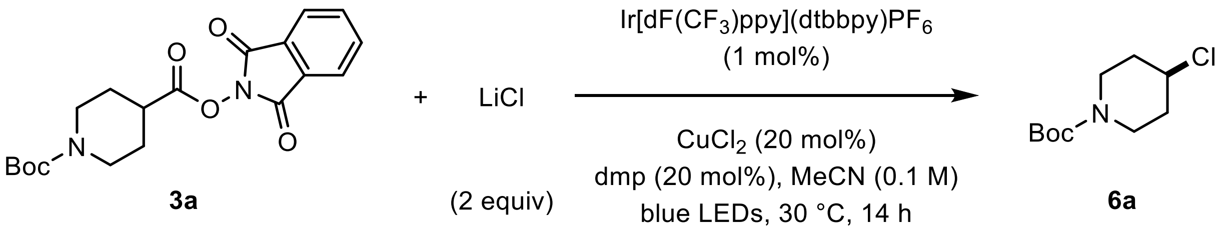

Prepared following **GP3**, using redox active ester **3a** (75 mg, 0.20 mmol), Ir[dF(CF_3)ppy)$_2$(dtbbpy)PF_6 (2.2 mg, 0.0020 mmol, 1.0 mol%), $CuCl_2$ (5.4 mg, 0.040 mmol, 20 mol%), dmp (8.3 mg, 0.040 mmol, 20 mol%), and LiCl (17 mg, 0.40 mmol, 2.0 equiv.) in MeCN (2 mL). Purification by flash column chromatography (Biotage, Sfär Silica 10 g, hexane/Et_2O = 100:0 to 80:20) gave **6a** (35 mg, 80%) as a colourless oil.

NMR Spectroscopy (_see spectra_):

^{1}H NMR (400 MHz, $CDCl_3$): δ_H 4.24 – 4.15 (m, 1H), 3.70 (ddd, J = 13.6, 7.1, 3.8 Hz, 2H), 3.29 (dddd, J = 13.2, 7.9, 3.8, 1.3 Hz, 2H), 2.02 (ddt, J = 14.3, 7.3, 3.7 Hz, 2H), 1.85 – 1.74 (m, 2H), 1.46 (s, 9H) ppm;

^{13}C NMR (100 MHz, $CDCl_3$): δ_C 154.8, 79.9, 57.1, 41.4, 35.1, 28.6 ppm.

All recorded spectroscopic data matched those previously reported in the literature.[19]

Chlorocyclododecane (6b)

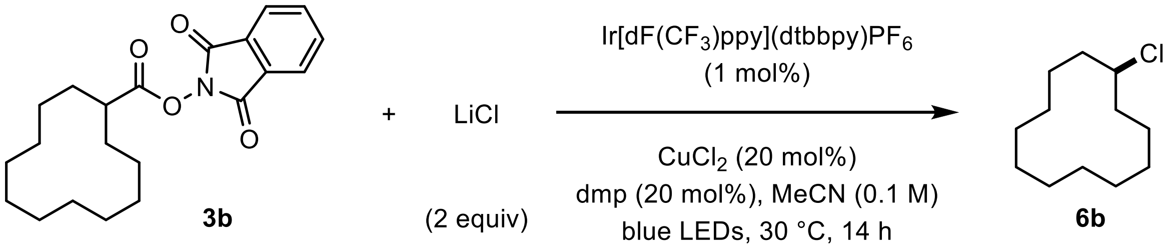

Prepared following **GP3**, using redox active ester **3b** (72 mg, 0.20 mmol), Ir[dF(CF_3)ppy)$_2$(dtbbpy)PF_6 (2.2 mg, 0.0020 mmol, 1.0 mol%), $CuCl_2$ (5.4 mg, 0.040 mmol, 20 mol%), dmp (8.3 mg, 0.040 mmol, 20 mol%), and LiCl (17 mg, 0.40 mmol, 2.0 equiv.) in MeCN (2 mL). Purification by flash column chromatography (Biotage, Sfär Silica 10 g, hexane/Et_2O = 100:0 to 95:5) gave **6b** (29 mg, 72%) as a colourless oil.

NMR Spectroscopy (_see spectra_):

^{1}H NMR (500 MHz, $CDCl_3$): δ_H 4.12 (tt, J = 7.2, 5.2 Hz, 1H), 1.99 – 1.89 (m, 2H), 1.79 – 1.70 (m, 2H), 1.58 – 1.48 (m, 2H), 1.46 – 1.27 (m, 16H) ppm;

^{13}C NMR (125 MHz, $CDCl_3$): δ_C 60.4, 34.0, 23.9, 23.8, 23.6, 23.5, 22.0 ppm.

All recorded spectroscopic data matched those previously reported in the literature.[20]

(1*r*,4*r*)-4-Chlorocyclohexan-1-ol (6c)

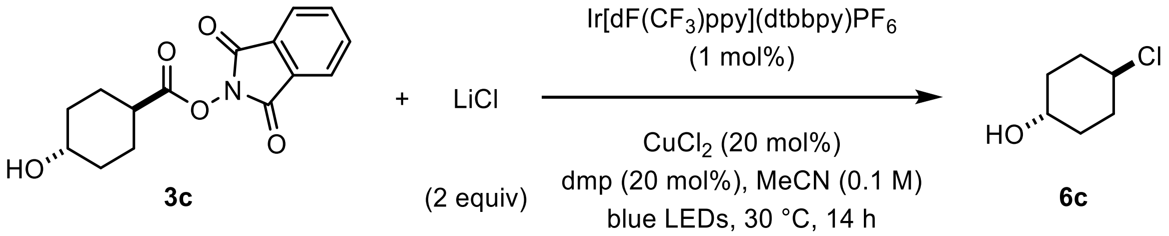

Prepared following **GP3**, using redox active ester **3c** (43 mg, 0.15 mmol), Ir[dF(CF_3)ppy)$_2$(dtbbpy)PF_6 (1.7 mg, 0.0015 mmol, 1.0 mol%), $CuCl_2$ (4.0 mg, 0.030 mmol, 20 mol%), dmp (6.2 mg, 0.030 mmol, 20 mol%), and LiCl (12 mg, 0.30 mmol, 2.0 equiv.) in MeCN (1.5 mL). Purification by flash column chromatography (Biotage, Sfär Silica 10 g, hexane/EtOAc = 100:0 to 65:35) gave **6c** (13 mg, 64%) as a colourless oil. The d.r. was determined to be 63:37 by ^{1}H NMR analysis of the purified product.

NMR Spectroscopy (*see spectra*):

^{1}**H NMR** (500 MHz, $CDCl_3$, mixture of diastereomers): δ_H 4.25 – 4.13 (m, 0.63H), 3.98 (tt, J = 9.3, 4.0 Hz, 0.37H), 3.82 – 3.70 (m, 1H), 2.25 – 2.18 (m, 0.63H), 2.10 – 1.98 (m, 2H), 1.90 – 1.78 (m, 2.37), 1.78 – 1.64 (m, 2H), 1.46 – 1.37 (m, 1H) ppm;

^{13}**C NMR** (125 MHz, $CDCl_3$, mixture of diastereomers): δ_C 68.4 (minor), 68.0 (major), 58.8 (minor), 58.5 (major), 33.5 (major), 33.2 (minor), 32.1 (major), 30.9 (minor) ppm.

All recorded spectroscopic data matched those previously reported in the literature.[21]

***tert*-Butyl (*S*)-2-(chloromethyl)pyrrolidine-1-carboxylate (6g)**

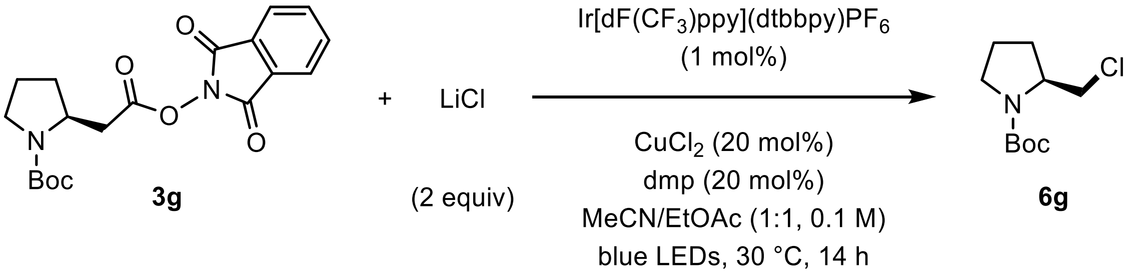

Prepared following a modified **GP3**, using redox active ester **3g** (75 mg, 0.20 mmol), Ir[dF(CF_3)ppy)$_2$(dtbbpy)PF_6 (2.2 mg, 0.0020 mmol, 1.0 mol%), $CuCl_2$ (5.4 mg, 0.040 mmol, 20 mol%), dmp (8.3 mg, 0.040 mmol, 20 mol%), and LiCl (17 mg, 0.40 mmol, 2.0 equiv.) in MeCN (1 mL) and EtOAc (1 mL). Purification by flash column chromatography (Biotage, Sfär Silica 10 g, hexane/Et_2O = 100:0 to 75:25) gave **6g** (27 mg, 61%) as a colourless oil.

NMR Spectroscopy (*see spectra*):

^{1}**H NMR** (400 MHz, $CDCl_3$): δ_H 4.10 – 3.88 (m, 1H), 3.78 – 3.62 (m, 1H), 3.59 – 3.29 (m, 3H), 2.05 – 1.95 (m, 2H), 1.95 – 1.75 (m, 2H), 1.46 (s, 9H) ppm;

^{13}**C NMR** (100 MHz, $CDCl_3$): δ_C 154.6, 79.9, 58.2, 47.4 + 47.0 (rotameric peaks), 45.6, 29.4 + 28.50 (rotameric peaks), 28.6, 23.7 + 22.9 (rotameric peaks) ppm.

All recorded spectroscopic data matched those previously reported in the literature.[20]

2-(3-Chloropropyl)thiophenecarboxylate (6h)

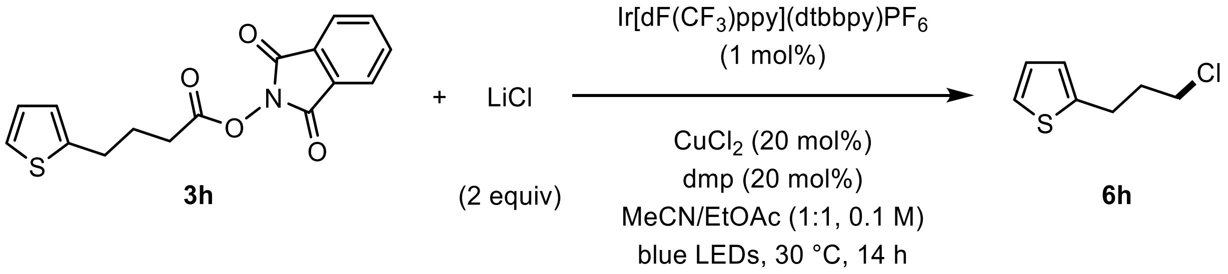

Prepared following a modified **GP3**, using redox active ester **3h** (63 mg, 0.20 mmol), Ir[dF(CF_3)ppy)$_2$(dtbbpy)PF_6 (2.2 mg, 0.0020 mmol, 1.0 mol%), $CuCl_2$ (5.4 mg, 0.040 mmol, 20 mol%), dmp (8.3 mg, 0.040 mmol, 20 mol%), and LiCl (17 mg, 0.40 mmol, 2.0 equiv.) in MeCN (1 mL) and EtOAc (1 mL). Purification by flash column chromatography (Biotage, Sfär Silica 10 g, hexane/Et_2O = 100:0 to 90:10) gave **6h** (12 mg, 37%) as a colourless oil.

NMR Spectroscopy (*see spectra*):

^{1}**H NMR** (400 MHz, $CDCl_3$): δ_H 7.14 (d, J = 5.2 Hz, 1H), 6.96 – 6.91 (m, 1H), 6.83 (d, J = 3.7 Hz, 1H), 3.57 (t, J = 6.4 Hz, 2H), 3.02 (t, J = 7.3 Hz, 2H), 2.18 – 2.08 (m, 2H) ppm;

^{13}**C NMR** (100 MHz, $CDCl_3$): δ_C 143.3, 127.0, 125.0, 123.6, 44.0, 34.4, 27.0 ppm.

IR (film): ν_{max} 2958, 1441, 1250, 895, 849, 730 cm^{-1}.

HRMS (ESI$^+$): m/z calc'd for C_7H_9ClS [M+H]$^+$ 161.0186; found 161.0183.

4-Chloro-1-phenylbutan-1-one (6i)

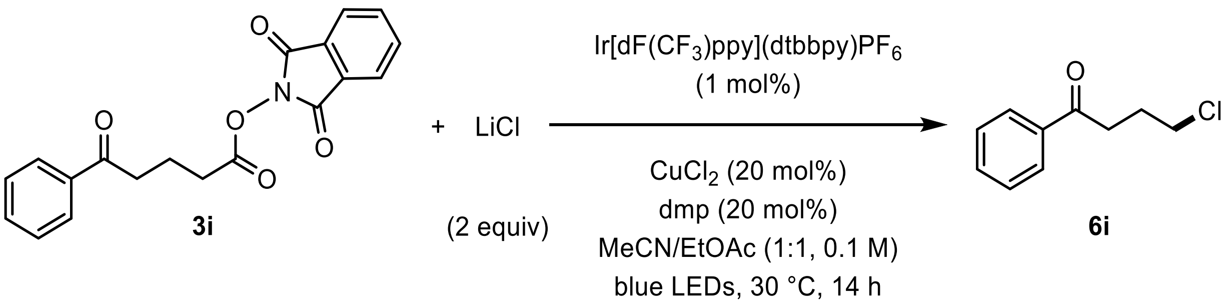

Prepared following a modified **GP3**, using redox active ester **3i** (68 mg, 0.20 mmol), Ir[dF(CF_3)ppy)$_2$(dtbbpy)PF_6 (2.2 mg, 0.0020 mmol, 1.0 mol%), $CuCl_2$ (5.4 mg, 0.040 mmol, 20 mol%), dmp (8.3 mg, 0.040 mmol, 20 mol%), and LiCl (17 mg, 0.40 mmol, 2.0 equiv.) in MeCN (1 mL) and EtOAc (1 mL). Purification by flash column

chromatography (Biotage, Sfär Silica 10 g, hexane/EtOAc = 100:0 to 75:25) gave **6i** (20 mg, 55%) as a colourless oil.

NMR Spectroscopy (*see spectra*):

> **^{1}H NMR** (400 MHz, $CDCl_3$): δ_H 8.01 – 7.96 (m, 2H), 7.60 – 7.54 (m, 1H), 7.51 – 7.44 (m, 2H), 3.68 (t, *J* = 6.2 Hz, 2H), 3.19 (t, *J* = 7.0 Hz, 2H), 2.29 – 2.19 (m, 2H) ppm;
>
> **^{13}C NMR** (100 MHz, $CDCl_3$): δ_C 199.1, 136.9, 133.4, 128.8, 128.2, 44.8, 35.4, 26.9 ppm.

All recorded spectroscopic data matched those previously reported in the literature.[15]

4-(2-Chloroethyl)-1,2-dimethoxybenzene (6j)

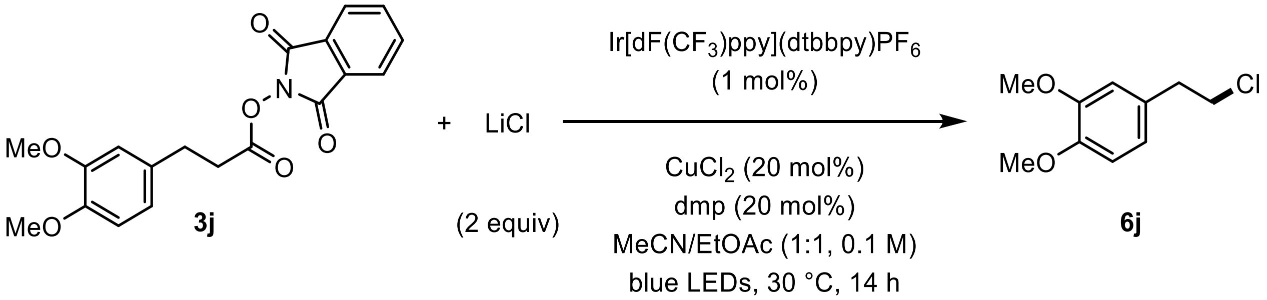

Prepared following a modified **GP3**, using redox active ester **3j** (36 mg, 0.10 mmol), Ir[dF(CF_3)ppy)$_2$(dtbbpy)PF_6 (1.1 mg, 0.0010 mmol, 1.0 mol%), $CuCl_2$ (2.7 mg, 0.020 mmol, 20 mol%), dmp (4.2 mg, 0.020 mmol, 20 mol%), and LiCl (8.5 mg, 0.20 mmol, 2.0 equiv.) in MeCN (0.5 mL) and EtOAc (0.5 mL). Purification by flash column chromatography (Biotage, Sfär Silica 10 g, hexane/EtOAc = 100:0 to 70:30) gave **6j** (10 mg, 50%) as a colourless oil.

Note: When acetone (1 mL) was used as the solvent instead of MeCN/EtOAc (1:1, 1 mL), **6j** was formed in an improved yield of 73% (15 mg)

NMR Spectroscopy (*see spectra*):

> **^{1}H NMR** (500 MHz, $CDCl_3$): δ_H 6.82 (d, *J* = 8.1 Hz, 1H), 6.78 – 6.71 (m, 2H), 3.88 (s, 3H), 3.86 (s, 3H), 3.69 (t, *J* = 7.5 Hz, 2H), 3.01 (t, *J* = 7.5 Hz, 2H) ppm;
>
> **^{13}C NMR** (125 MHz, $CDCl_3$): δ_C 149.1, 148.1, 130.8, 121.0, 112.2, 111.4, 56.04, 56.00, 45.3, 39.0 ppm.

All recorded spectroscopic data matched those previously reported in the literature.[22]

1-Chloroheptadecane (6k)

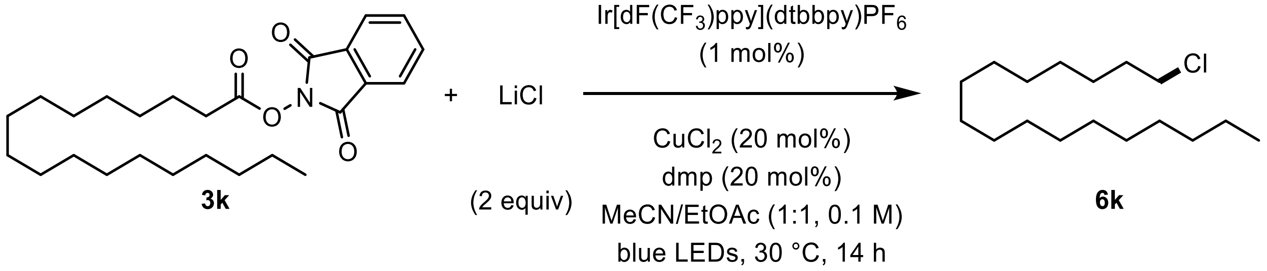

Prepared following a modified **GP3**, using redox active ester **3k** (86 mg, 0.20 mmol), Ir[dF(CF_3)ppy)$_2$(dtbbpy)PF_6 (2.2 mg, 0.0020 mmol, 1.0 mol%), $CuCl_2$ (5.4 mg, 0.040 mmol, 20 mol%), dmp (8.3 mg, 0.040 mmol, 20 mol%), and LiCl (17 mg, 0.40 mmol, 2.0 equiv.) in MeCN (1 mL) and EtOAc (1 mL). Purification by flash column chromatography (Biotage, Sfär Silica 10 g, pentane/Et_2O = 100:0 to 95:5) gave **6k** (48 mg, 87%) as a colourless oil.

NMR Spectroscopy (*see spectra*):

^{1}**H NMR** (500 MHz, $CDCl_3$): δ_H 3.53 (t, *J* = 6.8 Hz, 2H), 1.80 – 1.73 (m, 2H), 1.42 (p, *J* = 7.8, 7.1 Hz, 2H), 1.26 (s, 26H), 0.88 (t, *J* = 7.0 Hz, 3H) ppm;

^{13}**C NMR** (125 MHz, $CDCl_3$): δ_C 45.3, 32.8, 32.1, 29.85 (4C), 29.83, 29.82, 29.78, 29.7, 29.6, 29.5, 29.1, 27.1, 22.9, 14.3 ppm.

All recorded spectroscopic data matched those previously reported in the literature.[23]

Methyl (1*R*)-3-chloro-1,2,2-trimethylcyclopentane-1-carboxylate (6l)

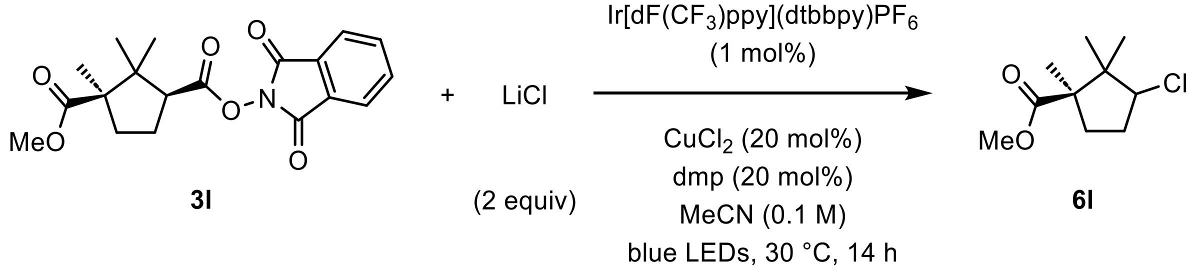

Prepared following **GP3**, using redox active ester **3l** (72 mg, 0.20 mmol), Ir[dF(CF_3)ppy)$_2$(dtbbpy)PF_6 (2.2 mg, 0.0020 mmol, 1.0 mol%), $CuCl_2$ (5.4 mg, 0.040 mmol, 20 mol%), dmp (8.3 mg, 0.040 mmol, 20 mol%), and LiCl (17 mg, 0.40 mmol, 2.0 equiv.) in MeCN (2 mL). Purification by flash column chromatography (Biotage, Sfär Silica 10 g, hexane/Et_2O = 100:0 to 95:5) gave **6l** (33 mg, 81%) as a colourless oil. The d.r. was determined to be 73:27 by ^{1}H NMR analysis of the purified product.

NMR Spectroscopy (*see spectra*):

^{1}**H NMR** (500 MHz, $CDCl_3$, major diastereomer): δ_H 4.27 (t, *J* = 8.8 Hz, 1H), 3.65 (s, 3H), 2.38 (dddd, *J* = 13.2, 9.8, 8.4, 4.7 Hz, 1H), 2.22 (ddd, *J* = 13.6, 9.8, 5.6 Hz, 1H), 1.95 – 1.83 (m, 1H), 1.61 (ddd, *J* = 13.7,

11.9, 4.7 Hz, 1H), 1.23 (s, 3H), 0.98 (s, 3H), 0.90 (s, 3H) ppm;

^{1}H NMR (500 MHz, $CDCl_3$, minor diastereomer): δ_H 4.07 (dd, *J* = 10.3, 8.6 Hz, 1H), 3.69 (s, 3H), 2.68 (td, *J* = 13.3, 4.8 Hz, 1H), 2.25 – 2.19 (m, 1H), 1.95 – 1.83 (m, 1H), 1.45 (ddd, *J* = 13.8, 9.6, 6.0 Hz, 1H), 1.19 (s, 3H), 1.13 (s, 3H), 0.87 (s, 3H) ppm;

^{13}C NMR (125 MHz, $CDCl_3$, major diastereomer): δ_C 177.7, 68.4, 54.2, 51.8, 48.0, 33.1, 32.2, 22.4, 20.8, 19.0 ppm.

^{13}C NMR (125 MHz, $CDCl_3$, minor diastereomer): δ_C 176.3, 68.0, 53.3, 51.9, 47.0, 31.3, 30.7, 22.8, 20.8, 19.5 ppm.

IR (film): ν_{max} 2955, 2922, 2851, 1738, 1460, 1257, 750 cm^{-1}.

((3*R*,5*R*,8*R*,9*S*,10*S*,13*R*,14*S*,17*R*)-17-((*R*)-4-Chlorobutan-2-yl)-10,13-dimethylhexadecahydro-1*H*-cyclopenta[*a*]phenanthren-3-ol (6m)

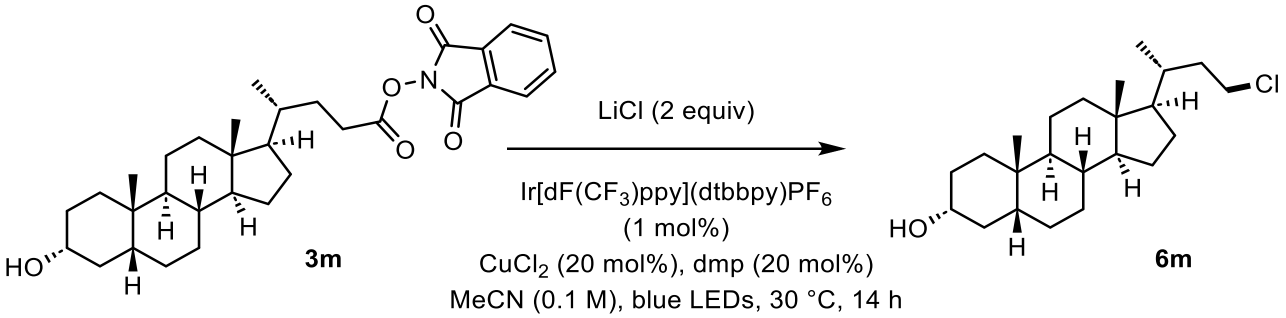

Prepared following **GP3**, using redox active ester **3m** (52 mg, 0.10 mmol), Ir[dF(CF_3)ppy)$_2$(dtbbpy)PF_6 (1.1 mg, 0.0010 mmol, 1.0 mol%), $CuCl_2$ (2.7 mg, 0.020 mmol, 20 mol%), dmp (4.2 mg, 0.020 mmol, 20 mol%), and LiCl (8.5 mg, 0.20 mmol, 2.0 equiv.) in MeCN (1 mL). Purification by flash column chromatography (Biotage, Sfär Silica 10 g, hexane/acetone = 100:0 to 85:15) gave **6m** (29 mg, 78%) as a white solid.

NMR Spectroscopy (*see spectra*):

^{1}H NMR (500 MHz, $CDCl_3$): δ_H 3.67 – 3.57 (m, 2H), 3.50 (ddd, *J* = 10.7, 8.5, 7.6 Hz, 1H), 1.96 (dt, *J* = 12.6, 3.3 Hz, 1H), 1.93 – 1.89 (m, 1H), 1.89 – 1.78 (m, 3H), 1.77 – 1.71 (m, 1H), 1.69 – 1.63 (m, 1H), 1.62 – 1.53 (m, 3H), 1.53 – 1.42 (m, 3H), 1.41 – 1.35 (m, 4H), 1.35 – 1.30 (m, 1H), 1.25 – 1.18 (m, 3H), 1.18 – 1.13 (m, 1H), 1.13 – 1.00 (m, 4H), 0.98 (dd, *J* = 14.2, 3.4 Hz, 1H), 0.93 (d, *J* = 6.6 Hz, 3H), 0.92 (s, 3H), 0.66 (s, 3H) ppm;

^{13}C NMR (125 MHz, $CDCl_3$): δ_C 72.0, 56.7, 56.3, 43.3, 43.0, 42.2, 40.6, 40.3, 39.1, 36.6, 36.0, 35.5, 34.7, 34.0, 30.7, 28.4, 27.3, 26.5, 24.3, 23.5, 21.0, 18.4, 12.2 ppm.

IR (film): ν_{max} 3321 (br), 2926, 2862, 1447, 1376, 1263, 1067, 1033, 1014, 938, 658, 613 cm^{-1}.

HRMS (ESI$^+$): m/z calc'd for $C_{23}H_{39}ClONa$ [M+Na]$^+$ 389.2582; found 389.2567.

Benzyl (*R*)-2-((*tert*-butoxycarbonyl)amino)-4-chlorobutanoate (6n)

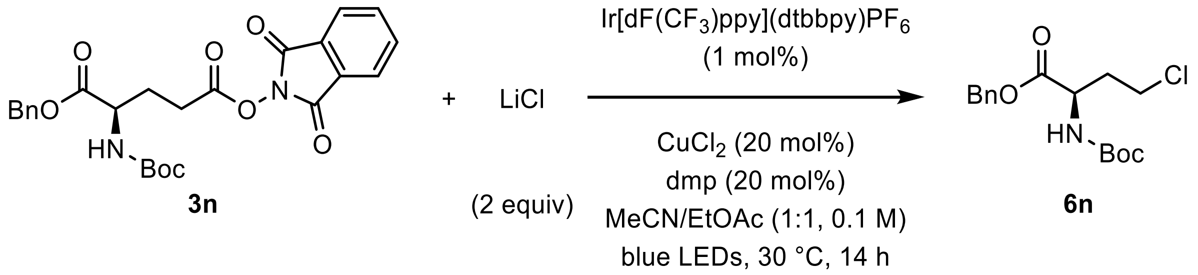

Prepared following a modified **GP3**, using redox active ester **3n** (72 mg, 0.15 mmol), Ir[dF(CF$_3$)ppy)$_2$(dtbbpy)PF$_6$ (1.7 mg, 0.0015 mmol, 1.0 mol%), CuCl$_2$ (4.0 mg, 0.030 mmol, 20 mol%), dmp (6.2 mg, 0.040 mmol, 20 mol%), and LiCl (12 mg, 0.30 mmol, 2.0 equiv.) in MeCN (0.75 mL) and EtOAc (0.75 mL). Purification by flash column chromatography (Biotage, Sfär Silica 10 g, hexane/EtOAc = 100:0 to 75:25) gave **6n** (27.5 mg, 56%) as a colourless oil.

NMR Spectroscopy (*see spectra*):

^{1}**H NMR** (500 MHz, CDCl$_3$): δ_H 7.43 – 7.30 (m, 5H), 5.20 (d, *J* = 12.2 Hz, 1H), 5.17 (d, *J* = 12.2 Hz, 1H), 5.13 (br d, *J* = 7.9 Hz, 1H), 4.56 – 4.36 (br m, 1H), 3.57 (t, *J* = 6.9 Hz, 2H), 2.40 – 2.26 (m, 1H), 2.14 (dq, *J* = 13.7, 6.3 Hz, 1H), 1.44 (s, 9H) ppm;

^{13}**C NMR** (125 MHz, CDCl$_3$): δ_C 171.9, 155.4, 135.3, 128.8, 128.7, 128.5, 80.4, 67.6, 51.7, 40.7, 35.6, 28.4 ppm.

IR (film): ν_{max} 3359 (br), 3092, 3069, 3033, 3006, 2977, 2931, 1710, 1499, 1366, 1251, 1159, 1050, 1026, 750, 697 cm^{-1}.

HRMS (ESI$^+$): m/z calc'd for $C_{16}H_{22}ClNO_4Na$ [M+Na]$^+$ 350.1130; found 350.1137.

(1*S*,2*S*,4a*R*,4b*R*,7*S*,9a*S*,10*S*,10a*R*)-10-Chloro-2,7-dihydroxy-1-methyl-8-methylene-1,2,4b,5,6,7,8,9,10,10a-decahydro-4a,1-(epoxymethano)-7,9a-methanobenzo[*a*]azulen-13-one (6o)

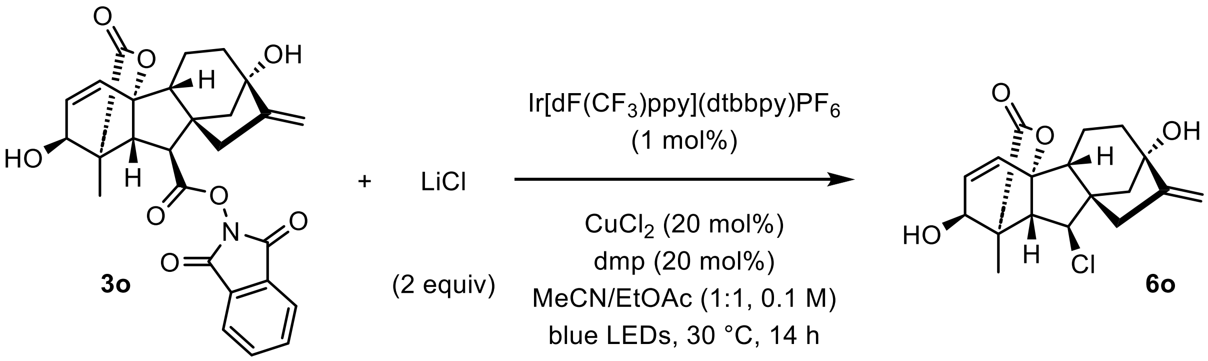

Prepared following a modified **GP3**, using redox active ester **3o** (49 mg, 0.10 mmol), Ir[dF(CF$_3$)ppy)$_2$(dtbbpy)PF$_6$ (1.1 mg, 0.0010 mmol, 1.0 mol%), CuCl$_2$ (2.7 mg, 0.020 mmol, 20 mol%), dmp (4.2 mg, 0.020 mmol, 20 mol%), and LiCl (8.5 mg, 0.20 mmol, 2.0 equiv.) in MeCN (0.5 mL) and EtOAc (0.5 mL). Purification by flash column

chromatography (Biotage, Sfär Silica 10 g, hexane/EtOAc = 100:0 to 50:50) gave **6o** (22 mg, 65%) as a white solid. The d.r. was determined to be >95:5 by ^{1}H NMR analysis of the purified product. Whilst only a single diastereomer was isolated, we cannot exclude the possibility that the minor diastereomer was removed during purification; therefore, the reported d.r. may not reflect the inherent diastereoselectivity of the reaction.

NMR Spectroscopy (*see spectra*):

^{1}H NMR (500 MHz, CD_3CN): δ_H 6.25 (d, *J* = 9.3 Hz, 1H), 5.78 (dd, *J* = 9.3, 3.7 Hz, 1H), 5.13 (dd, *J* = 3.2, 1.7 Hz, 1H), 4.90 (dd, *J* = 2.7, 1.5 Hz, 1H), 4.08 (d, *J* = 9.8 Hz, 1H), 3.98 (dd, *J* = 6.8, 3.8 Hz, 1H), 3.56 (d, *J* = 6.7 Hz, 1H), 2.92 (s, 1H), 2.86 (d, *J* = 9.8 Hz, 1H), 2.68 (dt, *J* = 15.8, 3.1 Hz, 1H), 2.22 – 2.16 (m, 1H), 2.01 (dd, *J* = 11.3, 5.5 Hz, 1H), 1.95 – 1.89 (m, 1H), 1.76 – 1.61 (m, 4H), 1.52 (d, *J* = 10.7 Hz, 1H), 1.30 (s, 3H) ppm;

^{13}C NMR (125 MHz, CD_3CN): δ_C 179.2, 158.6, 133.7, 132.9, 107.0, 89.7, 78.6, 70.4, 64.0, 59.5, 54.1, 52.2, 50.6, 44.0, 43.7, 39.6, 17.8, 15.0 ppm.

IR (film): ν_{max} 3392 (br), 3077, 3041, 2975, 2937, 2877, 1761, 1705, 1453, 1380, 1252, 1159, 1102, 1044, 1012, 889, 773 cm^{-1}.

HRMS (ESI$^+$): m/z calc'd for $C_{18}H_{21}ClO_4Na$ $[M+Na]^+$ 359.1021; found 359.1021.

(1*S*,2*S*,4a*R*,4b*R*,7*S*,9a*S*,10*S*,10a*R*)-10-Chloro-1-methyl-8-methylene-13-oxo-1,2,5,6,8,9,10,10a-octahydro-4a,1-(epoxymethano)-7,9a-methanobenzo[*a*]azulene-2,7(4b*H*)-diyl diacetate (6p)

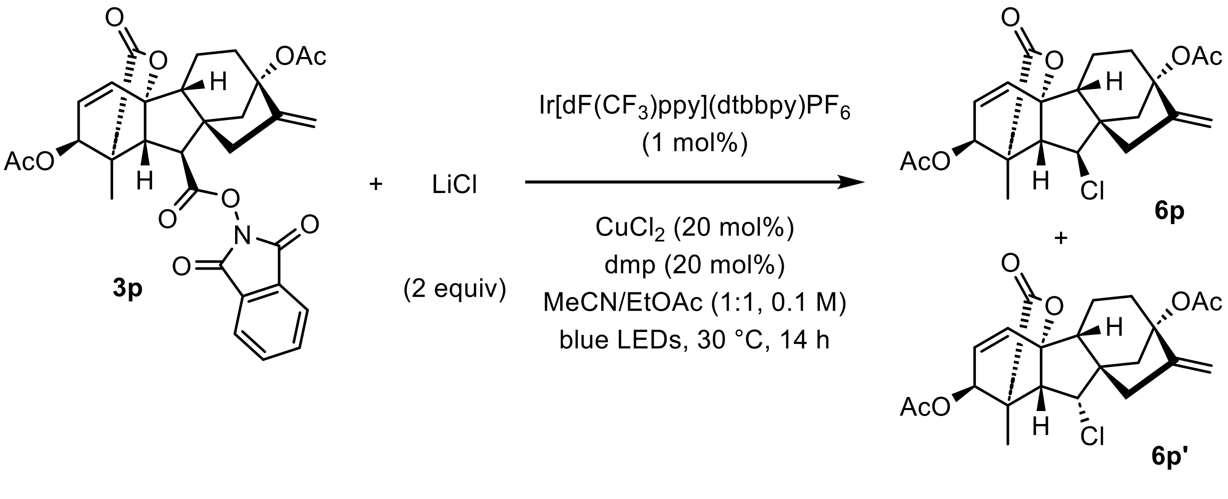

Prepared following a modified **GP3**, using redox active ester **3p** (58 mg, 0.10 mmol), $Ir[dF(CF_3)ppy)_2(dtbbpy)PF_6$ (1.1 mg, 0.0010 mmol, 1.0 mol%), $CuCl_2$ (2.7 mg, 0.020 mmol, 20 mol%), dmp (4.2 mg, 0.020 mmol, 20 mol%), and LiCl (8.5 mg, 0.20 mmol, 2.0 equiv.) in MeCN (0.5 mL) and EtOAc (0.5 mL). Purification by flash column chromatography (Biotage, Sfär Silica 10 g, hexane/EtOAc = 100:0 to 50:50) gave **6p** (29 mg, 69%) as a white solid, and **8p'** (10 mg, 23%) as a white solid.

For **6p**:

NMR Spectroscopy (*see spectra*):

^{1}H NMR (500 MHz, $CDCl_3$): δ_H 6.35 (d, *J* = 9.2 Hz, 1H), 5.88 (dd, *J* = 9.2, 3.7 Hz, 1H), 5.35 (d, *J* = 3.6 Hz, 1H), 5.21 (dd, *J* = 3.2, 1.5 Hz, 1H), 5.05 (dd, *J* = 2.9, 1.4 Hz, 1H), 3.99 (d, *J* = 10.1 Hz, 1H), 2.96 (d, *J* = 10.1 Hz, 1H), 2.92 (dt, *J* = 15.3, 2.9 Hz, 1H), 2.40 (dd, *J* = 12.4, 7.7 Hz, 1H), 2.13 – 2.24 (m, 3H), 2.14 (s, 3H), 2.04 (s, 3H), 2.02 – 1.92 (m, 2H), 1.85 – 1.78 (m, 1H), 1.74 – 1.68 (m, 1H), 1.38 (s, 3H) ppm;

^{13}C NMR (125 MHz, $CDCl_3$): δ_C 176.7, 170.00, 169.97, 153.4, 134.3, 129.4, 108.3, 88.2, 84.2, 70.6, 61.9, 59.3, 52.3, 52.2, 50.2, 43.2, 38.4, 36.5, 22.2, 20.9, 17.1, 14.6 ppm.

IR (film): ν_{max} 3080, 3045, 2979, 2938, 2881, 1781, 1737, 1453, 1369, 1226, 1156, 1087, 1017, 971, 896, 774 cm^{-1}.

HRMS (ESI^+): m/z calc'd for $C_{22}H_{25}ClO_6Na$ $[M+Na]^+$ 443.1232, found 443.1250.

For **6p'**:

NMR Spectroscopy (*see spectra*):

^{1}H NMR (500 MHz, $CDCl_3$): δ_H 6.35 (d, *J* = 9.2 Hz, 1H), 5.85 (dd, *J* = 9.3, 3.8 Hz, 1H), 5.31 (d, *J* = 3.8 Hz, 1H), 5.16 (d, *J* = 3.0 Hz, 1H), 5.00 (s, 1H), 4.54 (d, *J* = 6.7 Hz, 1H), 3.16 (d, *J* = 6.7 Hz, 1H), 2.70 (d, *J* = 14.1 Hz, 1H), 2.46 – 2.33 (m, 3H), 2.23 (d, *J* = 14.2 Hz, 1H), 2.12 (s, 3H), 2.07 – 1.97 (m, 1H), 2.03 (s, 3H), 1.87 – 1.79 (m, 2H), 1.72 (dt, *J* = 13.0, 6.3 Hz, 1H), 1.54 (s, 3H) ppm;

^{13}C NMR (125 MHz, $CDCl_3$): δ_C 177.4, 170.0, 169.9, 154.0, 135.6, 128.8, 108.7, 90.6, 84.3, 72.3, 65.7, 55.7, 55.4, 51.9, 51.5, 47.1, 38.1, 37.4, 22.2, 21.0, 17.2, 15.4 ppm.

IR (film): ν_{max} 3080, 3041, 2983, 2938, 2885, 2849, 1780, 1738, 1370, 1229, 1152, 1016, 971, 897 cm^{-1}.

HRMS (ESI^+): m/z calc'd for $C_{22}H_{25}ClO_6Na$ $[M+Na]^+$ 443.1232, found 443.1232.

2.7. Decarboxylative Thiocyanation

tert-Butyl 4-thiocyanatopiperidine-1-carboxylate (7a)

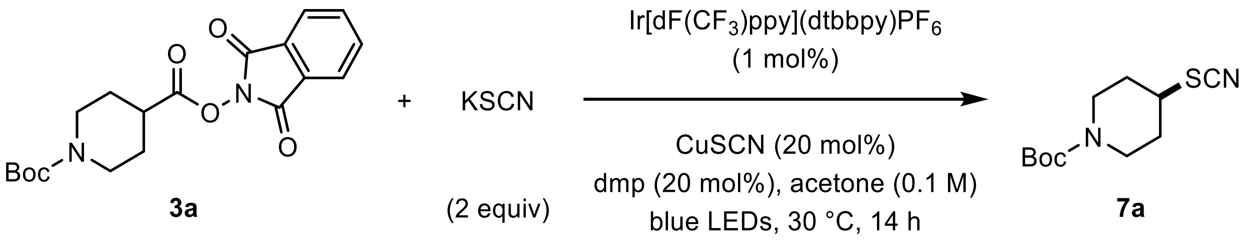

Prepared following **GP4**, using redox active ester **3a** (75 mg, 0.20 mmol), Ir[dF(CF_3)ppy$)_2$(dtbbpy)PF_6 (2.2 mg, 0.0020 mmol, 1.0 mol%), CuSCN (4.9 mg, 0.040 mmol, 20 mol%), dmp (8.3 mg, 0.040 mmol, 20 mol%), and KSCN (39 mg, 0.40 mmol, 2.0 equiv.) in acetone (2 mL). Purification by flash column chromatography (Biotage, Sfär Silica 10 g, hexane/acetone = 100:0 to 80:20) gave **7a** (38 mg, 79%) as a colourless oil.

NMR Spectroscopy (*see spectra*):

^{1}**H NMR** (400 MHz, $CDCl_3$): δ_H 4.06 (br d, *J* = 12.0 Hz, 2H), 3.36 (tt, *J* = 10.8, 3.9 Hz, 1H), 2.93 (br t, *J* = 12.5 Hz, 2H), 2.08 (dd, *J* = 13.1, 3.1 Hz, 2H), 1.78 – 1.68 (m, 2H), 1.45 (s, 9H) ppm;

^{13}**C NMR** (100 MHz, $CDCl_3$): δ_C 154.4, 110.7, 80.3, 45.4, 43.1 (br), 32.5, 28.4 ppm.

IR (film): ν_{max} 2998, 2955, 2933, 2834, 2180, 2097, 1516, 1463, 1263, 1238, 1157, 1141, 1028 cm^{-1}.

HRMS (ESI$^+$): m/z calc'd for $C_{11}H_{18}N_2O_2SNa$ [M+Na]$^+$ 265.0981; found 265.0983.

Thiocyanatocyclododecane (7b)

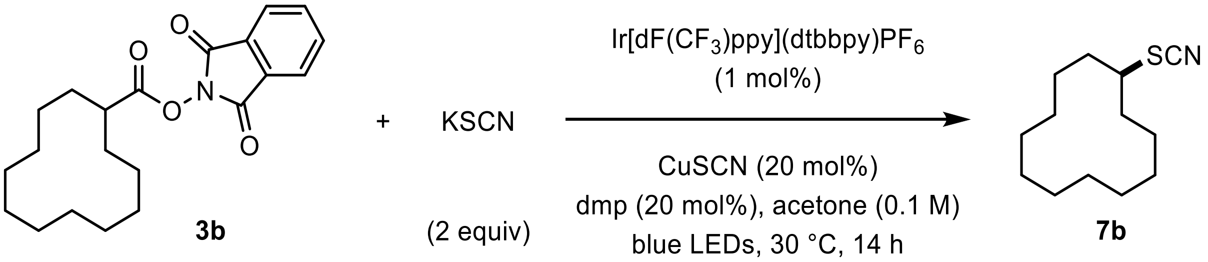

Prepared following **GP4**, using redox active ester **3b** (72 mg, 0.20 mmol), Ir[dF(CF_3)ppy$)_2$(dtbbpy)PF_6 (2.2 mg, 0.0020 mmol, 1.0 mol%), CuSCN (4.9 mg, 0.040 mmol, 20 mol%), dmp (8.3 mg, 0.040 mmol, 20 mol%), and KSCN (39 mg, 0.40 mmol, 2.0 equiv.) in acetone (2 mL). Purification by flash column chromatography (Biotage, Sfär Silica 10 g, pentane/Et_2O = 100:0 to 95:5) gave **7b** (33 mg, 73%) as a colourless oil.

NMR Spectroscopy (*see spectra*):

^{1}**H NMR** (500 MHz, $CDCl_3$): δ_H 3.37 – 3.30 (m, 1H), 1.93 (dq, *J* = 13.8, 6.9 Hz, 2H), 1.78 – 1.68 (m, 2H), 1.60 – 1.50 (m, 2H), 1.50 – 1.31 (m, 16H) ppm;

^{13}**C NMR** (125 MHz, $CDCl_3$): δ_C 112.2, 47.4, 31.1, 23.8, 23.6, 23.6 (2C), 22.3 ppm.

IR (film): ν_{max} 2930, 2861, 2151, 1469, 1445, 1249, 754, 719 cm^{-1}.

HRMS (EI$^+$): m/z calc'd for $C_{13}H_{23}NS$ [M-H]$^+$ 224.1467; found 224.1468.

4-Thiocyanatocyclohexan-1-ol (7c)

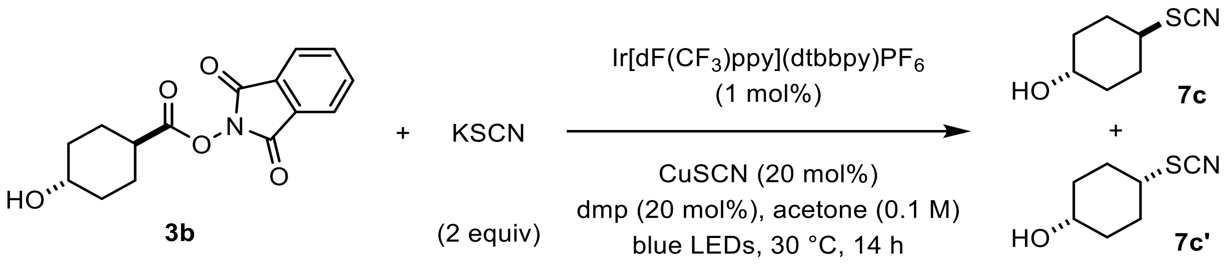

Prepared following **GP4**, using redox active ester **3c** (43 mg, 0.15 mmol), Ir[dF(CF_3)ppy)$_2$(dtbbpy)PF_6 (1.7 mg, 0.0015 mmol, 1.0 mol%), CuSCN (3.6 mg, 0.030 mmol, 20 mol%), dmp (6.2 mg, 0.020 mmol, 20 mol%), and KSCN (29 mg, 0.30 mmol, 2.0 equiv.) in acetone (1.5 mL). Purification by flash column chromatography (Biotage, Sfär Silica 10 g, pentane/Et_2O = 100:0 to 45:55) gave **7c** (7 mg, 30%) as a colourless oil, and **7c'** (6 mg, 25%) as a colourless oil.

For **7c**:

NMR Spectroscopy (*see spectra*):

^{1}**H NMR** (500 MHz, $CDCl_3$): δ_H 3.96 (tt, *J* = 5.7, 3.1 Hz, 1H), 3.42 (tt, *J* = 9.3, 3.9 Hz, 1H), 2.07 – 1.99 (m, 2H), 1.97 – 1.90 (m, 2H), 1.83 (dq, *J* = 14.0, 4.3 Hz, 2H), 1.74 – 1.66 (m, 2H), 1.52 (br s, 1H) ppm;

^{13}**C NMR** (125 MHz, $CDCl_3$): δ_C 111.8, 65.7, 47.2, 32.1, 28.0 ppm.

IR (film): ν_{max} 3424 (br), 2937, 2861, 2152, 1725, 1446, 1361, 1262, 1065, 1029, 761, 750 cm^{-1}.

HRMS (ESI$^+$): m/z calc'd for $C_7H_{11}NOSNa$ [M+Na]$^+$ 180.0454; found 180.0462.

For **7c'**:

NMR Spectroscopy (*see spectra*):

^{1}**H NMR** (500 MHz, $CDCl_3$): δ_H 3.71 (tt, *J* = 9.8, 4.2 Hz, 1H), 3.19 (tt, *J* = 11.3, 3.9 Hz, 1H), 2.22 (dd, *J* = 14.4, 3.9 Hz, 2H), 2.08 (dd, *J* = 13.7, 4.3 Hz, 2H), 1.67 (tdd, *J* = 13.8, 11.6, 3.5 Hz, 2H), 1.43 (tdd, *J* = 13.0, 10.2, 3.9 Hz, 2H) ppm;

^{13}**C NMR** (125 MHz, $CDCl_3$): δ_C 111.3, 68.6, 46.3, 34.5, 31.3 ppm.

IR (film): ν_{max} 3423 (br), 2937, 2861, 2152, 1742, 1715, 1499, 1366, 1275, 1260, 1161, 750, 698 cm^{-1}.

HRMS (ESI$^+$): m/z calc'd for $C_7H_{11}NOSNa$ [M+Na]$^+$ 180.0454; found 180.0454.

2-(3-Chloropropyl)thiophene (7h)

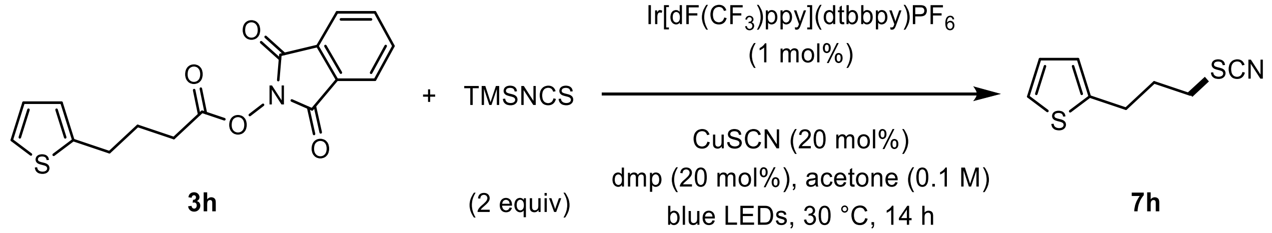

Prepared following **GP4**, using redox active ester **3h** (63 mg, 0.20 mmol), Ir[dF(CF$_3$)ppy)$_2$(dtbbpy)PF$_6$ (2.2 mg, 0.0020 mmol, 1.0 mol%), CuSCN (4.9 mg, 0.040 mmol, 20 mol%), dmp (8.3 mg, 0.040 mmol, 20 mol%), and TMSNCS (56 µL, 0.40 mmol, 2.0 equiv.) in acetone (2 mL). Purification by flash column chromatography (Biotage, Sfär Silica 10 g, hexane/acetone = 100:0 to 80:20) gave **7h** (29 mg, 77%) as a colourless oil.

NMR Spectroscopy (*see spectra*):

^{1}**H NMR** (400 MHz, CDCl$_3$): δ_H 7.16 (d, *J* = 5.1 Hz, 1H), 6.96 – 6.92 (m, 1H), 6.83 (d, *J* = 3.4 Hz, 1H), 3.03 (t, *J* = 7.1 Hz, 2H), 2.96 (t, *J* = 6.4 Hz, 2H), 2.26 – 2.15 (m, 2H) ppm;

^{13}**C NMR** (100 MHz, CDCl$_3$): δ_C 142.3, 127.1, 125.3, 124.0, 112.1, 32.9, 31.5, 27.8 ppm.

IR (film): ν_{max} 2945, 2155, 1439, 1250, 895, 823, 701cm^{-1}.

HRMS (APCI-MS$^+$): m/z calc'd for $C_8H_9NS_2$ [M+H]$^+$ 184.0429; found 184.0244.

1,2-Dimethoxy-4-(2-thiocyanatoethyl)benzene (7j)

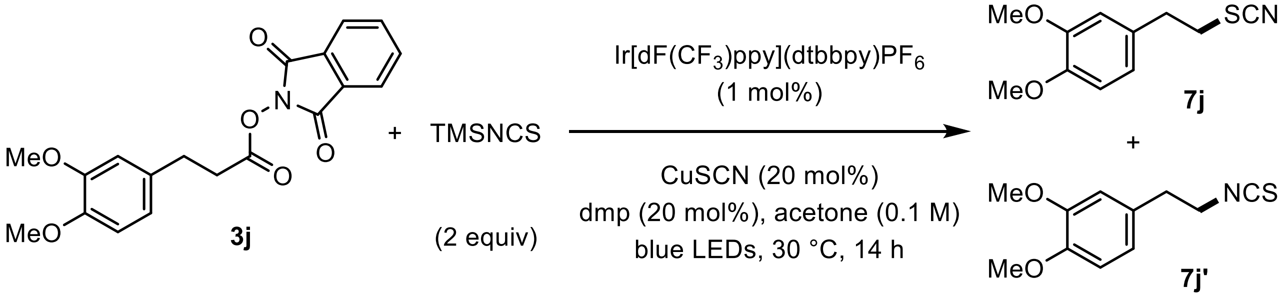

Prepared following **GP4**, using redox active ester **3j** (36 mg, 0.10 mmol), Ir[dF(CF$_3$)ppy)$_2$(dtbbpy)PF$_6$ (1.1 mg, 0.0010 mmol, 1.0 mol%), CuSCN (2.4 mg, 0.020 mmol, 20 mol%), dmp (4.2 mg, 0.020 mmol, 20 mol%), and TMSNCS (28 µL, 0.20 mmol, 2.0 equiv.) in acetone (1 mL). Purification by flash column chromatography (Biotage, Sfär Silica 10 g, pentane/acetone = 100:0 to 80:20) gave **7j** (13.5 mg, 60%) as a colourless oil, and **7j'** (2.5 mg, 11%) as a colourless oil.

For **7j**:

NMR Spectroscopy (*see spectra*):

^{1}**H NMR** (500 MHz, CDCl$_3$): δ_H 6.83 (d, *J* = 8.1 Hz, 1H), 6.76 (dd, *J* = 8.2, 2.0 Hz, 1H), 6.72 (d, *J* = 2.0 Hz, 1H), 3.88 (s, 3H), 3.87 (s, 3H), 3.20 – 3.12 (m, 2H), 3.06 (dd, *J* = 8.2, 6.2 Hz, 2H) ppm;

^{13}C NMR (125 MHz, $CDCl_3$): δ_C 149.3, 148.4, 130.3, 120.9, 112.3, 112.0, 111.6, 56.1 (2C), 35.9, 35.5 ppm.

IR (film): ν_{max} 3000, 2936, 2835, 2152, 1606, 1591, 1514, 1464, 1452, 1260, 1239, 1147, 1026, 808, 765 cm^{-1}.

HRMS (ESI$^+$): m/z calc'd for $C_{11}H_{13}NO_2SNa$ $[M+Na]^+$ 246.0559; found 246.0569.

For **7j'**:

NMR Spectroscopy (*see spectra*):

^{1}H NMR (500 MHz, $CDCl_3$): δ_H 6.84 (d, J = 8.0 Hz, 1H), 6.79 – 6.70 (m, 2H), 3.90 (s, 3H), 3.88 (s, 3H), 3.70 (t, J = 6.8 Hz, 2H), 2.93 (t, J = 6.8 Hz, 2H) ppm;

^{13}C NMR (125 MHz, $CDCl_3$): δ_C 149.1, 148.2, 130.7, 129.6, 120.8, 112.1, 111.5, 56.0, 55.9, 46.7, 36.2 ppm.

IR (film): ν_{max} 2998, 2955, 2933, 2834, 2180, 2097, 1516, 1464, 1263, 1238, 1158, 1142, 1028 cm^{-1}.

HRMS (ESI$^+$): m/z calc'd for $C_{11}H_{13}NO_2SNa$ $[M+Na]^+$ 246.0559; found 246.0571.

1-Thiocyanatoheptadecane (7k)

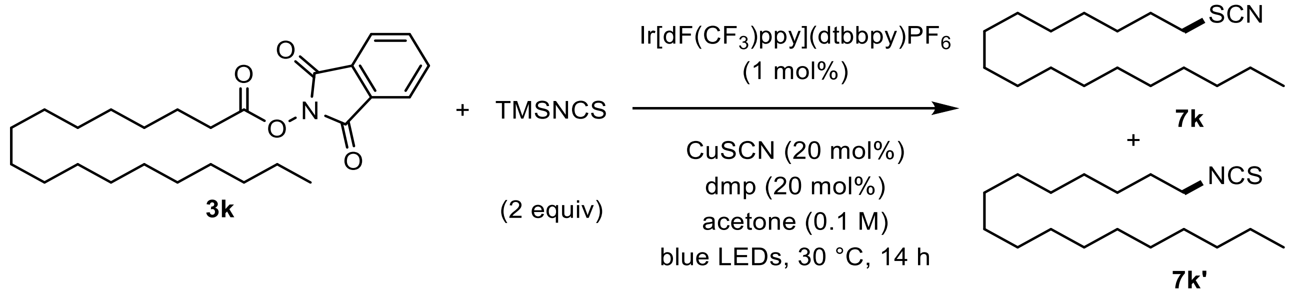

Prepared following **GP4**, using redox active ester **3k** (43 mg, 0.10 mmol), Ir[dF(CF_3)ppy)$_2$(dtbbpy)PF_6 (1.1 mg, 0.0010 mmol, 1.0 mol%), CuSCN (2.4 mg, 0.020 mmol, 20 mol%), dmp (4.2 mg, 0.020 mmol, 20 mol%), and TMSNCS (28 µL, 0.20 mmol, 2.0 equiv.) in acetone (1 mL). Purification by flash column chromatography (Biotage, Sfär Silica 10 g, pentane/Et_2O = 100:0 to 95:5) gave **7k** (22 mg, 74%) as a colourless oil, and **7k'** (3 mg, 10%) as a colourless oil.

For **7k**:

NMR Spectroscopy (*see spectra*):

^{1}H NMR (500 MHz, $CDCl_3$): δ_H 2.94 (t, J = 7.3 Hz, 2H), 1.82 (p, J = 7.4 Hz, 2H), 1.43 (p, J = 7.0 Hz, 2H), 1.34 – 1.21 (m, 26H), 0.88 (t, J = 6.8 Hz, 3H) ppm;

^{13}C NMR (125 MHz, $CDCl_3$): δ_C 112.5, 34.2, 32.1, 30.0, 29.83 (3C), 29.81 (2C), 29.88, 29.7, 29.6, 29.5 (2C), 29.0, 28.1, 22.8, 14.3 ppm.

IR (film): ν_{max} 2921, 2852, 2155, 1466, 1377, 721 cm^{-1}.

HRMS (ESI$^+$): m/z calc'd for $C_{18}H_{35}NSNa$ $[M+Na]^+$ 320.2382; found 320.2394.

For **7k'**:

NMR Spectroscopy (see spectra):

^{1}H NMR (500 MHz, $CDCl_3$): δ_H 3.50 (t, J = 6.6 Hz, 2H), 1.69 (dq, J = 8.5, 6.8 Hz, 2H), 1.45 – 1.37 (m, 2H), 1.35 – 1.21 (m, 26H), 0.88 (t, J = 6.9 Hz, 3H) ppm;

^{13}C NMR (125 MHz, $CDCl_3$): δ_C 129.5, 45.1, 31.9, 30.0, 29.67 (3C), 29.65, 29.64, 29.63, 29.58, 29.5, 29.38, 29.35, 28.8, 26.6, 22.7, 14.1 ppm.

IR (film): ν_{max} 2922, 2853, 2180, 2090, 1465, 1346, 750 cm^{-1}.

HRMS (EI$^+$): m/z calc'd for $C_{18}H_{35}NS$ [M-H]$^+$ 296.2406; found 296.2407.

Methyl (1*R*,3*R*)-1,2,2-trimethyl-3-thiocyanatocyclopentane-1-carboxylate (7l)

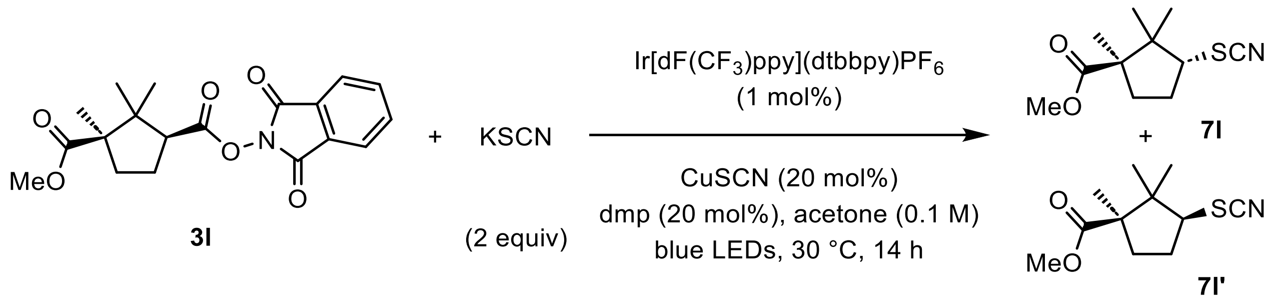

Prepared following **GP4**, using redox active ester **3l** (55 mg, 0.20 mmol), Ir[dF(CF_3)ppy)$_2$(dtbbpy)PF_6 (2.2 mg, 0.0020 mmol, 1.0 mol%), CuSCN (4.9 mg, 0.040 mmol, 20 mol%), dmp (8.3 mg, 0.040 mmol, 20 mol%), and KSCN (39 mg, 0.40 mmol, 2.0 equiv.) in acetone (2 mL). Purification by flash column chromatography (Biotage, Sfär Silica 10 g, hexane/EtOAc = 100:0 to 90:10) gave **7l** (22 mg, 48%) as a colourless oil, and **7l'** (7 mg, 15%) as a colourless oil.

For **7l**:

NMR Spectroscopy (see spectra):

^{1}H NMR (500 MHz, $CDCl_3$): δ_H 3.73 (dd, J = 10.0, 9.0 Hz, 1H), 3.67 (s, 3H), 2.46 (dddd, J = 13.7, 9.8, 9.0, 4.9 Hz, 1H), 2.24 (ddd, J = 13.6, 9.9, 5.4 Hz, 1H), 1.87 (dddd, J = 13.5, 11.7, 10.1, 5.4 Hz, 1H), 1.63 (ddd, J = 13.6, 11.8, 4.9 Hz, 1H), 1.19 (s, 3H), 0.97 (s, 3H), 0.96 (s, 3H) ppm;

^{13}C NMR (125 MHz, $CDCl_3$): δ_C 177.1, 112.4, 57.0, 55.1, 51.8, 48.2, 33.4, 29.7, 22.5, 19.8, 19.4 ppm.

IR (film): ν_{max} 2973, 2965, 2877, 2153, 1719, 1461, 1278, 1201, 1121, 1096, 750 cm^{-1}.

HRMS (ESI$^+$): m/z calc'd for $C_{11}H_{17}NO_2SNa$ [M+Na]$^+$ 250.0872; found 250.0881.

For **7l'**:

NMR Spectroscopy (see spectra):

^{1}H NMR (500 MHz, $CDCl_3$): δ_H 3.70 (s, 3H), 3.52 (t, J = 9.7 Hz, 1H), 2.66 (td, J = 13.1, 5.5 Hz, 1H), 2.34 (dtd, J = 14.8, 9.4, 5.5 Hz, 1H), 1.89 (dddd, J = 14.5, 12.0, 10.1, 5.2 Hz, 1H), 1.59 – 1.54 (m, 1H), 1.22 (s, 3H), 1.21 (s, 3H), 0.89 (s, 3H) ppm;

^{13}C NMR (125 MHz, CDCl$_3$): δ$_C$ 175.7, 112.5, 57.8, 54.6, 52.0, 47.5, 31.9, 28.6, 22.2, 21.7, 20.3 ppm.

IR (film): v_{max} 2969, 2951, 2881, 2846, 2153, 1728, 1460, 1380, 1275, 1260, 1208, 1156, 1112, 750 cm^{-1}.

HRMS (ESI$^+$): m/z calc'd for $C_{11}H_{17}NO_2SNa$ [M+Na]$^+$ 250.0872; found 250.0878.

(3*R*,5*R*,8*R*,9*S*,10*S*,13*R*,14*S*,17*R*)-10,13-Dimethyl-17-((*R*)-4-thiocyanatobutan-2-yl)hexadecahydro-1*H*-cyclopenta[*a*]phenanthren-3-ol (7m)

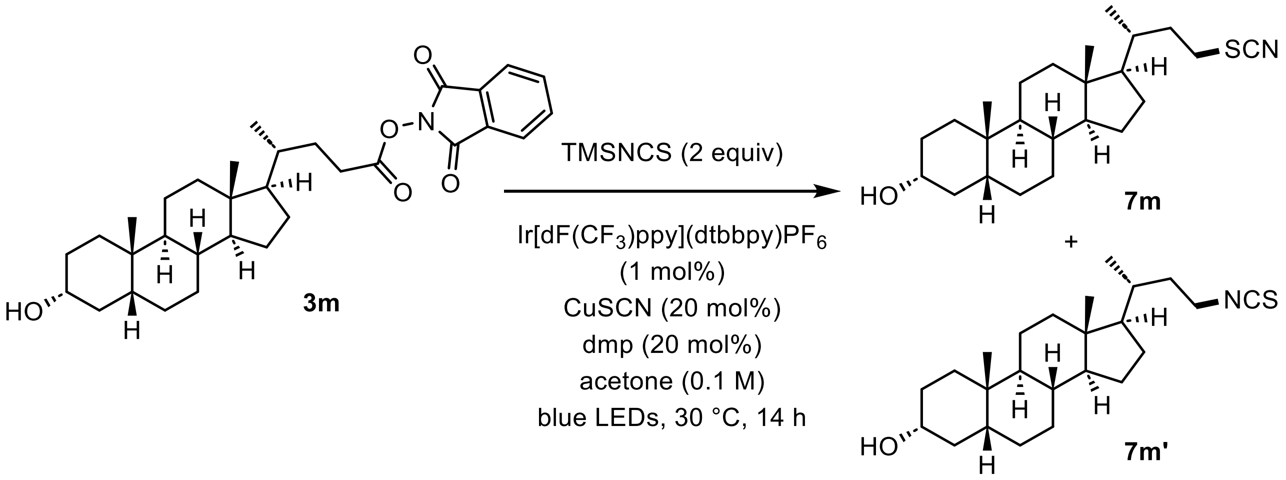

Prepared following **GP4**, using redox active ester **3m** (52 mg, 0.10 mmol), Ir[dF(CF_3)ppy)$_2$(dtbbpy)PF_6 (1.1 mg, 0.0010 mmol, 1.0 mol%), CuSCN (2.4 mg, 0.020 mmol, 20 mol%), dmp (4.2 mg, 0.020 mmol, 20 mol%), and TMSNCS (28 µL, 0.20 mmol, 2.0 equiv.) in acetone (1 mL). Purification by flash column chromatography (Biotage, Sfär Silica 10 g, pentane/acetone = 100:0 to 85:15) gave **7m** (23 mg, 59%) as a colourless oil, and **7m'** (7.5 mg, 19%) as a colourless oil.

For **7m**:

NMR Spectroscopy (*see spectra*):

^{1}**H NMR** (500 MHz, CDCl$_3$): δ$_H$ 3.62 (tt, *J* = 10.6, 4.7 Hz, 1H), 3.06 (ddd, *J* = 13.3, 9.9, 3.8 Hz, 1H), 2.83 (dt, *J* = 12.4, 8.2 Hz, 1H), 1.97 – 1.71 (m, 6H), 1.69 – 1.62 (m, 1H), 1.62 – 1.56 (m, 2H), 1.54 – 1.47 (m, 2H), 1.44 – 1.35 (m, 5H), 1.34 – 1.20 (m, 4H), 1.20 – 1.03 (m, 5H), 1.00 – 0.93 (m, 1H), 0.95 (d, *J* = 6.0 Hz, 3H), 0.91 (s, 3H), 0.66 (s, 3H) ppm;

^{13}**C NMR** (125 MHz, CDCl$_3$): δ$_C$ 112.6, 72.0, 56.6, 55.9, 43.0, 42.2, 40.5, 40.3, 36.6, 36.4, 36.0, 35.5, 35.1, 34.7, 31.8, 30.7, 28.4, 27.3, 26.5, 24.3, 23.5, 20.9, 18.4, 12.2 ppm.

IR (film): v_{max} 3292 (br), 2936, 2928, 2863, 2853, 2152, 1466, 1383, 1364, 1070, 1042, 1014, 741 cm^{-1}.

HRMS (ESI$^+$): m/z calc'd for $C_{24}H_{39}NOSNa$ [M+Na]$^+$ 412.2645; found 412.2649.

For **7m'**:

NMR Spectroscopy (*see spectra*):

^{1}**H NMR** (500 MHz, CDCl$_3$): δ$_H$ 3.63 (tt, *J* = 10.5, 4.6 Hz, 1H), 3.58 – 3.45 (m, 2H), 1.96 (dt, *J* = 12.5, 3.2

Hz, 1H), 1.92 – 1.72 (m, 5H), 1.66 (d, *J* = 12.7 Hz, 1H), 1.62 – 1.57 (m, 1H), 1.54 – 1.48 (m, 2H), 1.44–1.36 (m, 6H), 1.35 – 1.26 (m, 3H), 1.24 – 1.03 (m, 6H), 0.99 (dd, *J* = 14.2, 3.4 Hz, 1H), 0.94 (d, *J* = 6.6 Hz, 3H), 0.92 (s, 3H), 0.66 (s, 3H) ppm;

^{13}C NMR (125 MHz, $CDCl_3$): δ_C 129.7, 72.0, 56.6, 56.2, 43.0, 43.0, 42.2, 40.6, 40.3, 36.6, 36.3, 36.0, 35.5, 34.7, 33.7, 30.7, 28.4, 27.3, 26.5, 24.3, 23.5, 21.0, 18.4, 12.2 ppm.

IR (film): ν_{max} 3334 (br), 2929, 2863, 2180, 2092, 1448, 1376, 1068, 1033, 750 cm^{-1}.

HRMS (ESI$^+$): m/z calc'd for $C_{24}H_{39}NOSNa$ [M+Na]$^+$ 412.2645, found 412.2632.

Benzyl *N*-(*tert*-butoxycarbonyl)-*S*-cyano-*D*-homocysteinate (7n)

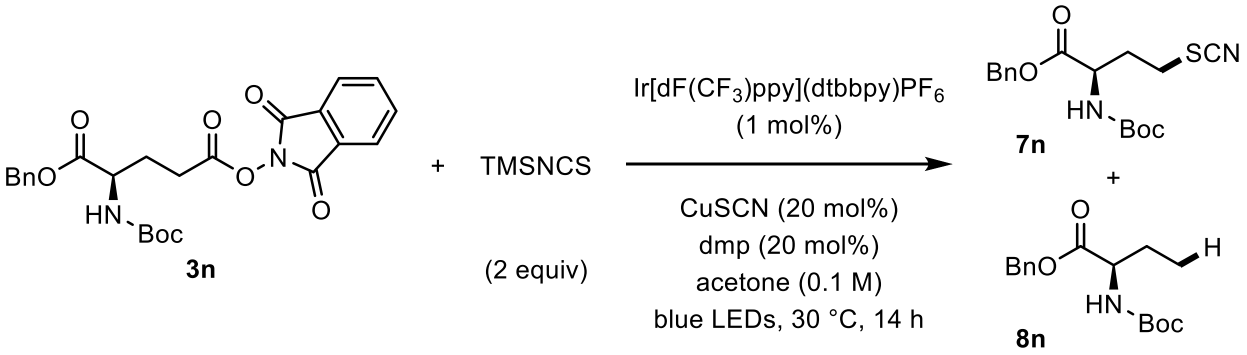

Prepared following **GP4**, using redox active ester **3n** (72 mg, 0.15 mmol), Ir[dF(CF_3)ppy$)_2$(dtbbpy)PF_6 (1.7 mg, 0.0015 mmol, 1.0 mol%), CuSCN (3.6 mg, 0.030 mmol, 20 mol%), dmp (6.2 mg, 0.020 mmol, 20 mol%), and TMSNCS (42 µL, 0.30 mmol, 2.0 equiv.) in acetone (1.5 mL). Purification by flash column chromatography (Biotage, Sfär Silica 10 g, hexane/EtOAc = 100:0 to 60:40) gave **7n** (23 mg, 44%) as a colourless oil, and **8n** (4 mg, 9%) as a colourless oil.

For **7n**:

NMR Spectroscopy (*see spectra*):

^{1}H NMR (500 MHz, $CDCl_3$): δ_H 7.44 – 7.31 (m, 5H), 5.25 – 5.15 (m, 3H), 4.55 – 4.39 (m, 1H), 2.96 (t, *J* = 7.5 Hz, 2H), 2.44 – 2.34 (m, 1H), 2.17 – 2.07 (m, 1H), 1.43 (s, 9H) ppm;

^{13}C NMR (125 MHz, $CDCl_3$): δ_C 171.3, 155.6, 135.0, 128.9 (2C), 128.6, 112.0, 80.7, 67.9, 52.2, 33.8, 30.4, 28.4 ppm.

IR (film): ν_{max} 3363 (br), 3069, 3034, 3006, 2978, 2932, 2155, 1709, 1499, 1455, 1367, 1254, 1161, 1052, 1024, 750, 698 cm^{-1}.

HRMS (ESI$^+$): m/z calc'd for $C_{17}H_{22}N_2O_4SNa$ [M+Na]$^+$ 373.1192; found 373.1178.

For **8n**:

NMR Spectroscopy (*see spectra*):

^{1}H NMR (500 MHz, $CDCl_3$): δ_H 7.39 – 7.31 (m, 5H), 5.21 (d, *J* = 12.5 Hz, 1H), 5.14 (d, *J* = 12.4 Hz, 1H),

5.09 – 4.60 (br m, 1H), 4.38 – 4.02 (br m, 1H), 1.91 – 1.80 (m, 1H), 1.73 – 1.63 (m, 1H), 1.44 (s, 9H), 0.90 (t, *J* = 7.4 Hz, 3H) ppm;

^{13}C NMR (125 MHz, $CDCl_3$): δ_C 172.8, 155.5, 135.6, 128.7, 128.5, 128.4, 80.0, 67.1, 54.8, 28.5, 26.1, 9.7 ppm.

IR (film): ν_{max} 3370 (br), 3065, 3034, 3006, 2975, 2932, 2877, 1742, 1715, 1499, 1366, 1275, 1260, 1161, 750, 698 cm^{-1}.

HRMS (ESI$^+$): m/z calc'd for $C_{16}H_{23}NO_4Na$ [M+Na]$^+$ 316.1519; found 316.1532.

(1*S*,2*S*,4a*R*,4b*R*,7*S*,9a*S*,10*S*,10a*R*)-2,7-Dihydroxy-1-methyl-8-methylene-10-thiocyanato-1,2,4b,5,6,7,8,9,10,10a-decahydro-4a,1-(epoxymethano)-7,9a-methanobenzo[*a*]azulen-13-one (7o)

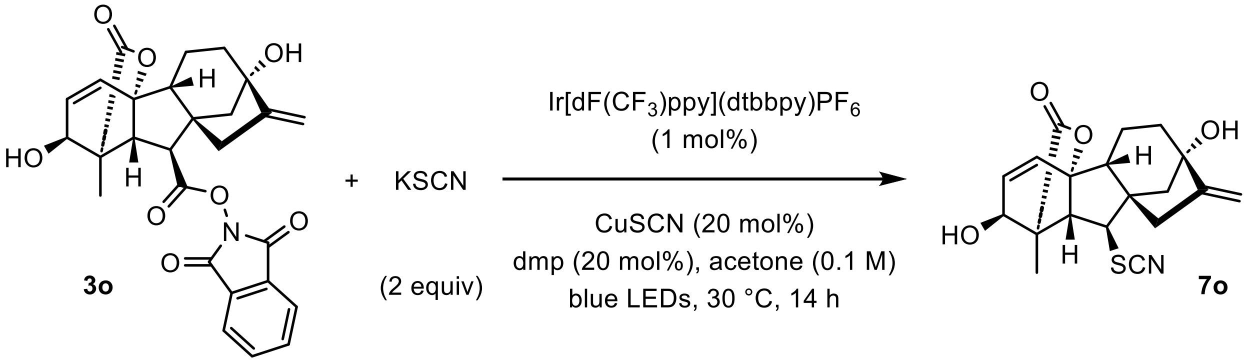

Prepared following **GP4**, using redox active ester **3o** (49 mg, 0.10 mmol), Ir[dF(CF_3)ppy]$_2$(dtbbpy)PF_6 (1.1 mg, 0.0010 mmol, 1.0 mol%), CuSCN (2.4 mg, 0.020 mmol, 20 mol%), dmp (4.2 mg, 0.020 mmol, 20 mol%), and KSCN (19 mg, 0.20 mmol, 2.0 equiv.) in acetone (1 mL). Purification by flash column chromatography (Biotage, Sfär Silica 10 g, hexane/acetone = 100:0 to 60:40) gave **7o** (23 mg, 64%) as a white solid. The d.r. was determined to be >95:5 by ^{1}H NMR analysis of the purified product.

NMR Spectroscopy (*see spectra*):

^{1}H NMR (500 MHz, CD_3CN): δ_H 6.30 (d, *J* = 9.3 Hz, 1H), 5.83 (dd, *J* = 9.3, 3.7 Hz, 1H), 5.16 (d, *J* = 2.4 Hz, 1H), 4.97 (s, 1H), 4.03 (dd, *J* = 6.5, 3.8 Hz, 1H), 3.69 (d, *J* = 6.4 Hz, 1H), 3.41 (d, *J* = 10.6 Hz, 1H), 2.97 (s, 1H), 2.89 (d, *J* = 10.5 Hz, 1H), 2.78 (dt, *J* = 15.5, 3.1 Hz, 1H), 2.40 (dd, *J* = 16.1, 2.9 Hz, 1H), 2.04 (dd, *J* = 10.6, 6.1 Hz, 1H), 1.91 – 1.95 (m, 1H), 1.82 – 1.73 (m, 2H), 1.72 – 1.64 (m, 2H), 1.59 (d, *J* = 10.8 Hz, 1H), 1.35 (s, 3H) ppm;

^{13}C NMR (125 MHz, CD_3CN): δ_C 178.8, 157.9, 133.8, 132.7, 112.5, 107.3, 90.1, 78.4, 70.6, 56.6, 54.4, 52.8, 52.6, 50.6, 44.9, 44.1, 39.4, 17.8, 15.3 ppm.

IR (film): ν_{max} 3410 (br), 3076, 3041, 2975, 2937, 2877, 2154, 1768, 1705, 1452, 1380, 1275, 1258, 1159, 1110, 1044, 889, 750 cm^{-1}.

HRMS (ESI$^+$): m/z calc'd for $C_{19}H_{21}NO_4SNa$ [M+Na]$^+$ 382.1083; found 382.1092.

(1*S*,2*S*,4a*R*,4b*R*,7*S*,9a*S*,10*S*,10a*R*)-1-Methyl-8-methylene-13-oxo-10-thiocyanato-1,2,5,6,8,9,10,10a-octahydro-4a,1-(epoxymethano)-7,9a-methanobenzo[*a*]azulene-2,7(4b*H*)-diyl diacetate (7p)

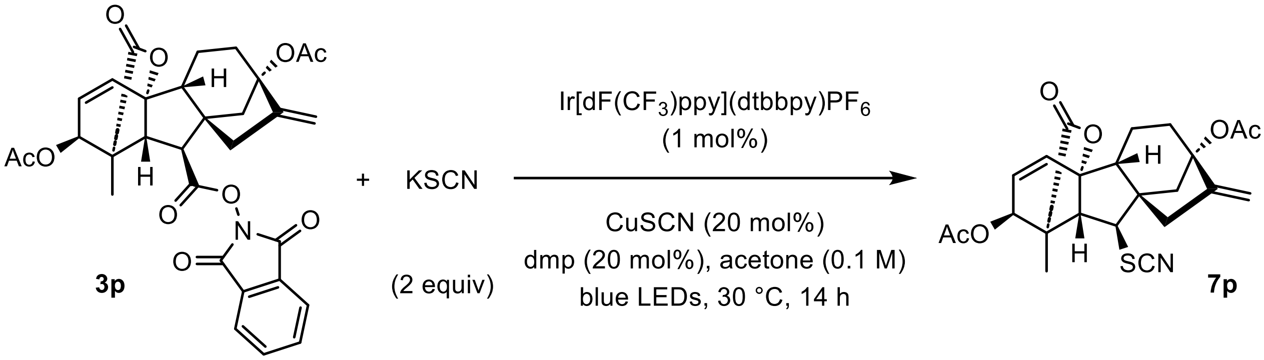

3p **7p**

Prepared following **GP4**, using redox active ester **3p** (58 mg, 0.10 mmol), Ir[dF(CF_3)ppy)$_2$(dtbbpy)PF_6 (1.1 mg, 0.0010 mmol, 1.0 mol%), CuSCN (2.4 mg, 0.020 mmol, 20 mol%), dmp (4.2 mg, 0.020 mmol, 20 mol%), and KSCN (19 mg, 0.20 mmol, 2.0 equiv.) in acetone (1 mL). Purification by flash column chromatography (Biotage, Sfär Silica 10 g, hexane/acetone = 100:0 to 60:40) gave **7p** (29 mg, 65%) as a white solid. The d.r. was determined to be >95:5 by ^{1}H NMR analysis of the purified product.

NMR Spectroscopy (*see spectra*):

^{1}H NMR (500 MHz, $CDCl_3$): δ_H 6.37 (dd, *J* = 9.3, 0.8 Hz, 1H), 5.91 (dd, *J* = 9.3, 3.8 Hz, 1H), 5.36 (dd, *J* = 3.9, 0.8 Hz, 1H), 5.21 (dd, *J* = 3.3, 1.5 Hz, 1H), 5.10 (dd, *J* = 2.8, 1.3 Hz, 1H), 3.15 (d, *J* = 10.8 Hz, 1H), 3.12 (d, *J* = 10.9 Hz, 1H), 2.94 (dt, *J* = 15.0, 3.0 Hz, 1H), 2.41 – 2.35 (m, 2H), 2.22 – 2.19 (m, 2H), 2.14 (s, 3H), 2.03 (s, 3H), 2.02 – 1.96 (m, 2H), 1.84 – 1.72 (m, 2H), 1.41 (s, 3H) ppm;

^{13}C NMR (125 MHz, $CDCl_3$): δ_C 176.1, 170.02, 169.98, 152.4, 134.0, 129.5, 110.5, 108.8, 88.6, 83.9, 70.7, 56.4, 52.9, 52.4, 51.6, 50.1, 43.2, 39.5, 36.2, 22.1, 20.9, 17.1, 14.9 ppm.

IR (film): ν_{max} 3080, 3049, 2979, 2939, 2877, 2154, 1781, 1737, 1370, 1258, 1227, 1157, 1087, 1019 cm^{-1}.

HRMS (ESI$^+$): m/z calc'd for $C_{23}H_{25}NO_6SNa$ [M+Na]$^+$ 466.1295; found 466.1301.

4-Thiocyanatotetrahydro-2*H*-pyran (7q)

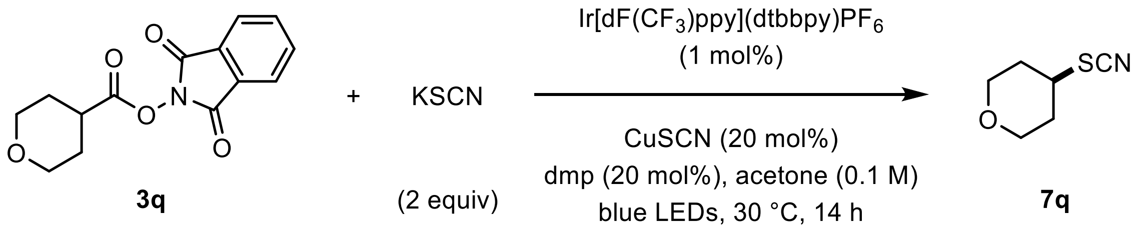

3q **7q**

Prepared following **GP4**, using redox active ester **3q** (55 mg, 0.20 mmol), Ir[dF(CF_3)ppy)$_2$(dtbbpy)PF_6 (2.2 mg, 0.0020 mmol, 1.0 mol%), CuSCN (4.9 mg, 0.040 mmol, 20 mol%), dmp (8.3 mg, 0.040 mmol, 20 mol%), and KSCN (39 mg, 0.40 mmol, 2.0 equiv.) in acetone (2 mL). Purification by flash column chromatography (Biotage, Sfär Silica 10 g, hexane/acetone = 100:0 to 80:20) gave **7q** (18 mg, 63%) as a colourless oil.

NMR Spectroscopy (*see spectra*):

^{1}H NMR (500 MHz, $CDCl_3$): δ_H 4.02 (dt, *J* = 12.0, 3.9 Hz, 2H), 3.53 – 3.36 (m, 3H), 2.11 – 2.02 (m, 2H), 1.93 – 1.84 (m, 2H) ppm;

^{13}C NMR (125 MHz, $CDCl_3$): δ_C 110.7, 67.1, 44.4, 33.4 ppm.

IR (film): ν_{max} 2956, 2928, 2850, 2152, 1445, 1386, 1302, 1239, 1136, 1085, 1004, 828 cm^{-1}.

HRMS (ESI^+): m/z calc'd for C_6H_9NOSNa $[M+Na]^+$ 166.0297; found 166.0305.

2.8. Unsuccessful Decarboxylative Functionalisations

2.8.1. Fluorination

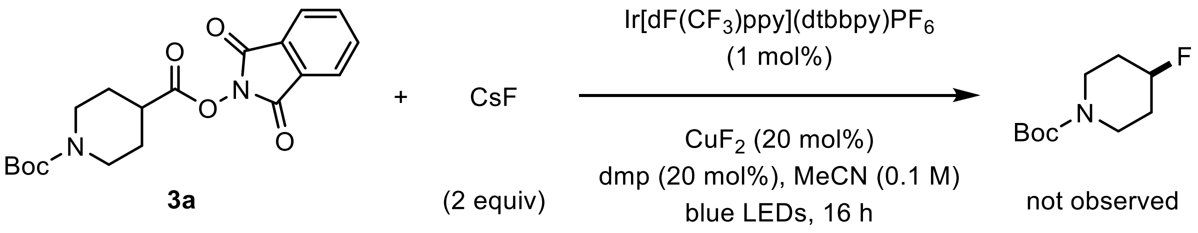

An oven-dried vial (7 mL) was charged with redox active ester **3a** (37 mg, 0.10 mmol), Ir[dF(CF_3)ppy]$_2$(dtbbpy)PF_6 (1.1 mg, 0.0010 mmol, 1.0 mol%), CuF_2 (2.0 mg, 0.020 mmol, 20 mol%), 2,9-dimethyl-1,10-phenanthroline (dmp, 4.2 mg, 0.020 mmol, 20 mol%), and CsF (30 mg, 0.20 mmol, 2.0 equiv.). The vial was sealed with a septum and placed under a N_2 atmosphere before adding anhydrous MeCN (1.0 mL). The mixture was subsequently degassed by sparging with N_2 for 1 min. The reaction was stirred under blue LED irradiation in the photoreactor for 16 h. The reaction was concentrated under reduced pressure before analysis by ^{1}H NMR and GC-MS. No fluorination product was observed.

The reaction was also performed with 2 equivalents CuF_2 and no CsF, however, no fluorination product was observed.

2.8.2. Azidation

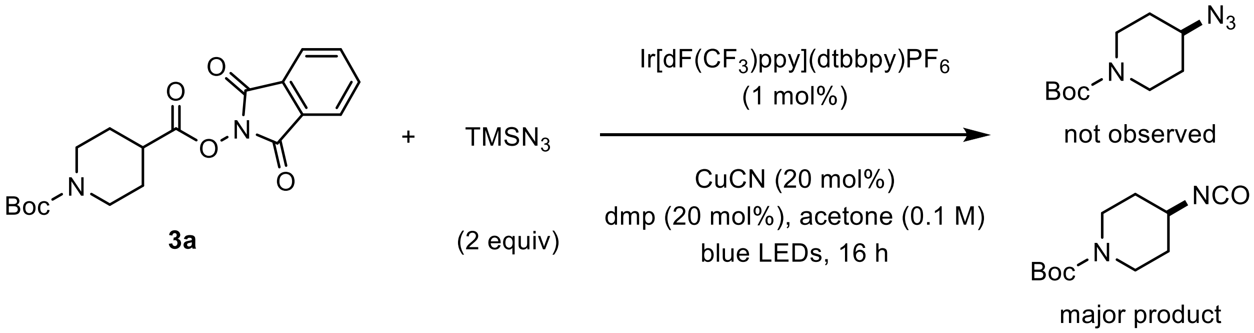

An oven-dried vial (7 mL) was charged with redox active ester **3a** (37 mg, 0.10 mmol), Ir[dF(CF_3)ppy]$_2$(dtbbpy)PF_6 (1.1 mg, 0.0010 mmol, 1.0 mol%), CuCN (1.8 mg, 0.020 mmol, 20 mol%), 2,9-dimethyl-1,10-phenanthroline (dmp, 4.2 mg, 0.020 mmol, 20 mol%), and $TMSN_3$ (23 mg, 0.20 mmol, 2.0 equiv.). The vial was sealed with a septum and placed under a N_2 atmosphere before adding acetone (1.0 mL). The mixture was subsequently degassed by sparging with N_2 for 1 min. The reaction was stirred under blue LED irradiation in the photoreactor for 16 h. The reaction was concentrated under reduced pressure before analysis by ^{1}H NMR and GC-MS. No azide product was observed and the isocyanate product was the major product.

The isocyanate was also observed when the reaction was performed in the absence of photocatalyst or light, which indicates that the azide of $TMSN_3$ readily displaces the NHP group to form an acyl azide, which then undergoes a thermally promoted Curtius rearrangement.

3. MECHANISTIC STUDIES

3.1. Radical Clock Experiments

3.1.1. Decarboxylative Bromination

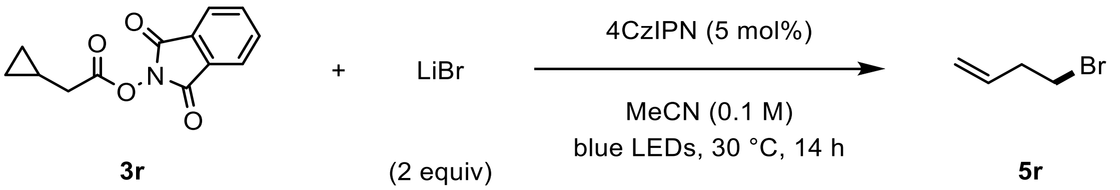

Prepared following a modified **GP2**, using redox active ester **3r** (25 mg, 0.10 mmol), 4CzIPN (3.9 mg, 0.0050 mmol, 5.0 mol%), and LiBr (18 mg, 0.20 mmol, 2.0 equiv.) in anhydrous CD_3CN (1 mL). After 14 h irradiation, 1,3,5-trimethoxybenzene (5.6 mg, 0.033 mmol) was added as an internal standard before 0.3 mL of the mixture was transferred to an NMR tube and diluted with CD_3CN (0.3 mL). ^1H NMR analysis showed the yield of **5r** was 66% (see spectrum below). Purification was not attempted due to the high volatility of **5r**.

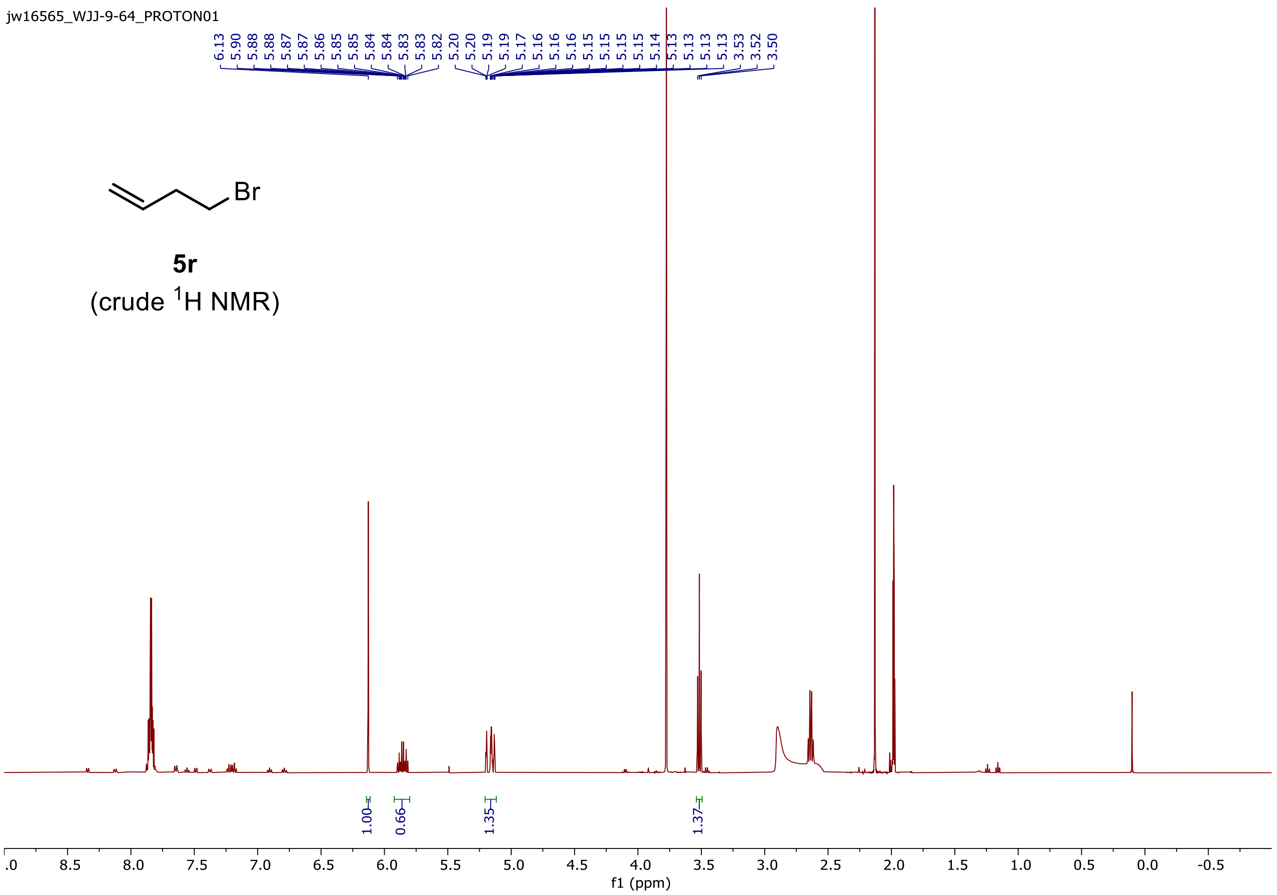

3.1.2. Decarboxylative Chlorination

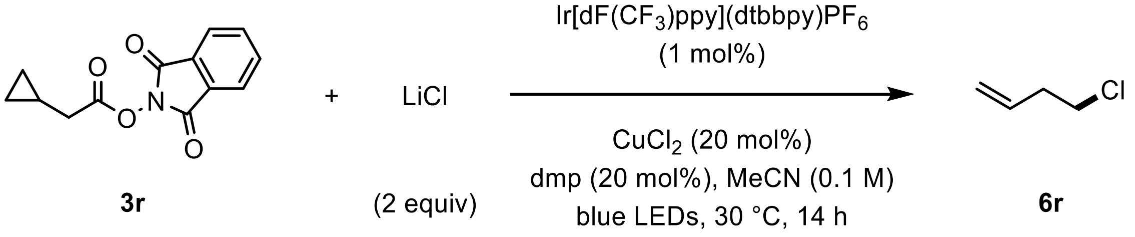

Prepared following a modified **GP3**, using redox active ester **3r** (25 mg, 0.10 mmol), Ir[dF(CF_3)ppy$)_2$(dtbbpy)PF_6 (1.2 mg, 0.0010 mmol, 1.0 mol%), $CuCl_2$ (2.7 mg, 0.020 mmol, 20 mol%), dmp (4.2 mg, 0.020 mmol, 20 mol%), and LiCl (8.5 mg, 0.20 mmol, 2.0 equiv.) in anhydrous CD_3CN (1 mL). After 14 h irradiation, 1,3,5-trimethoxybenzene (5.6 mg, 0.033 mmol) was added as an internal standard before 0.3 mL of the mixture was transferred to an NMR tube and diluted with CD_3CN (0.3 mL). ^{1}H NMR analysis showed the yield of **6r** was 24% (see spectrum below). Purification was not attempted due to the high volatility of **6r**.

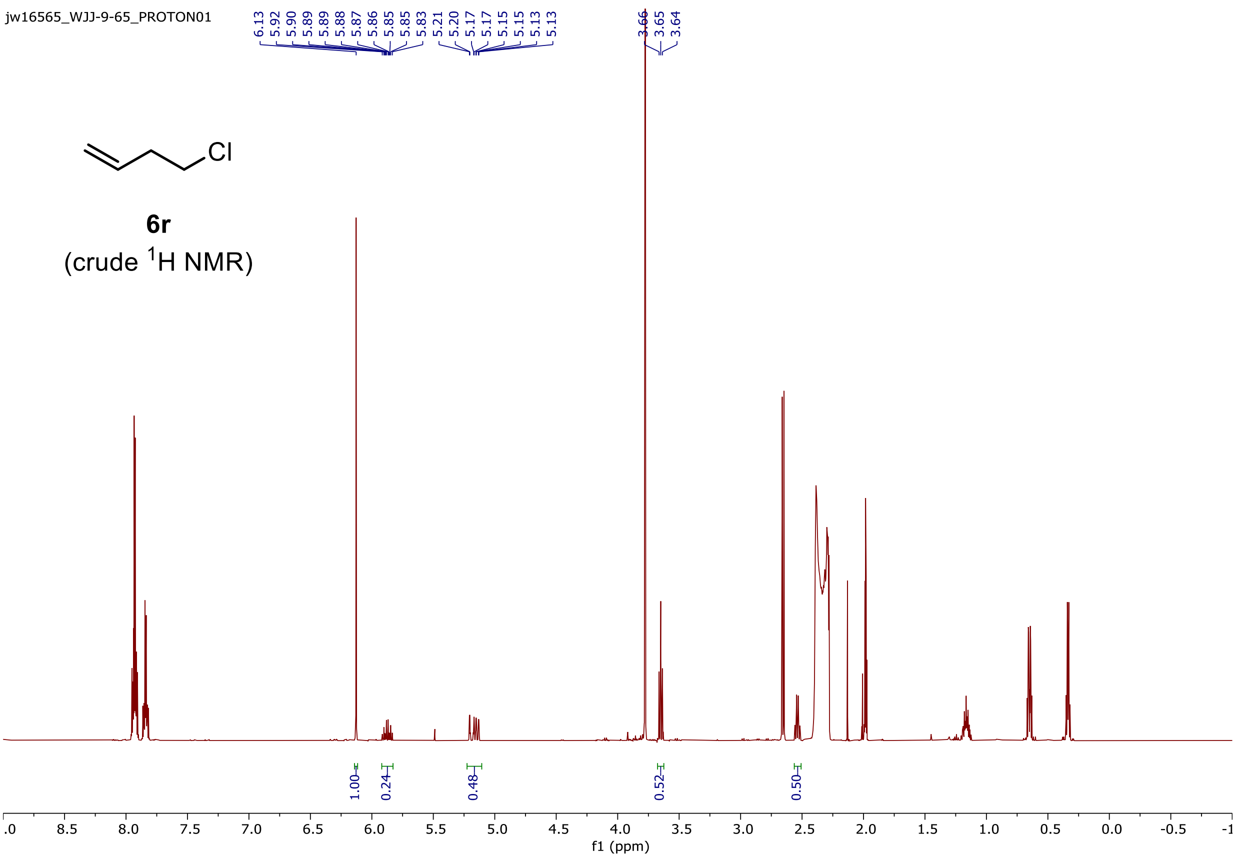

3.1.3. Decarboxylative Thiocyanation

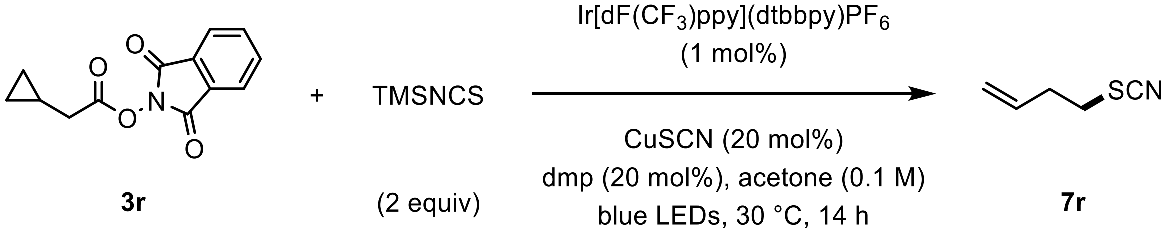

Prepared following **GP4**, using redox active ester **3r** (49 mg, 0.2 mmol), Ir[dF(CF_3)ppy$)_2$(dtbbpy)PF_6 (2.3 mg, 0.0020 mmol, 1.0 mol%), CuSCN (4.9 mg, 0.040 mmol, 20 mol%), dmp (8.3 mg, 0.040 mmol, 20 mol%), and TMSNCS (56 µL, 0.40 mmol, 2.0 equiv.) in acetone (2 mL). Purification by flash column chromatography (Biotage, Sfär Silica 10 g, hexane/Et_2O = 100:0 to 82:18) gave **7r** (12 mg, 53%) as a colourless oil.

NMR Spectroscopy (*see spectra*):

^{1}H NMR (500 MHz, $CDCl_3$): δ_H 5.79 (ddt, J = 17.0, 10.2, 6.7 Hz, 1H), 5.22 – 5.15 (m, 2H), 3.01 (t, J = 7.2 Hz, 2H), 2.57 (qt, J = 7.0, 1.3 Hz, 2H) ppm;

^{13}C NMR (125 MHz, $CDCl_3$): δ_C 133.8, 118.6, 112.2, 33.9, 33.4 ppm.

All recorded spectroscopic data matched those previously reported in the literature.[24]

3.2. Response to a Reviewer's Comment on C(sp^3)–H Bromination

During our decarboxylative bromination reactions, we did not observe any competing bromination of C(sp^3)–H bonds, despite the formation of bromine radicals during our proposed mechanism. We think that this is for the following reasons:

(1) The Br-radical is not a good hydrogen atom transfer (HAT) reagent for most C(sp^3)–H bonds. This is because the bond dissociation energy (BDE) of H–Br is around 88 kcal/mol, which is weaker than the majority of C(sp^3)-H bonds (e.g., BDE for non-activated alkyl C–H bonds = 96–99 kcal mol^{-1}; BDE for benzylic C–H bonds ~ 89 kcal mol^{-1}).[25] Therefore, HAT is thermodynamically disfavoured and unlikely to occur under our reaction conditions.

(2) If HAT from a C(sp^3)–H bond to a Br-radical did occur, it would generate H–Br and a transient alkyl radical. Since H–Br is not a brominating agent, and the active brominating agent (Br-radical or dibromide radical anion) is only generated in catalytic amounts, the transient alkyl radical is unlikely to have a long enough lifetime to be brominated.

(3) C(sp^3)–H bromination requires an oxidising brominating agent, whereas LiBr is a reductant. Therefore, productive bromination with LiBr would require a stoichiometric oxidant. Whilst the NHP-ester is the oxidant in our reaction, decarboxylative bromination likely outcompetes C(sp^3)–H bromination.

4. SPECTROSCOPIC DATA

^{1}H NMR (500 MHz, $CDCl_3$) of **3c** (*see procedure*)

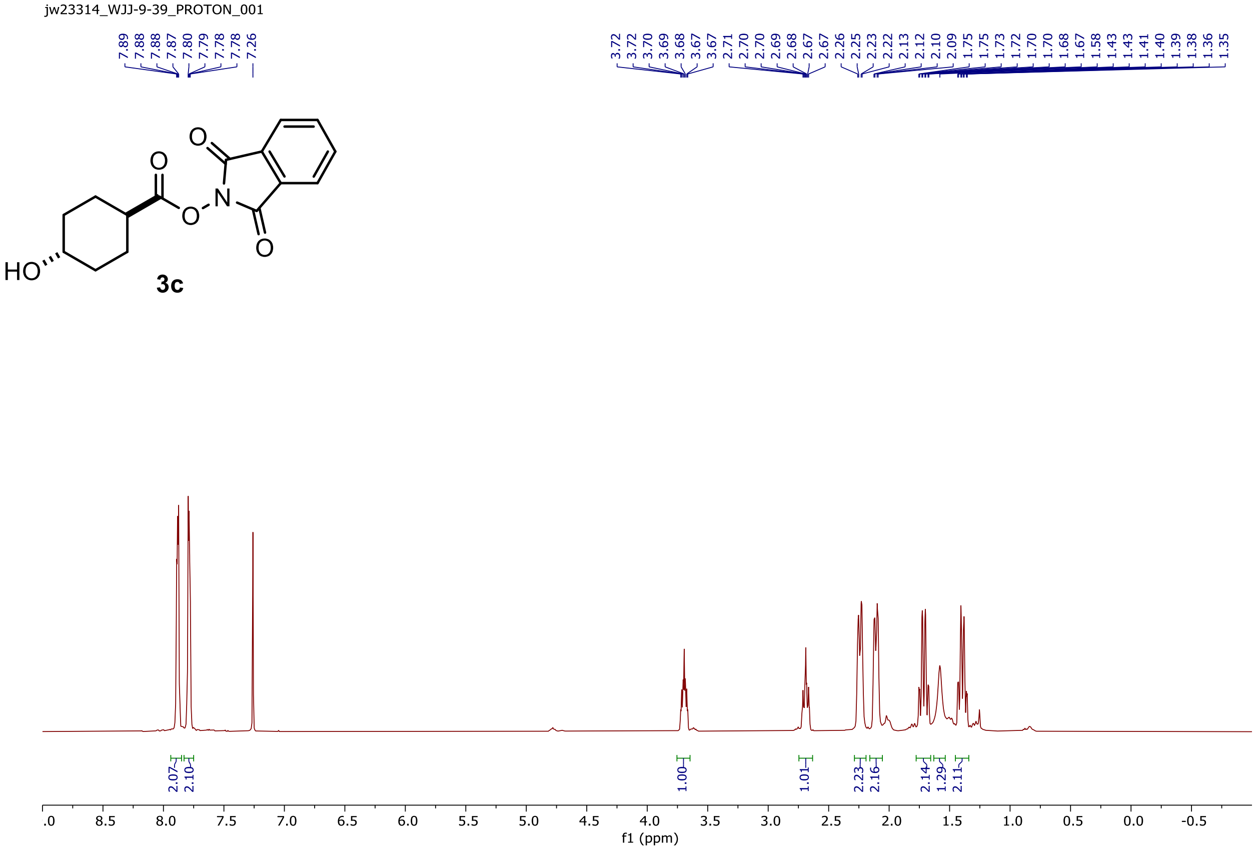

^{13}C NMR (125 MHz, $CDCl_3$) of **3c**

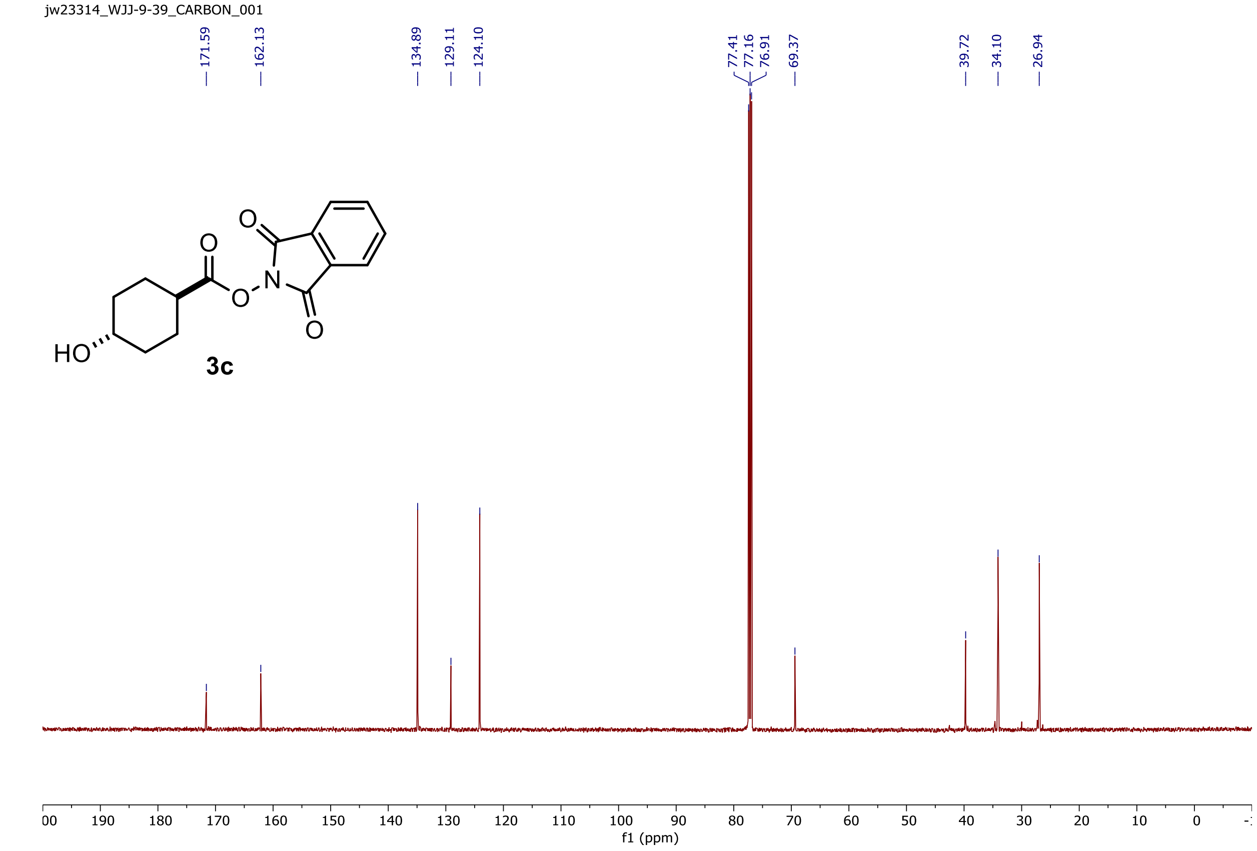

^{1}H NMR (400 MHz, $CDCl_3$) of **3h** (*see procedure*)

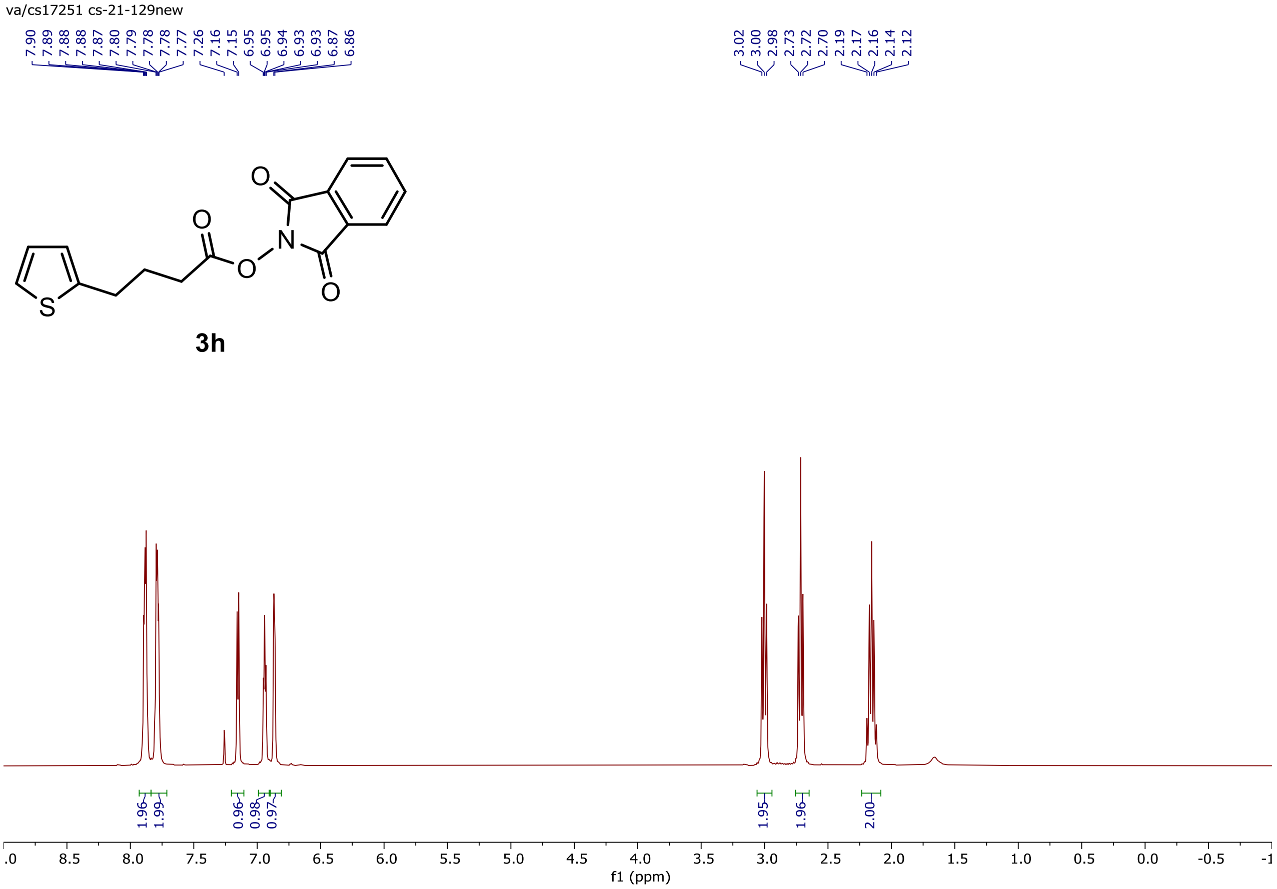

^{13}C NMR (100 MHz, $CDCl_3$) of **3h**

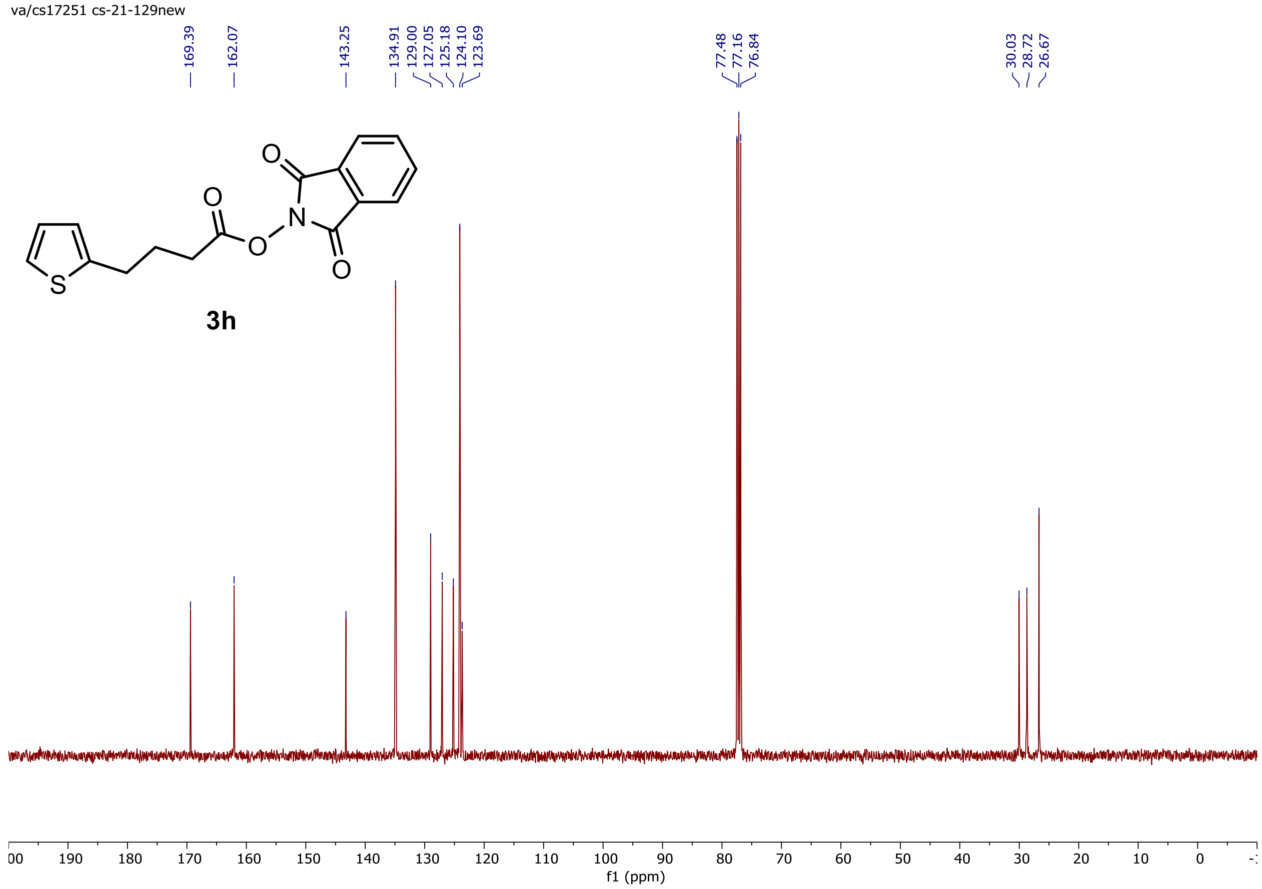

^{1}H NMR (400 MHz, $CDCl_3$) of **3i** (see procedure)

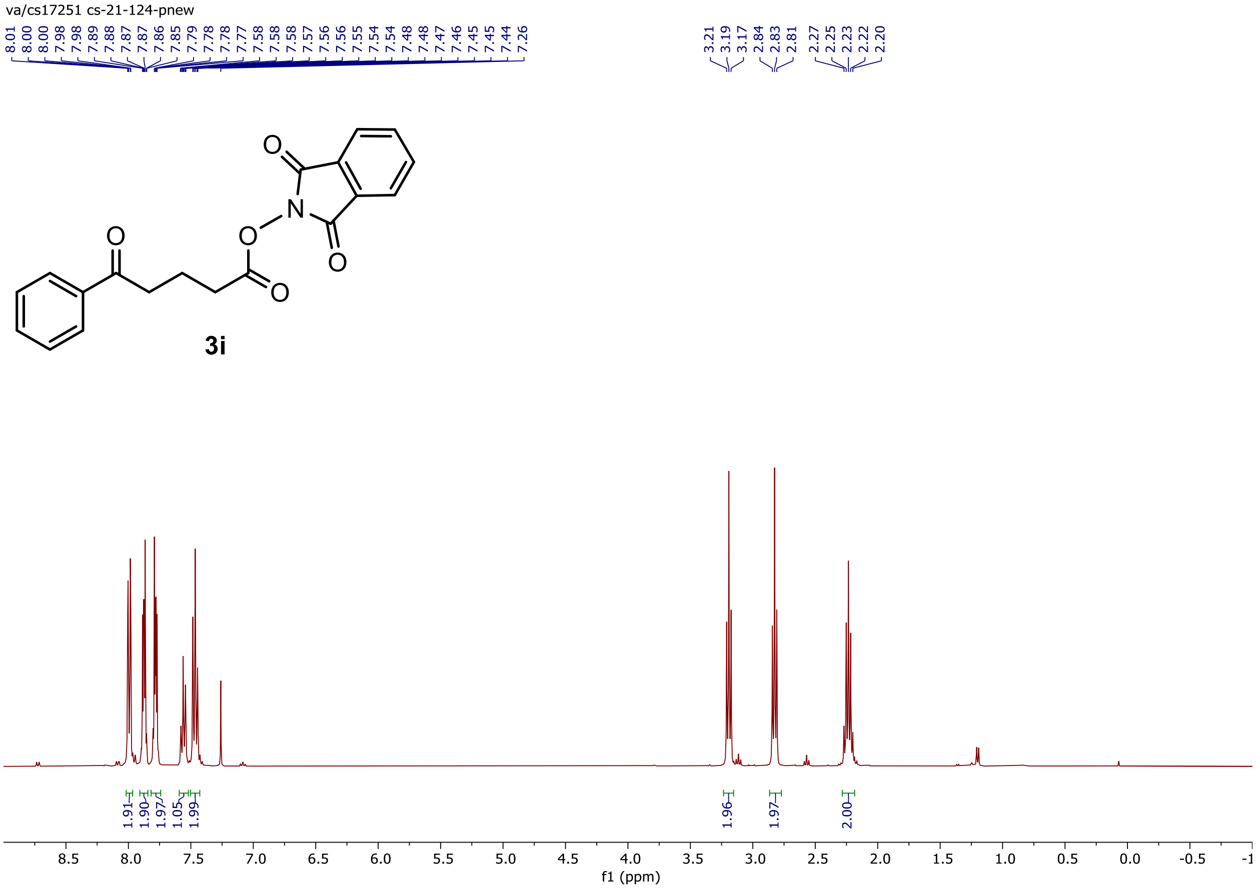

^{13}C NMR (100 MHz, $CDCl_3$) of **3i**

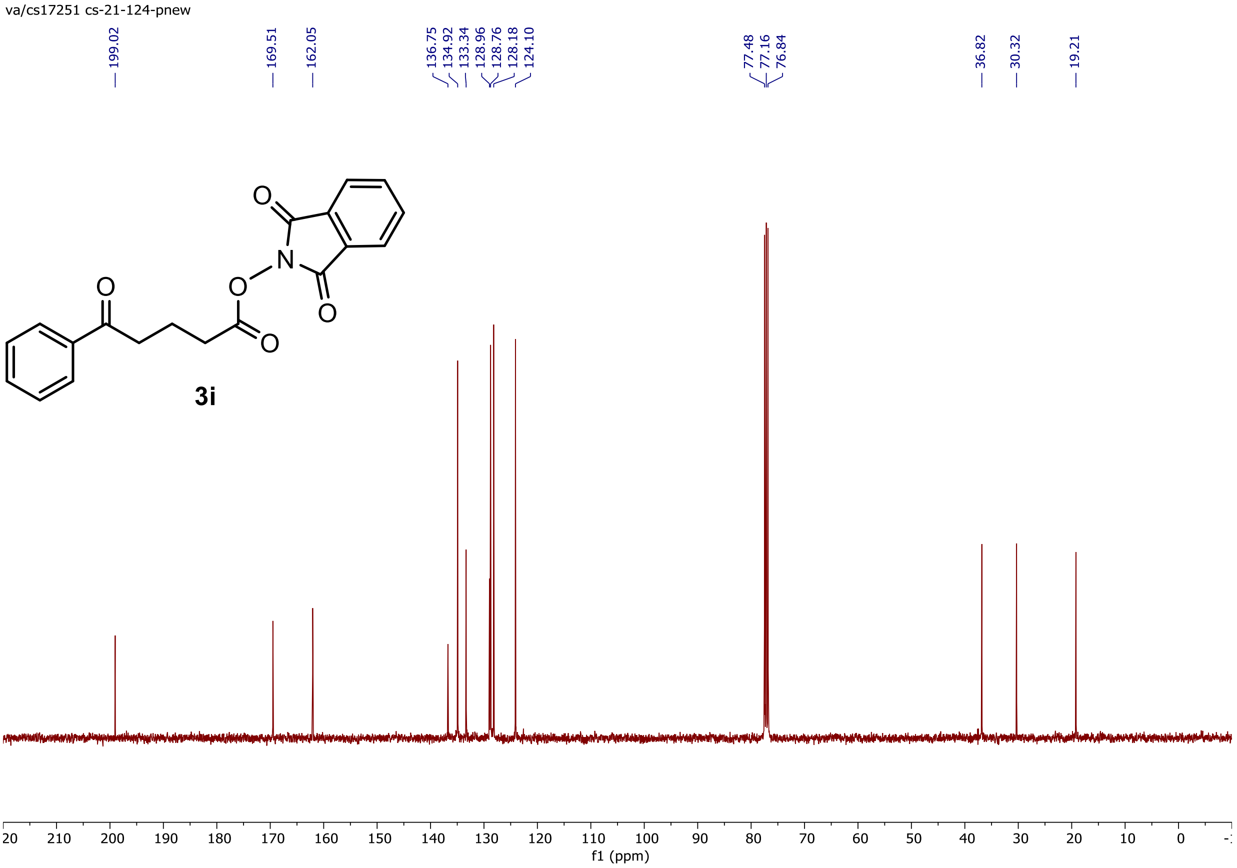

^{1}H NMR (500 MHz, $CDCl_3$) of **3j** (*see procedure*)

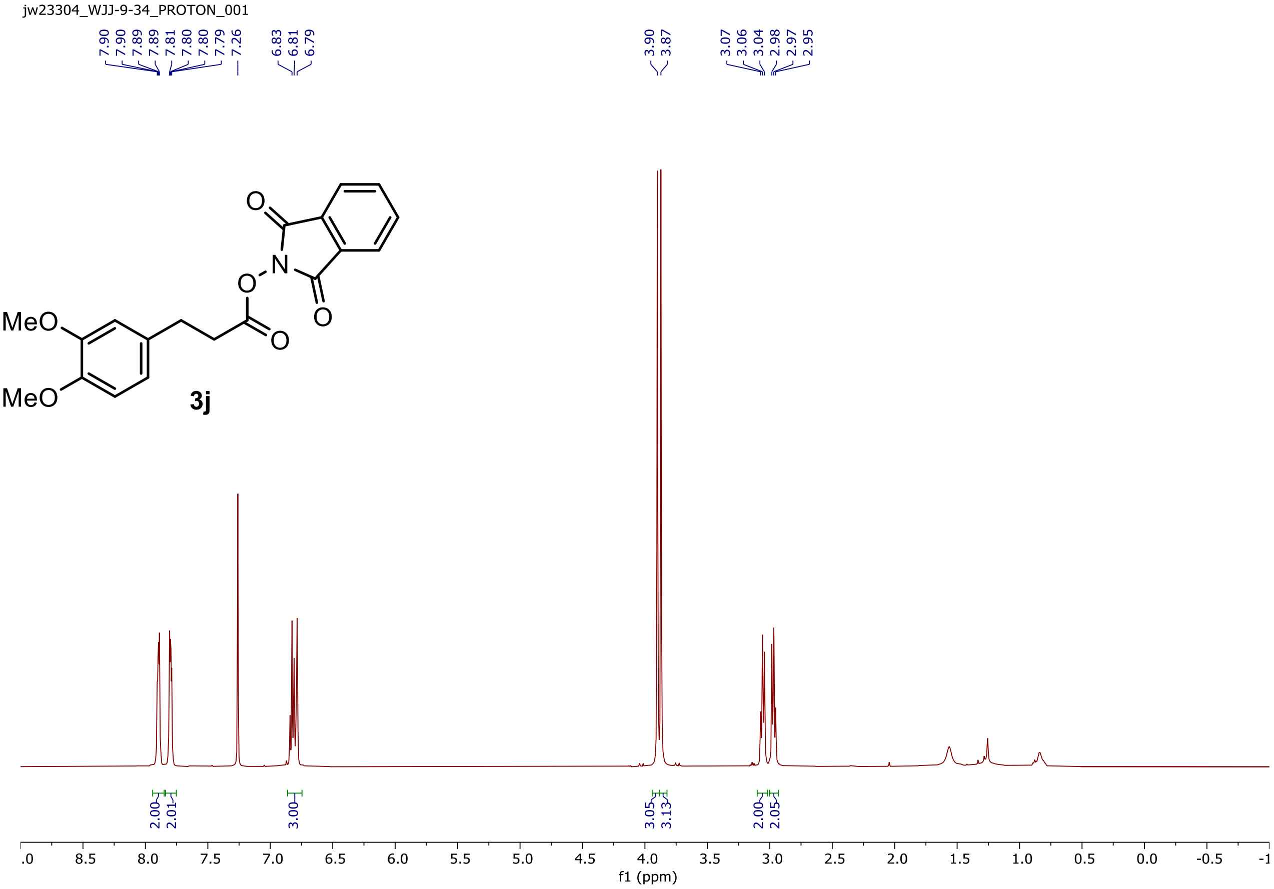

^{13}C NMR (125 MHz, $CDCl_3$) of **3j**

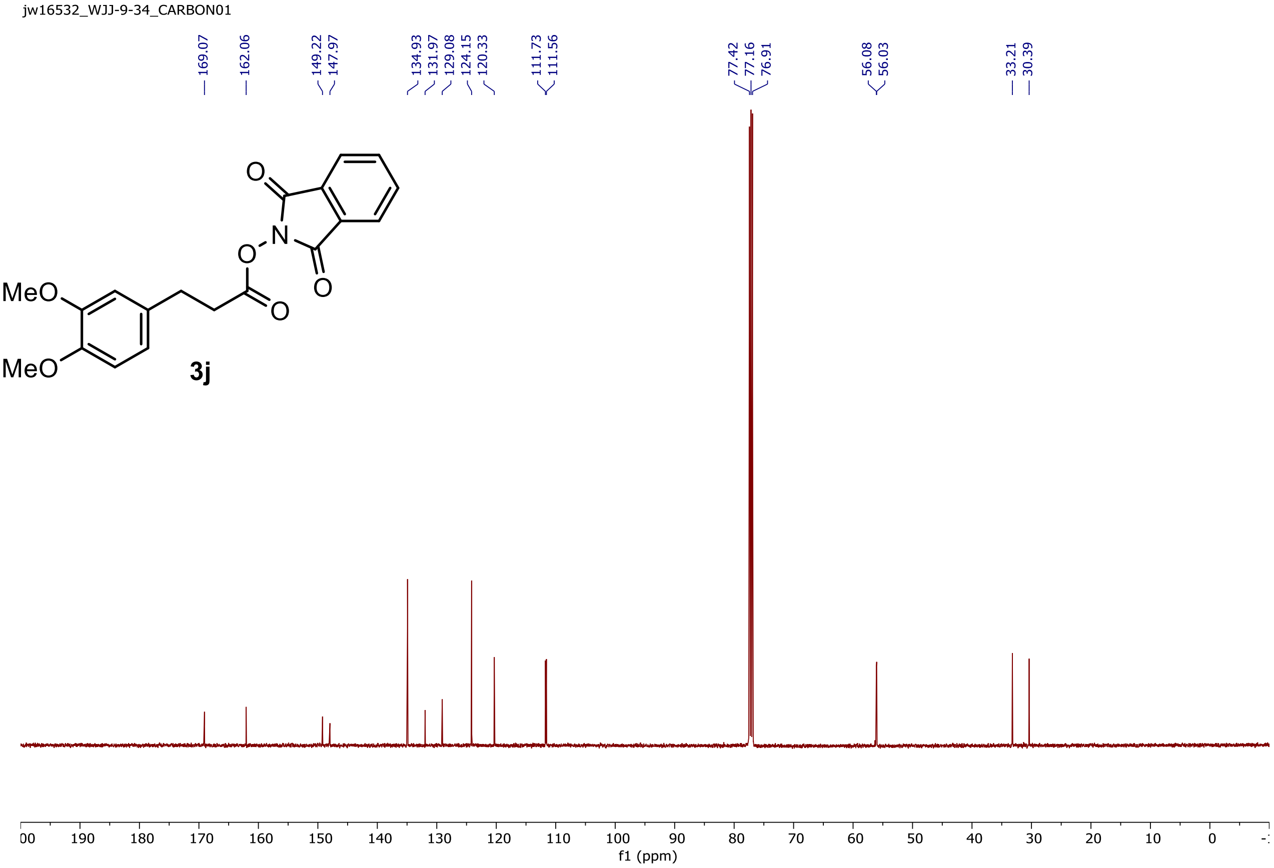

^{1}H NMR (500 MHz, $CDCl_3$) of **3l** (*see procedure*)

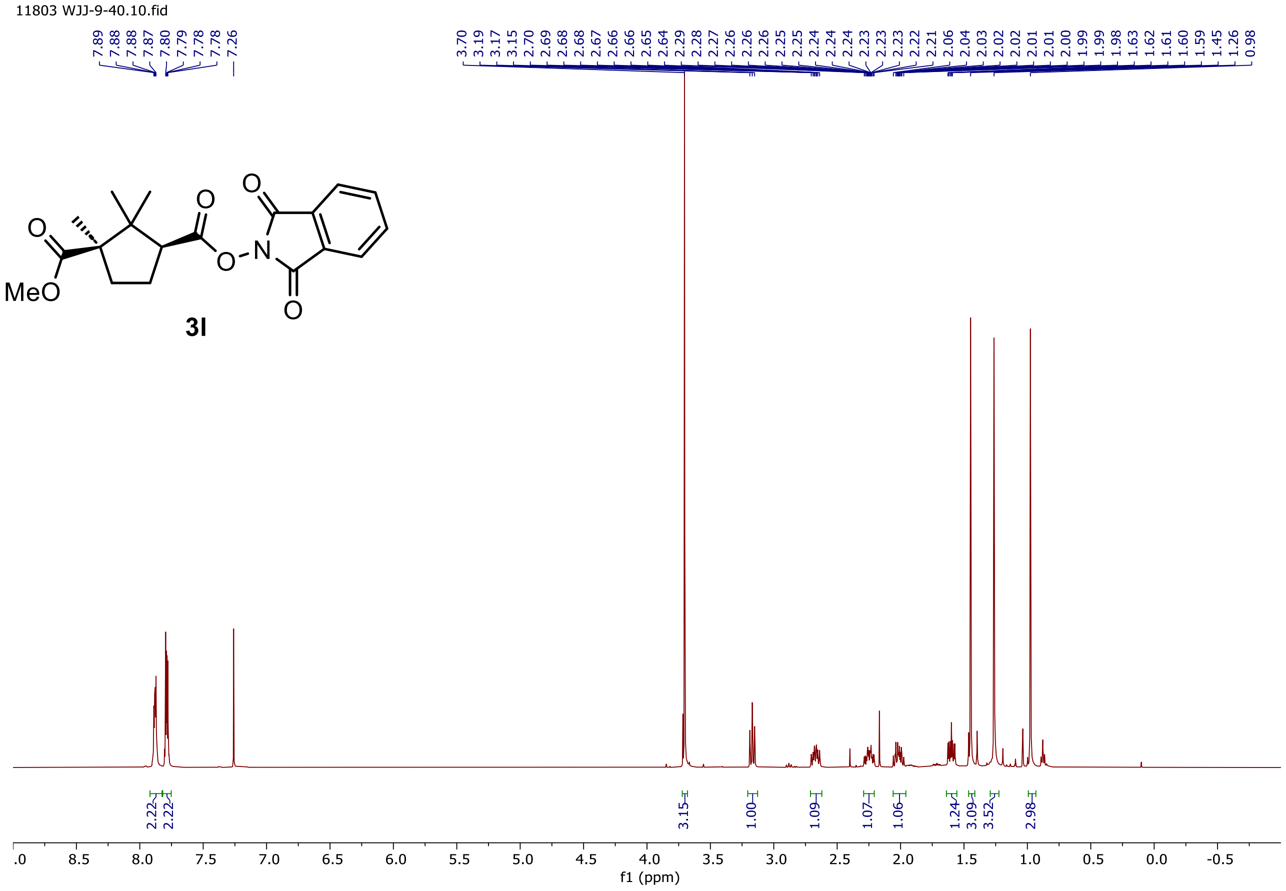

^{13}C NMR (125 MHz, $CDCl_3$) of **3l**

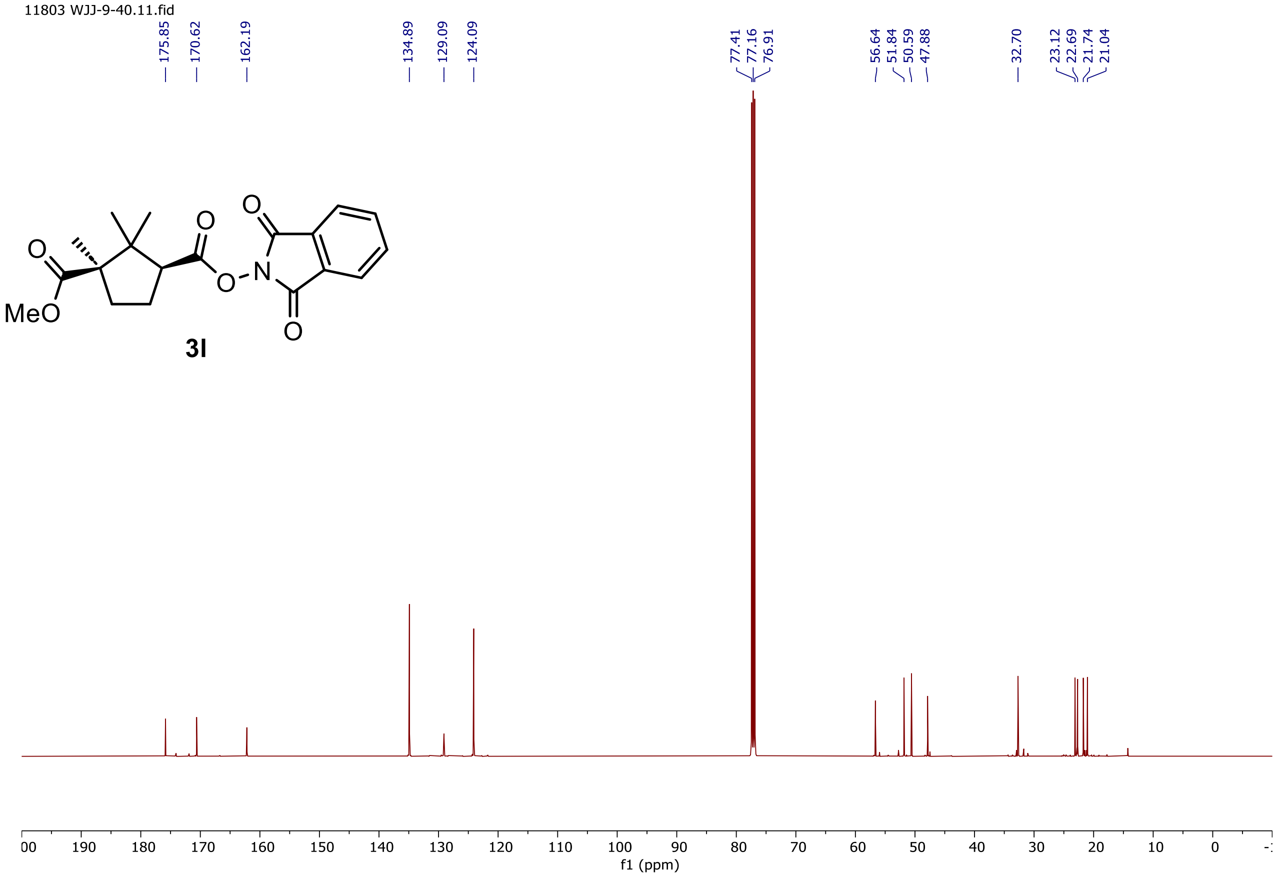

^{1}H NMR (500 MHz, $CDCl_3$) of **3n** (*see procedure*)

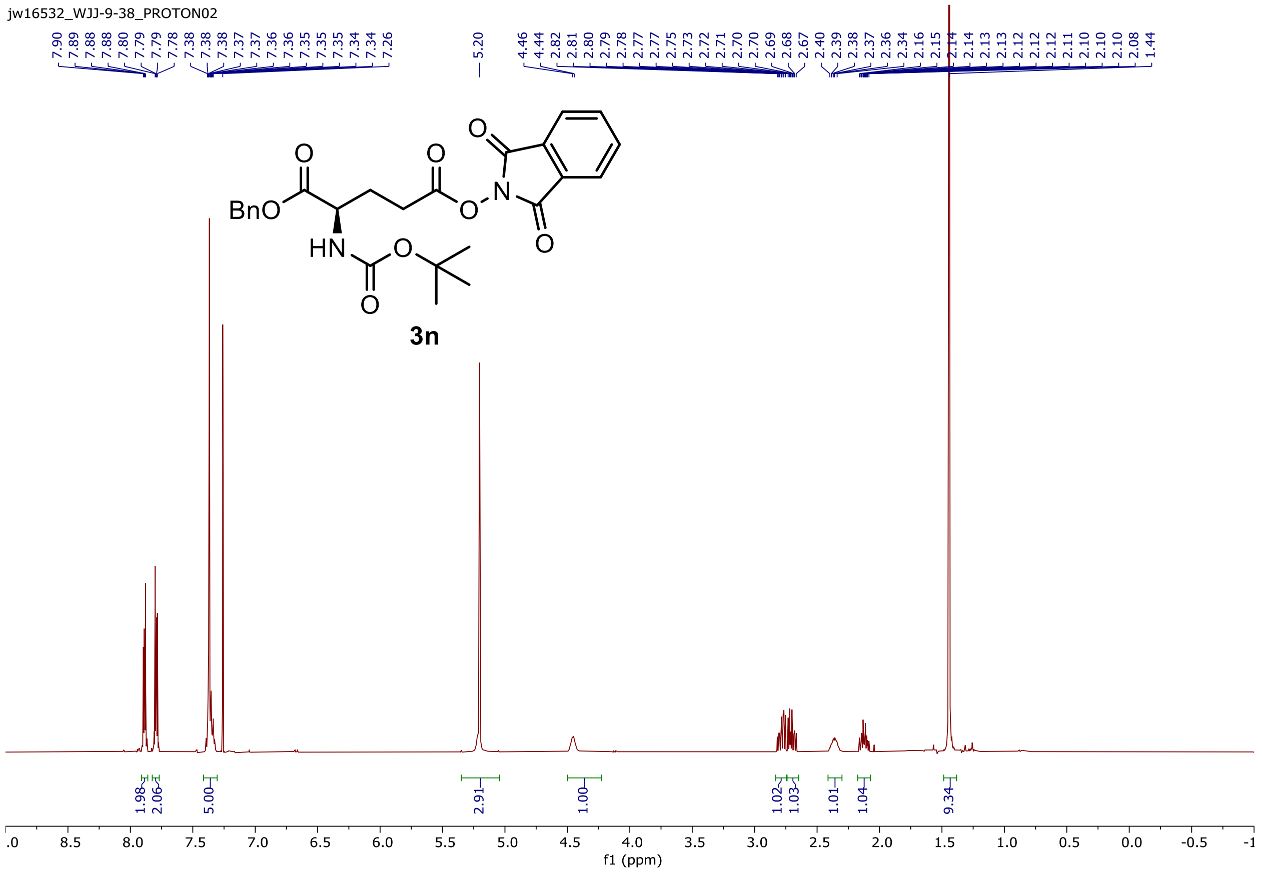

^{13}C NMR (125 MHz, $CDCl_3$) of **3n**

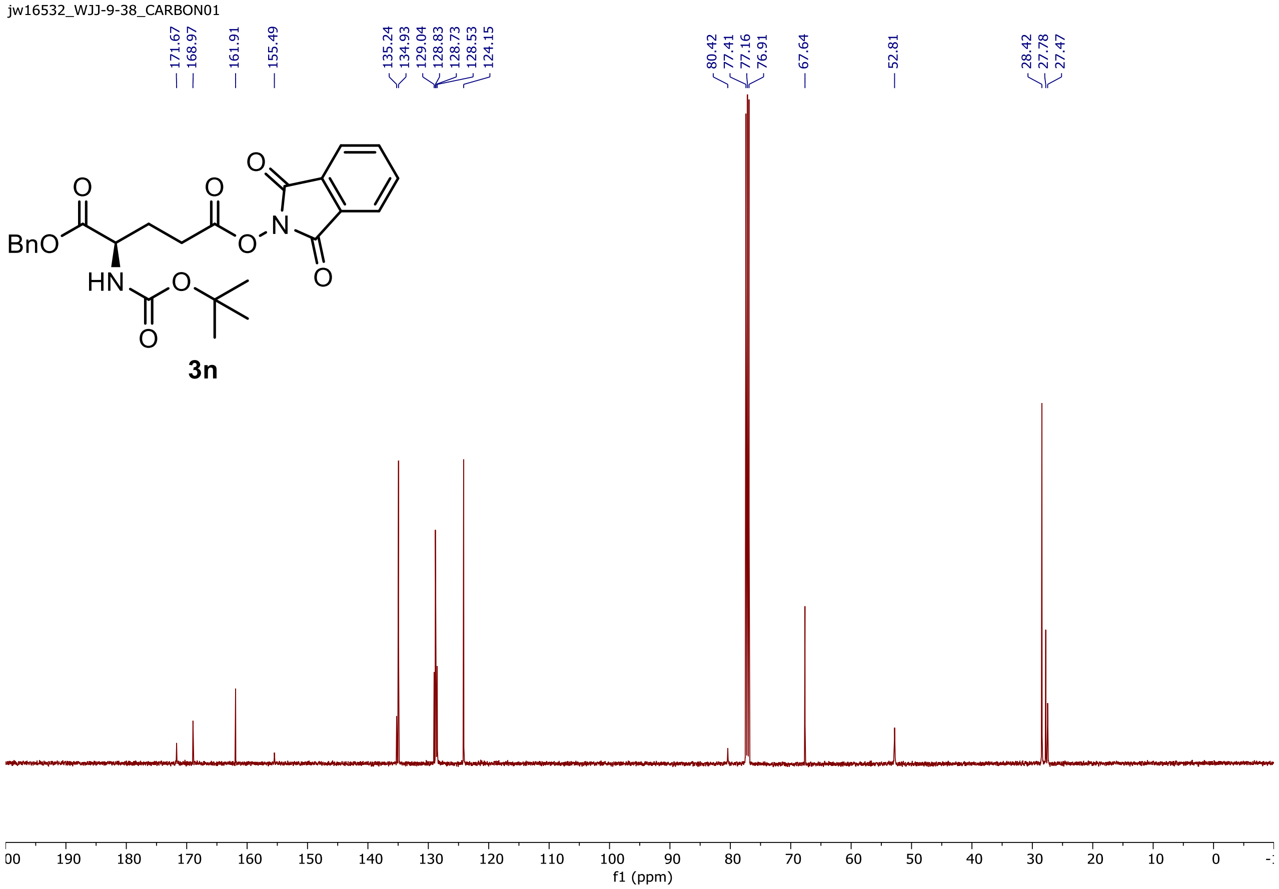

^{1}H NMR (500 MHz, CDCl3) of **3o** (*see procedure*)

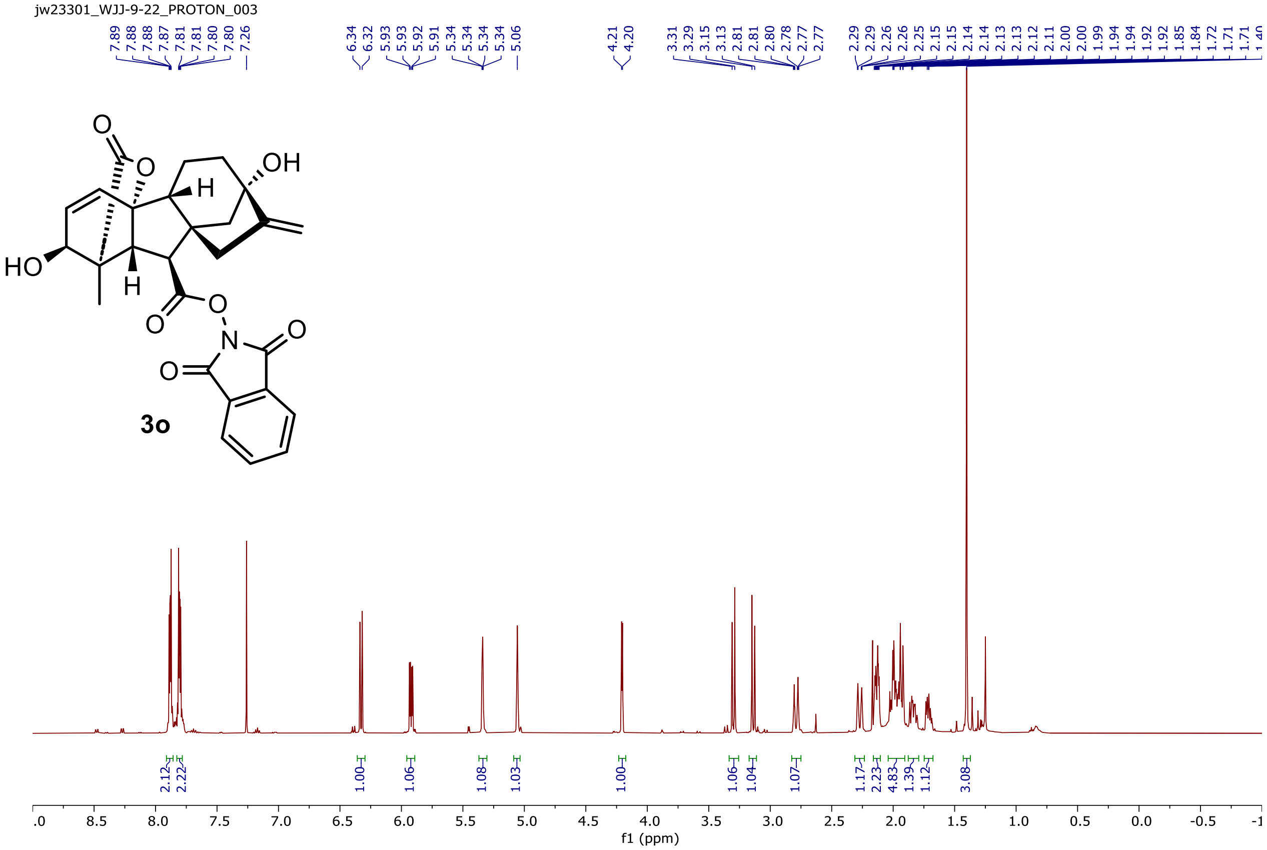

^{13}C NMR (125 MHz, CDCl3) of **3o**

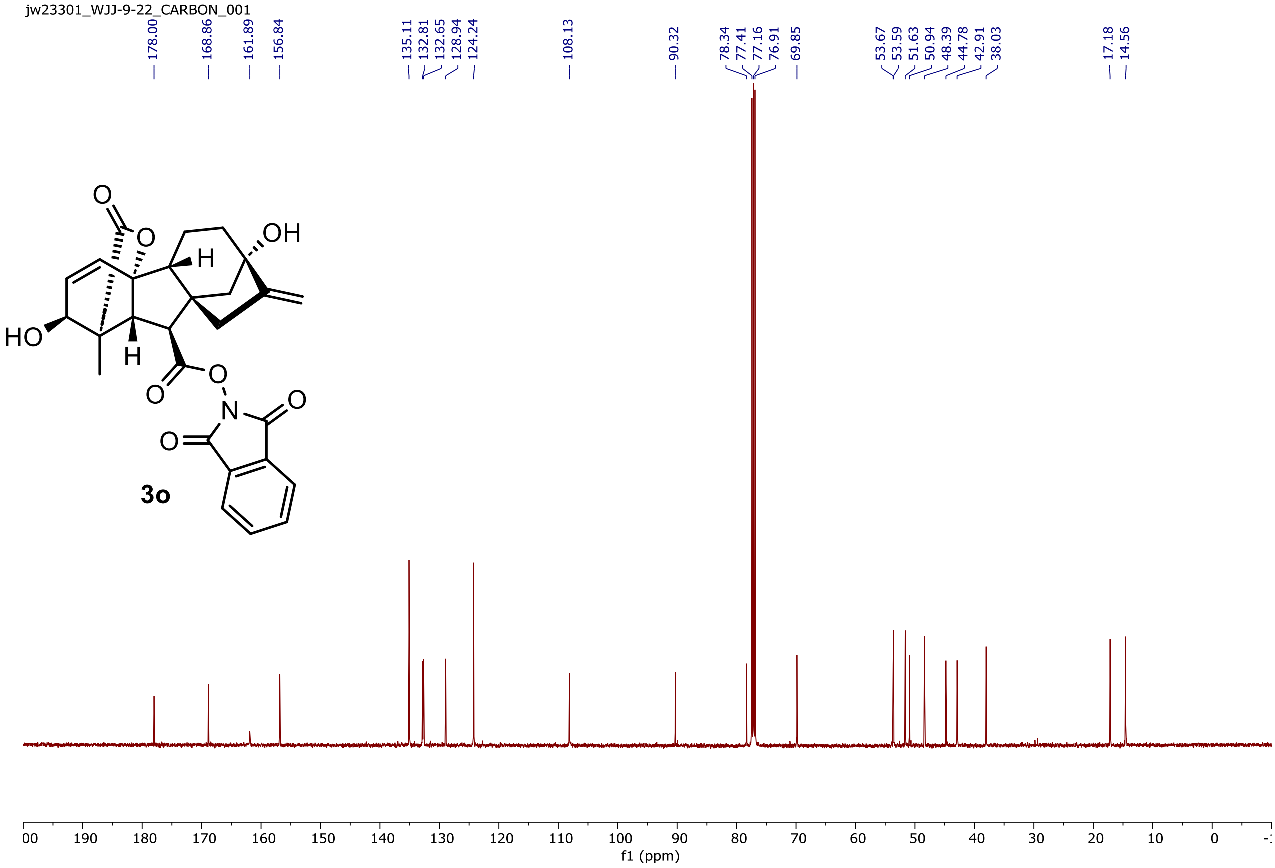

^{1}H–^{1}H COSY ($CDCl_3$) of **3o**

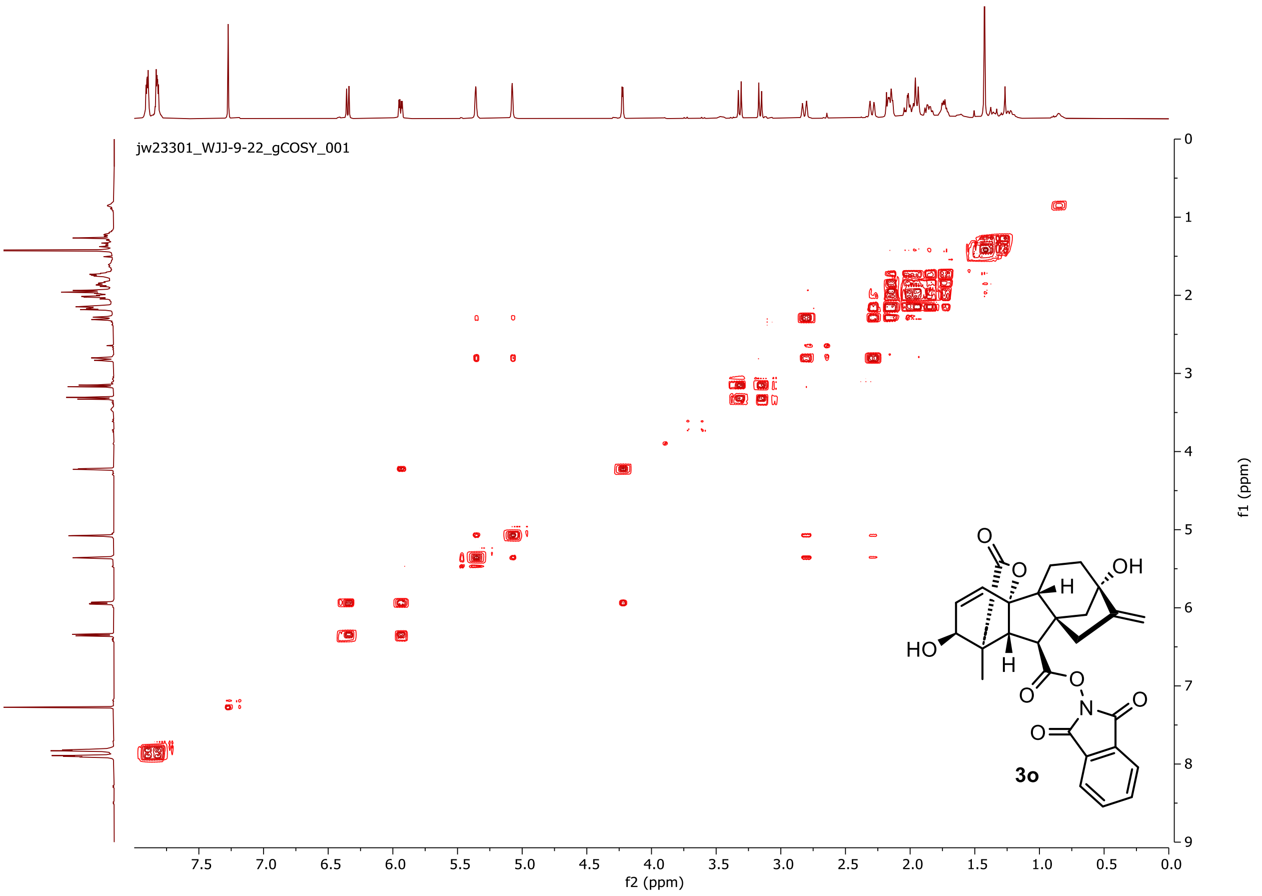

^{1}H–^{13}C HSQC ($CDCl_3$) of **3o**

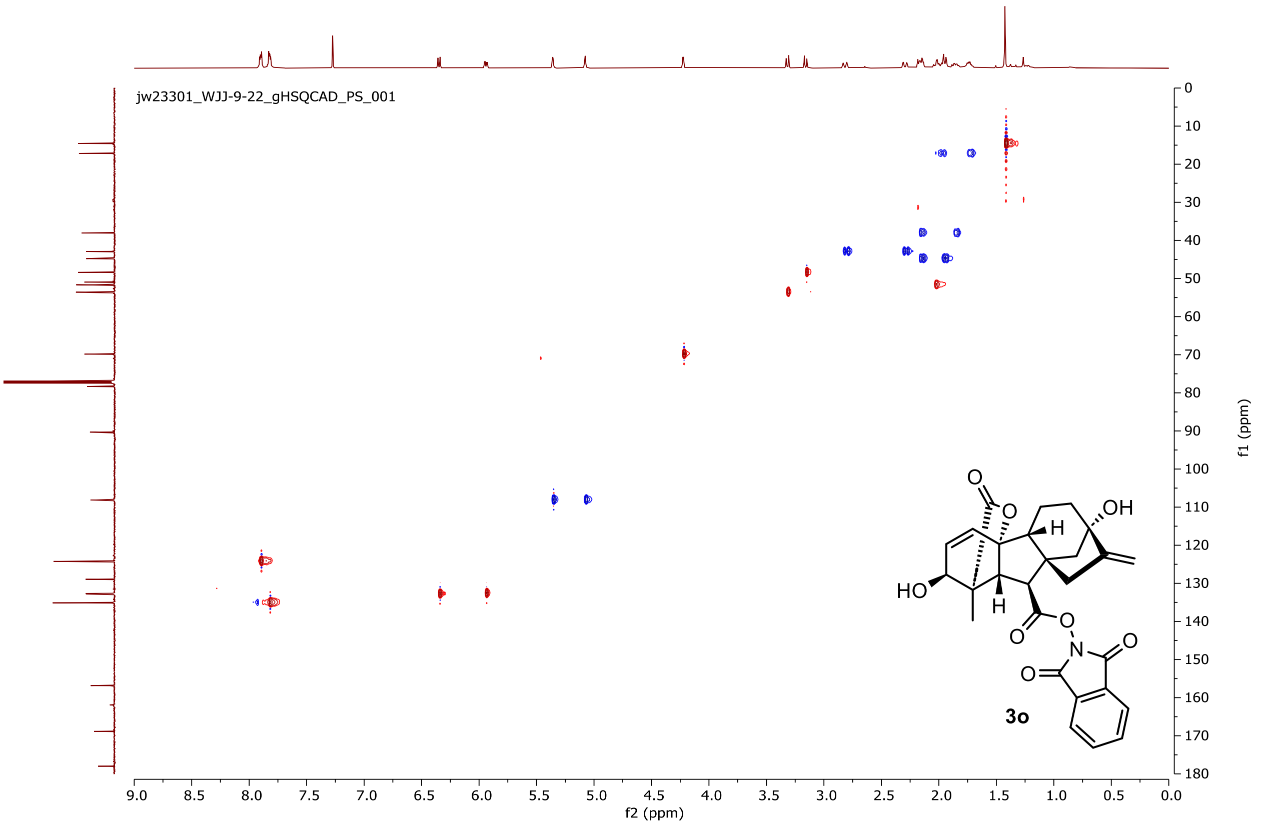

^{1}H NMR (500 MHz, $CDCl_3$) of **4a** (*see procedure*)

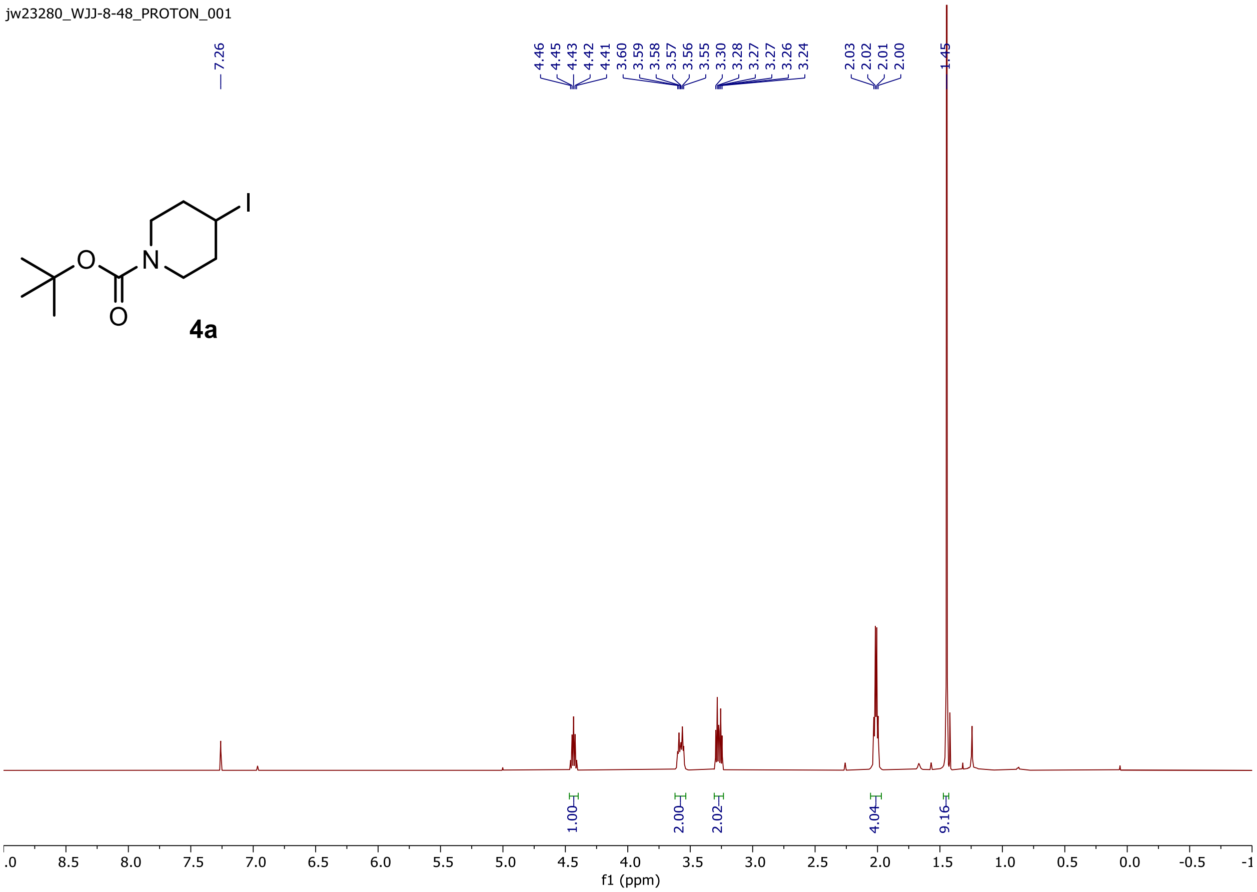

^{13}C NMR (125 MHz, $CDCl_3$) of **4a**

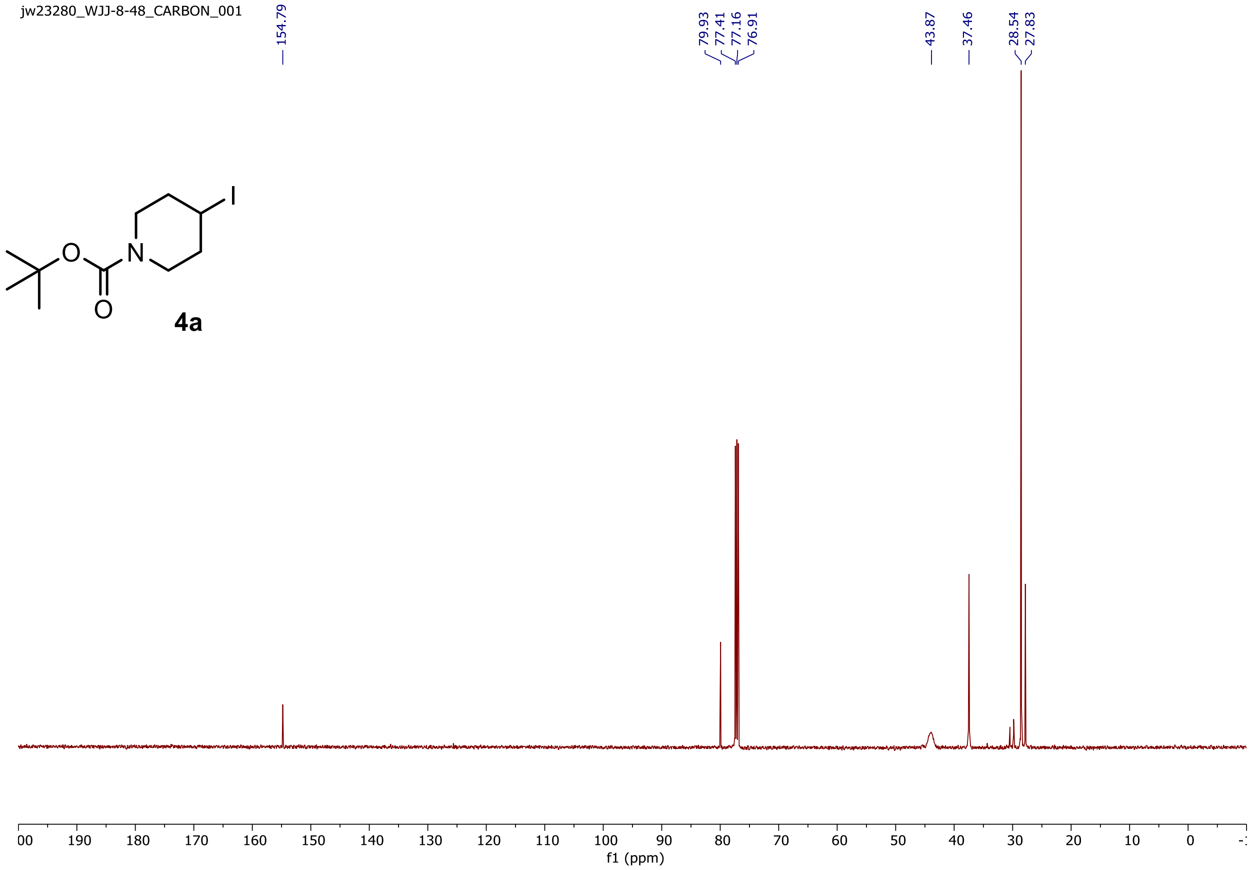

^{1}H NMR (400 MHz, $CDCl_3$) of **5a** (*see procedure*)

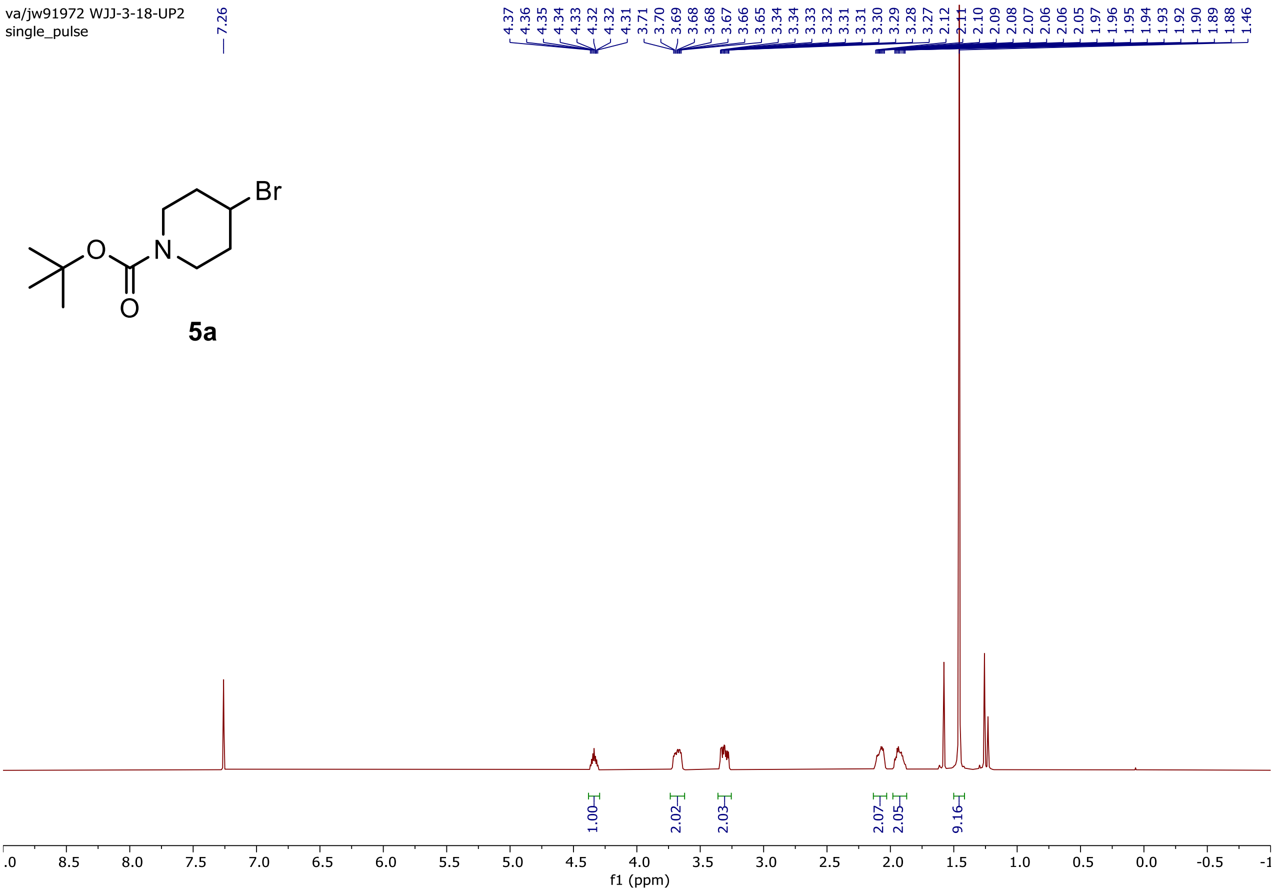

5a

^{1}H NMR (500 MHz, $CDCl_3$) of **5b** (see procedure)

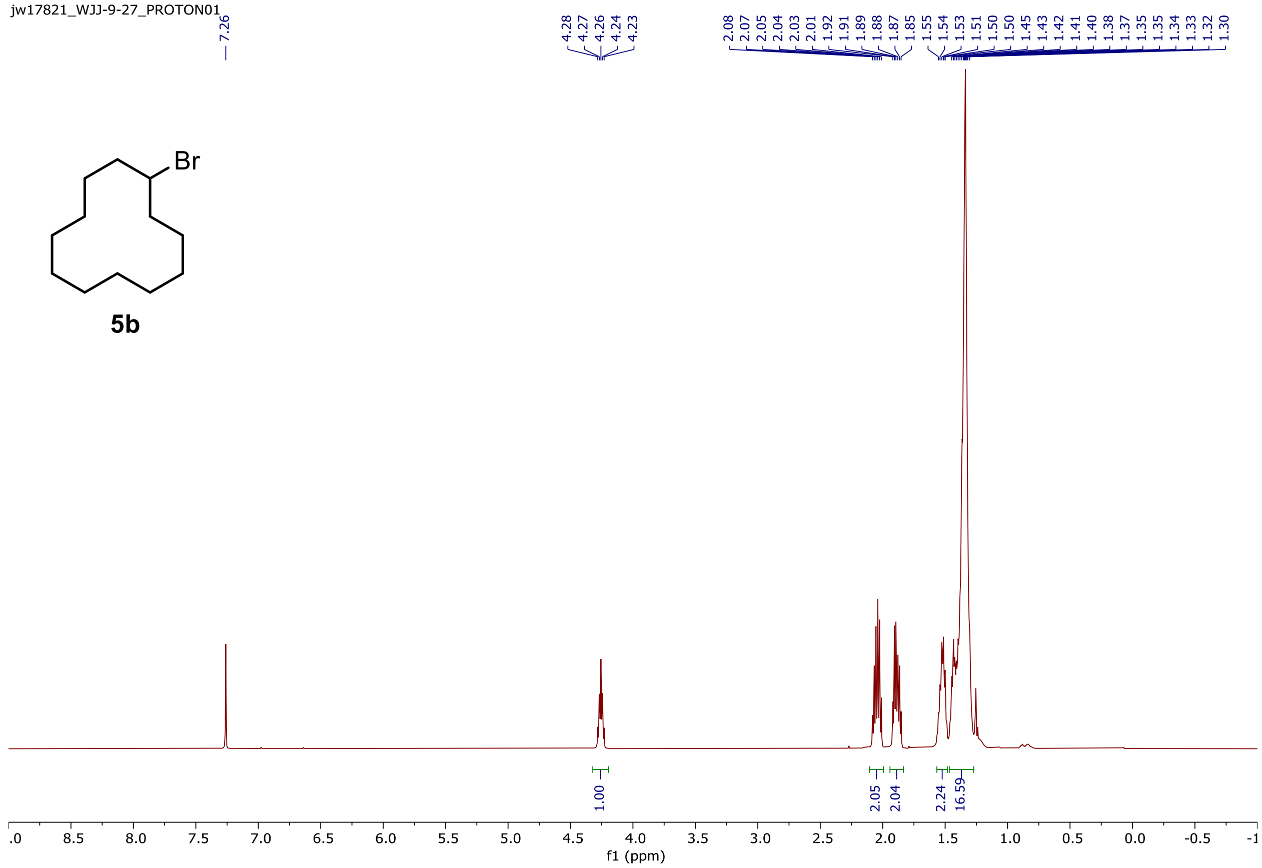

^{13}C NMR (125 MHz, $CDCl_3$) of **5b**

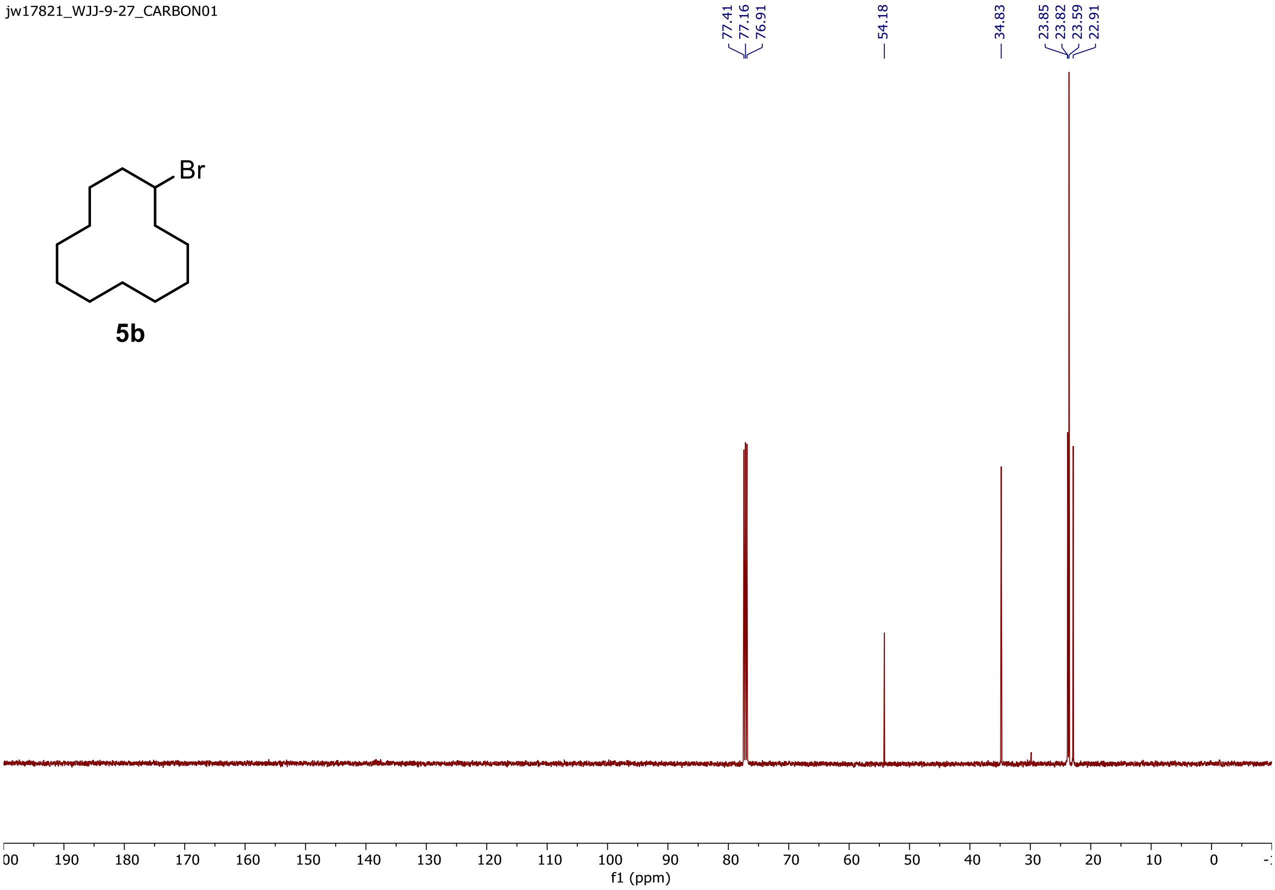

^{1}H NMR (500 MHz, $CDCl_3$) of **5c** (*see procedure*)

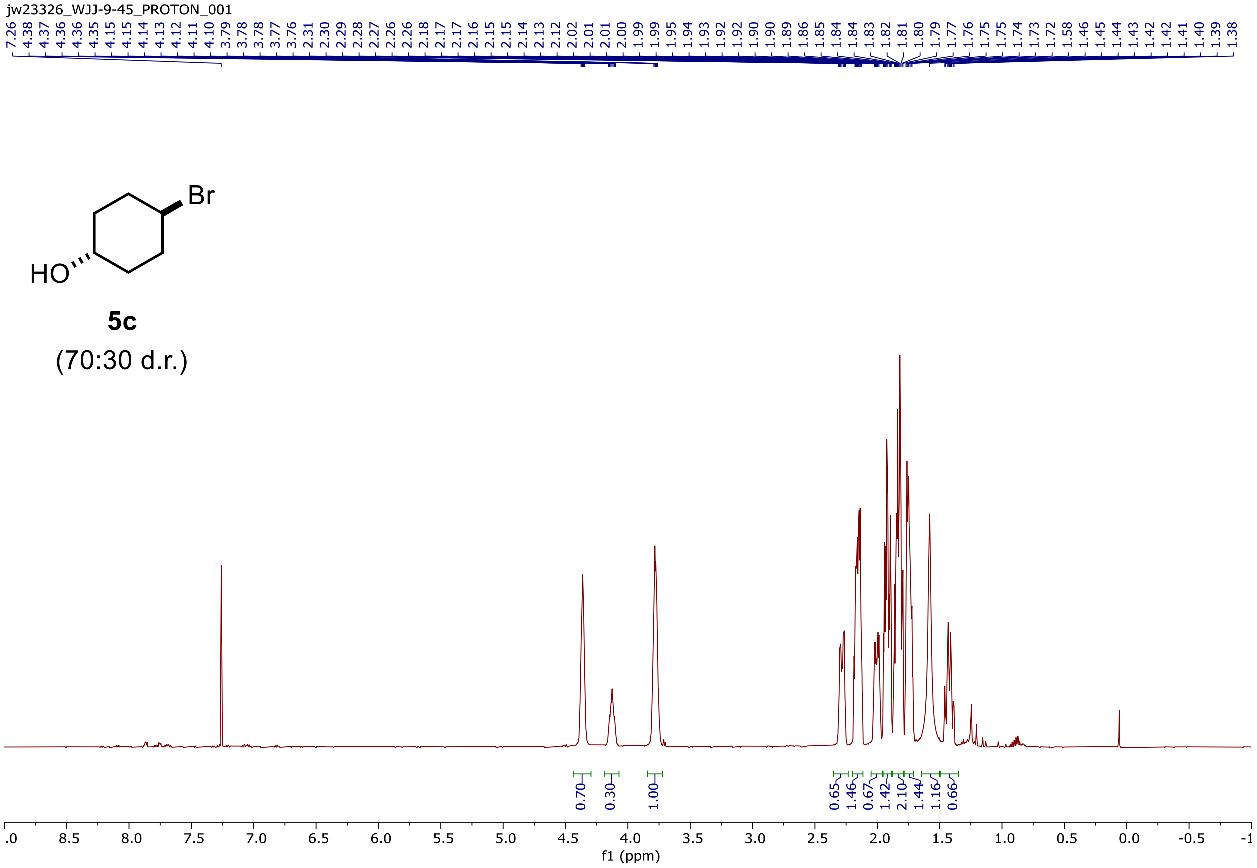

5c

(70:30 d.r.)

^{13}C NMR (125 MHz, $CDCl_3$) of **5c**

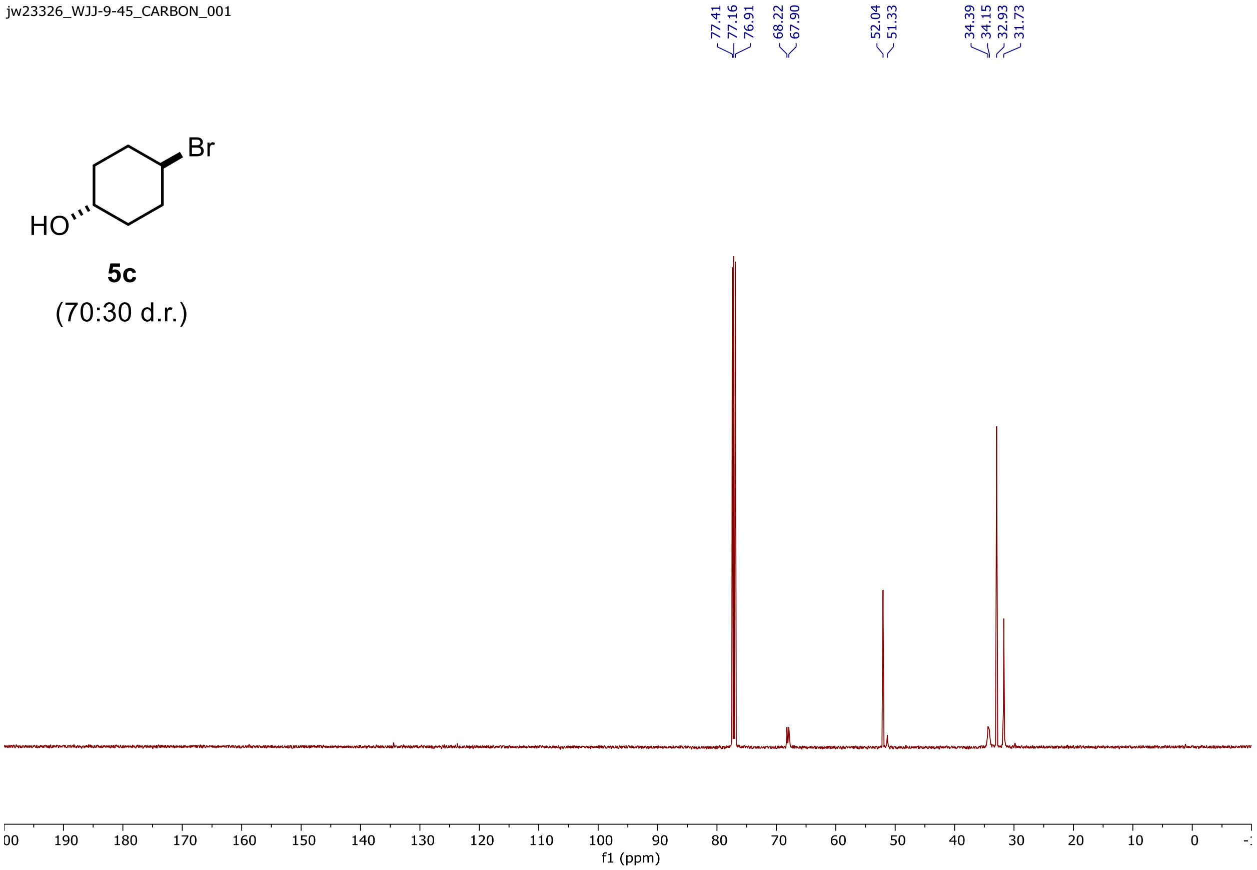

5c

(70:30 d.r.)

^{1}H NMR (500 MHz, $CDCl_3$) of **5d** (*see procedure*)

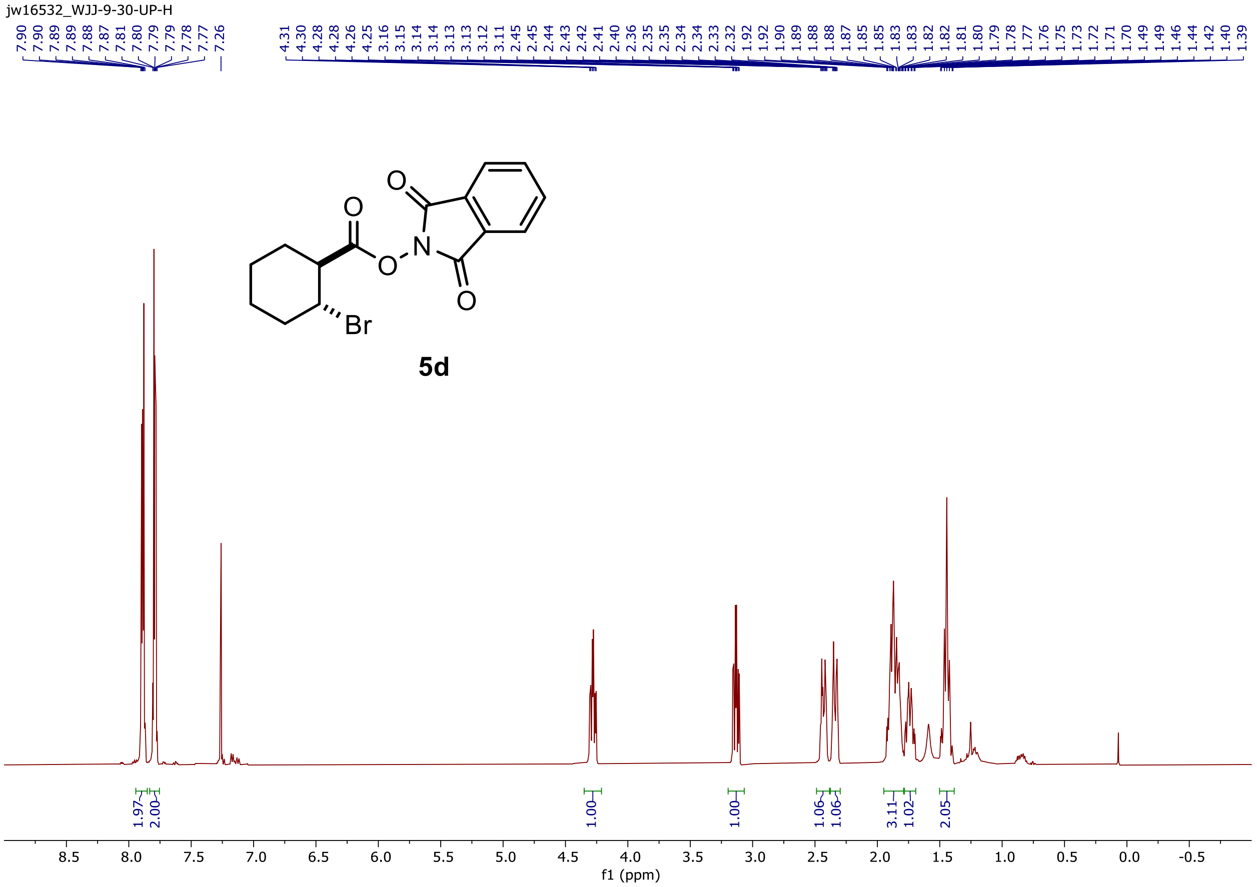

^{13}C NMR (125 MHz, $CDCl_3$) of **5d**

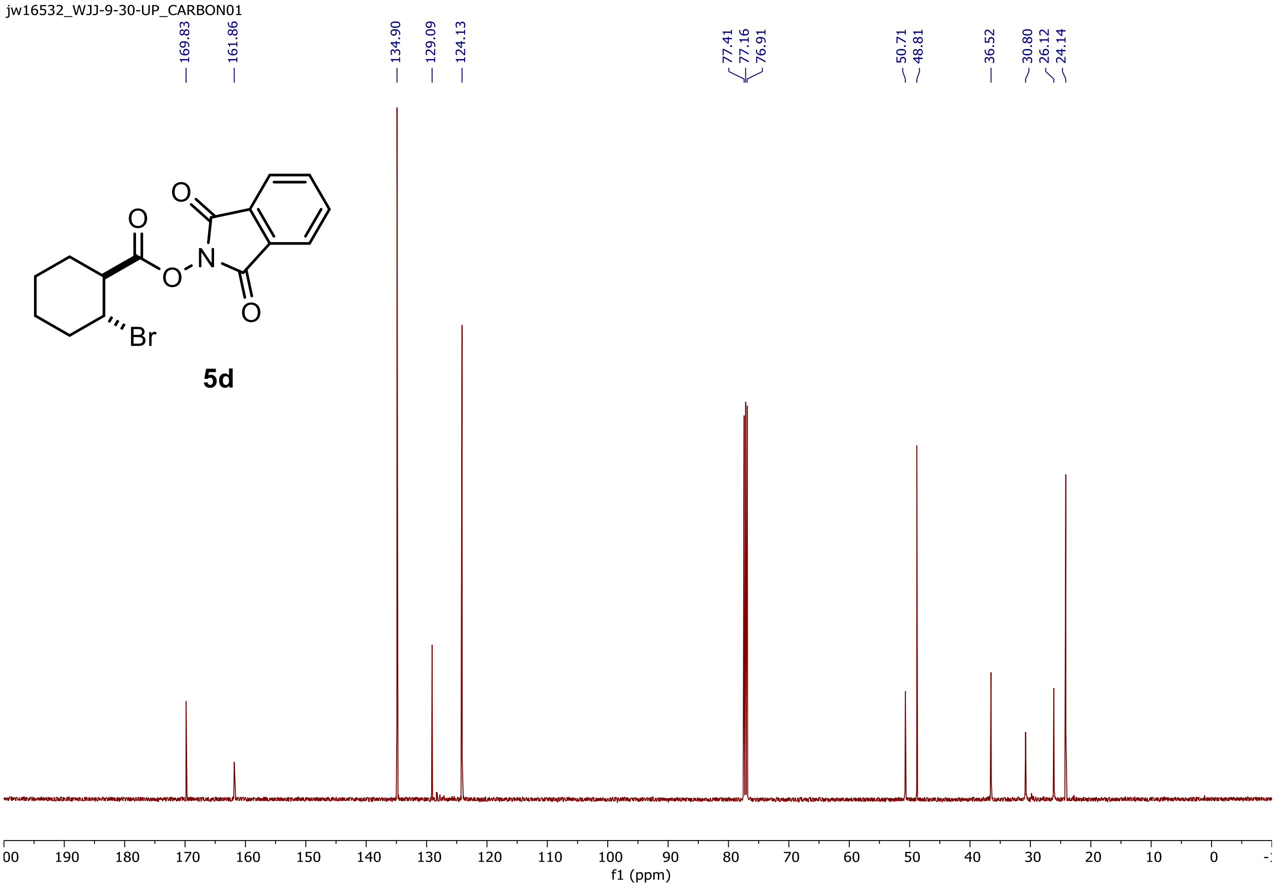

^{1}H NMR (500 MHz, $CDCl_3$) of **9d** (*see procedure*)

jw16532_WJJ-9-30-d_PROTON02

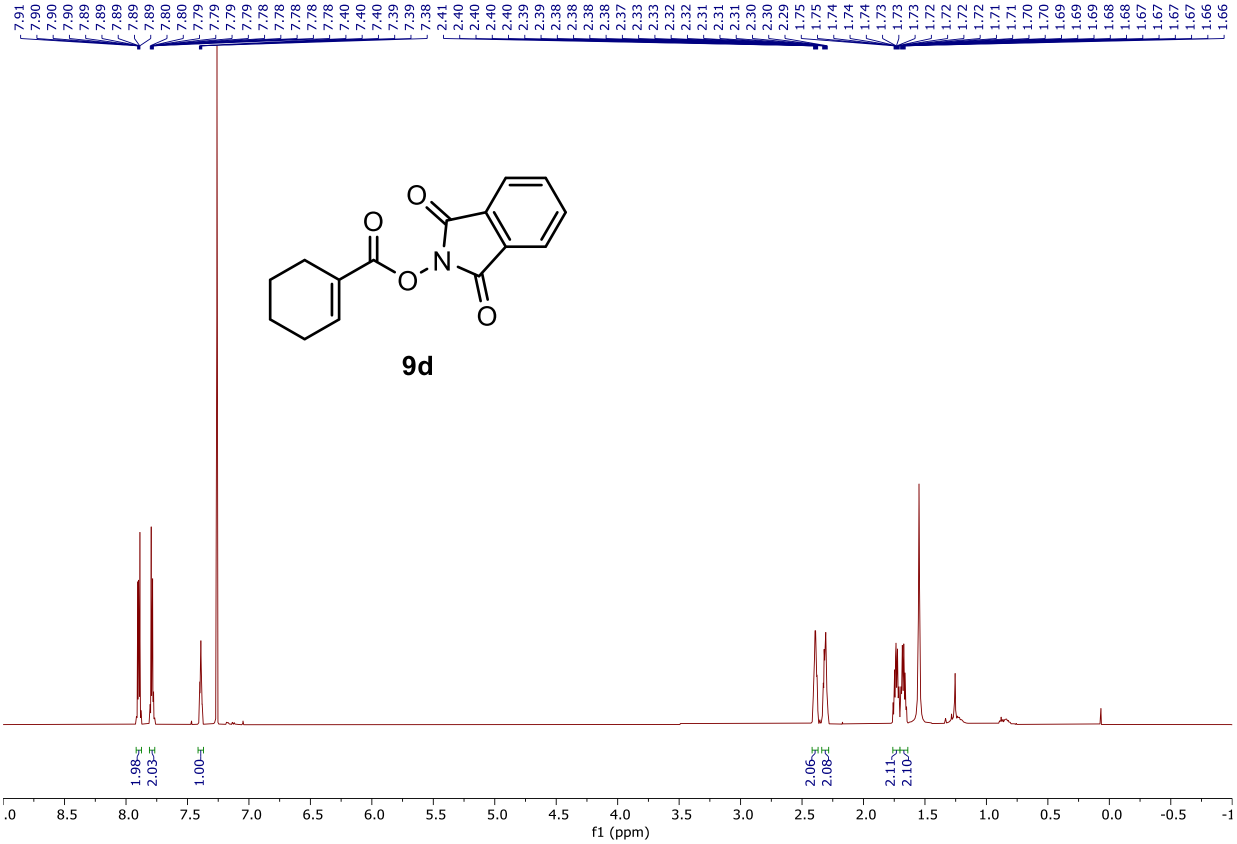

^{13}C NMR (125 MHz, $CDCl_3$) of **9d**

jw16532_WJJ-9-30-d_CARBON01

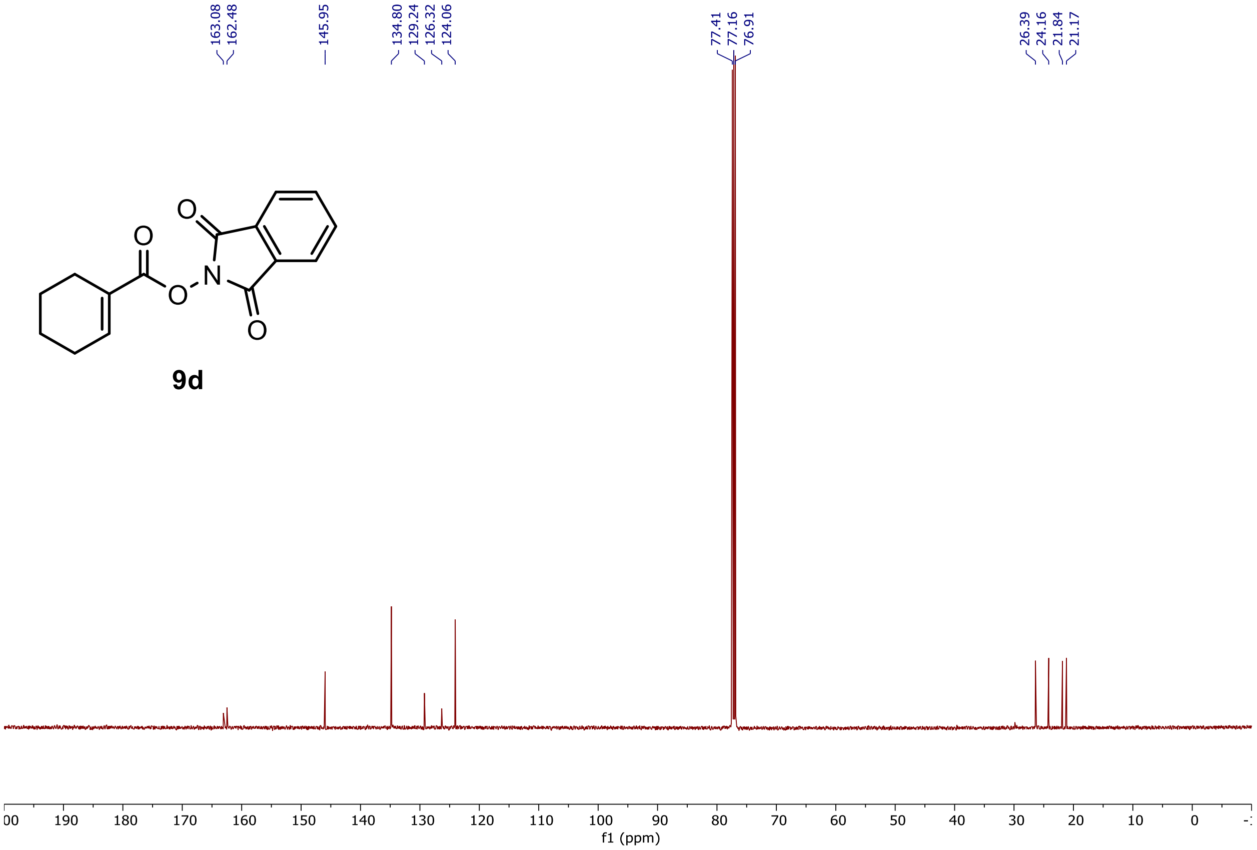

^{1}H NMR (500 MHz, $CDCl_3$) of **5e** (*see procedure*)

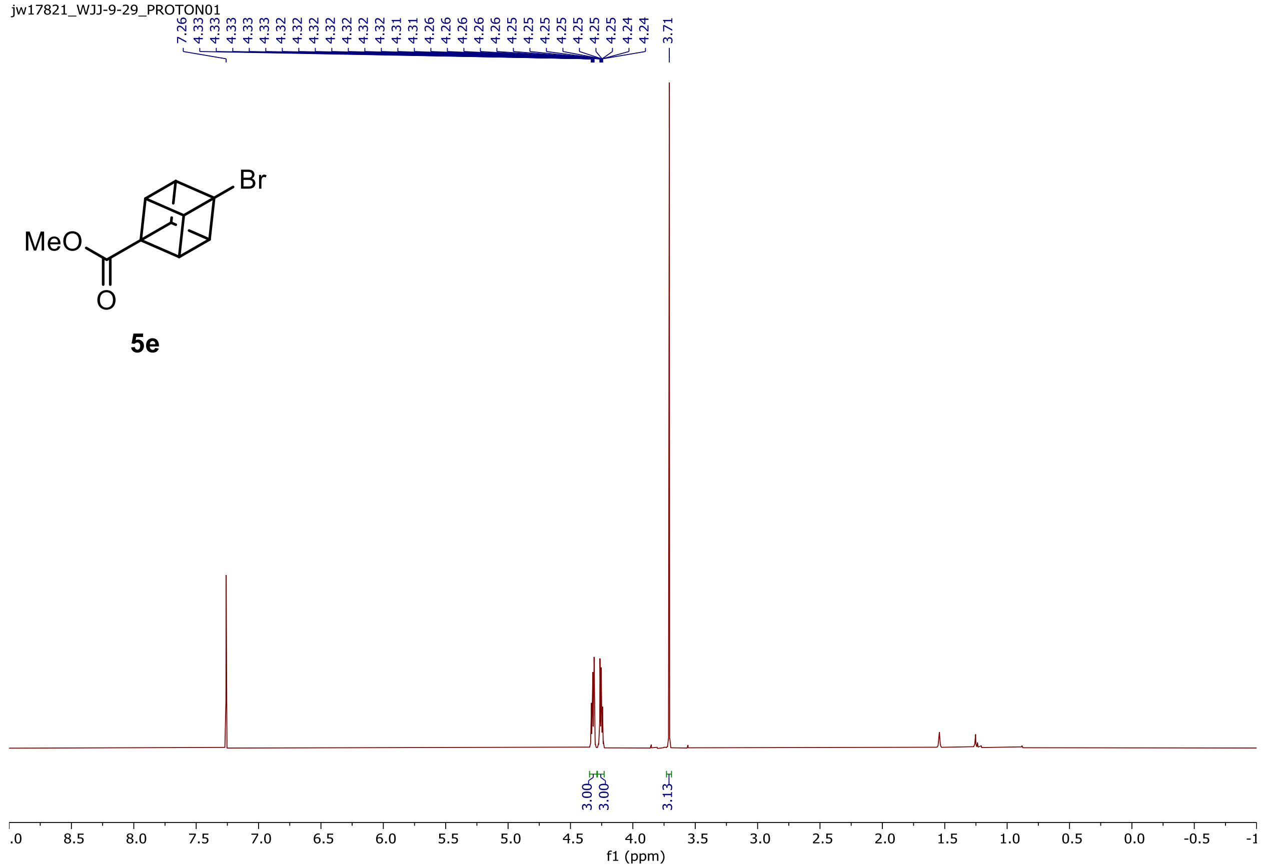

^{13}C NMR (125 MHz, $CDCl_3$) of **5e**

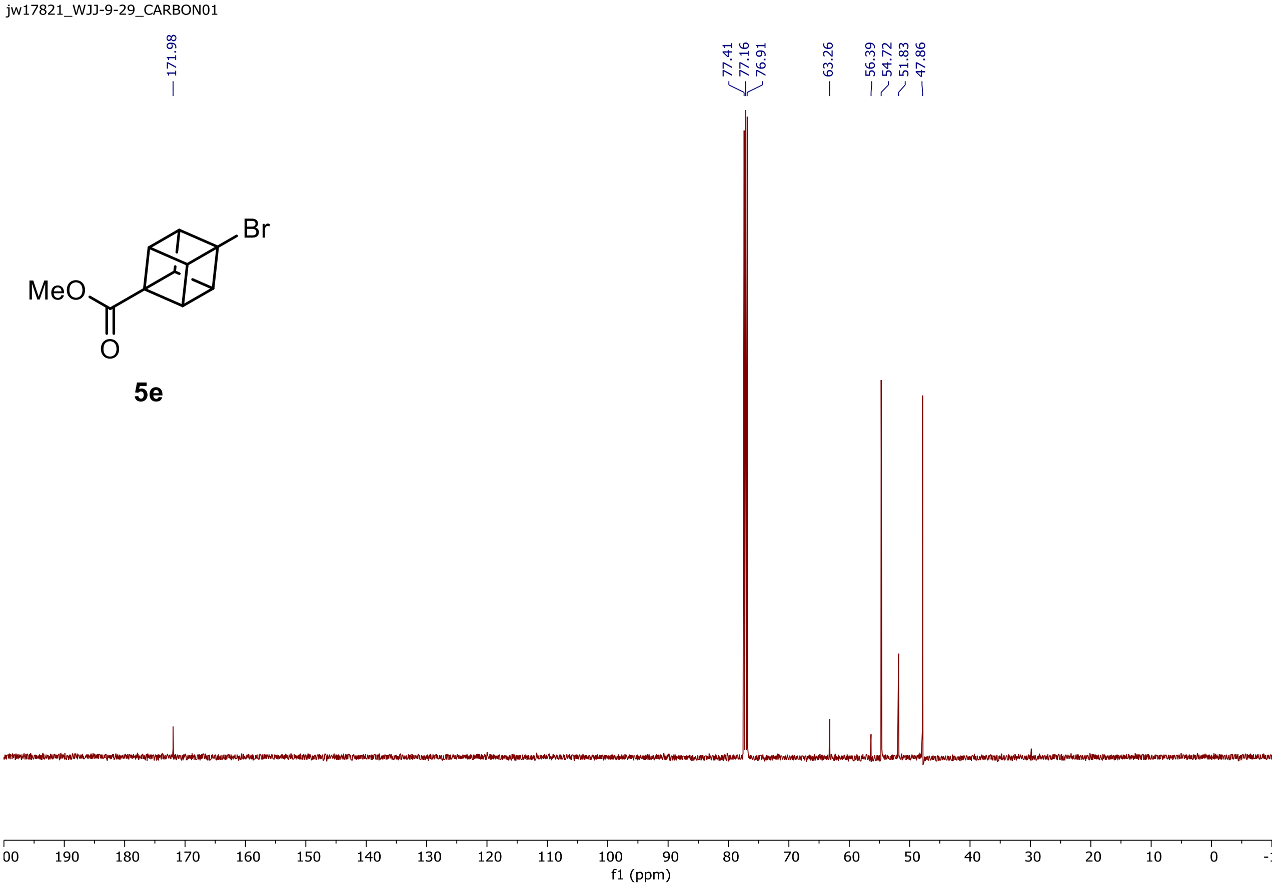

^{1}H NMR (500 MHz, $CDCl_3$) of **8e** (*see procedure*)

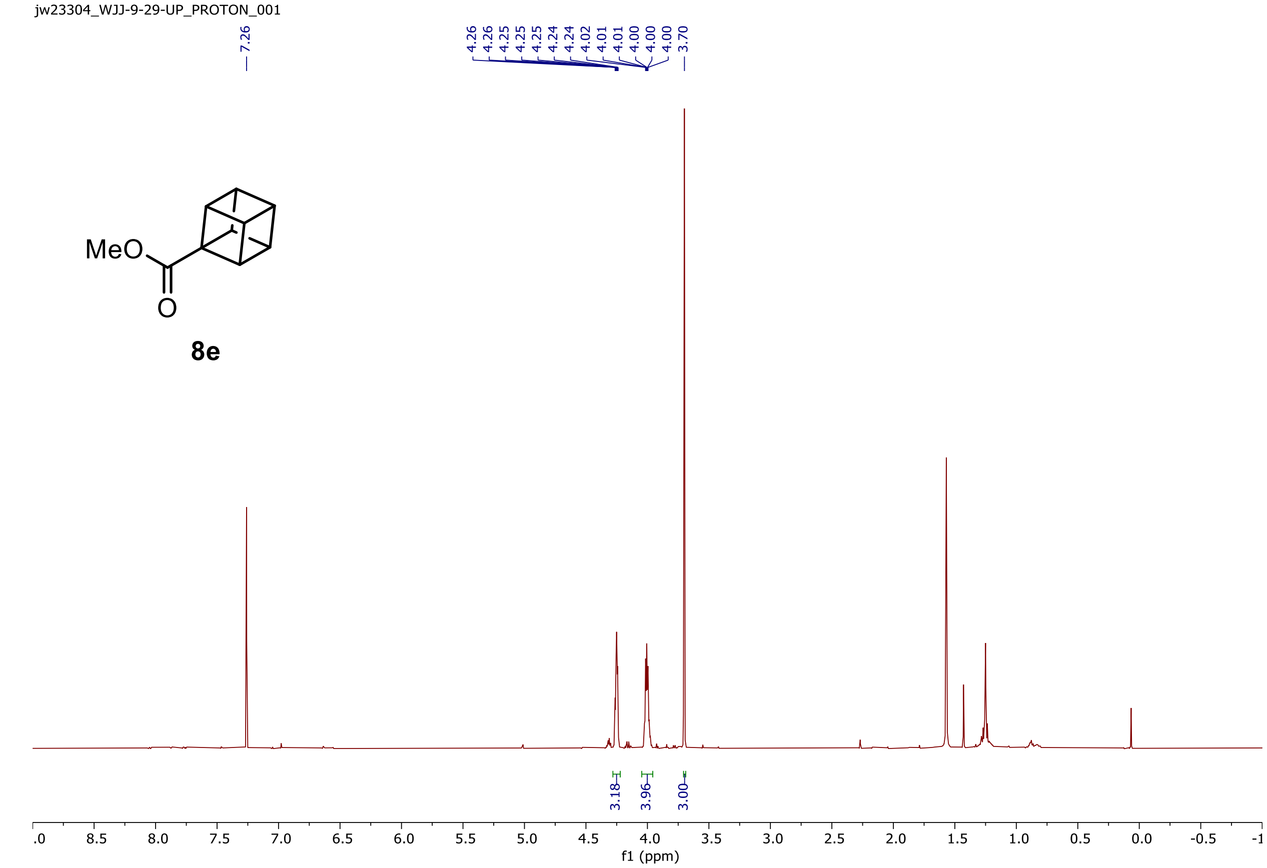

^{13}C NMR (125 MHz, $CDCl_3$) of **8e**

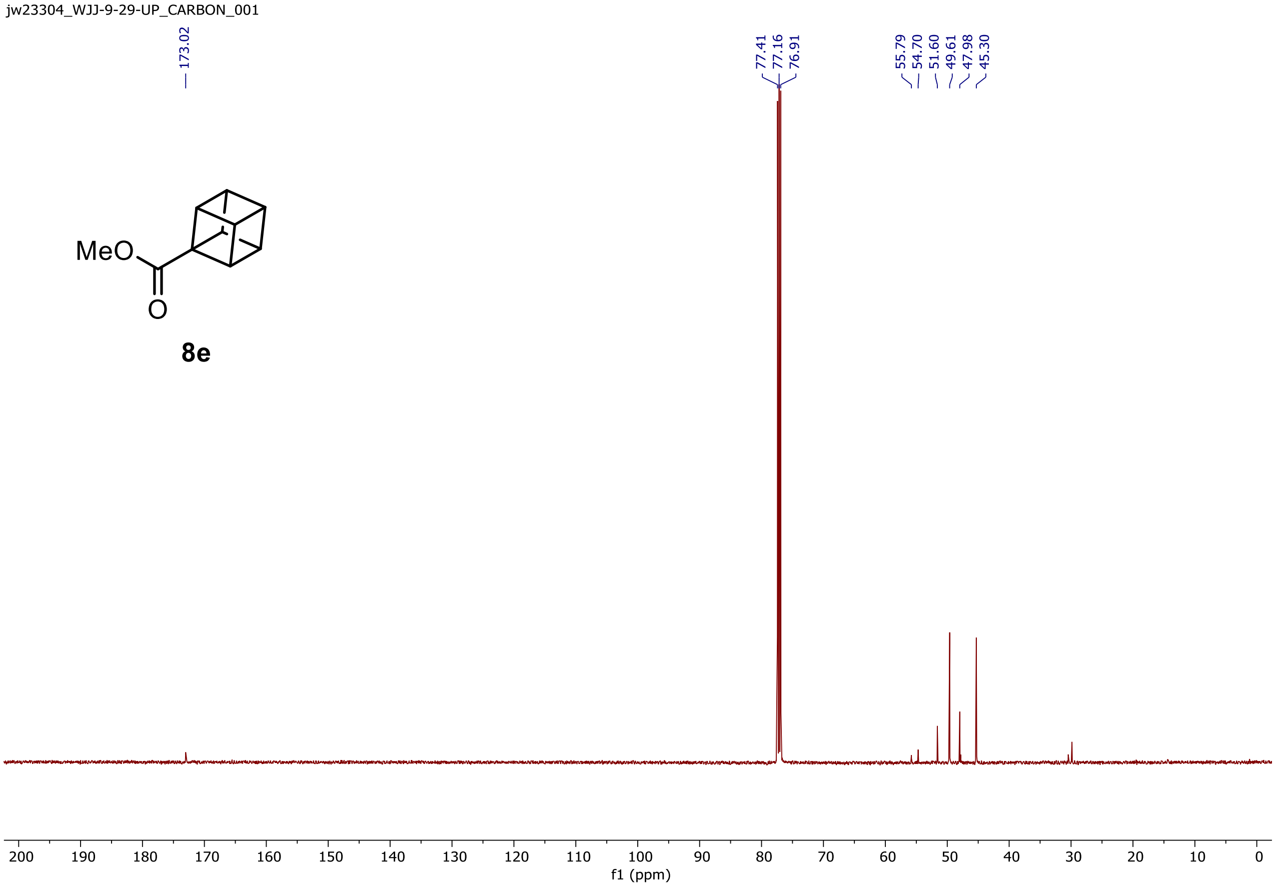

^{1}H NMR (500 MHz, $CDCl_3$) of **5f** (*see procedure*)

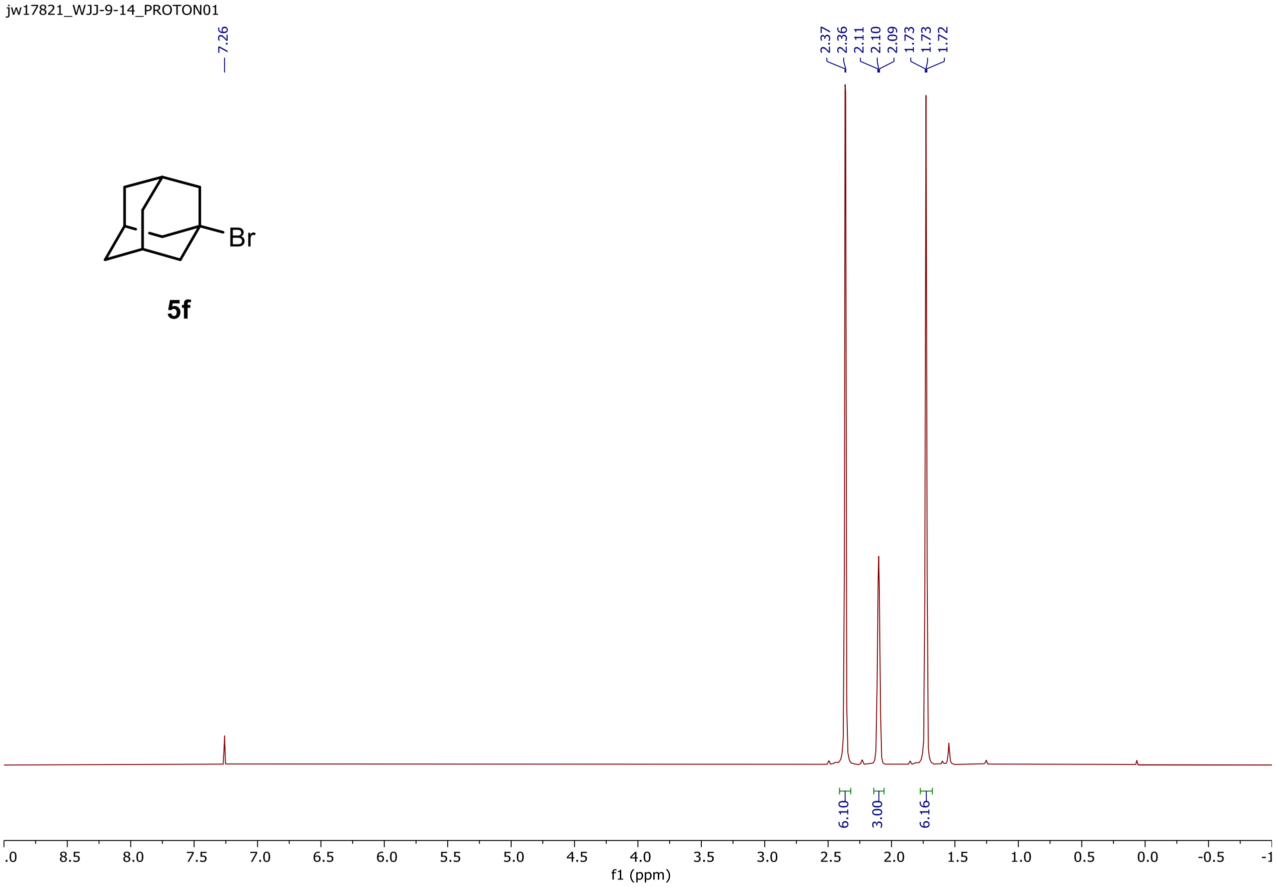

^{13}C NMR (125 MHz, $CDCl_3$) of **5f**

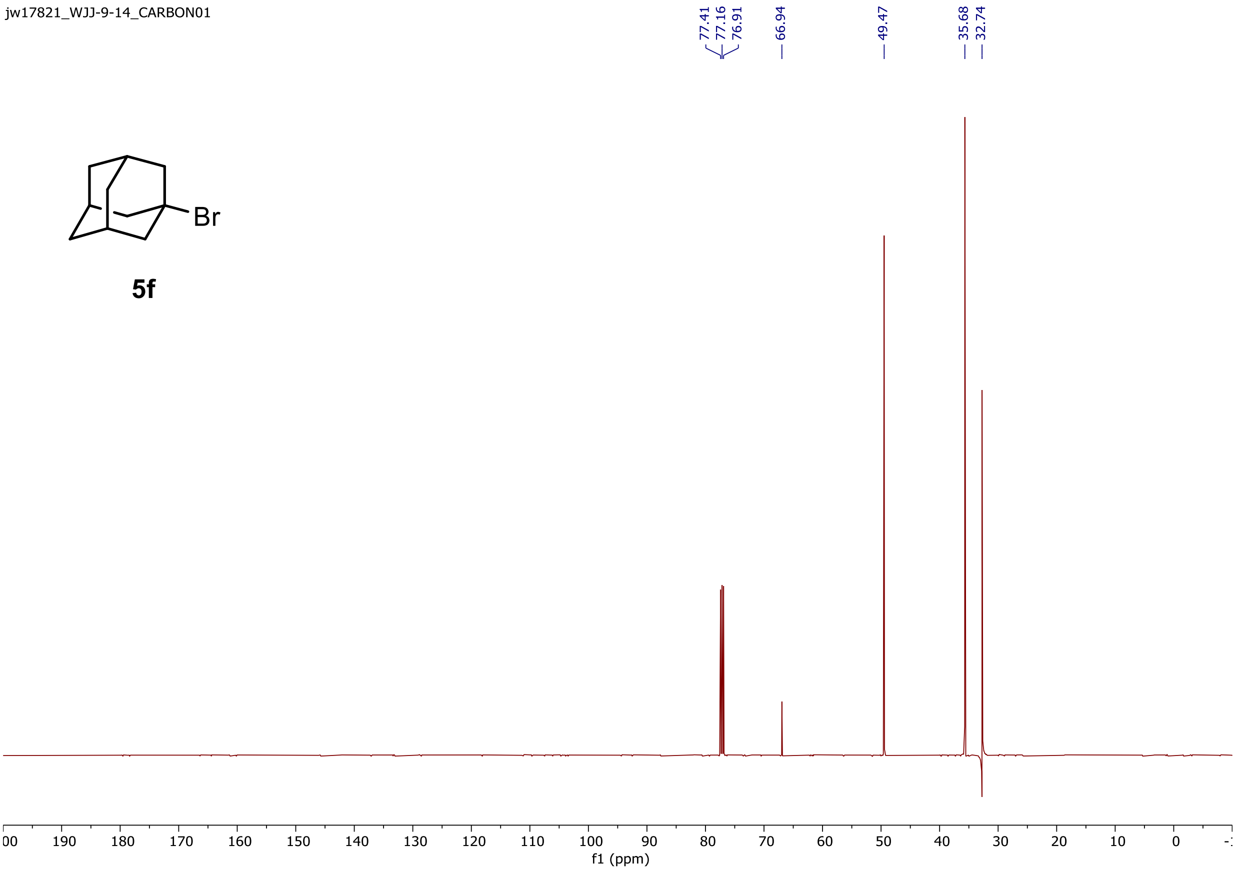

^{1}H NMR (500 MHz, $CDCl_3$) of **5f'** (see procedure)

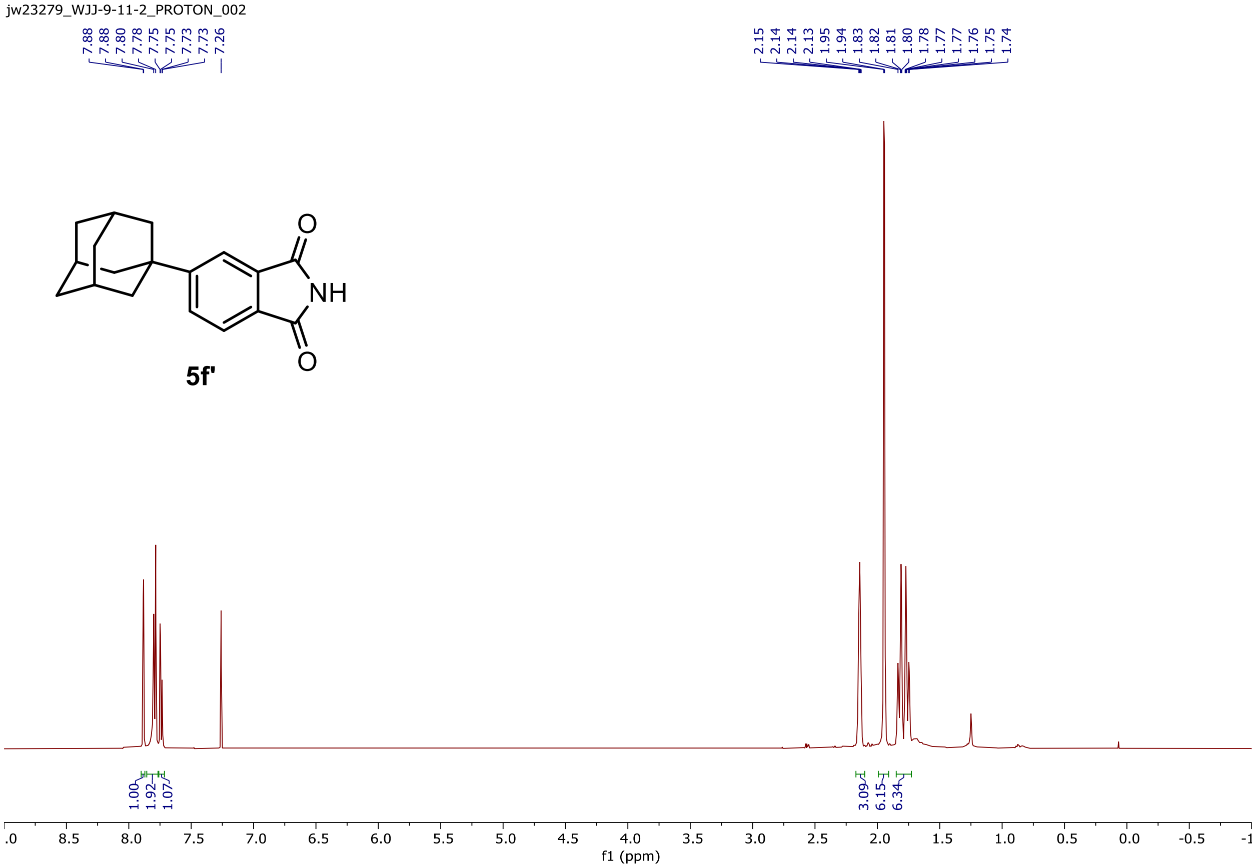

^{13}C NMR (125 MHz, $CDCl_3$) of **5f'**

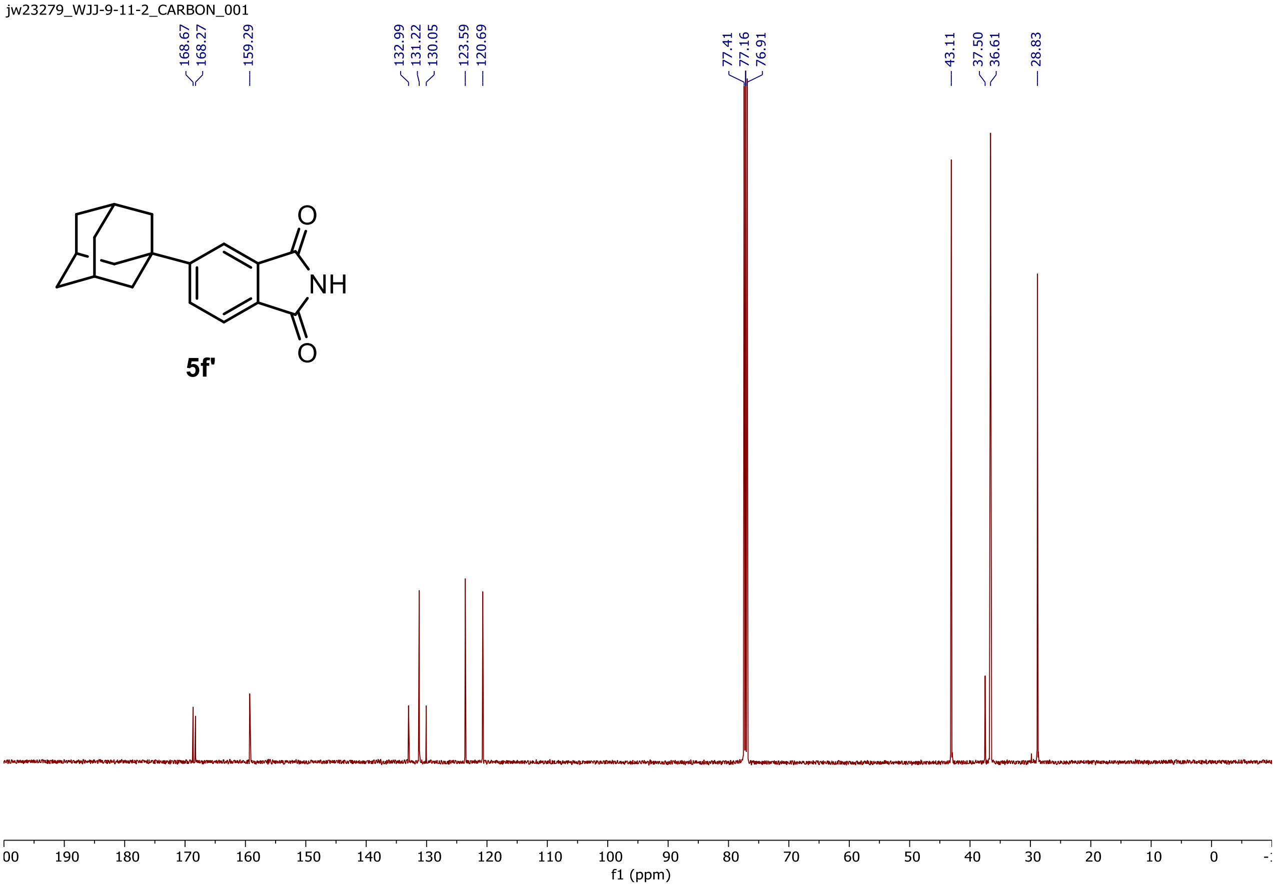

^{1}H NMR (400 MHz, $CDCl_3$) of **5g** (*see procedure*)

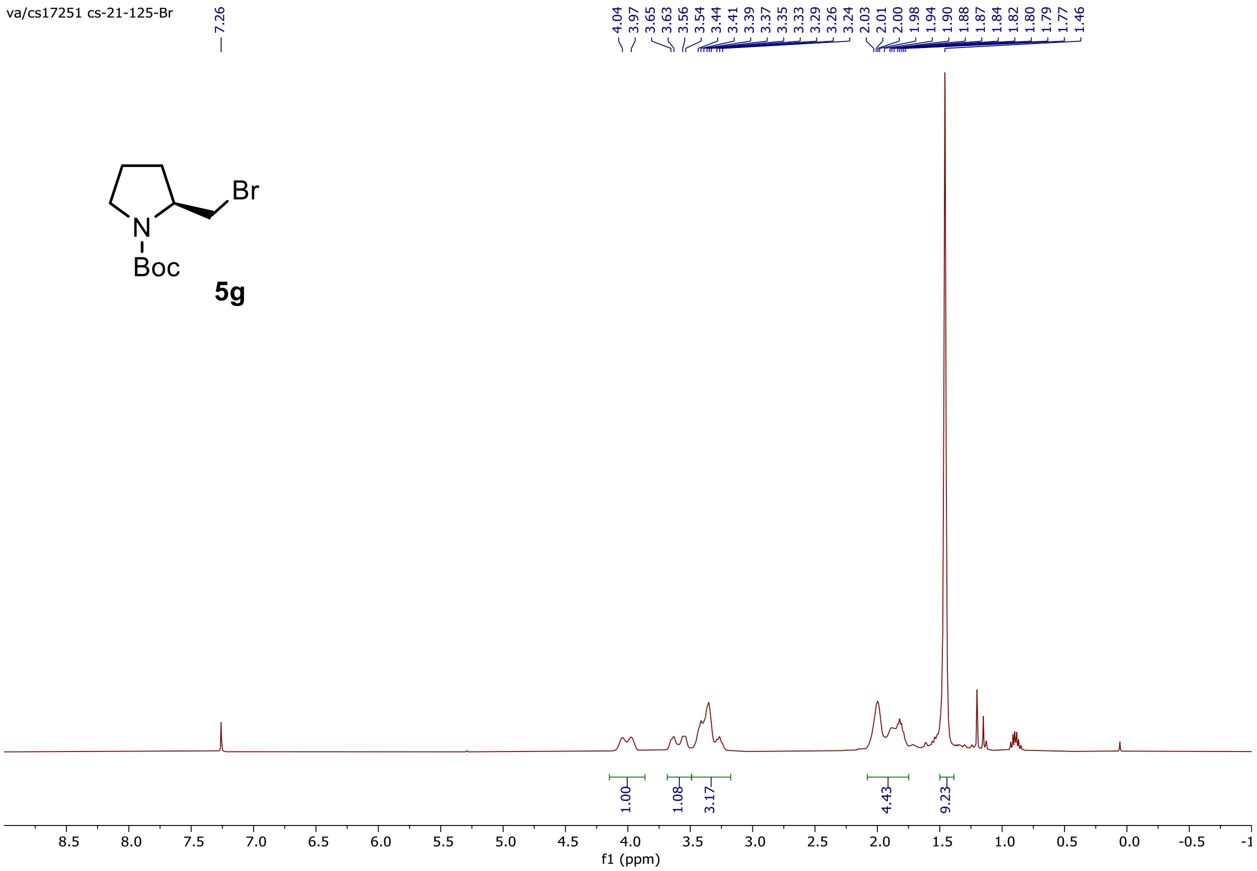

^{13}C NMR (100 MHz, $CDCl_3$) of **5g**

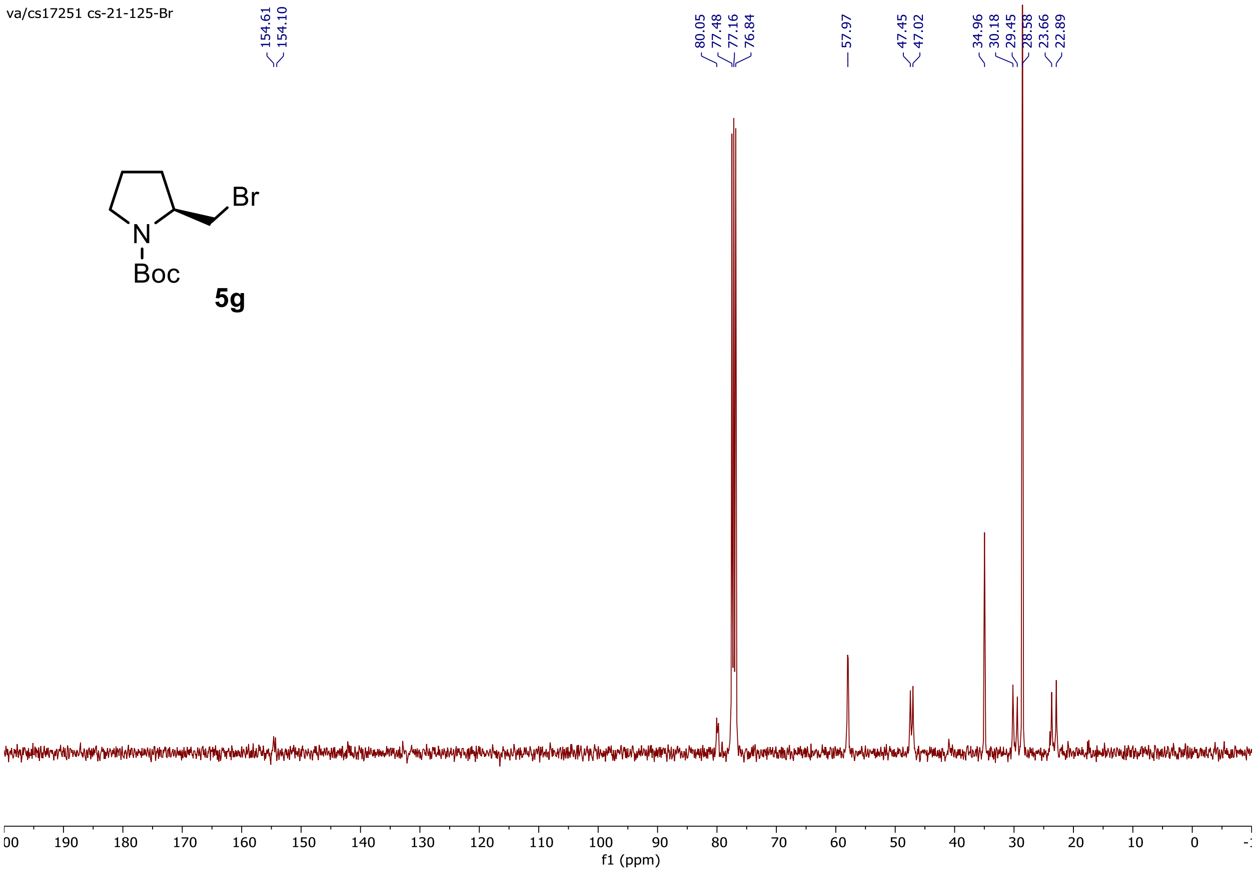

^{1}H NMR (400 MHz, $CDCl_3$) of **5h** (*see procedure*)

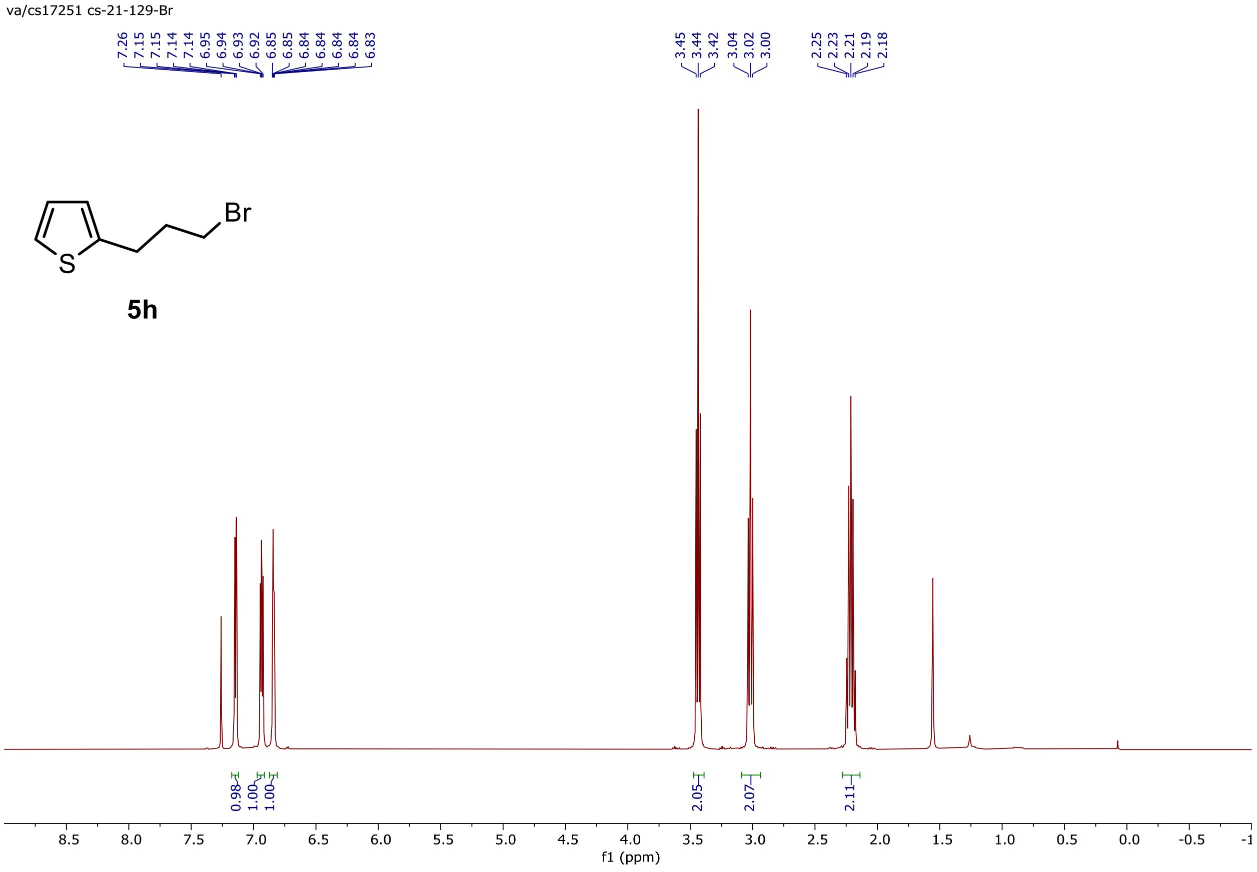

^{13}C NMR (100 MHz, $CDCl_3$) of **5h**

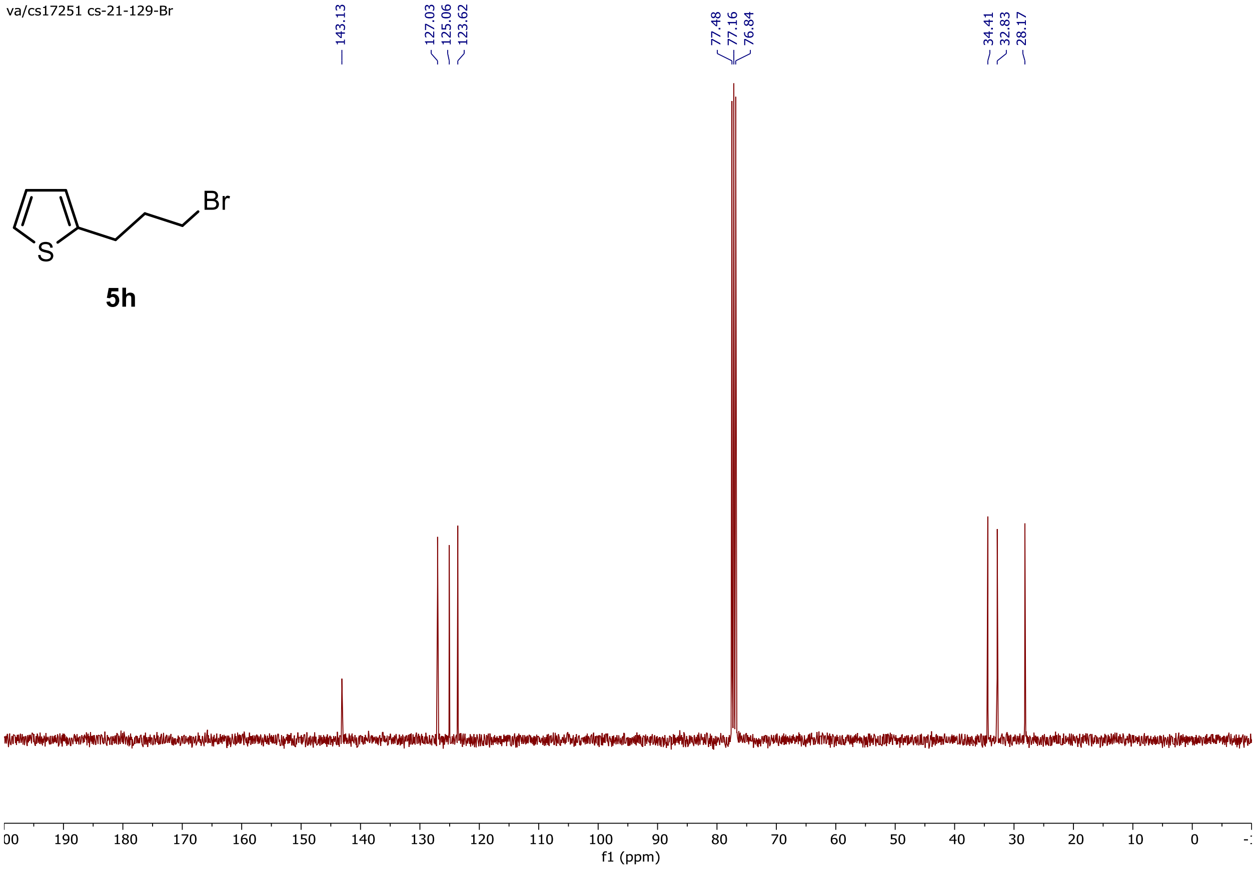

^{1}H NMR (400 MHz, $CDCl_3$) of **5i** (*see procedure*)

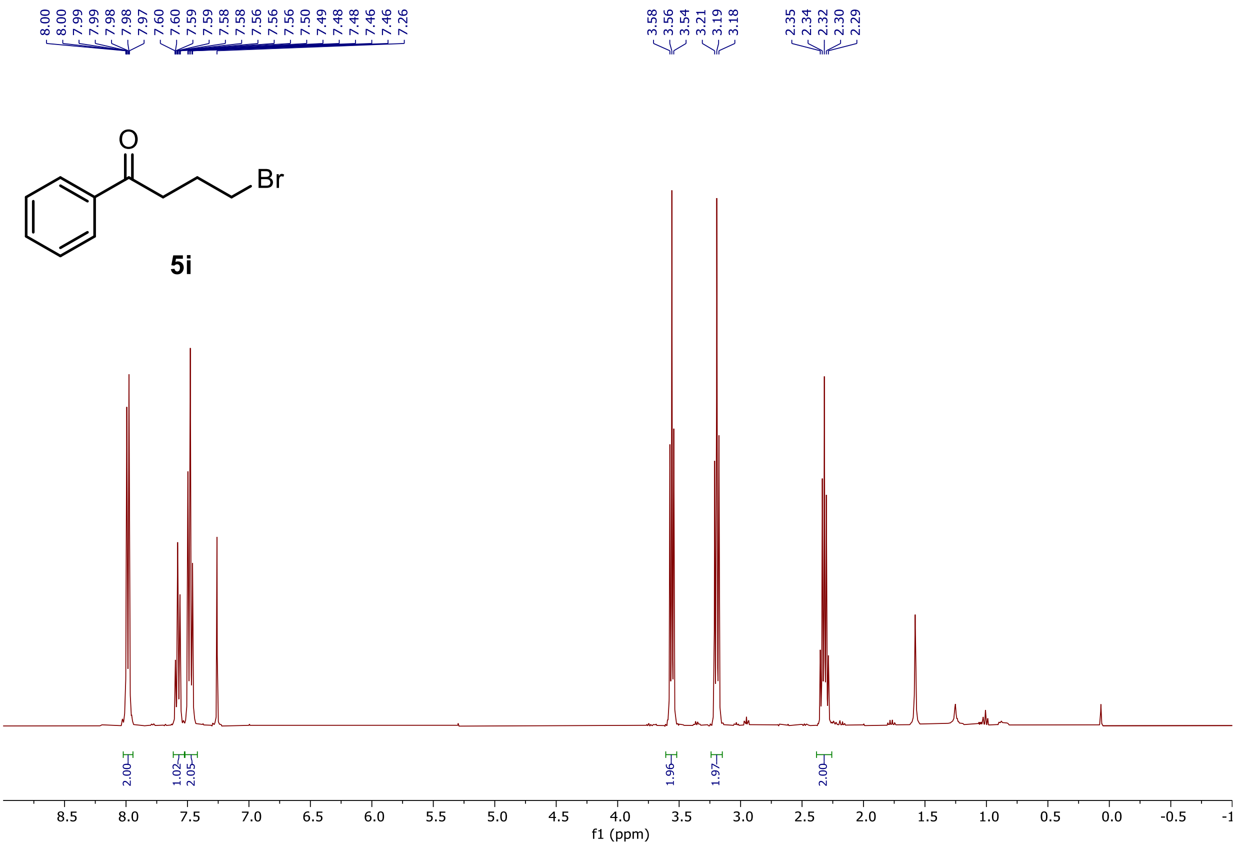

^{13}C NMR (100 MHz, $CDCl_3$) of **5i**

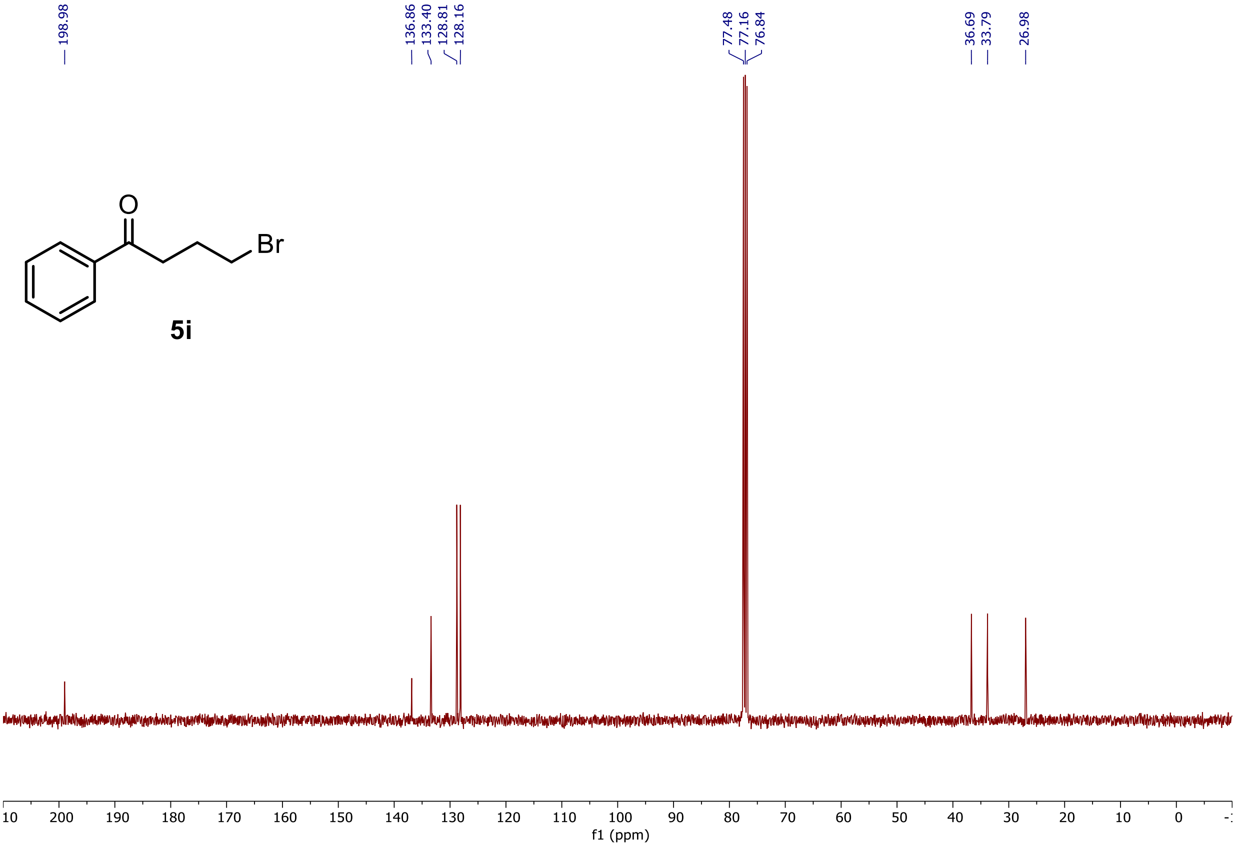

^{1}H NMR (500 MHz, $CDCl_3$) of **5j** (*see procedure*)

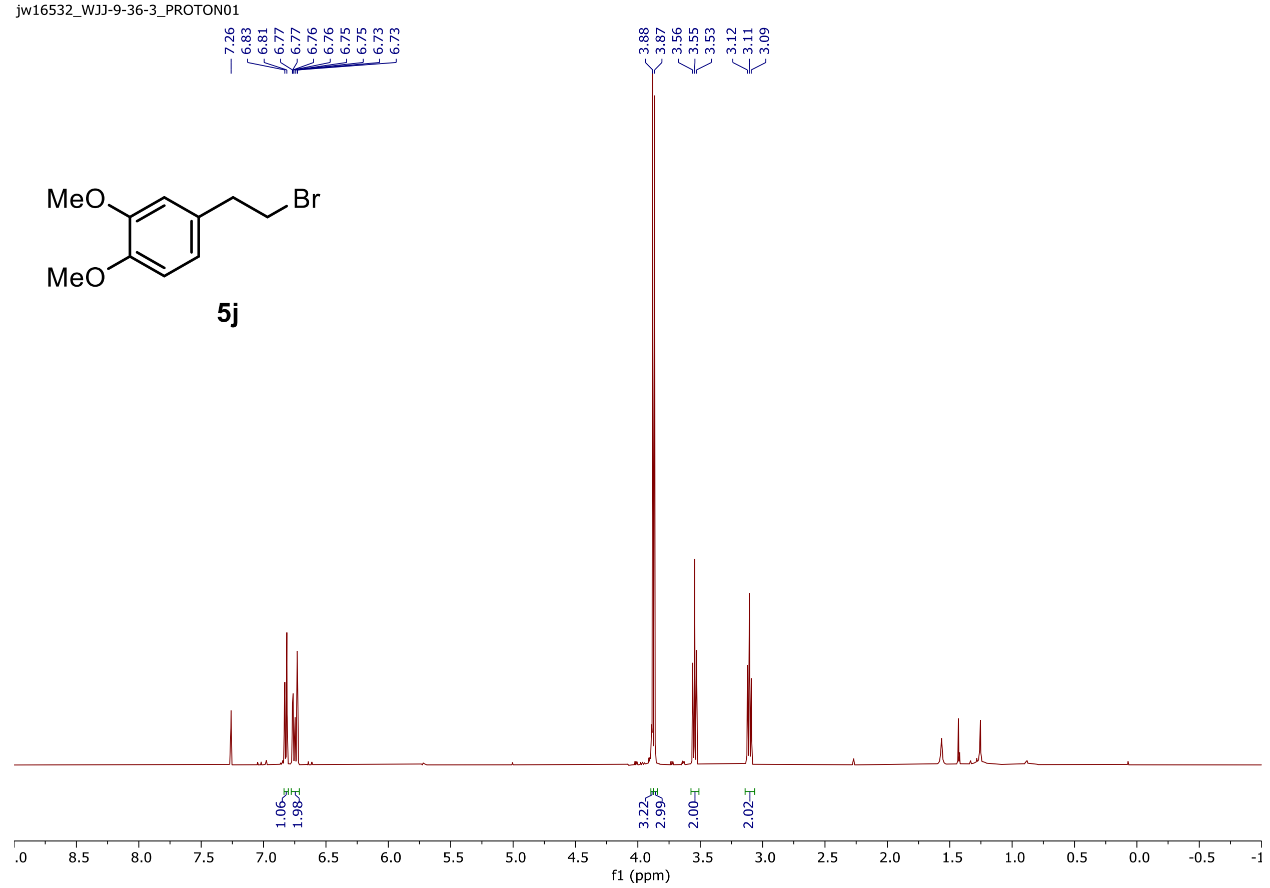

^{13}C NMR (125 MHz, $CDCl_3$) of **5j**

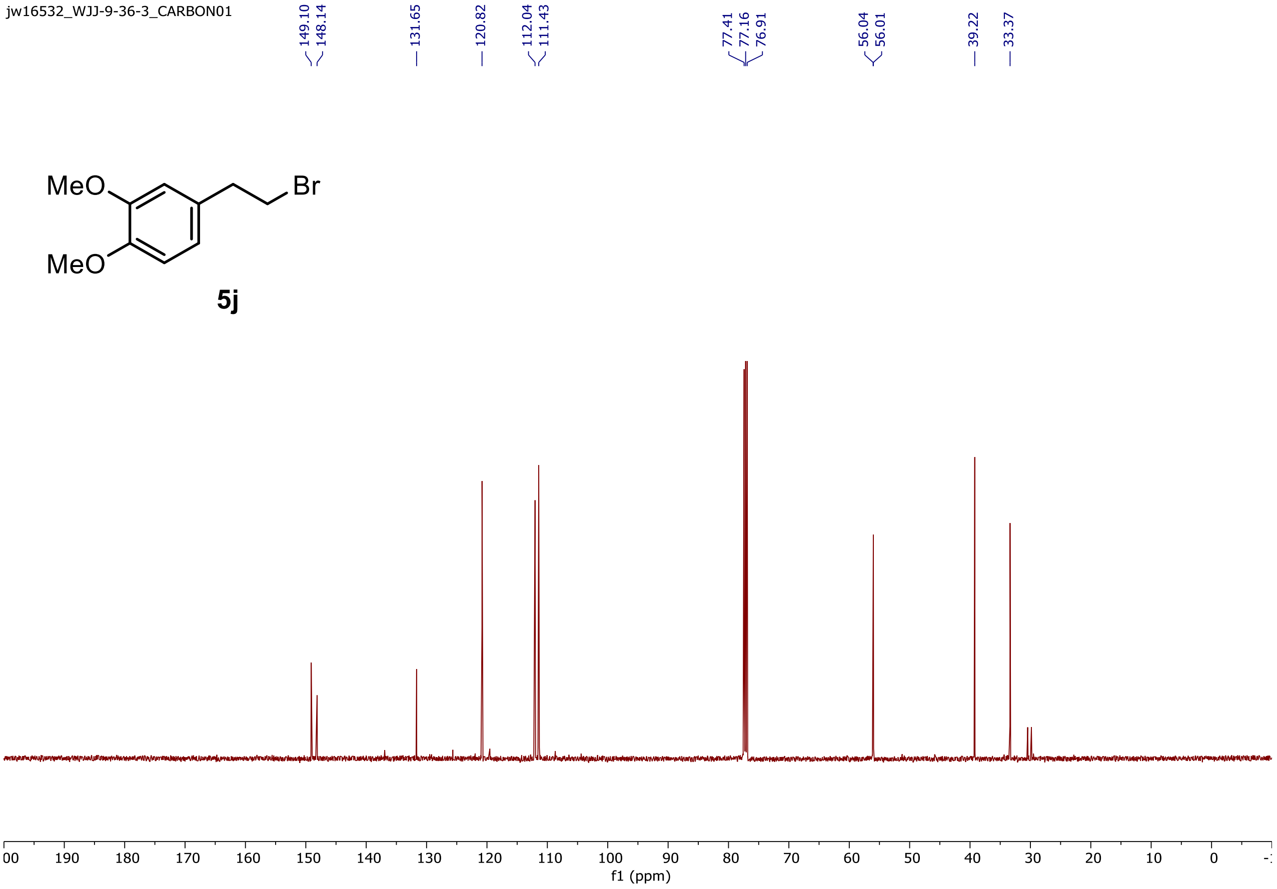

^{1}H NMR (500 MHz, $CDCl_3$) of **9j** (*see procedure*)

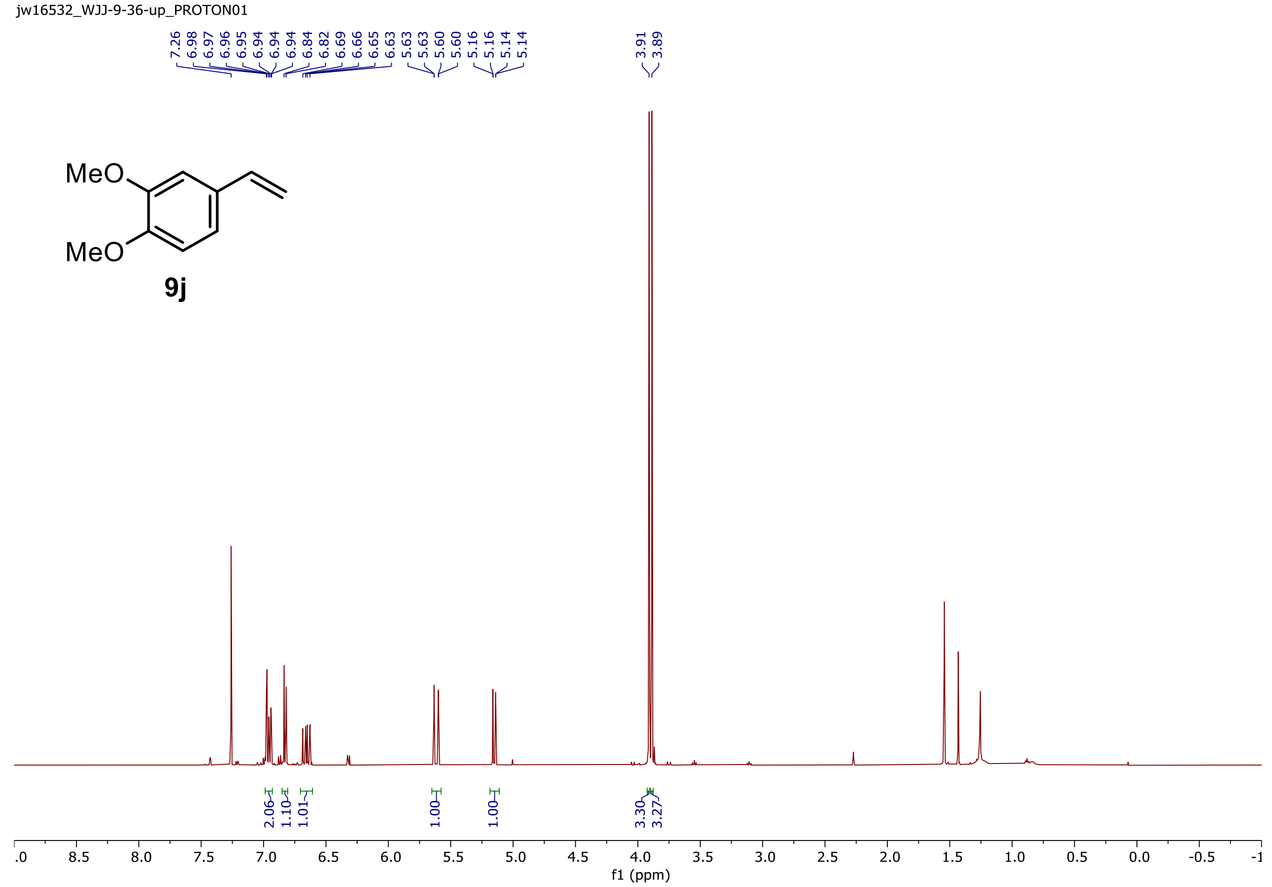

^{13}C NMR (125 MHz, $CDCl_3$) of **9j**

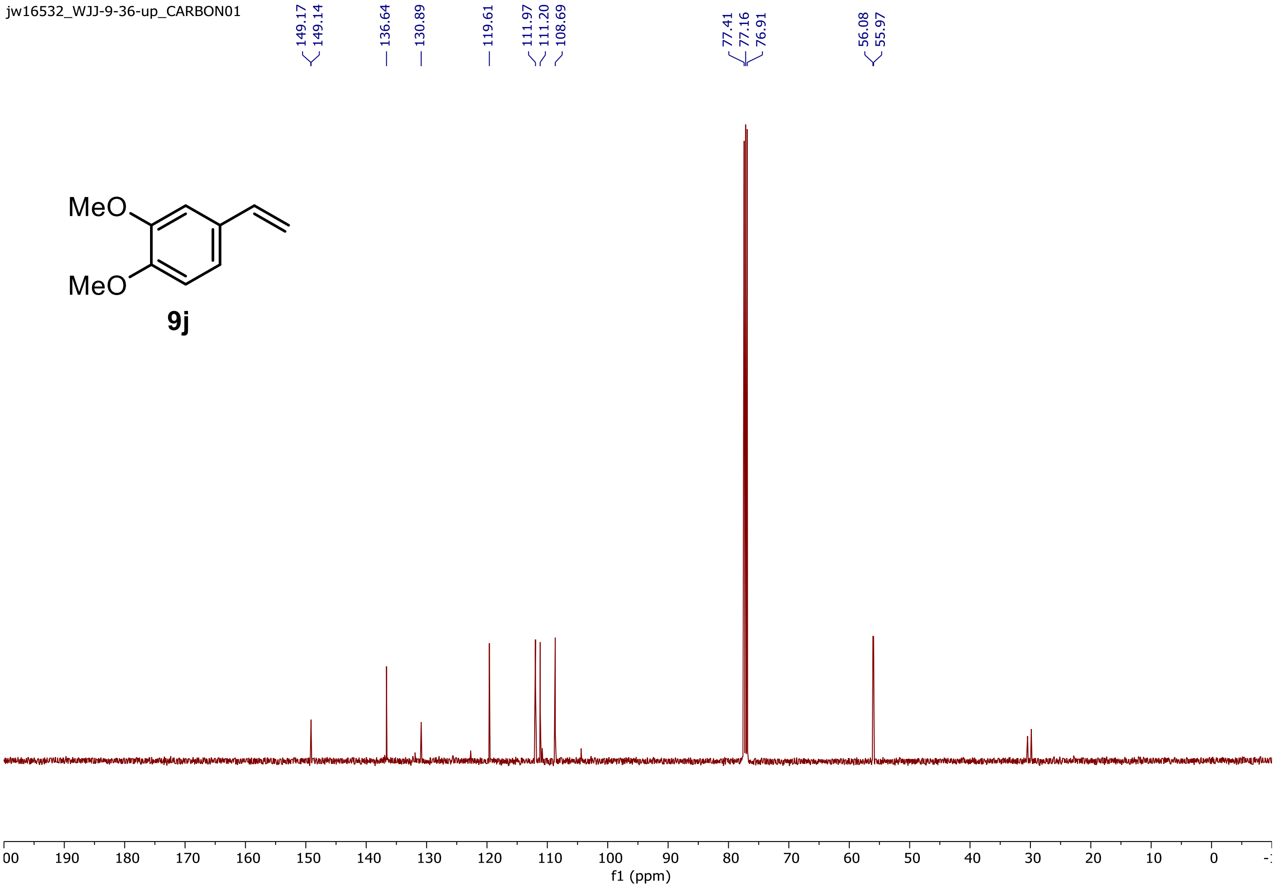

^{1}H NMR (500 MHz, $CDCl_3$) of **5k** (*see procedure*)

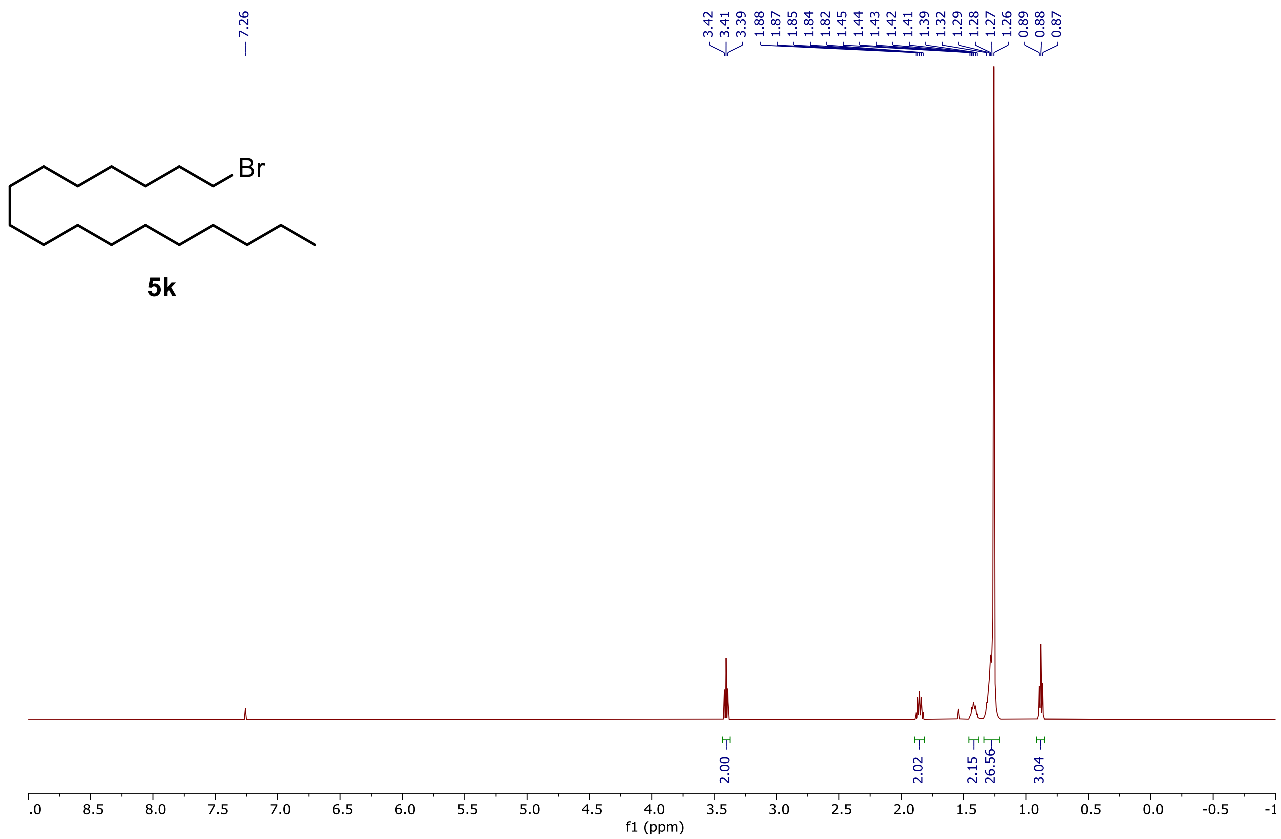

^{13}C NMR (125 MHz, $CDCl_3$) of **5k**

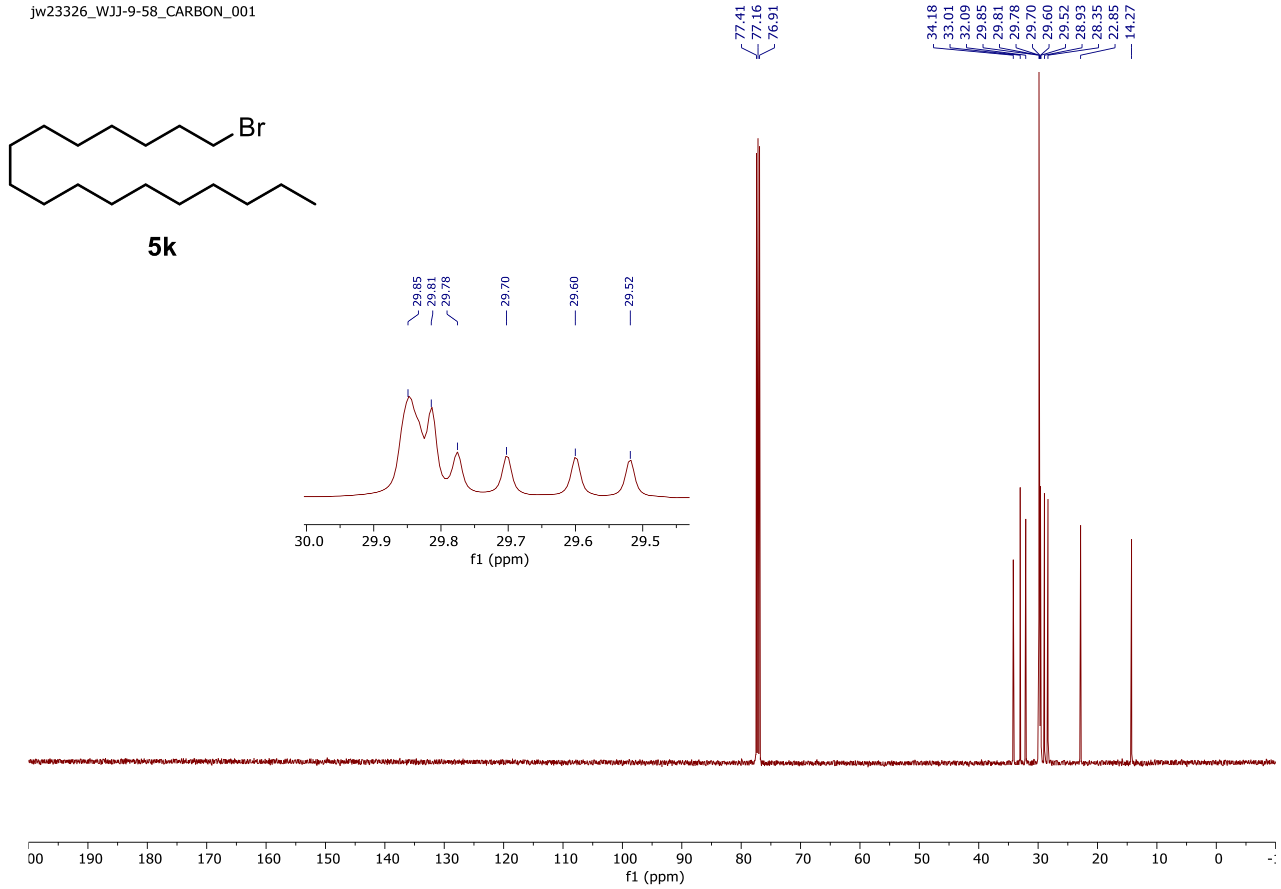

^{1}H NMR (500 MHz, $CDCl_3$) of **5l** (*see procedure*)

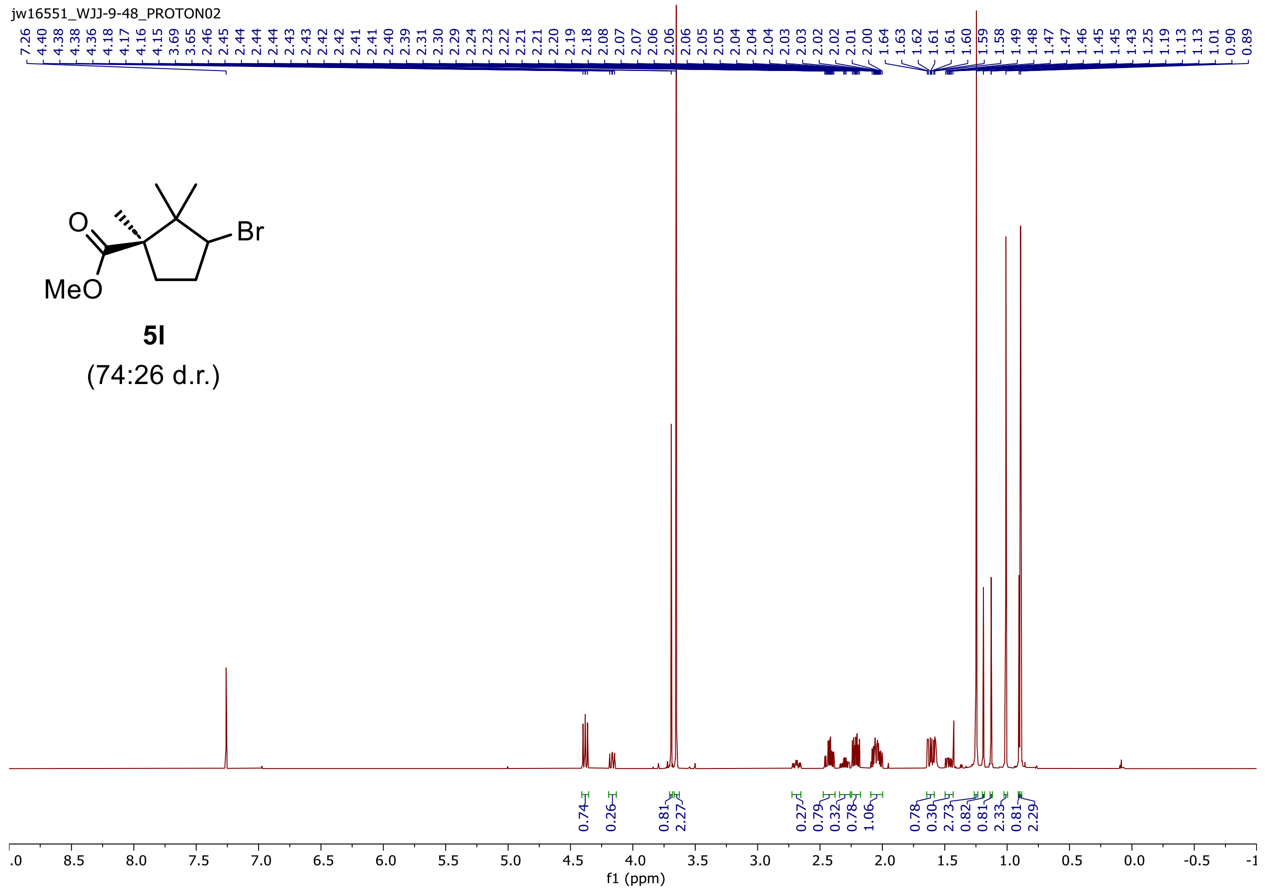

^{13}C NMR (125 MHz, $CDCl_3$) of **5l**

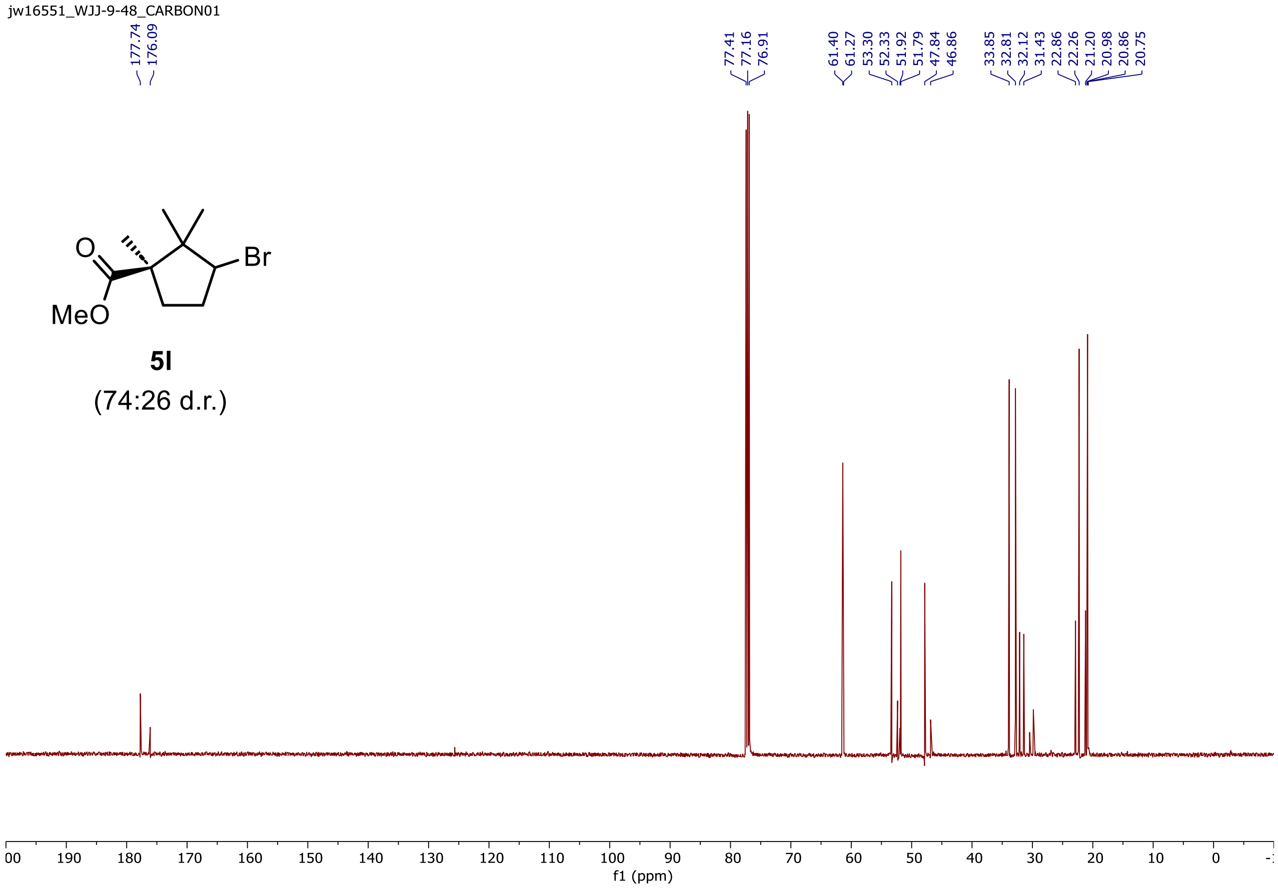

^{1}H NMR (500 MHz, $CDCl_3$) of **5m** (*see procedure*)

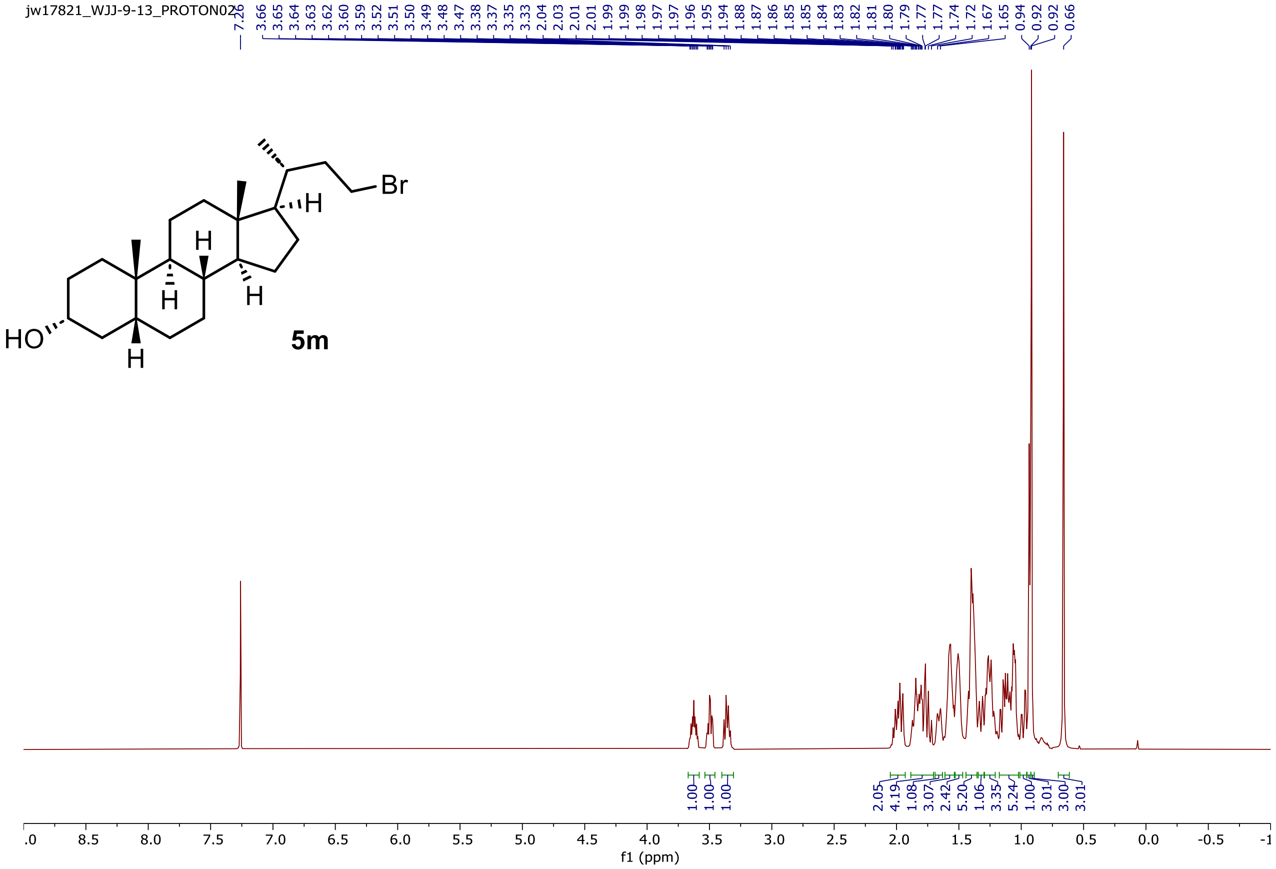

^{13}C NMR (125 MHz, $CDCl_3$) of **5m**

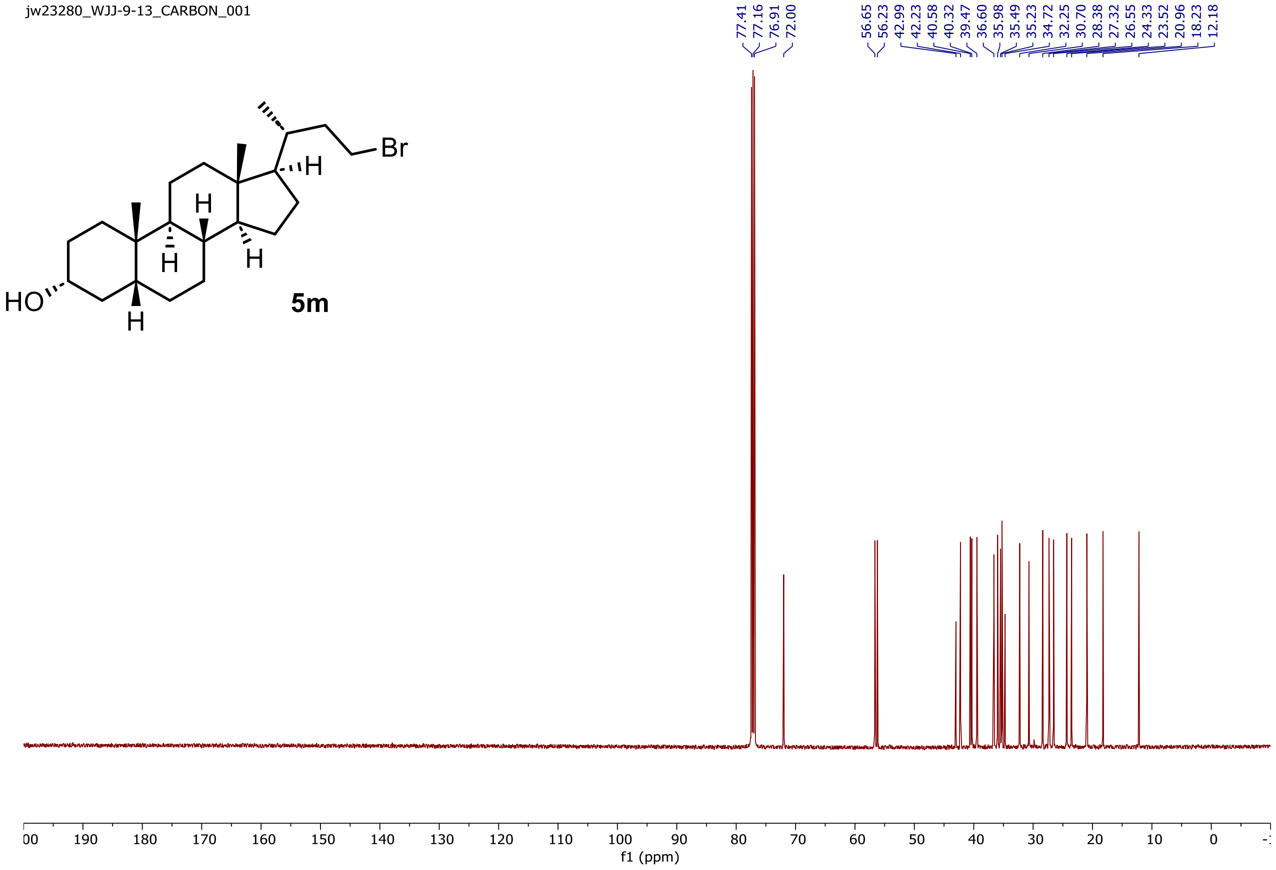

$^{1}H–^{1}H$ COSY ($CDCl_3$) of **5m**

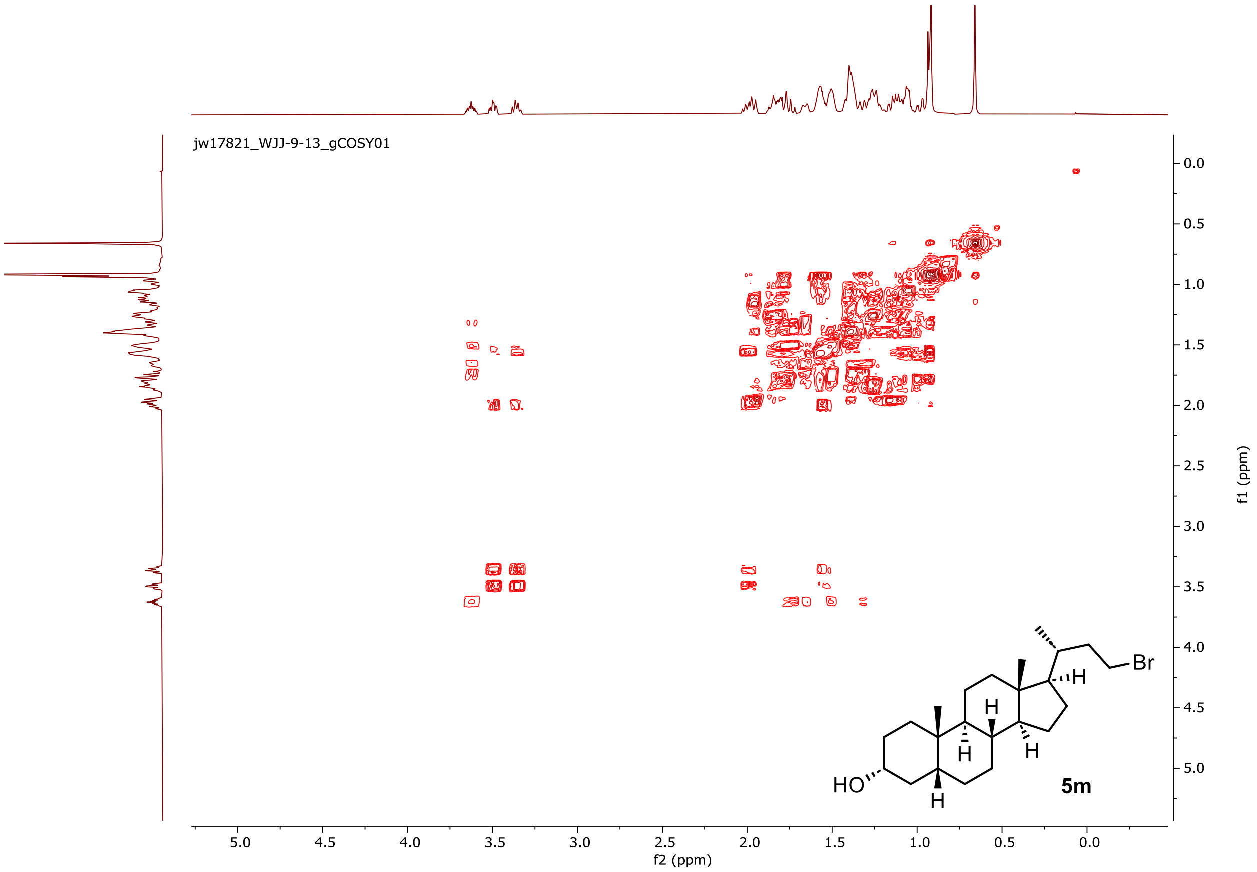

$^{1}H–^{13}C$ HSQC ($CDCl_3$) of **5m**

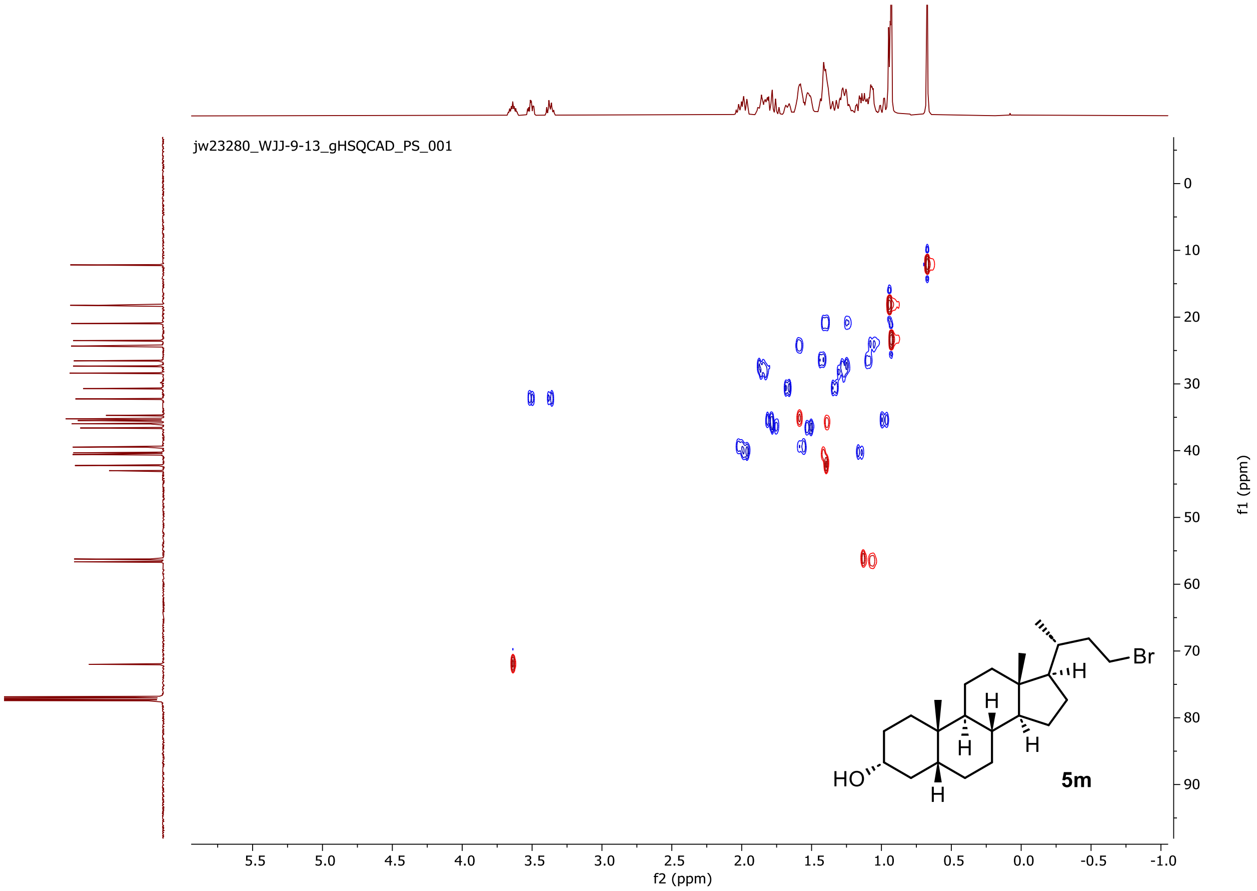

^{1}H NMR (500 MHz, $CDCl_3$) of **5n** (*see procedure*)

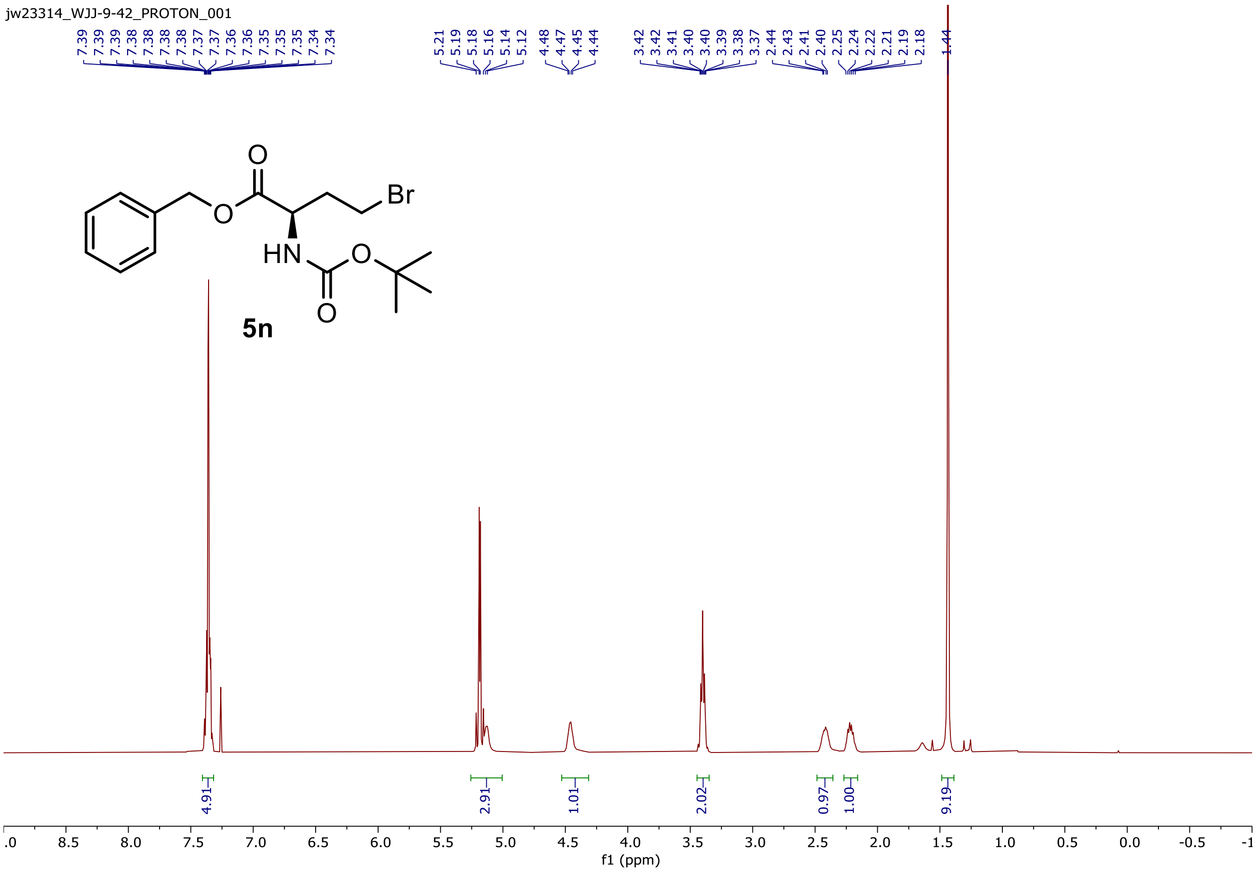

^{13}C NMR (125 MHz, $CDCl_3$) of **5n**

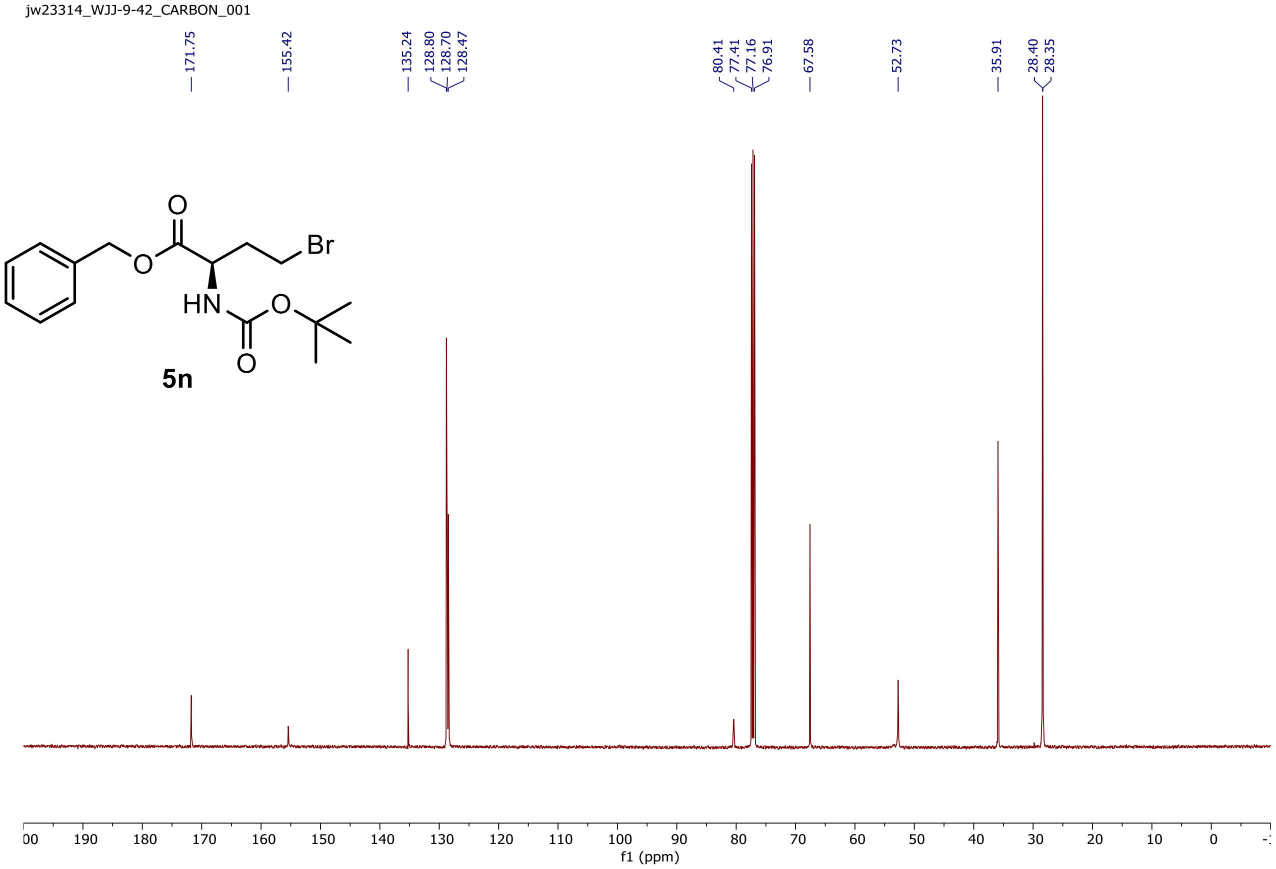

^{1}H NMR (500 MHz, CD_3CN) of **5o** (*see procedure*)

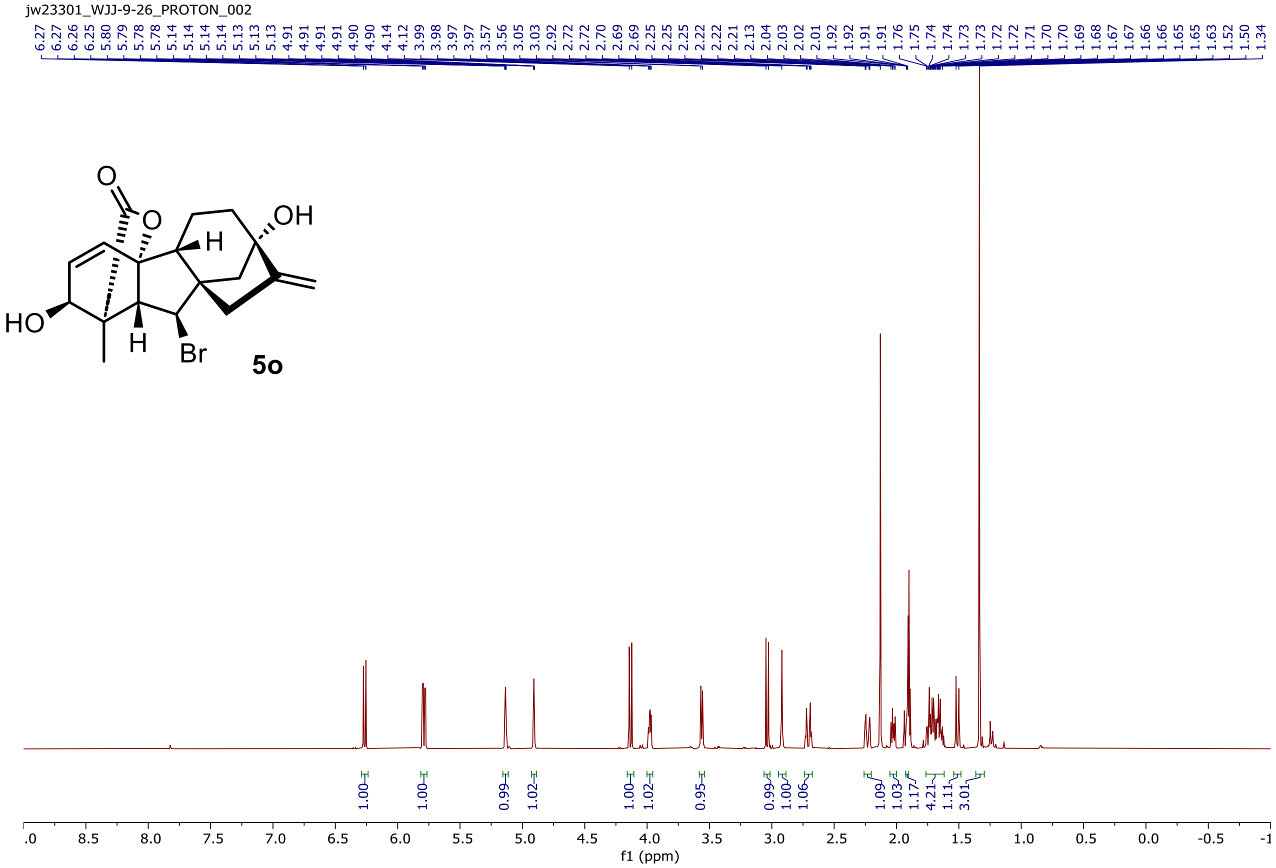

^{13}C NMR (125 MHz, CD_3CN) of **5o**

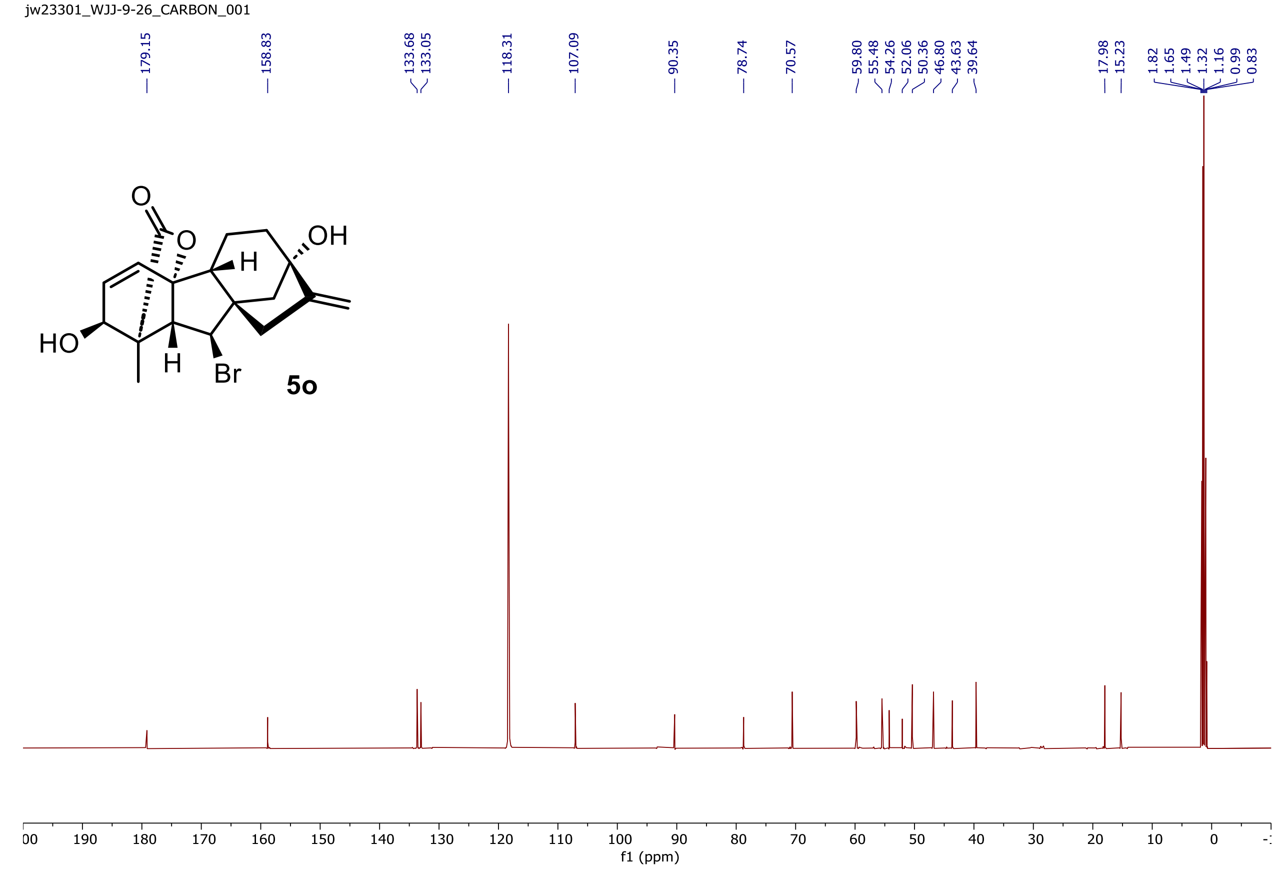

^{1}H–^{1}H COSY (CD_3CN) of **5o**

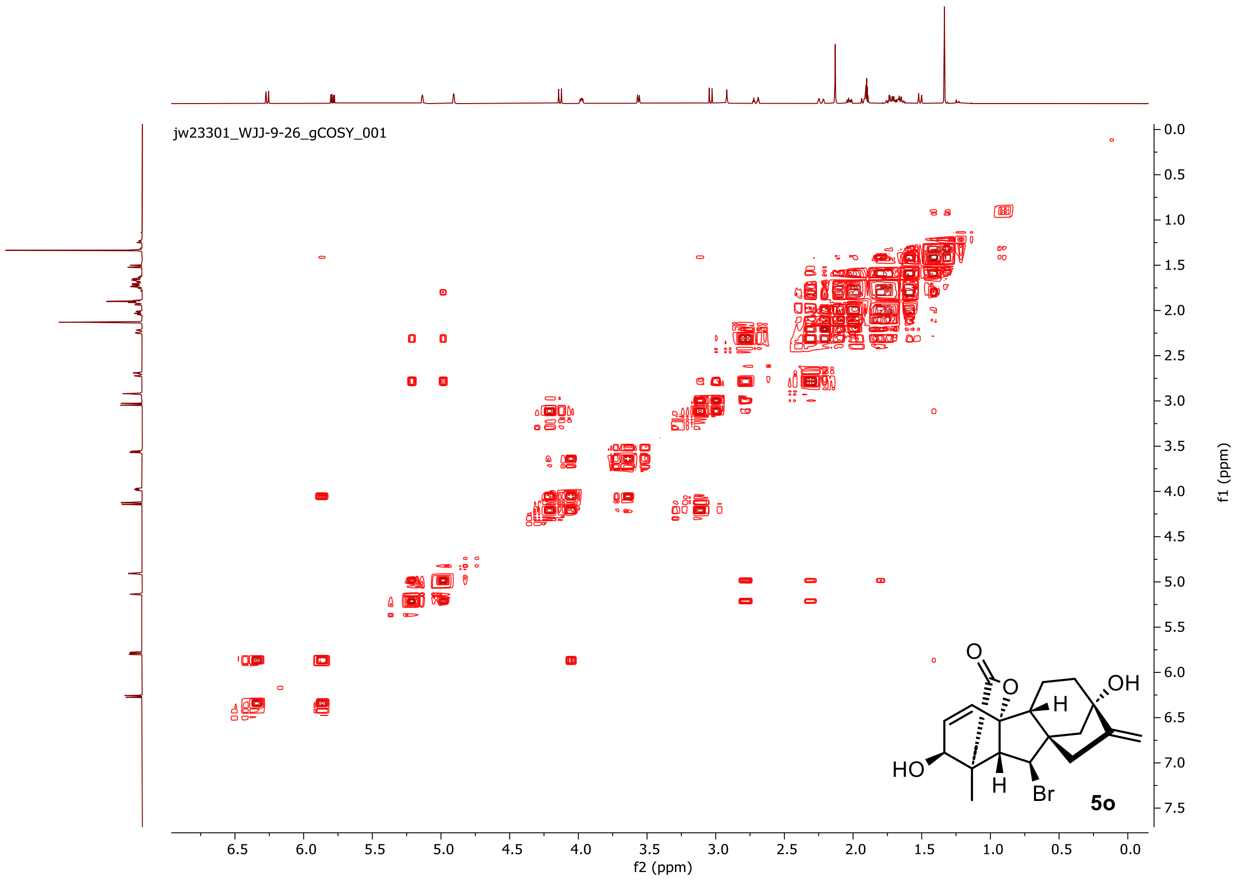

^{1}H–^{13}C HSQC (CD_3CN) of **5o**

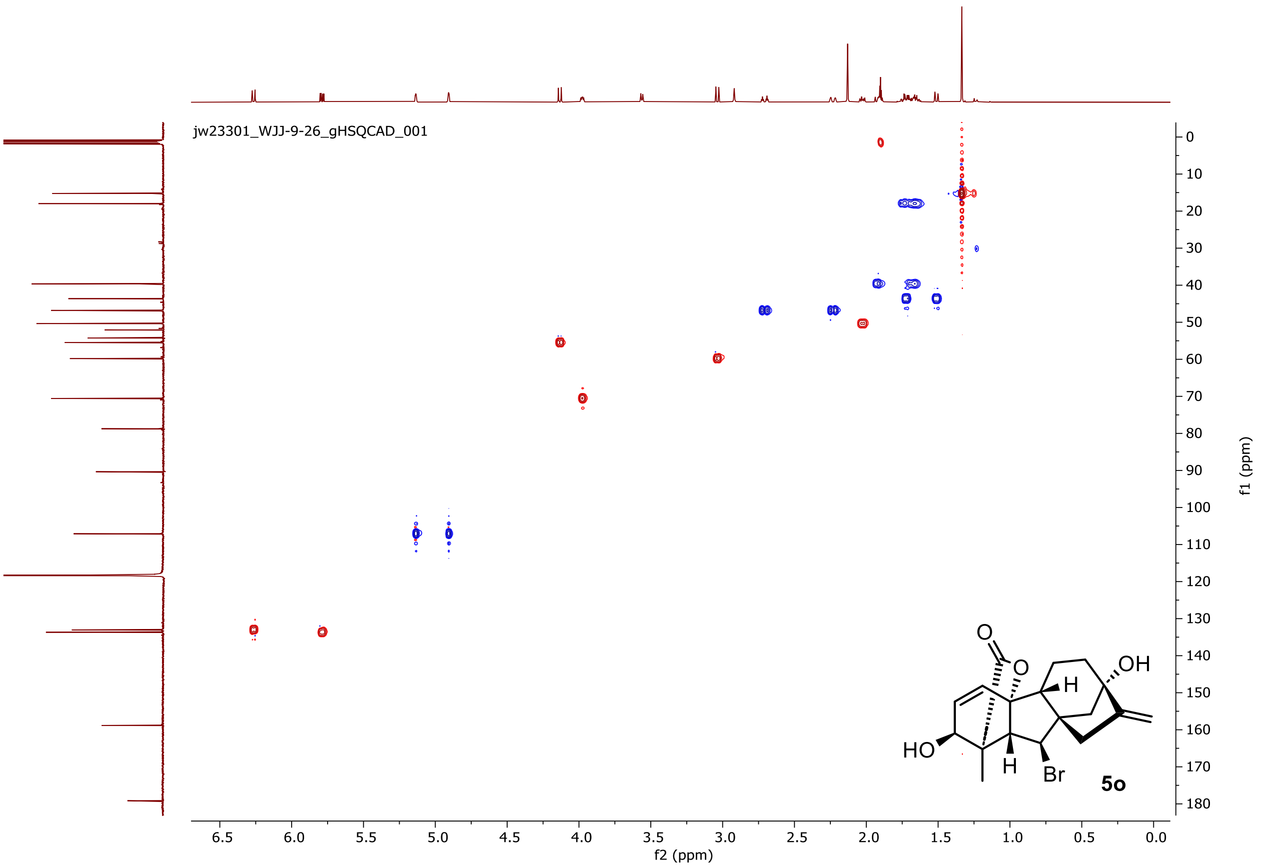

^{1}H NMR (500 MHz, $CDCl_3$) of **5p** (*see procedure*)

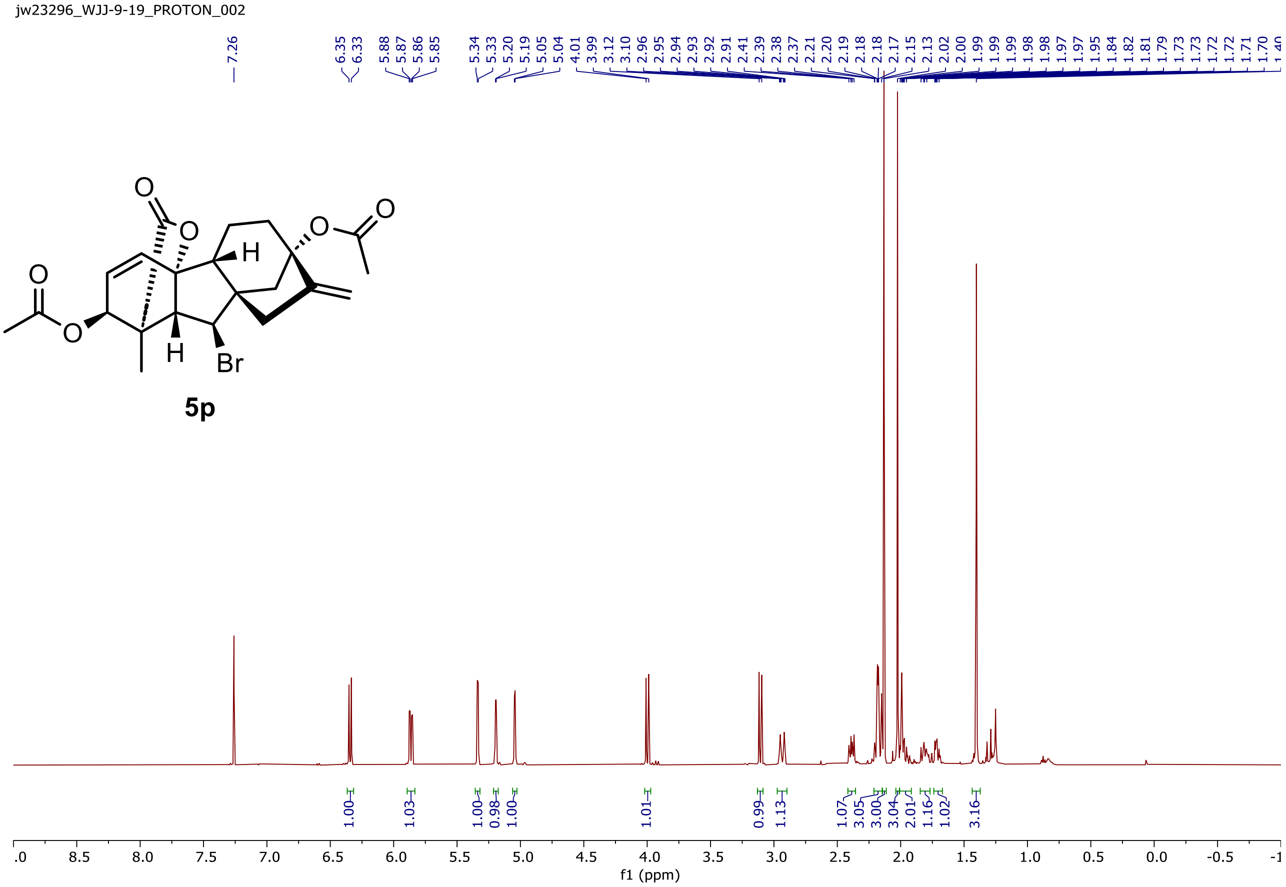

^{13}C NMR (125 MHz, $CDCl_3$) of **5p**

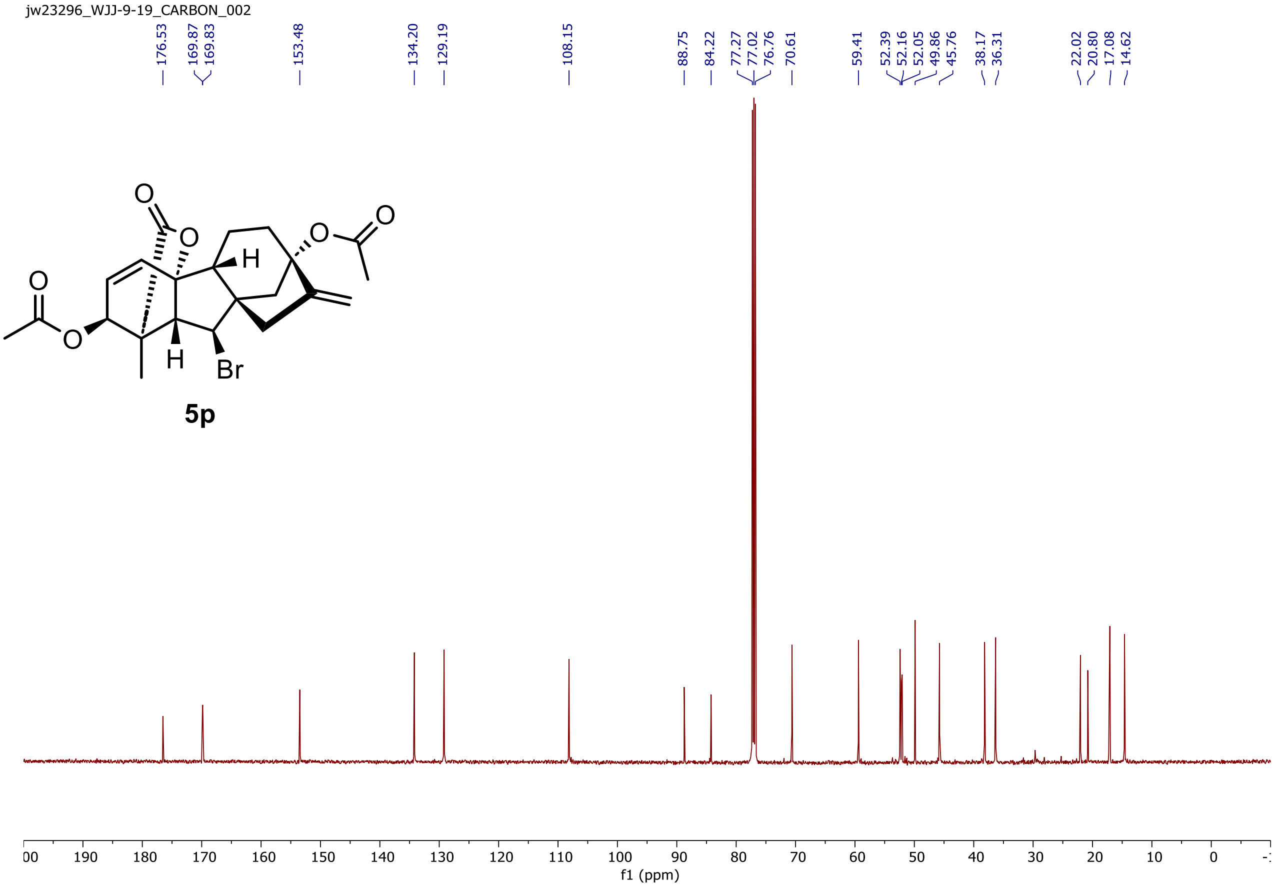

^1H–^1H COSY ($CDCl_3$) of **5p**

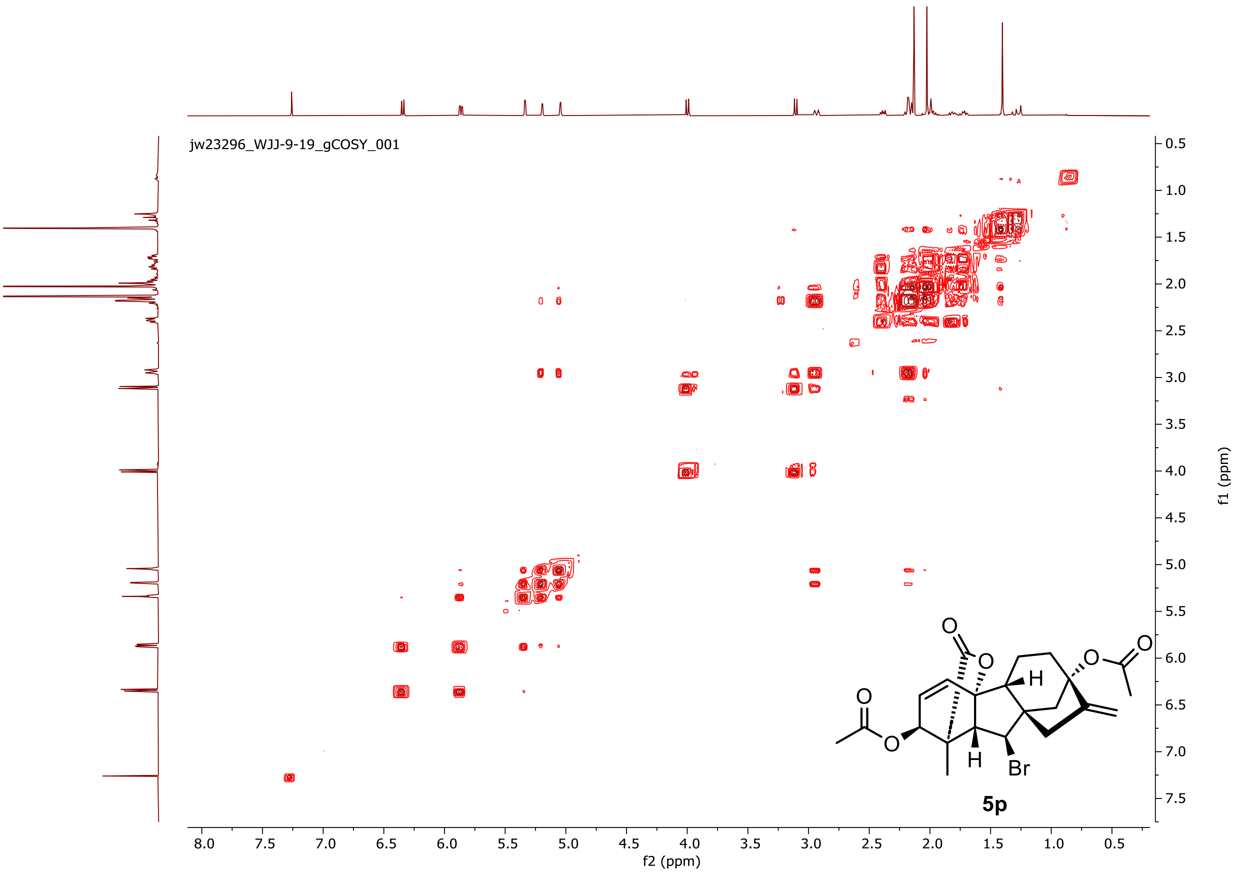

^1H–^{13}C HSQC ($CDCl_3$) of **5p**

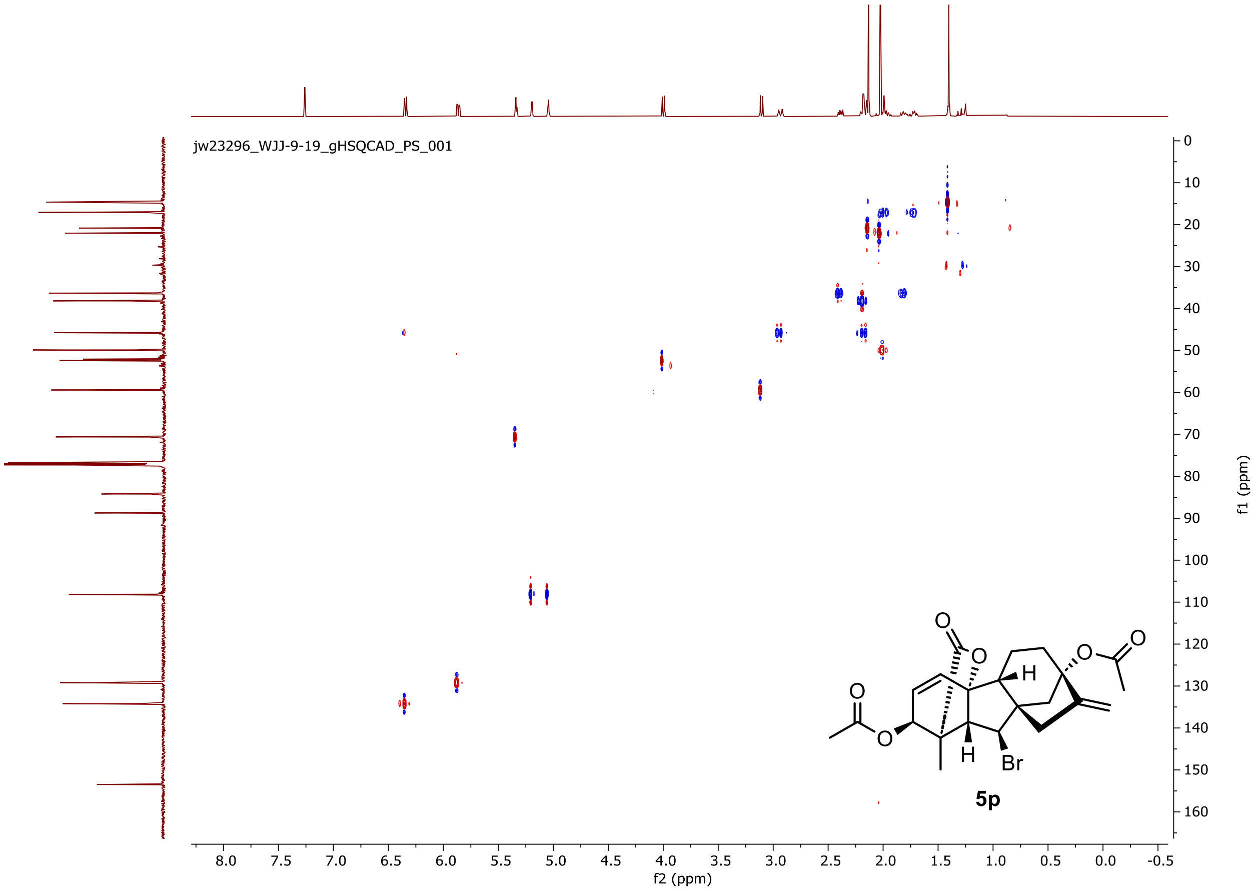

^{1}H NMR (500 MHz, $CDCl_3$) of **5p'** (*see procedure*)

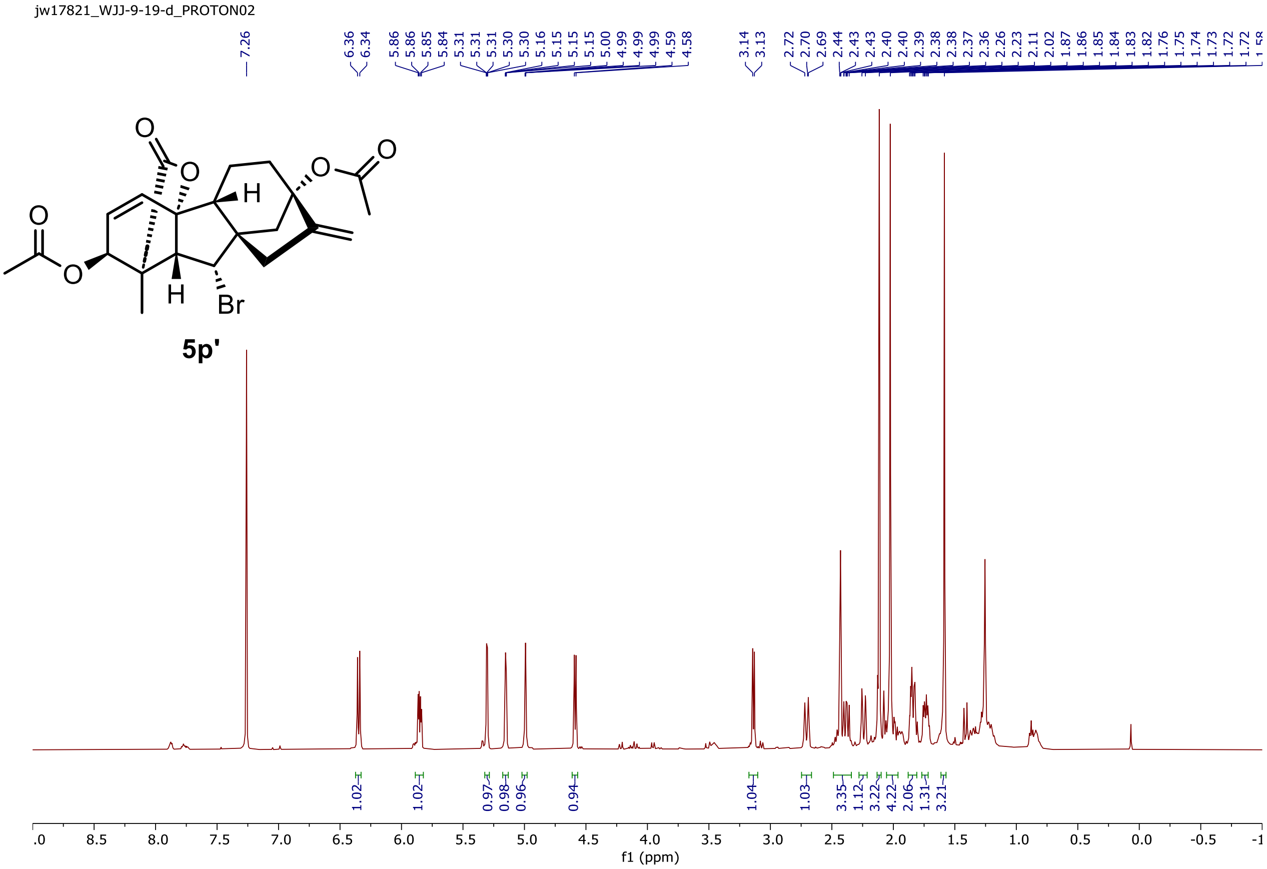

^{13}C NMR (125 MHz, $CDCl_3$) of **5p'**

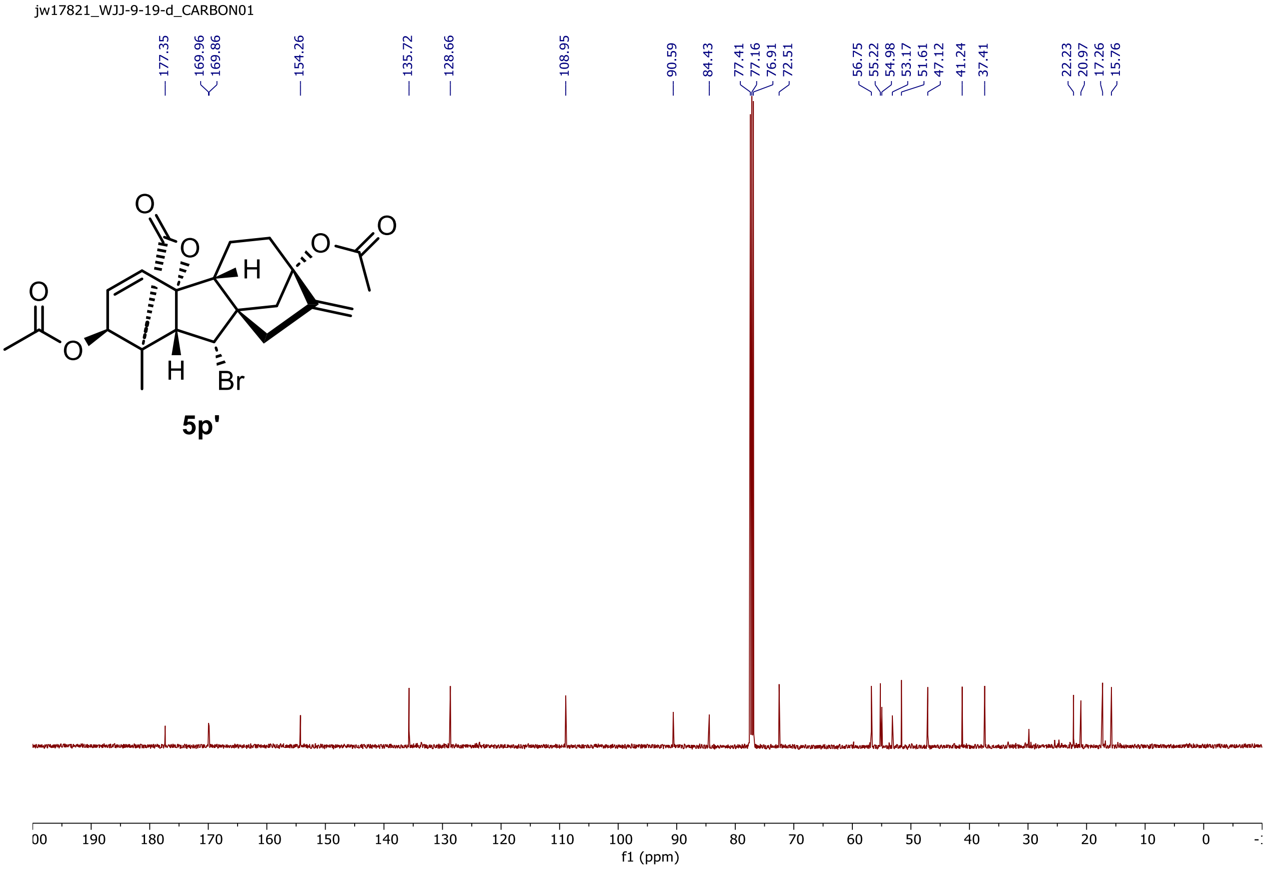

^{1}H–^{1}H COSY ($CDCl_3$) of **5p'**

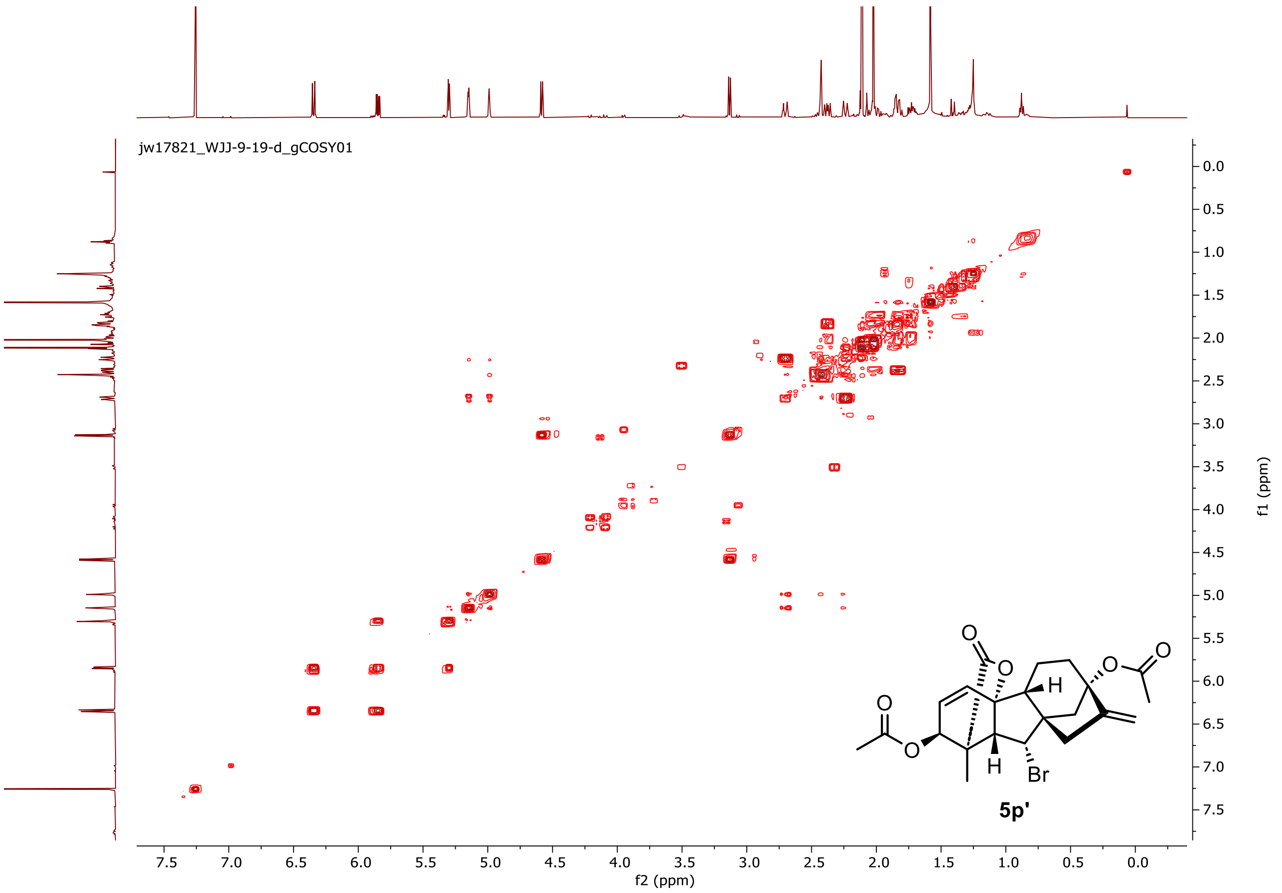

^{1}H–^{13}C HSQC ($CDCl_3$) of **5p'**

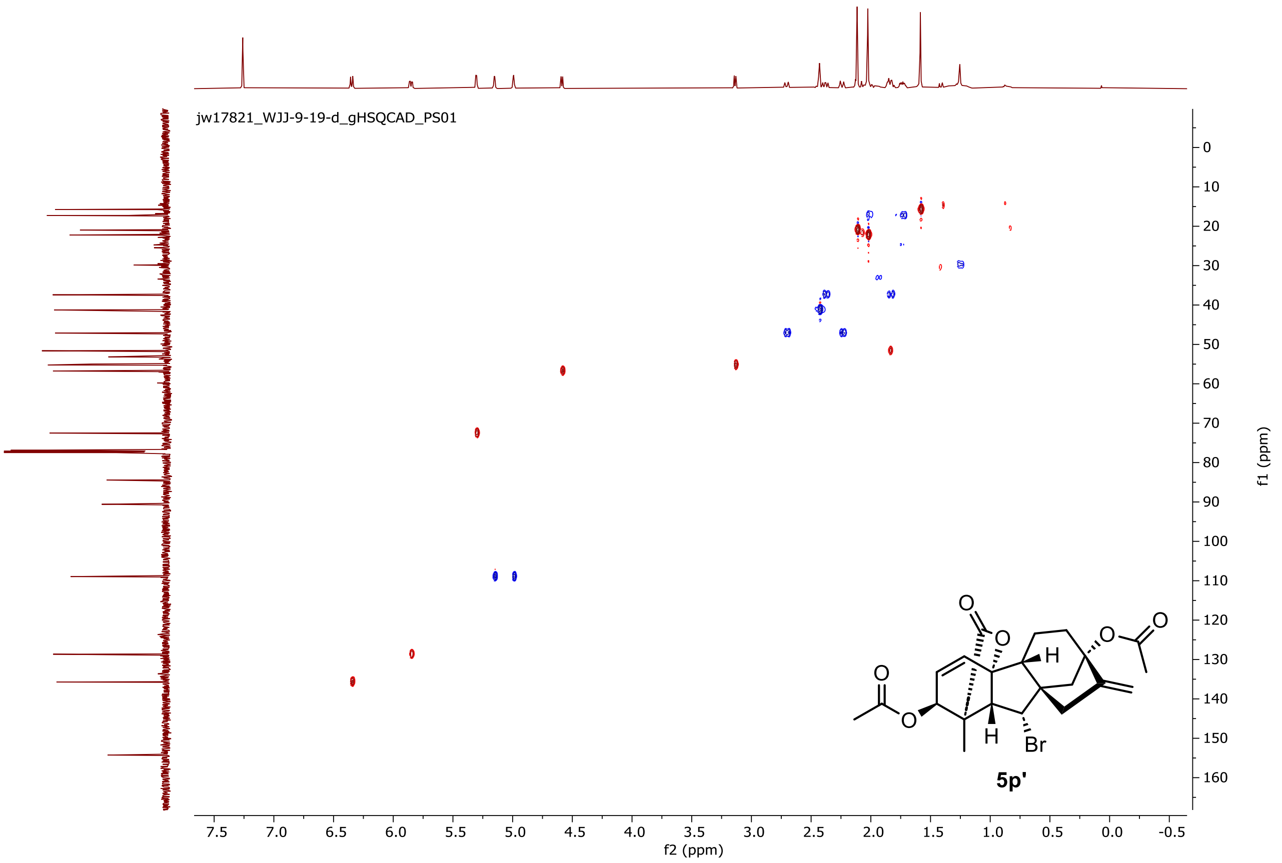

^{1}H NMR (400 MHz, $CDCl_3$) of **6a** (*see procedure*)

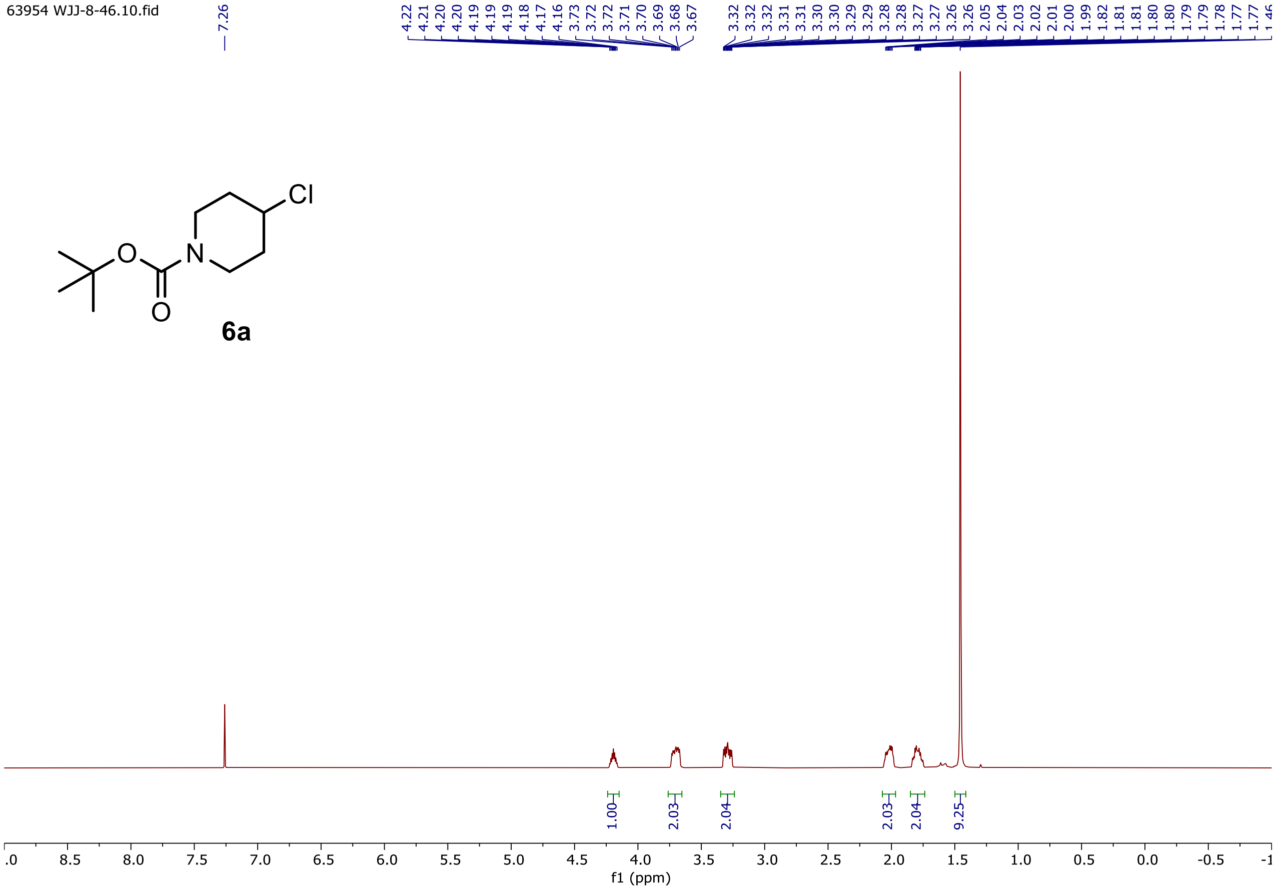

^{13}C NMR (100 MHz, $CDCl_3$) of **6a**

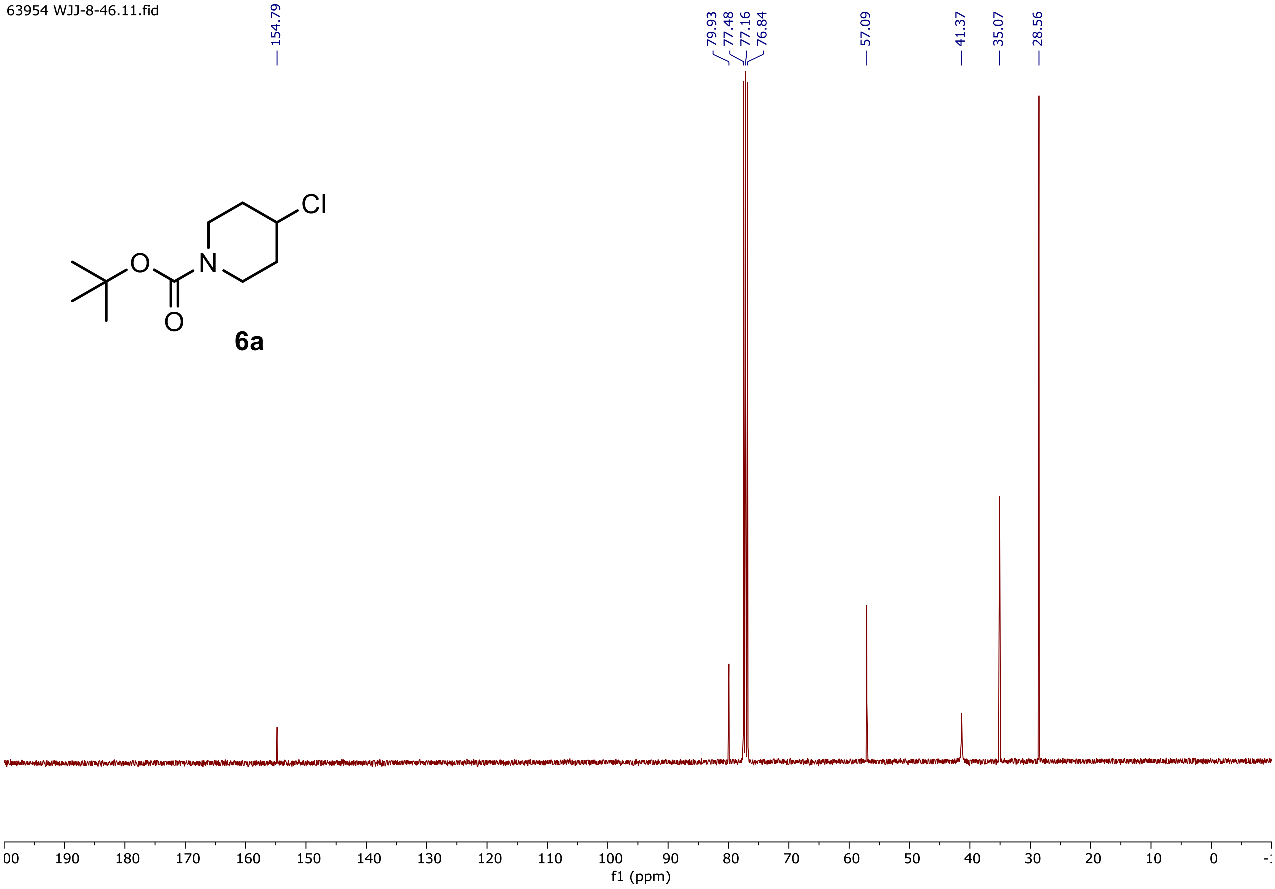

^{1}H NMR (500 MHz, $CDCl_3$) of **6b** (*see procedure*)

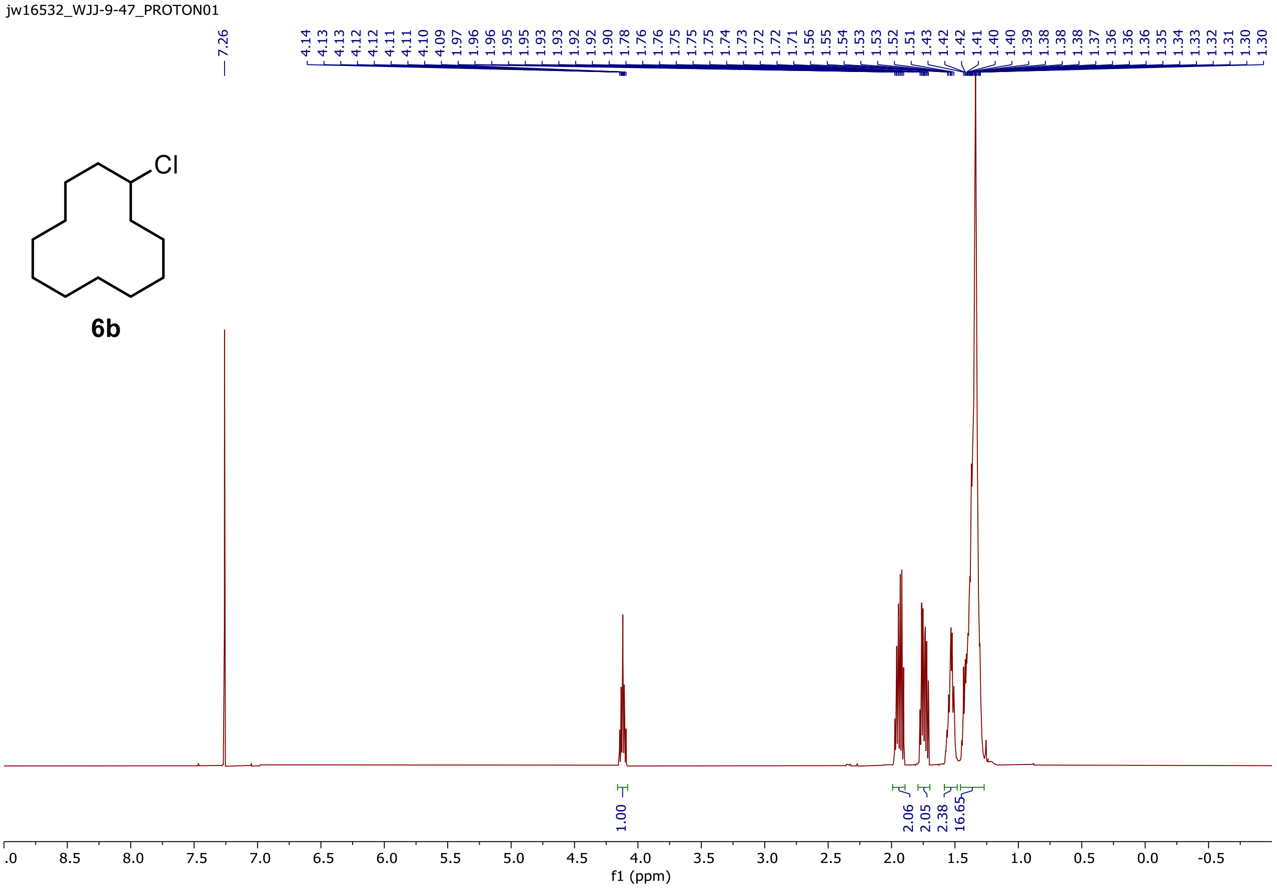

^{13}C NMR (125 MHz, $CDCl_3$) of **6b**

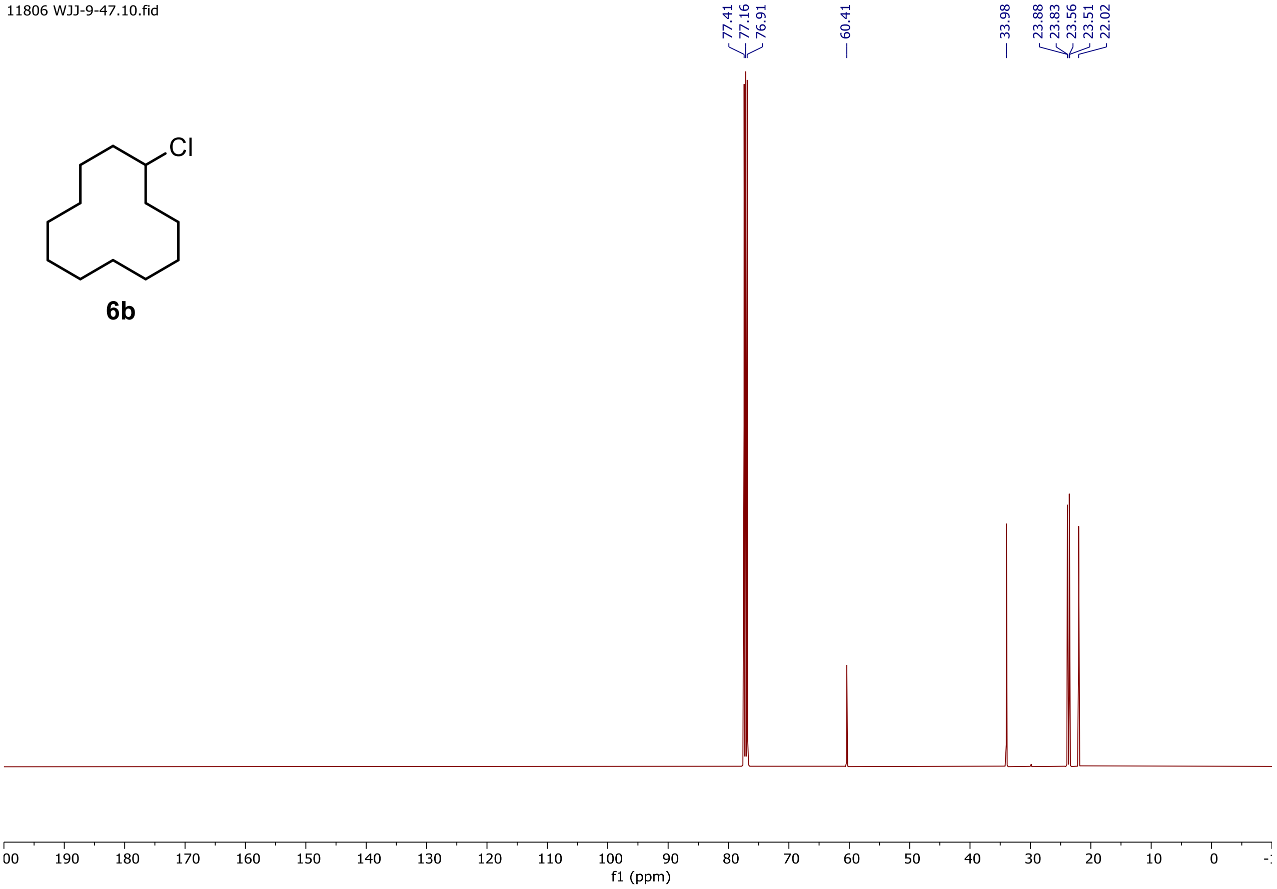

^{1}H NMR (500 MHz, $CDCl_3$) of **6c** (*see procedure*)

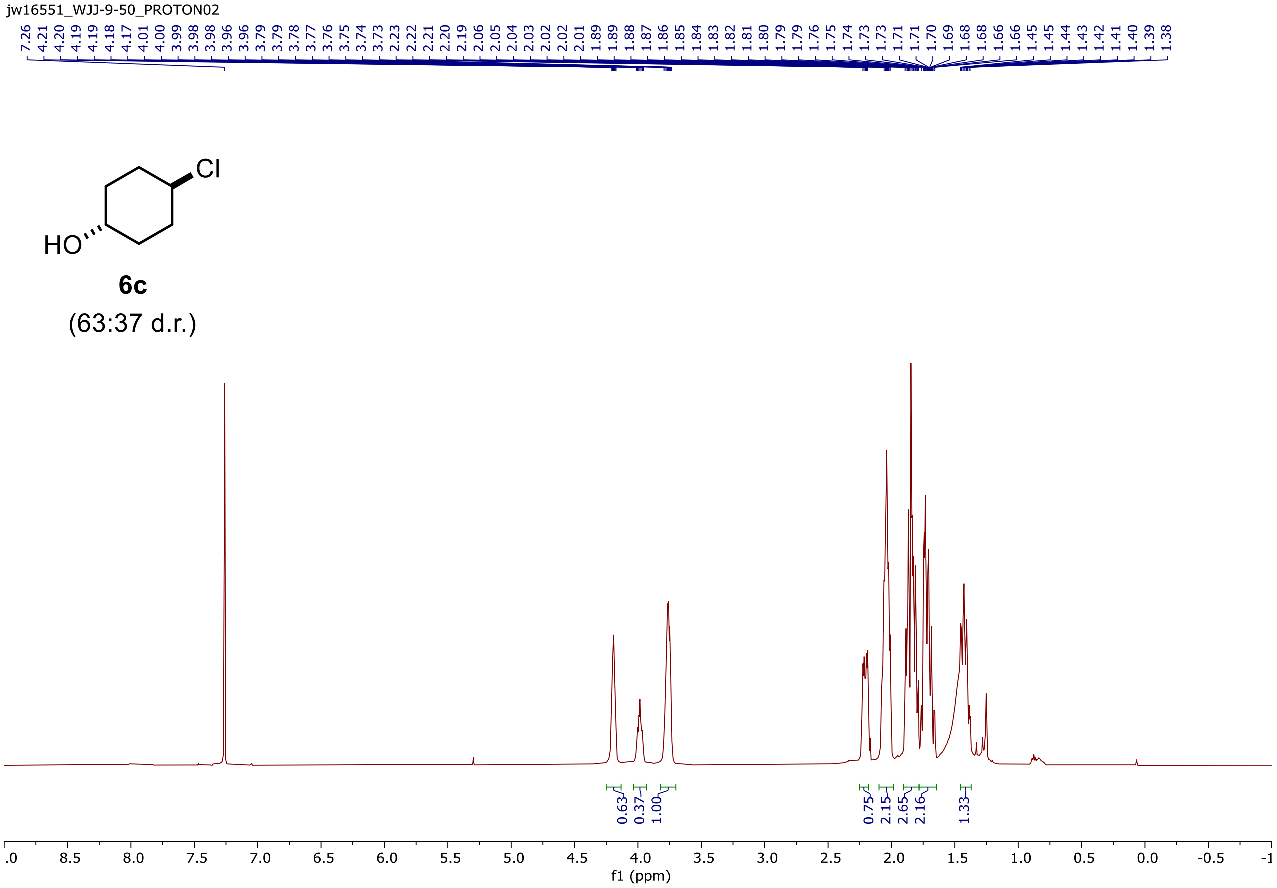

^{13}C NMR (125 MHz, $CDCl_3$) of **6c**

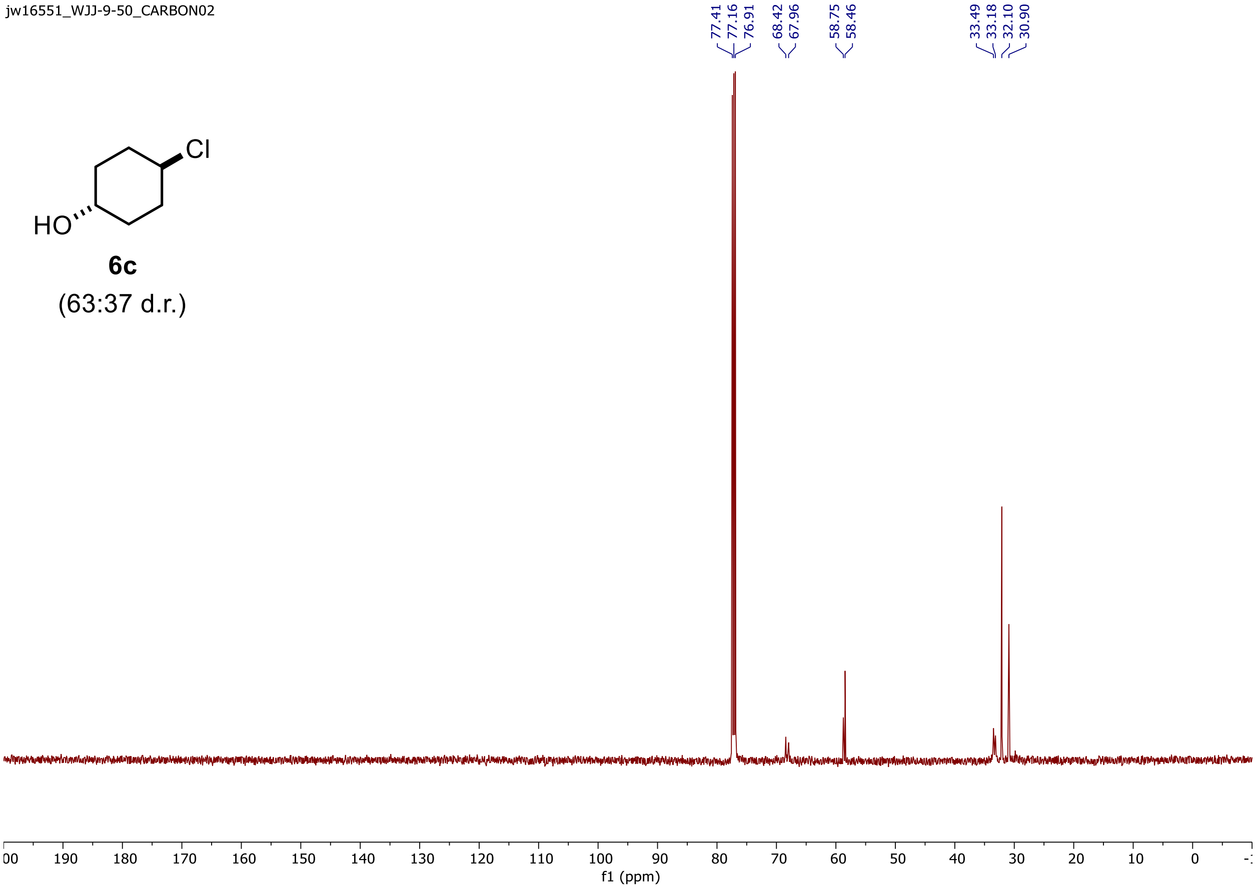

^{1}H NMR (400 MHz, $CDCl_3$) of **6g** (*see procedure*)

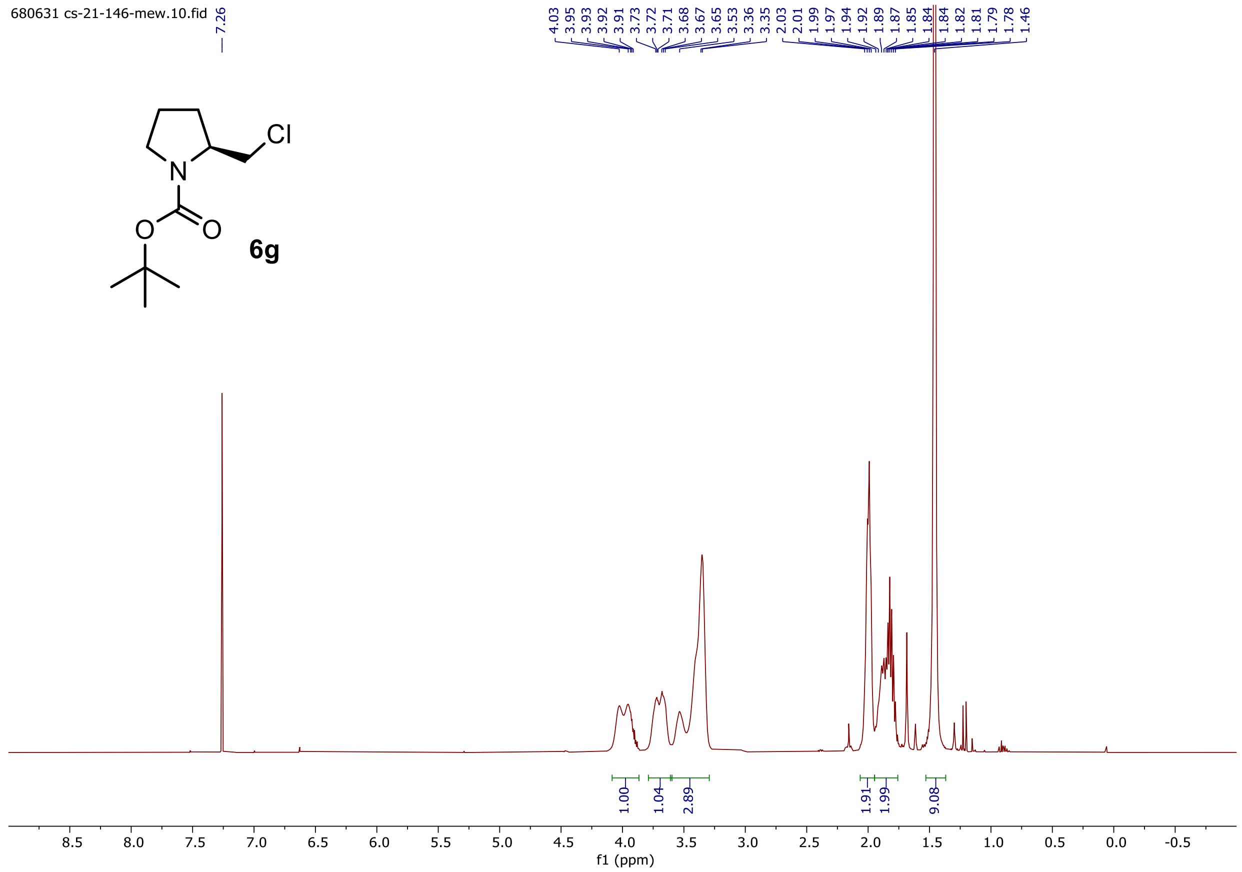

^{13}C NMR (100 MHz, $CDCl_3$) of **6g**

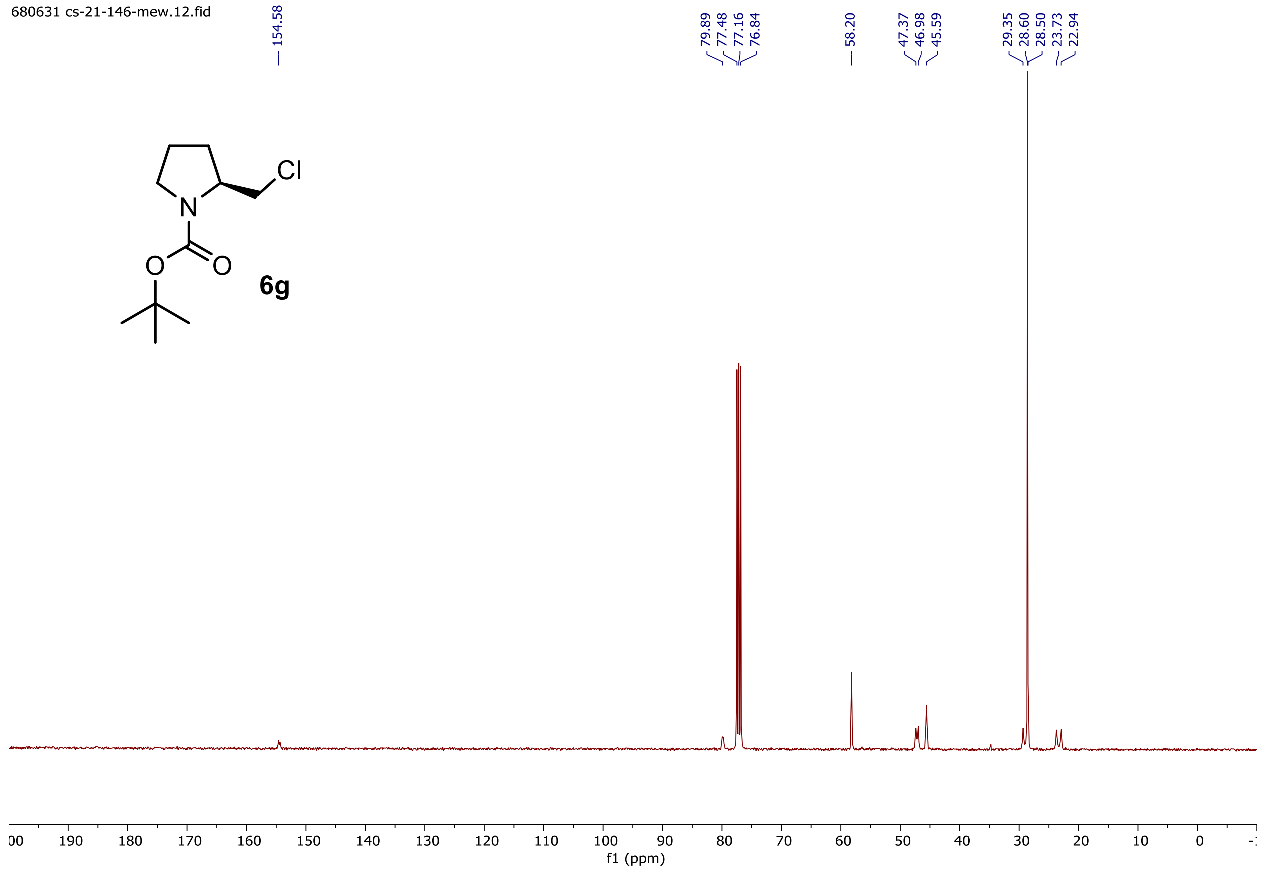

^{1}H NMR (400 MHz, $CDCl_3$) of **6h** (*see procedure*)

66984 cs-21-141-p.10.fid

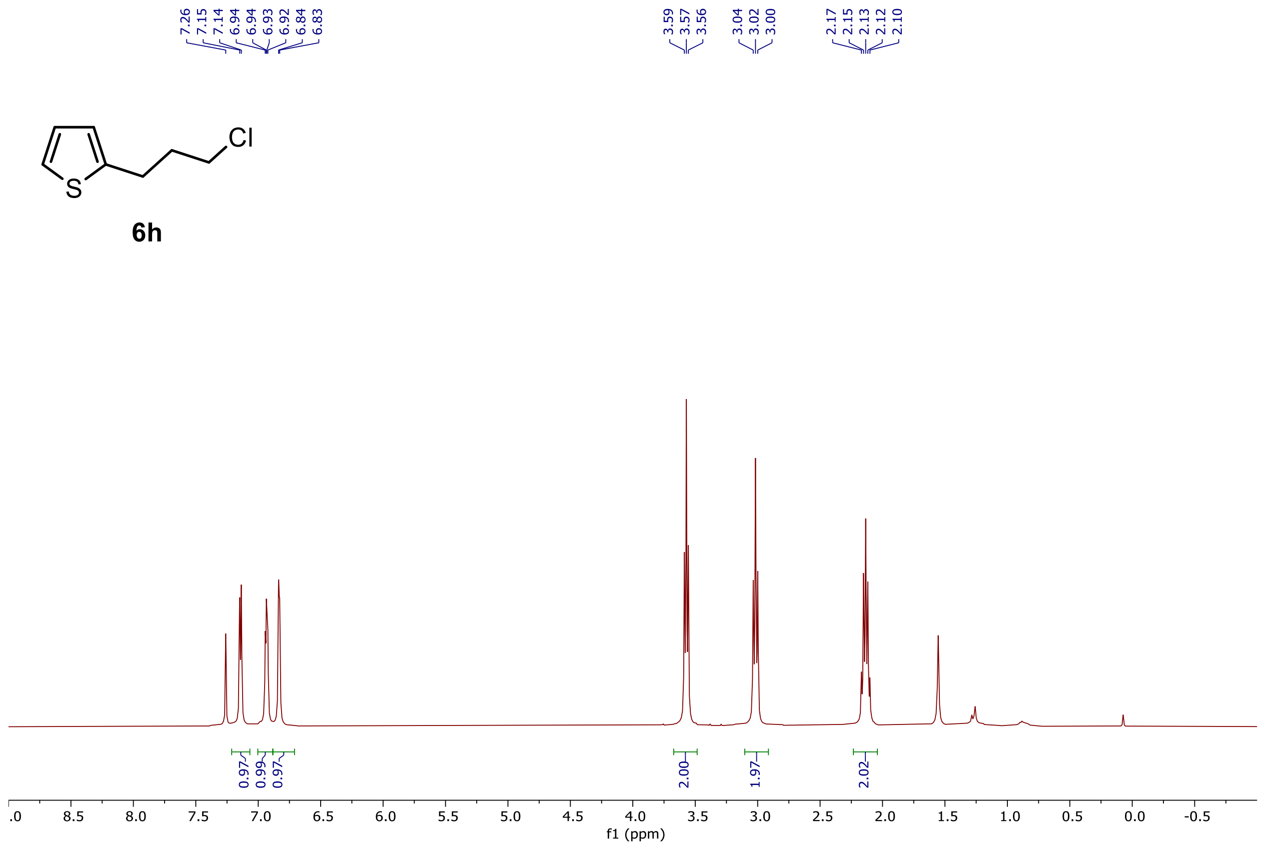

^{13}C NMR (100 MHz, $CDCl_3$) of **6h**

66984 cs-21-141-p.11.fid

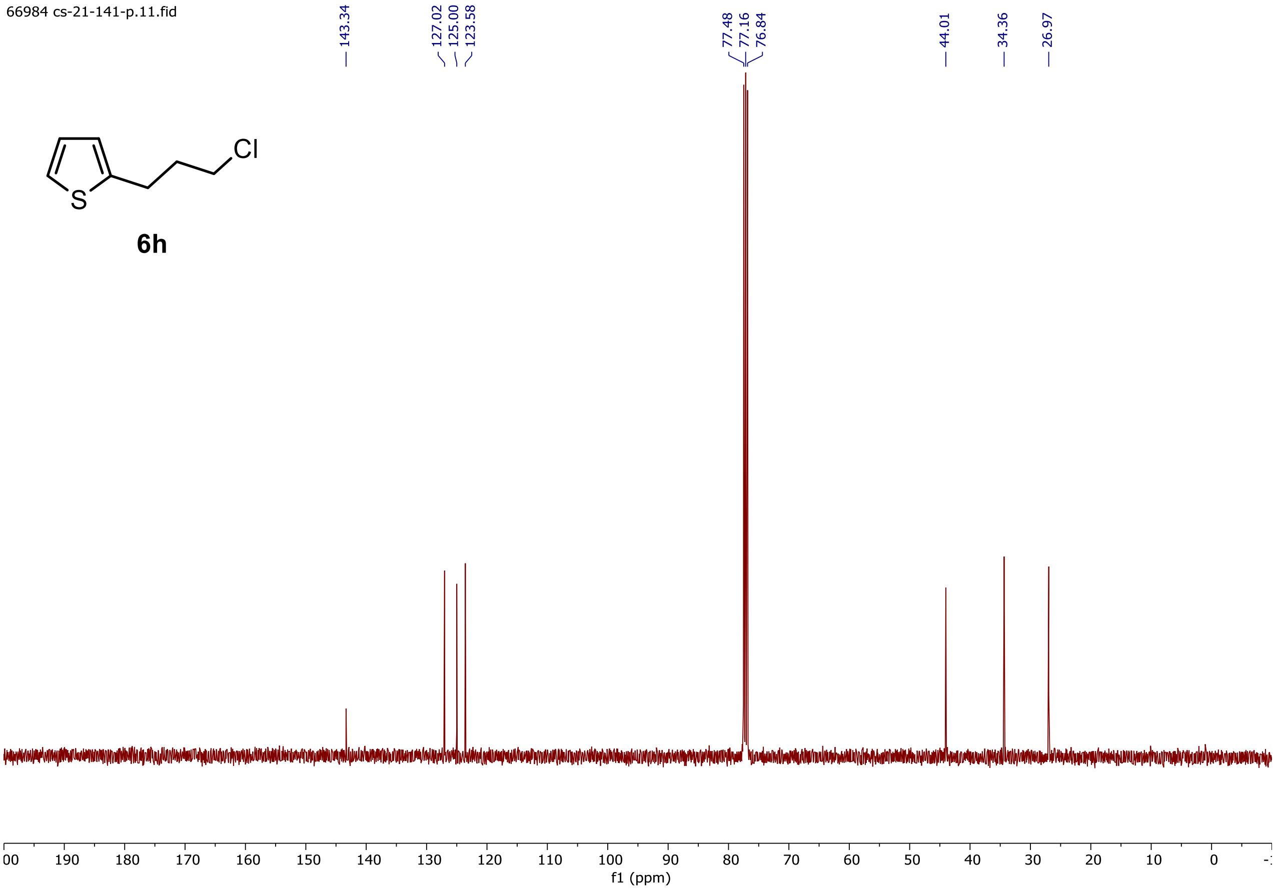

^{1}H NMR (400 MHz, $CDCl_3$) of **6i** (*see procedure*)

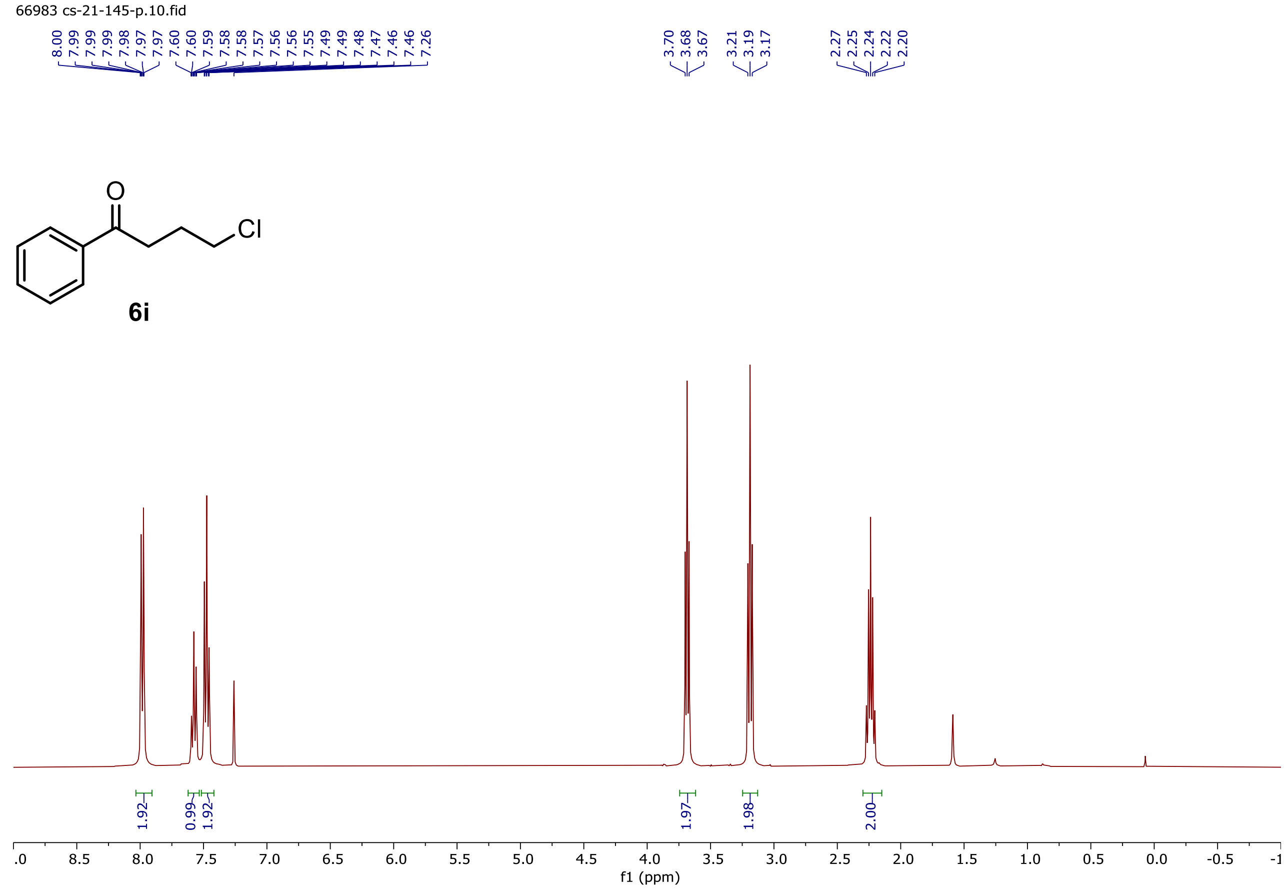

^{13}C NMR (100 MHz, $CDCl_3$) of **6i**

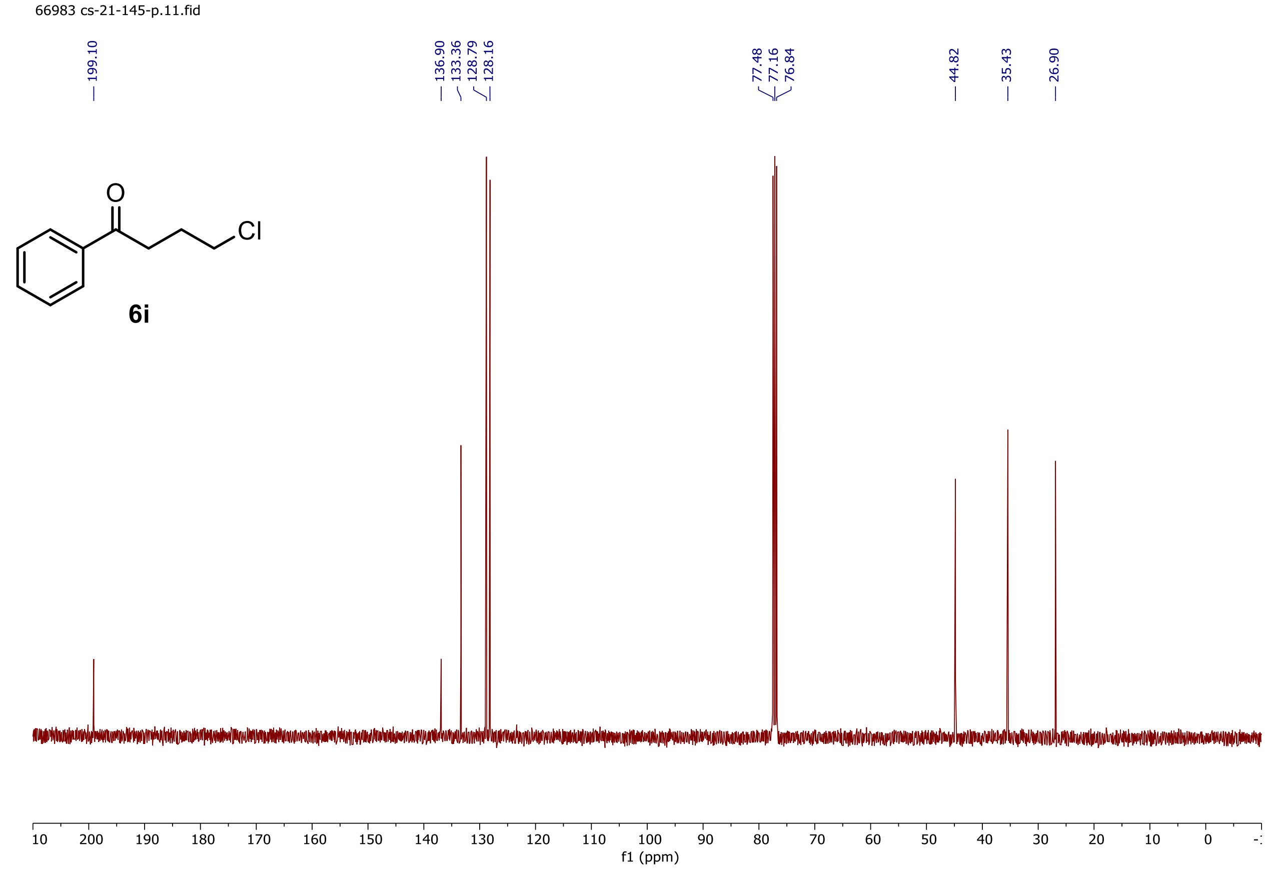

^{1}H NMR (500 MHz, $CDCl_3$) of **6j** (*see procedure*)

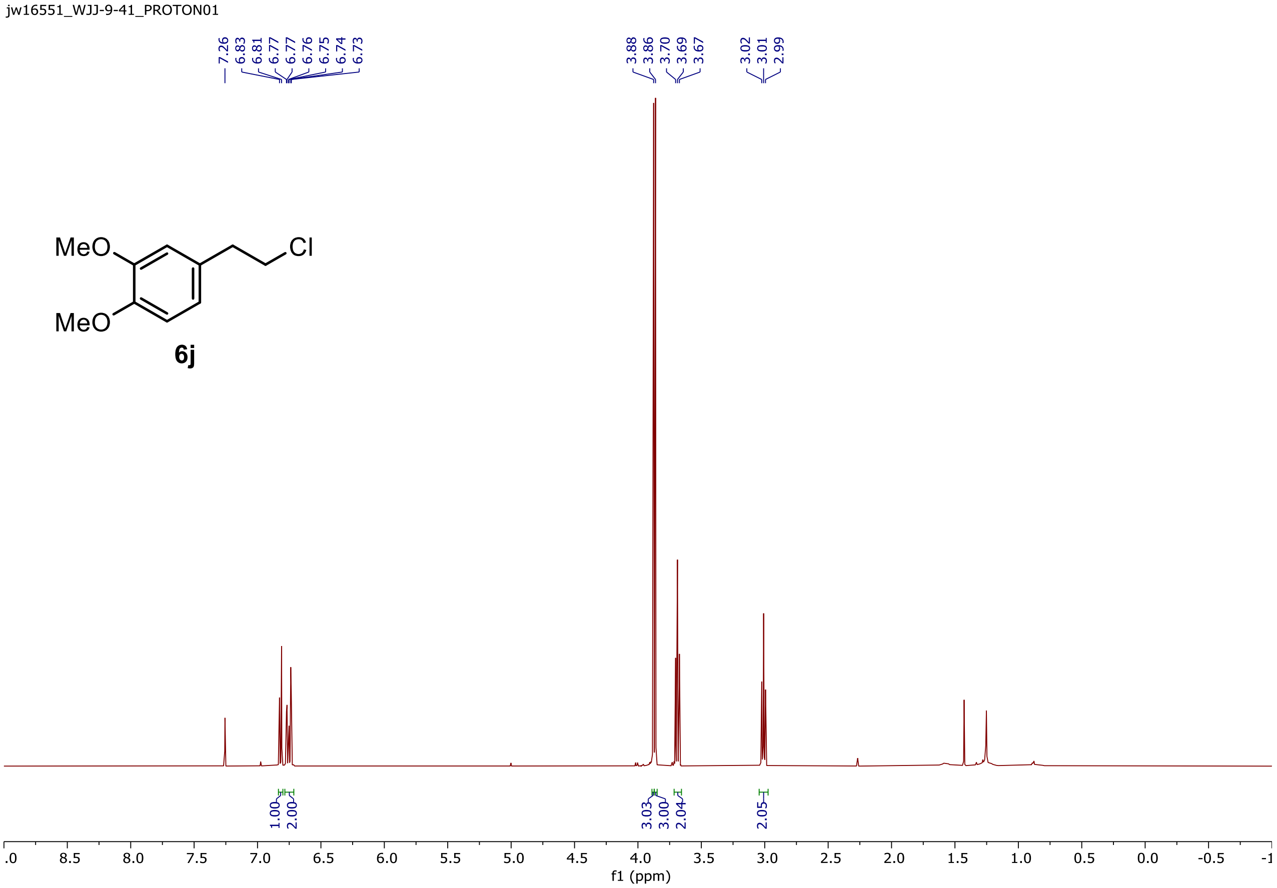

^{13}C NMR (125 MHz, $CDCl_3$) of **6j**

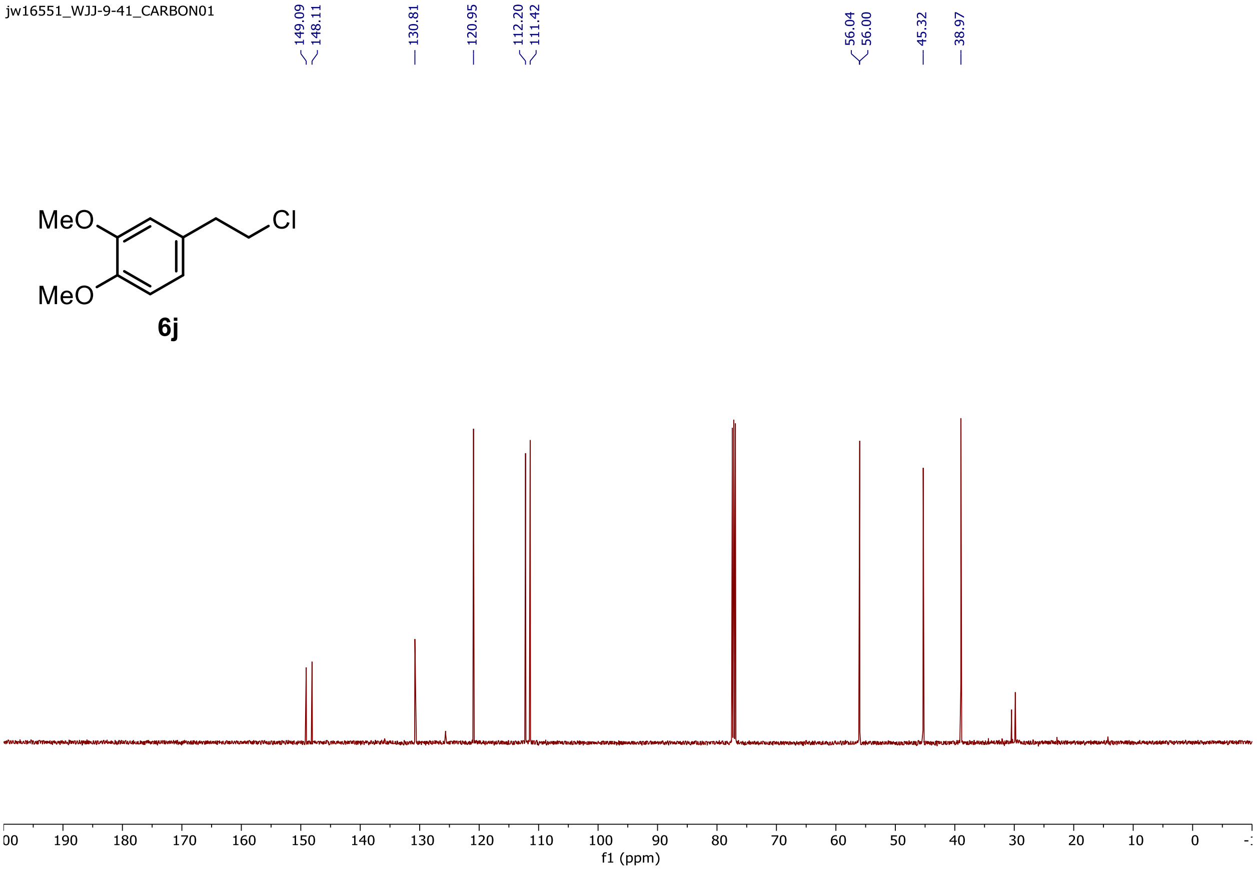

^{1}H NMR (500 MHz, $CDCl_3$) of **6k** (*see procedure*)

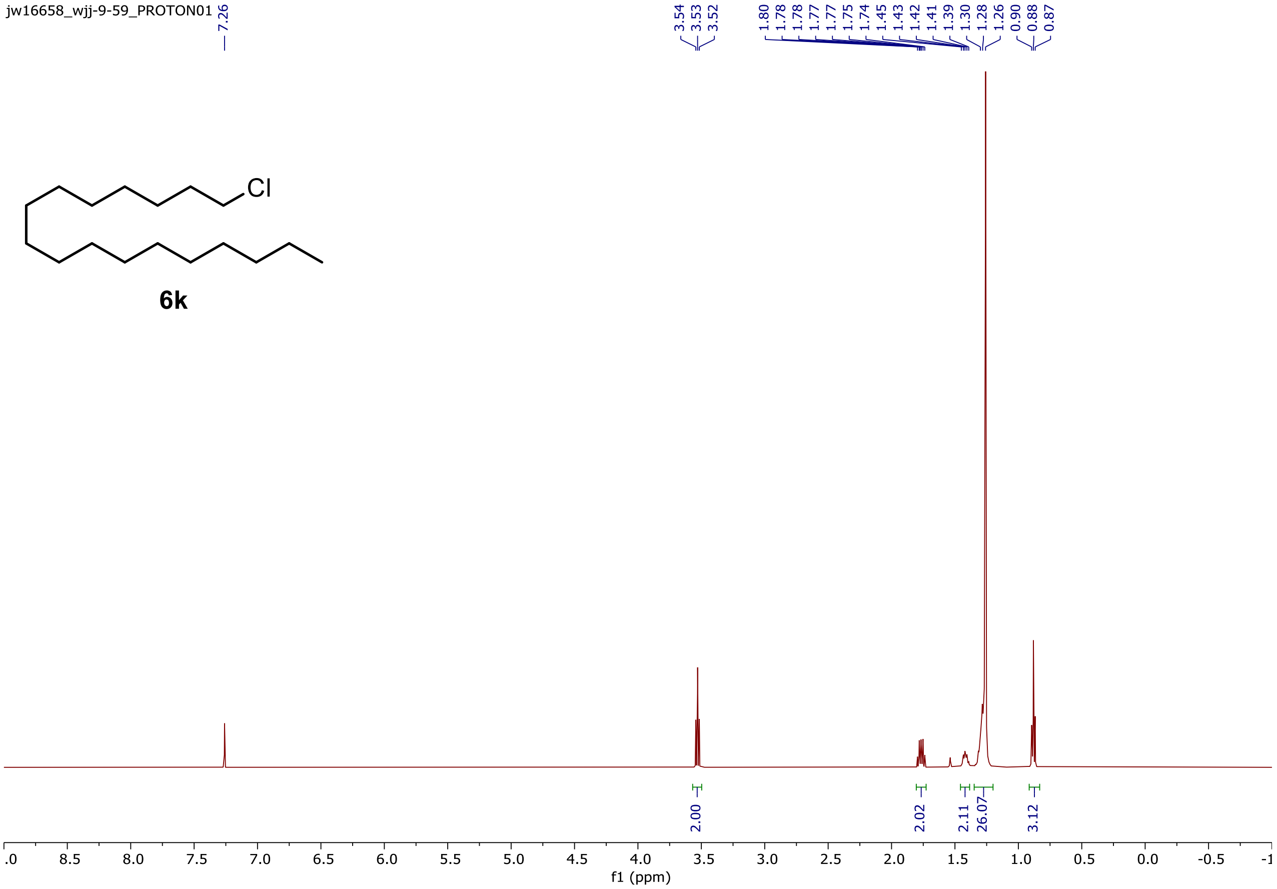

^{13}C NMR (125 MHz, $CDCl_3$) of **6k**

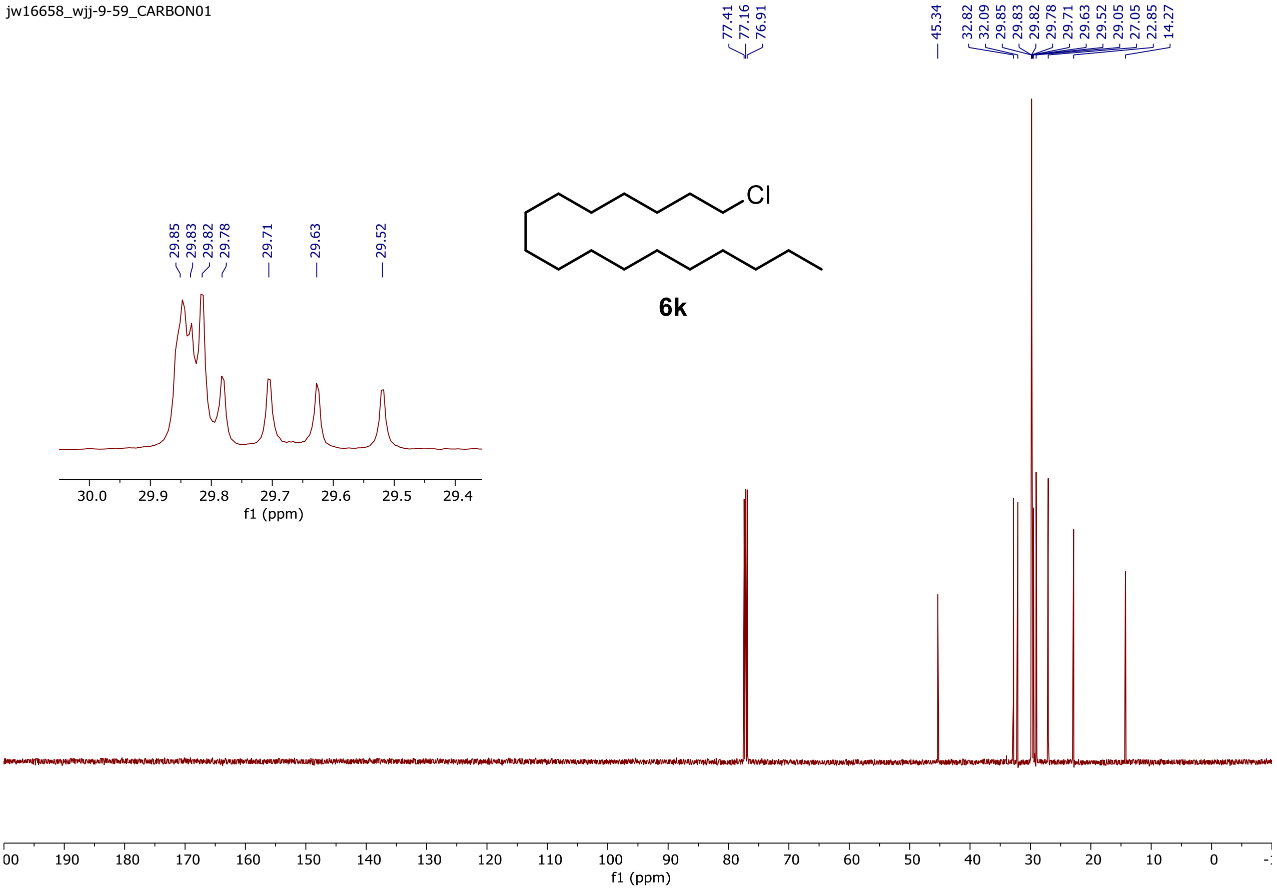

^{1}H NMR (500 MHz, $CDCl_3$) of **6l** (*see procedure*)

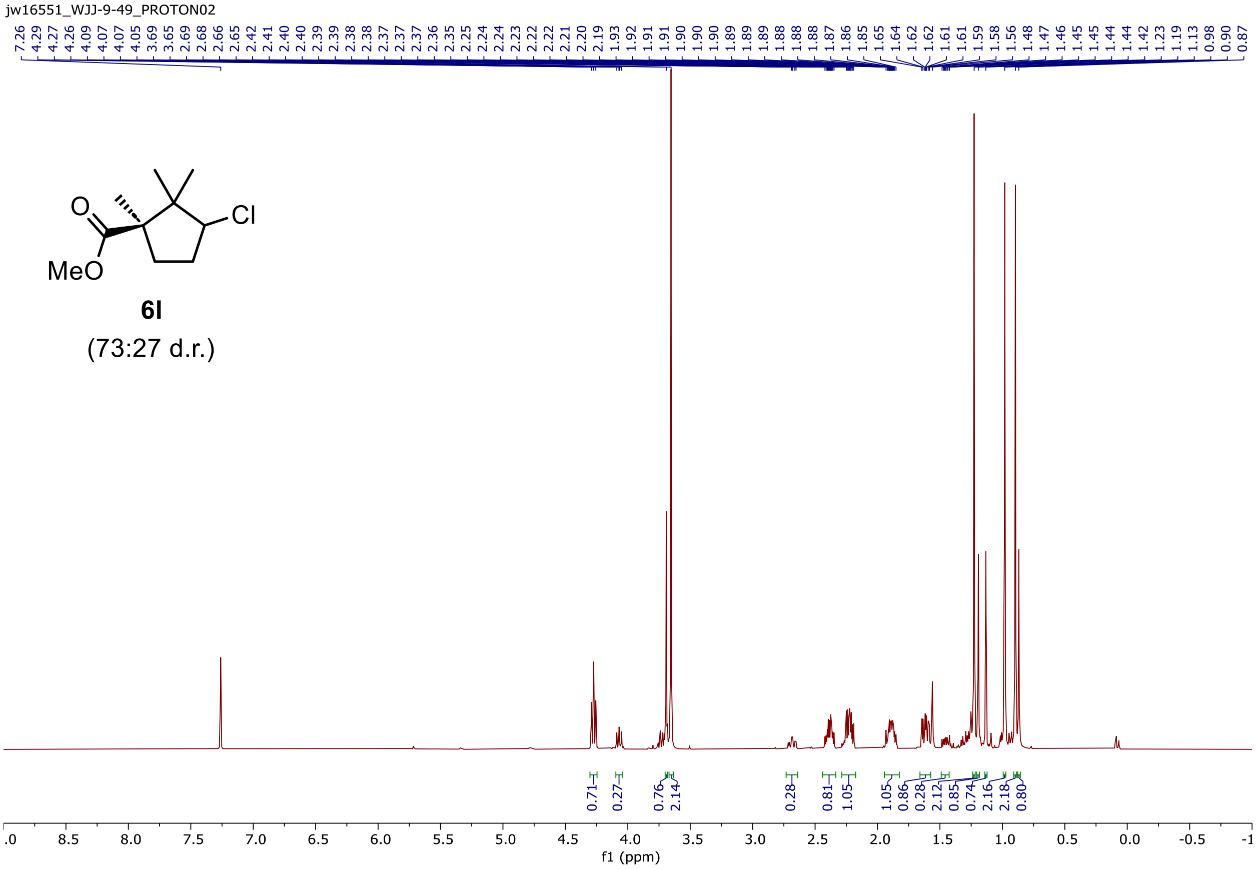

^{13}C NMR (125 MHz, $CDCl_3$) of **6l**

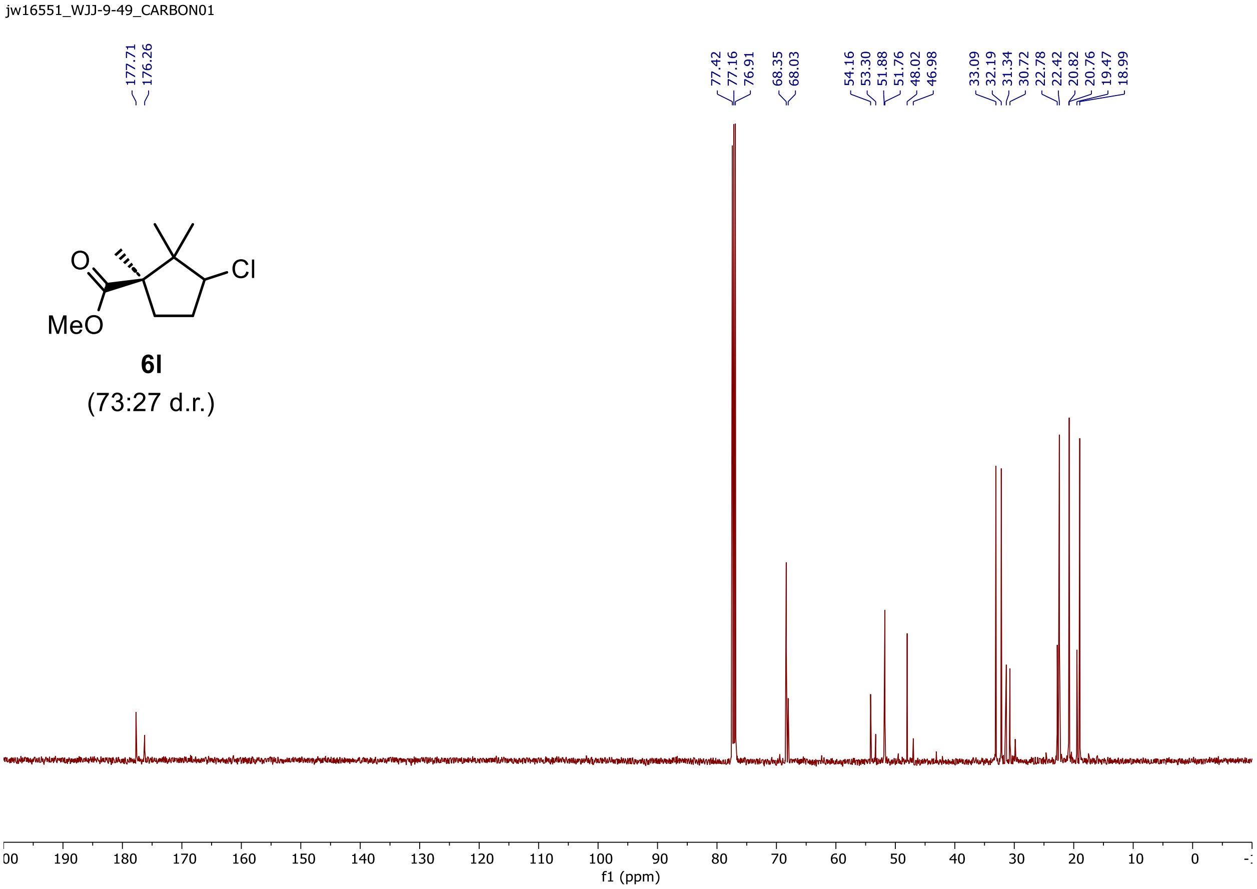

^{1}H NMR (500 MHz, $CDCl_3$) of **6m** (*see procedure*)

jw23280_WJJ-9-15_PROTON_001

6m

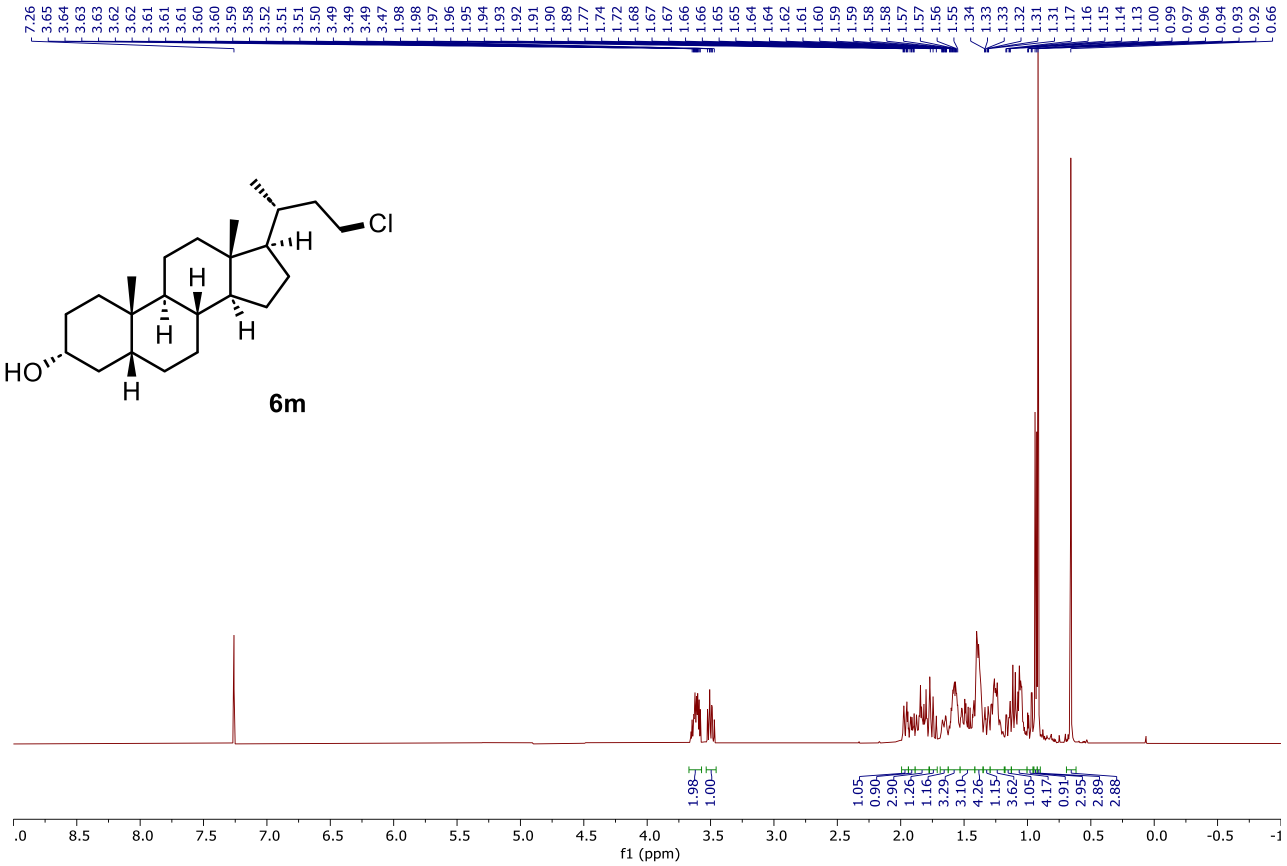

^{13}C NMR (125 MHz, $CDCl_3$) of **6m**

jw23280_WJJ-9-15_CARBON_001

6m

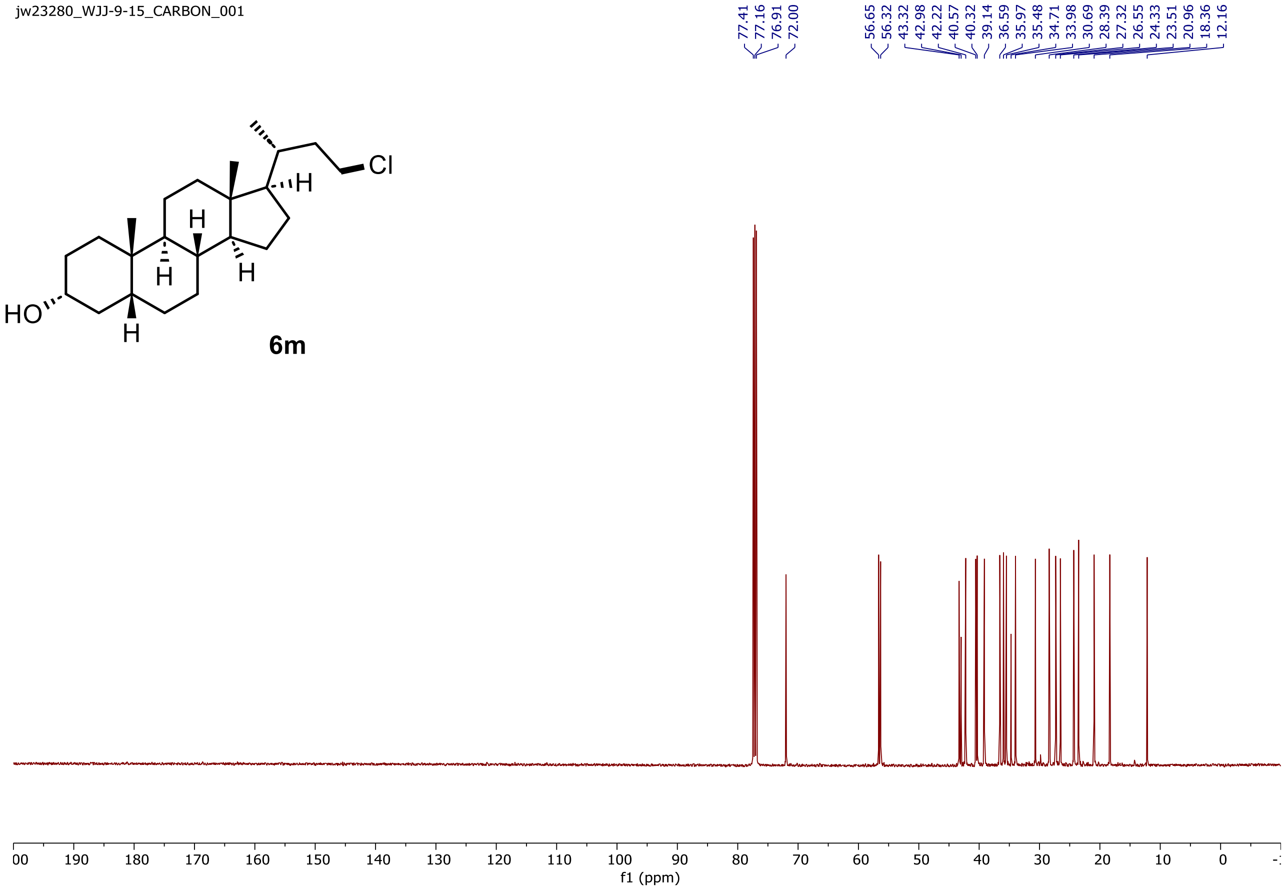

^{1}H–^{1}H COSY (CDCl$_3$) of **6m**

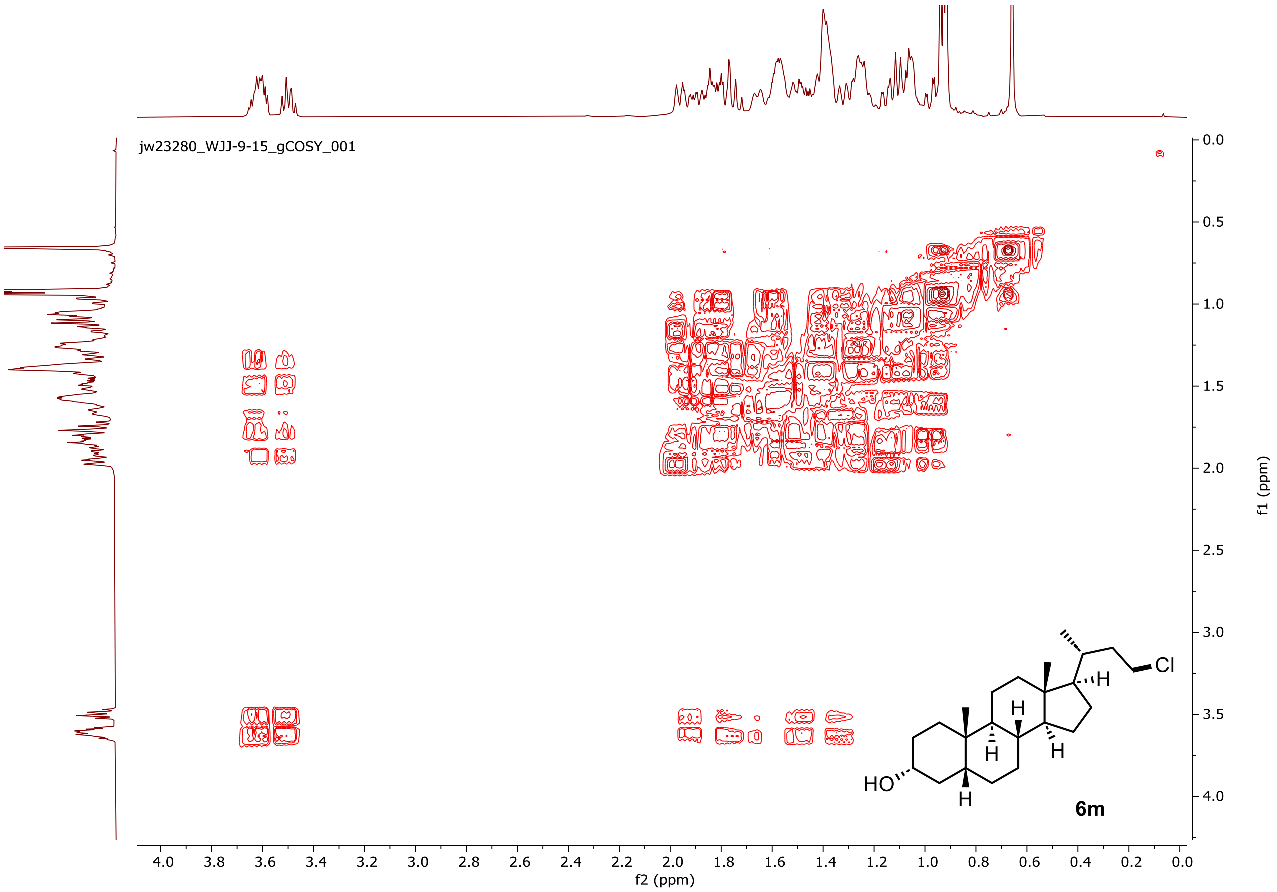

^{1}H–^{13}C HSQC (CDCl$_3$) of **6m**

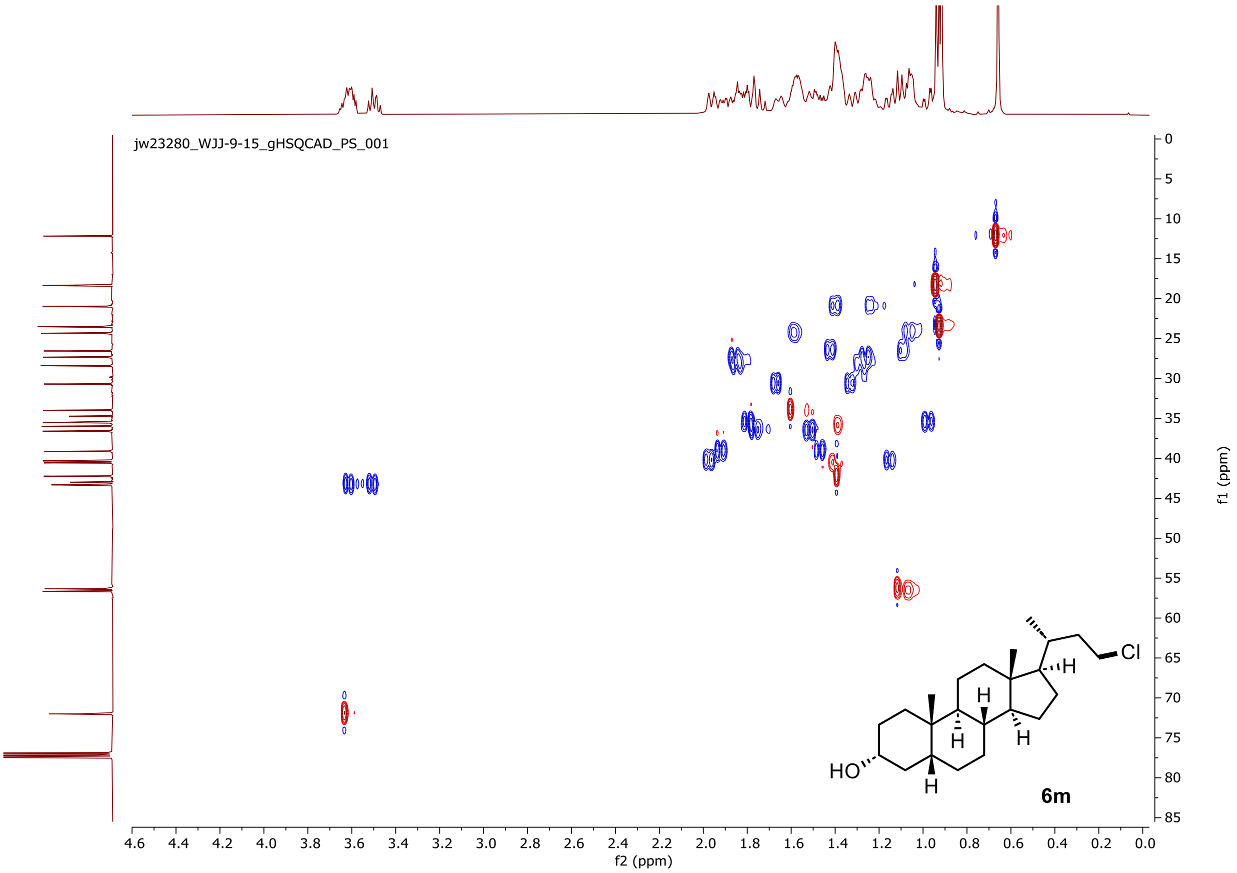

^{1}H NMR (500 MHz, CDCl$_3$) of **6n** (*see procedure*)

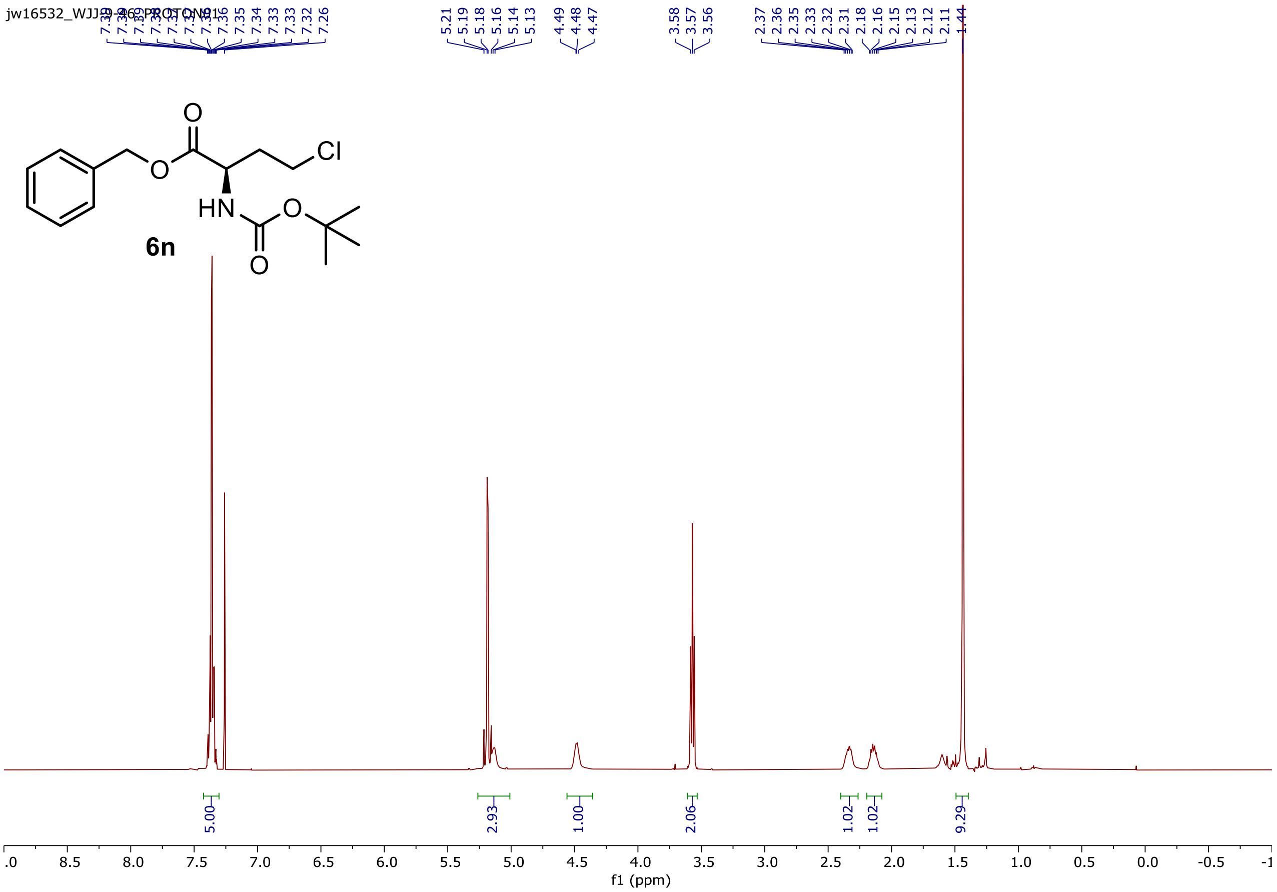

^{13}C NMR (125 MHz, CDCl$_3$) of **6n**

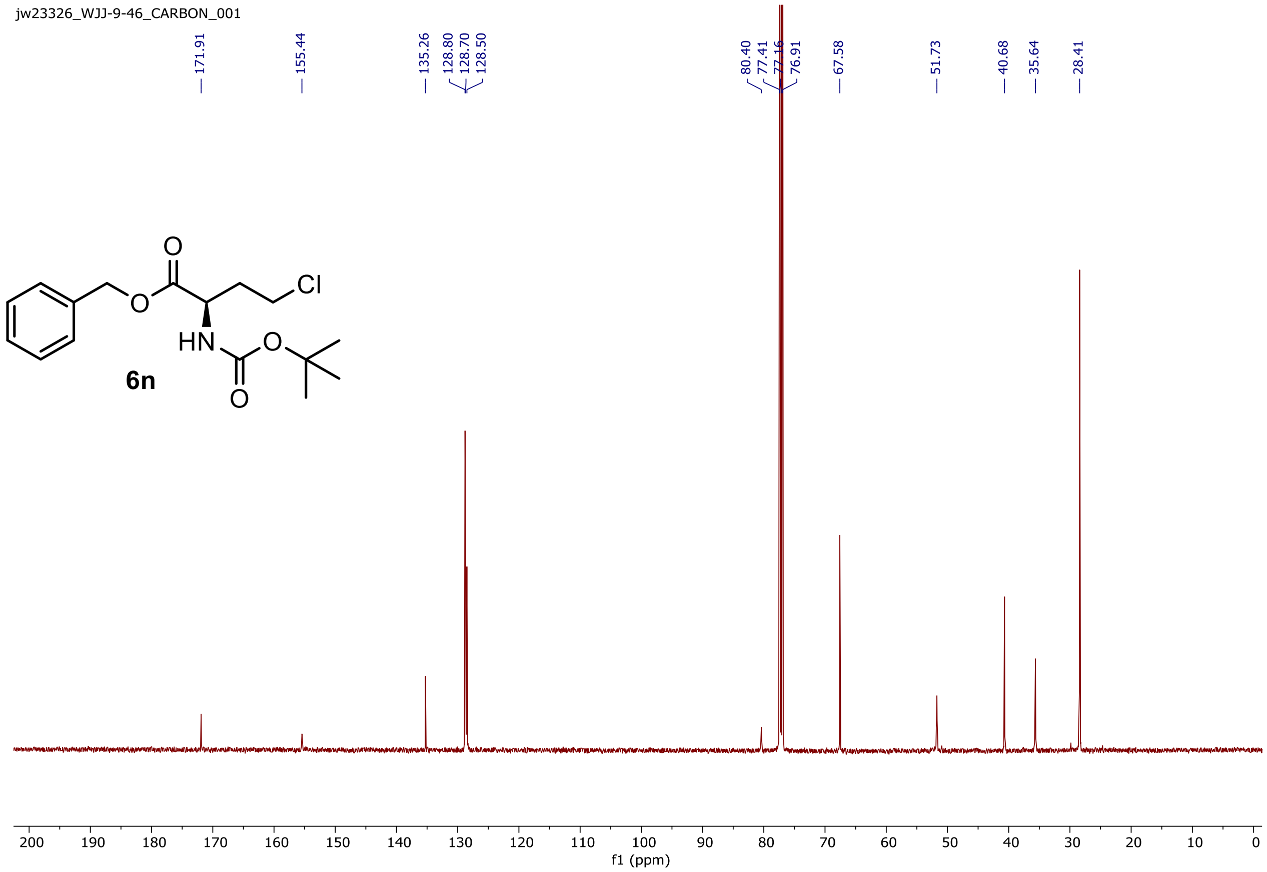

^{1}H NMR (500 MHz, CD_3CN) of **6o** (*see procedure*)

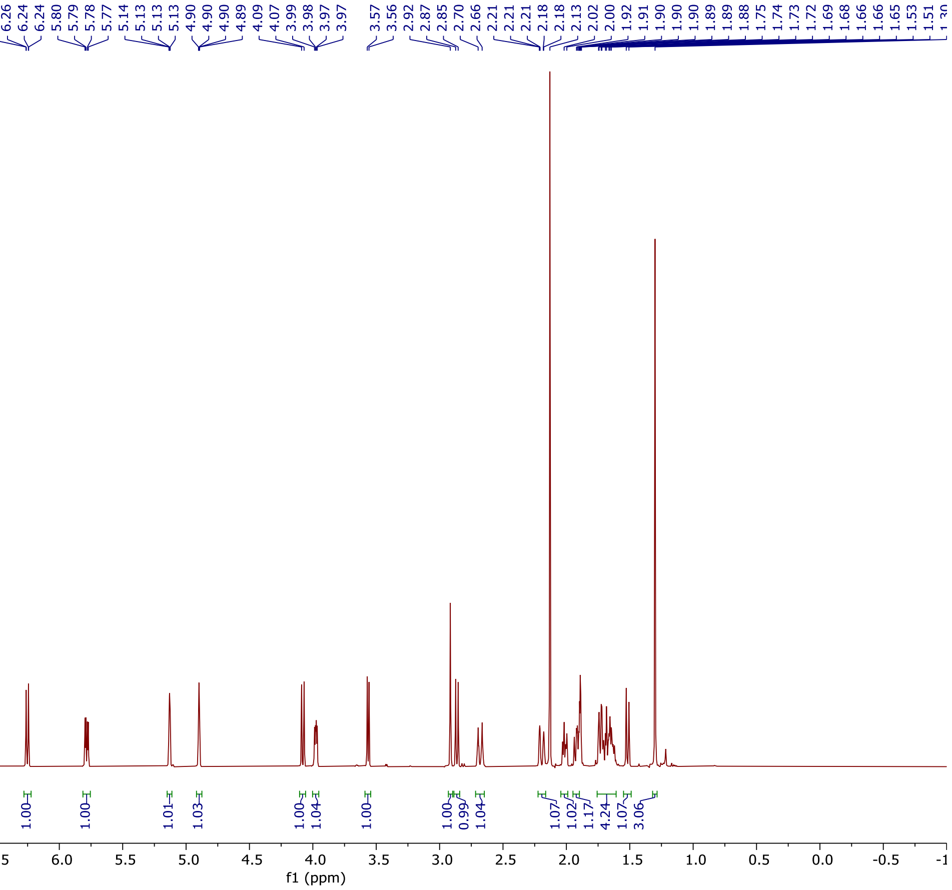

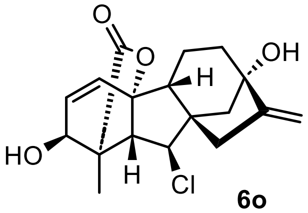

6o

^{13}C NMR (125 MHz, CD_3CN) of **6o**

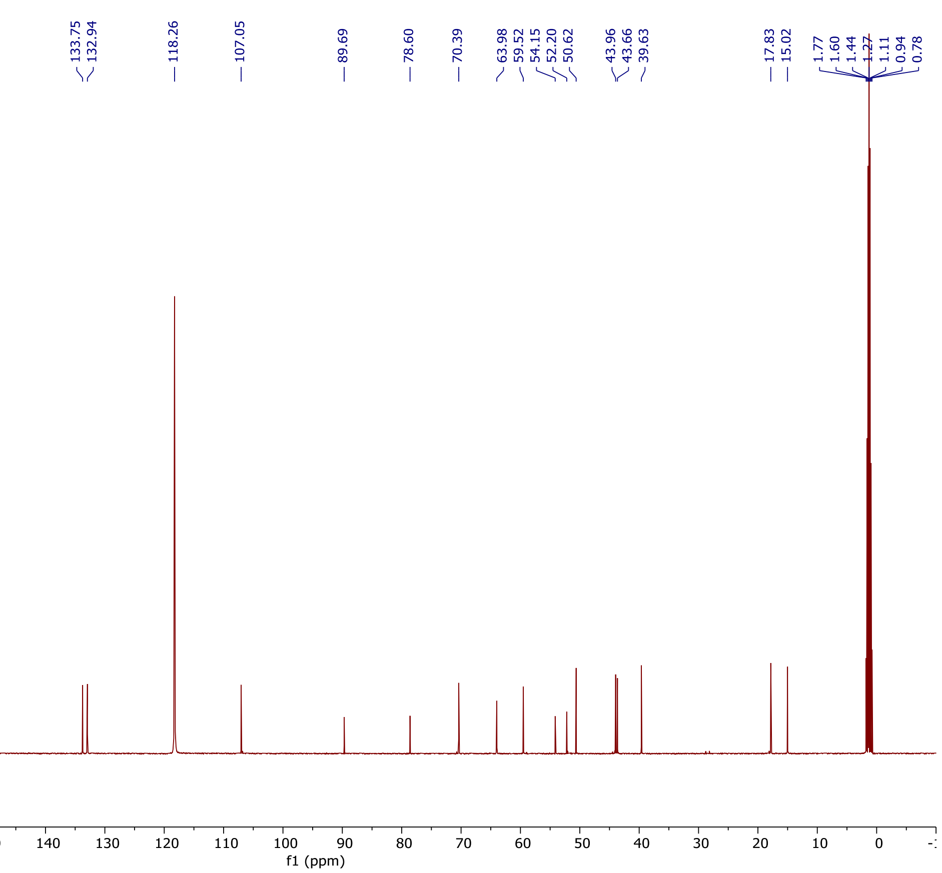

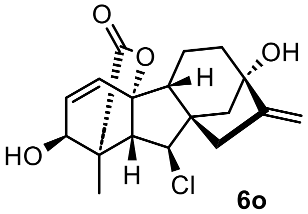

6o

^1H–^1H COSY (CD_3CN) of **6o**

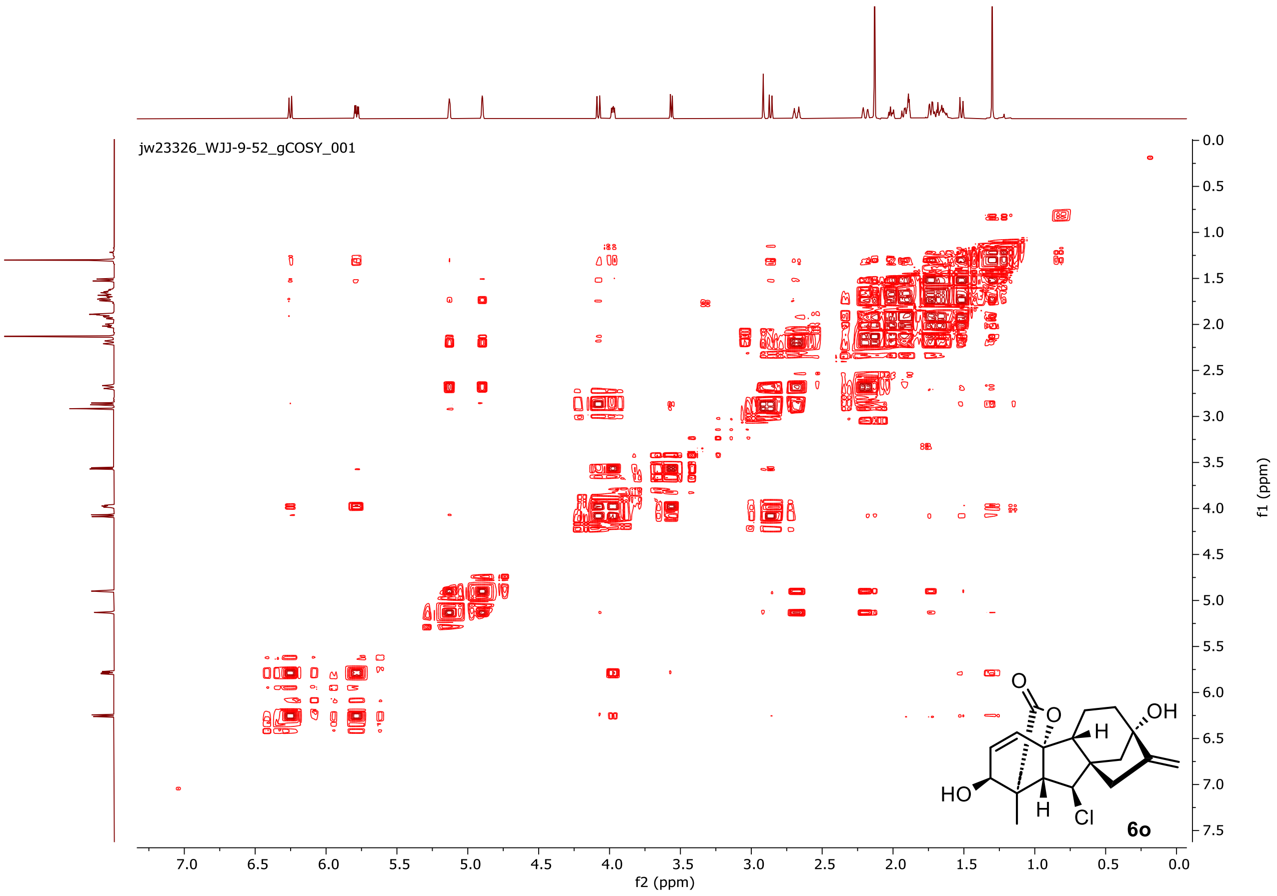

^1H–^{13}C HSQC (CD_3CN) of **6o**

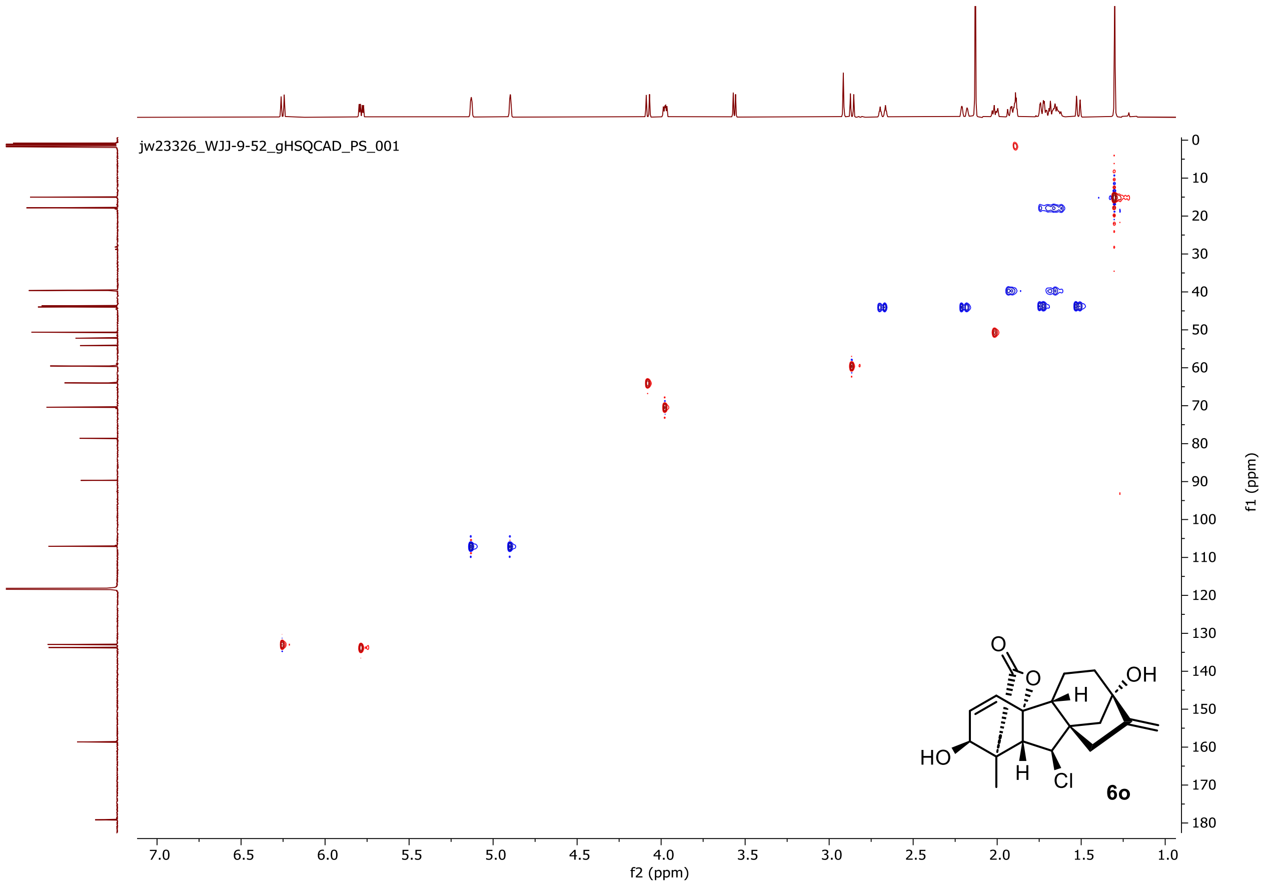

^{1}H NMR (500 MHz, $CDCl_3$) of **6p** (see procedure)

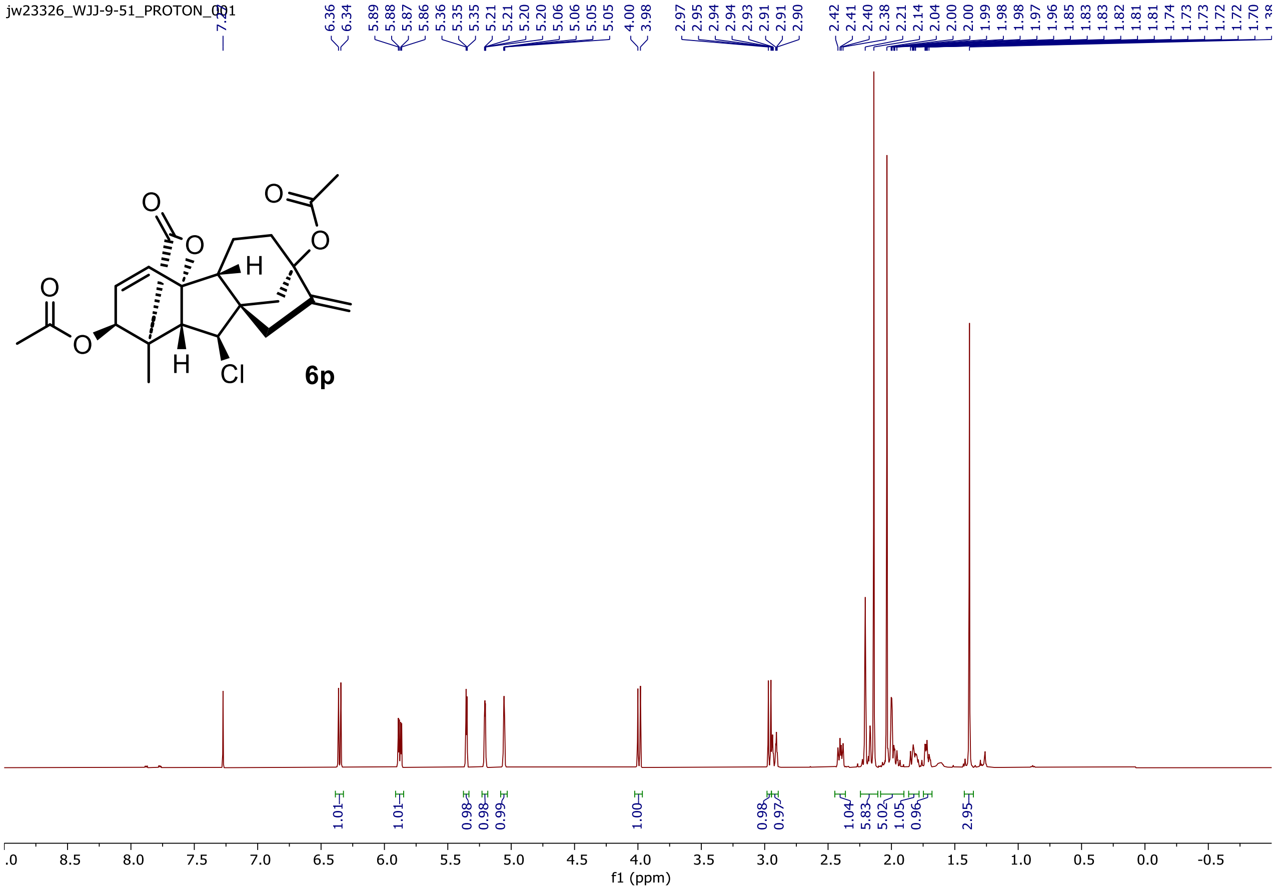

^{13}C NMR (125 MHz, $CDCl_3$) of **6p**

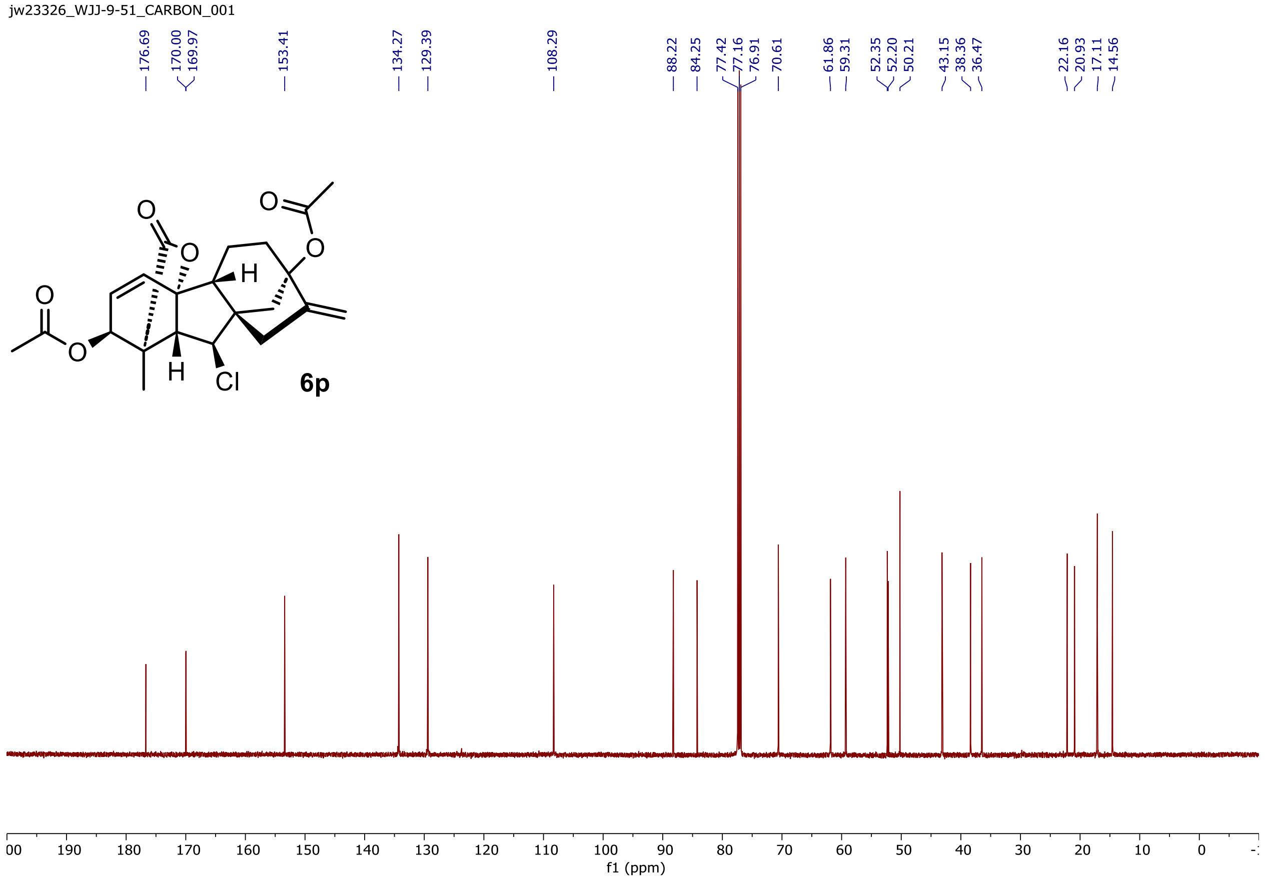

^{1}H–^{1}H COSY ($CDCl_3$) of **6p**

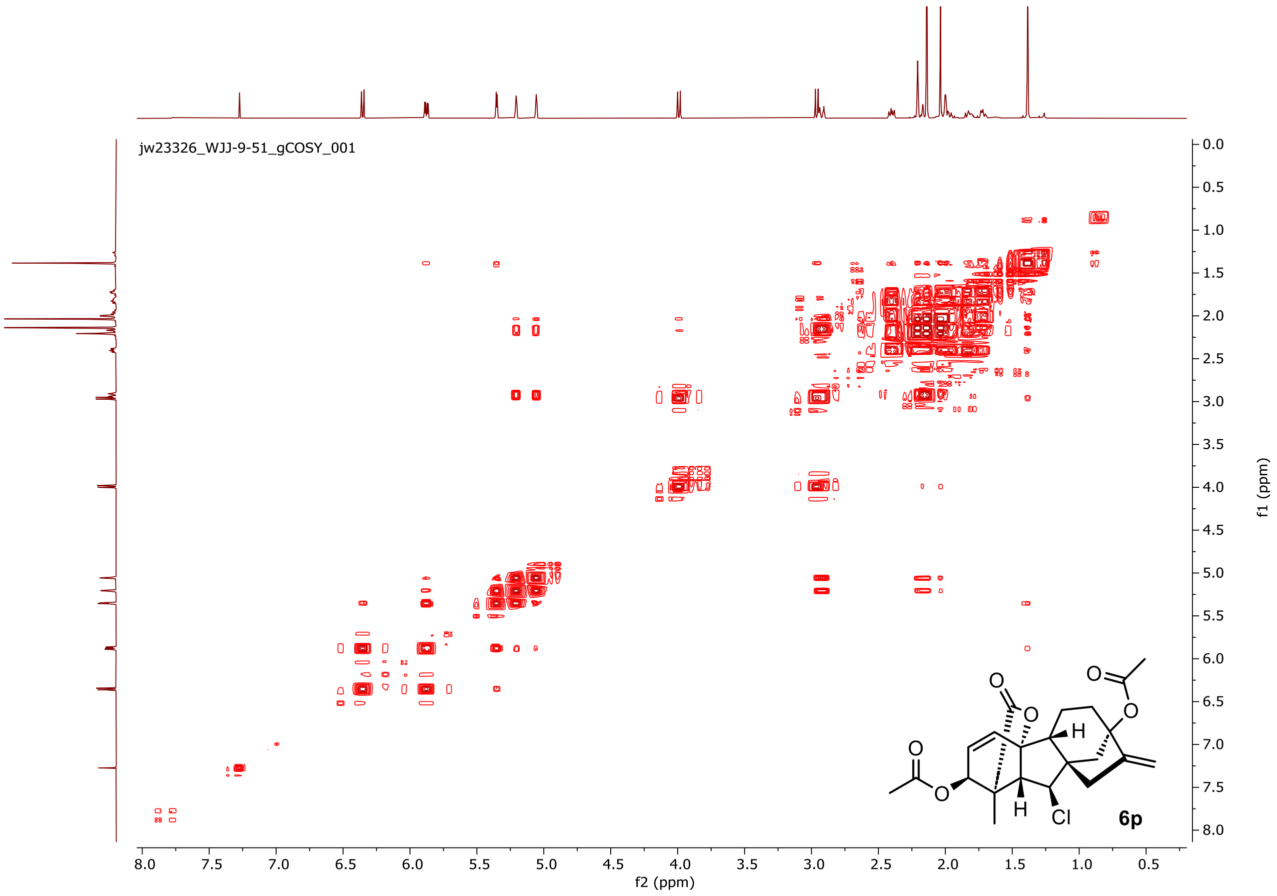

^{1}H–^{13}C HSQC ($CDCl_3$) of **6p**

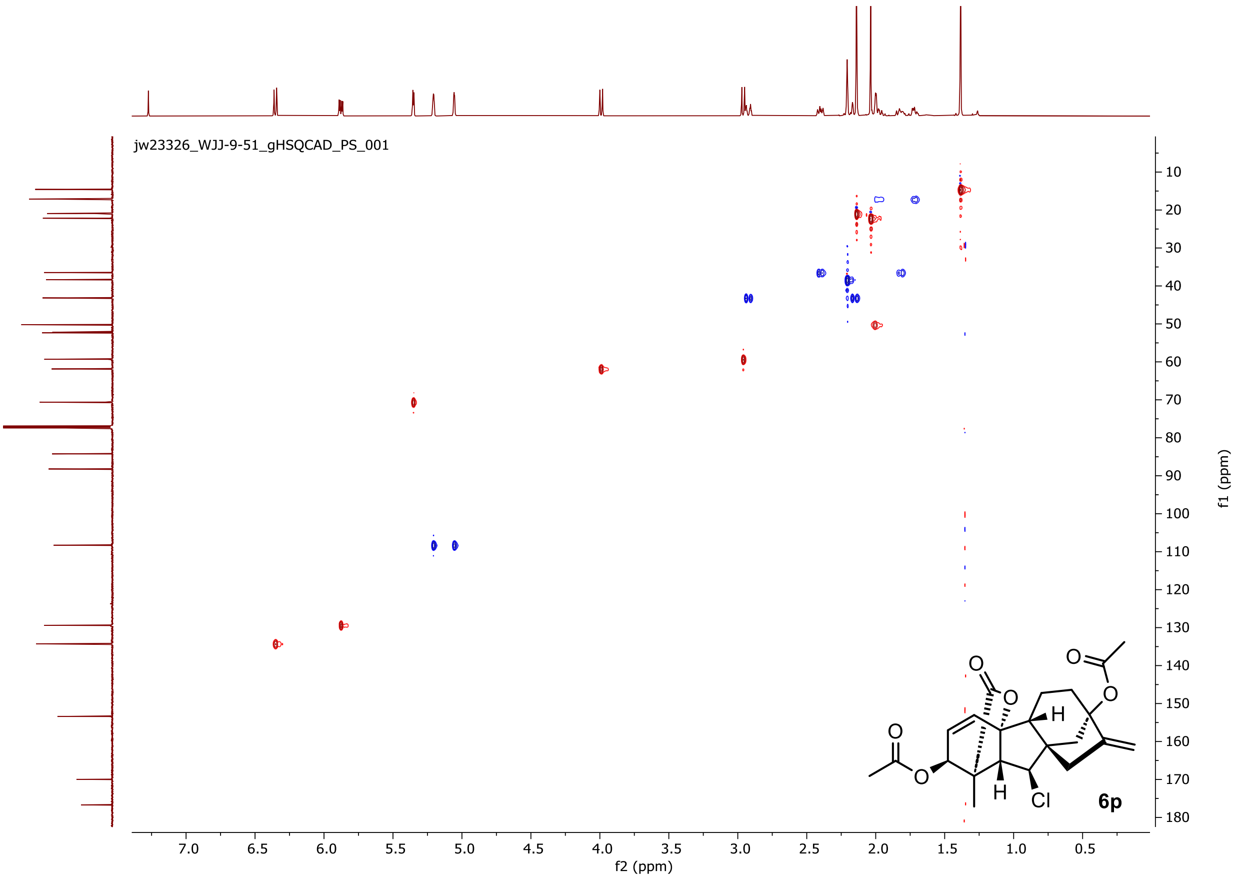

^{1}H NMR (500 MHz, $CDCl_3$) of **6p'** (*see procedure*)

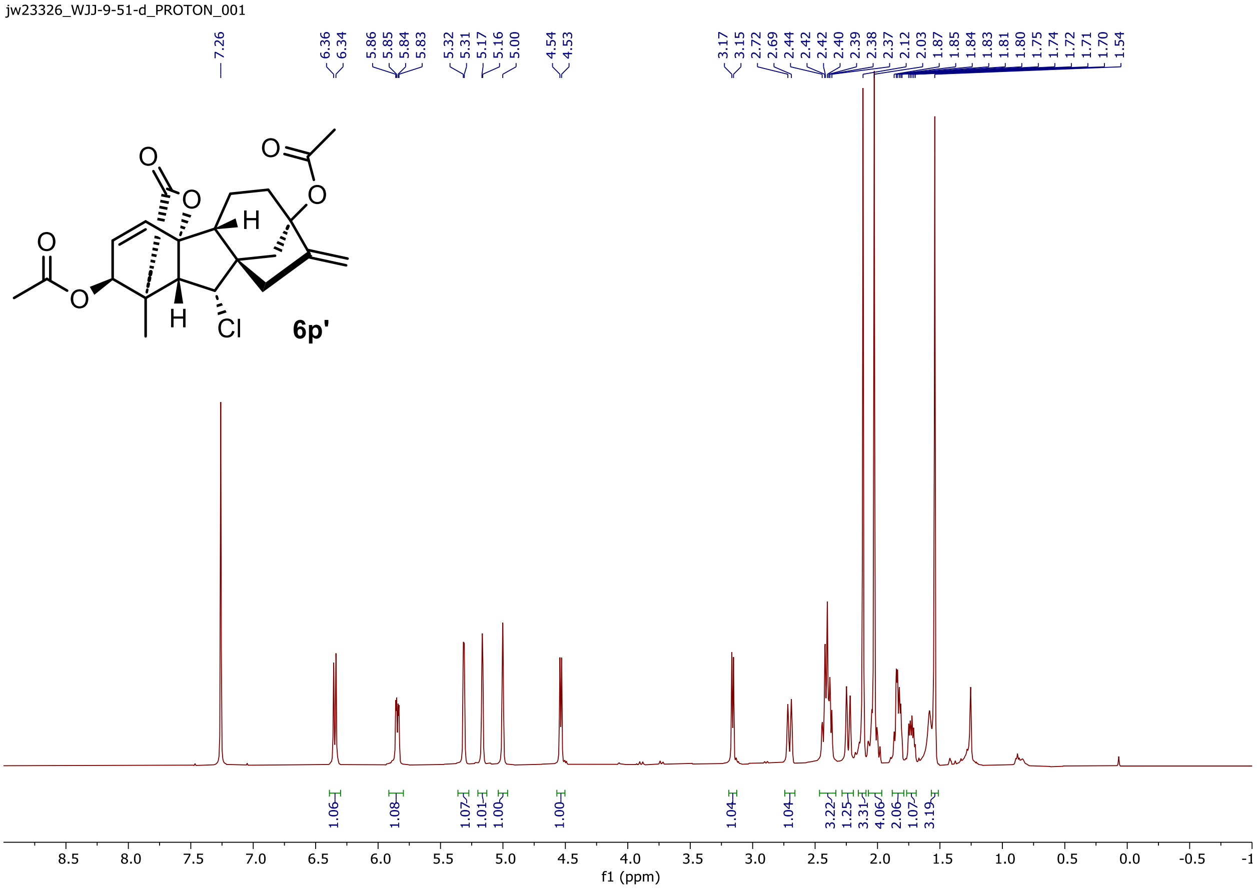

^{13}C NMR (125 MHz, $CDCl_3$) of **6p'**

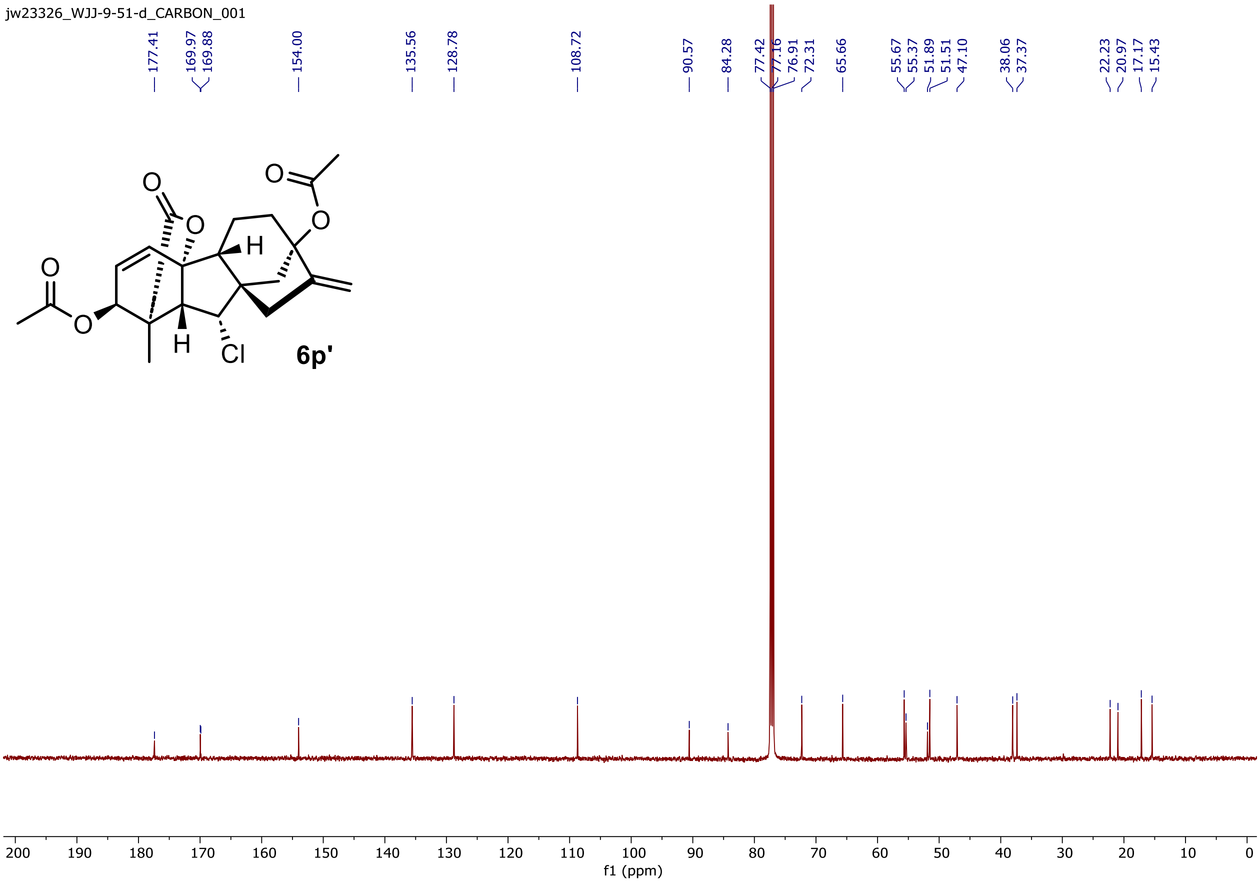

^{1}H–^{1}H COSY ($CDCl_3$) of **6p'**

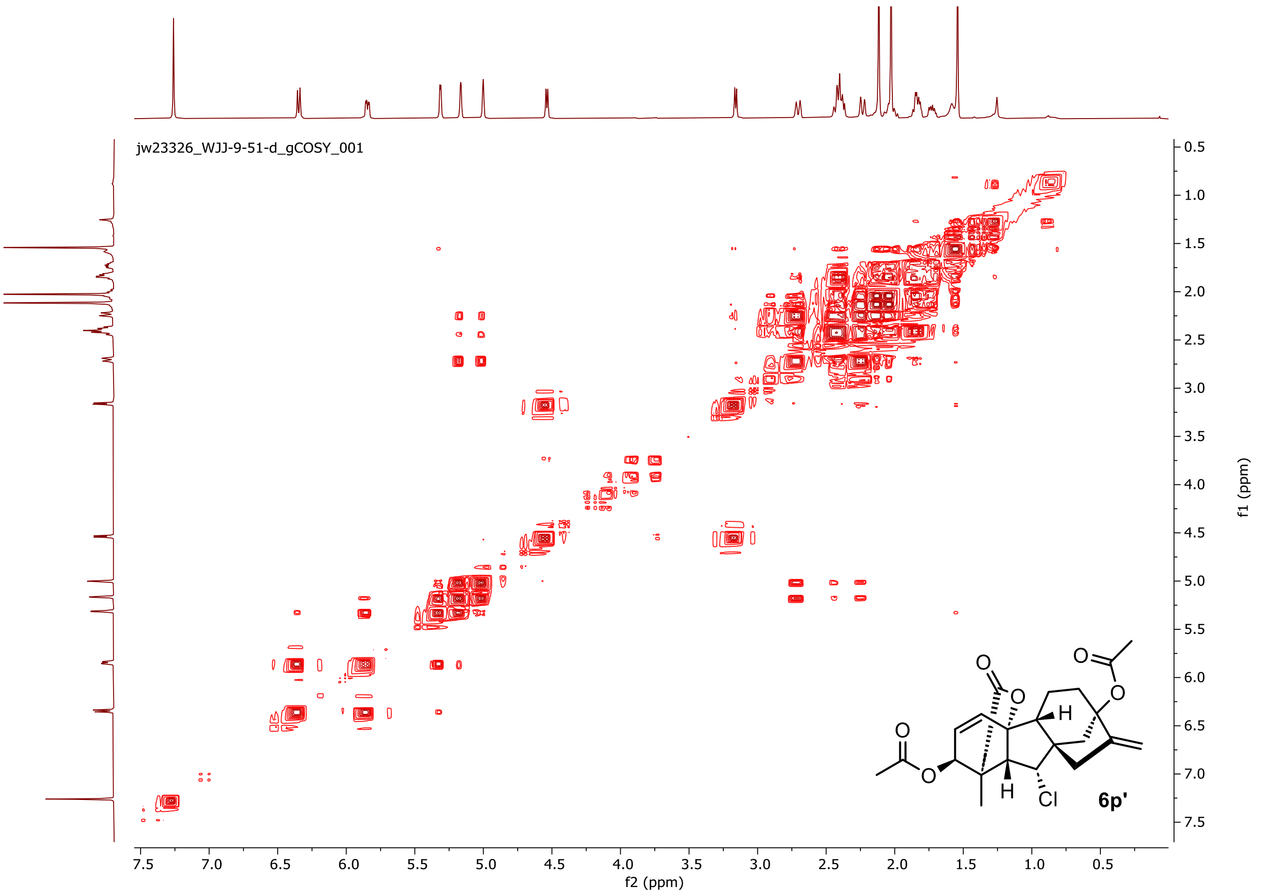

^{1}H–^{13}C HSQC ($CDCl_3$) of **6p'**

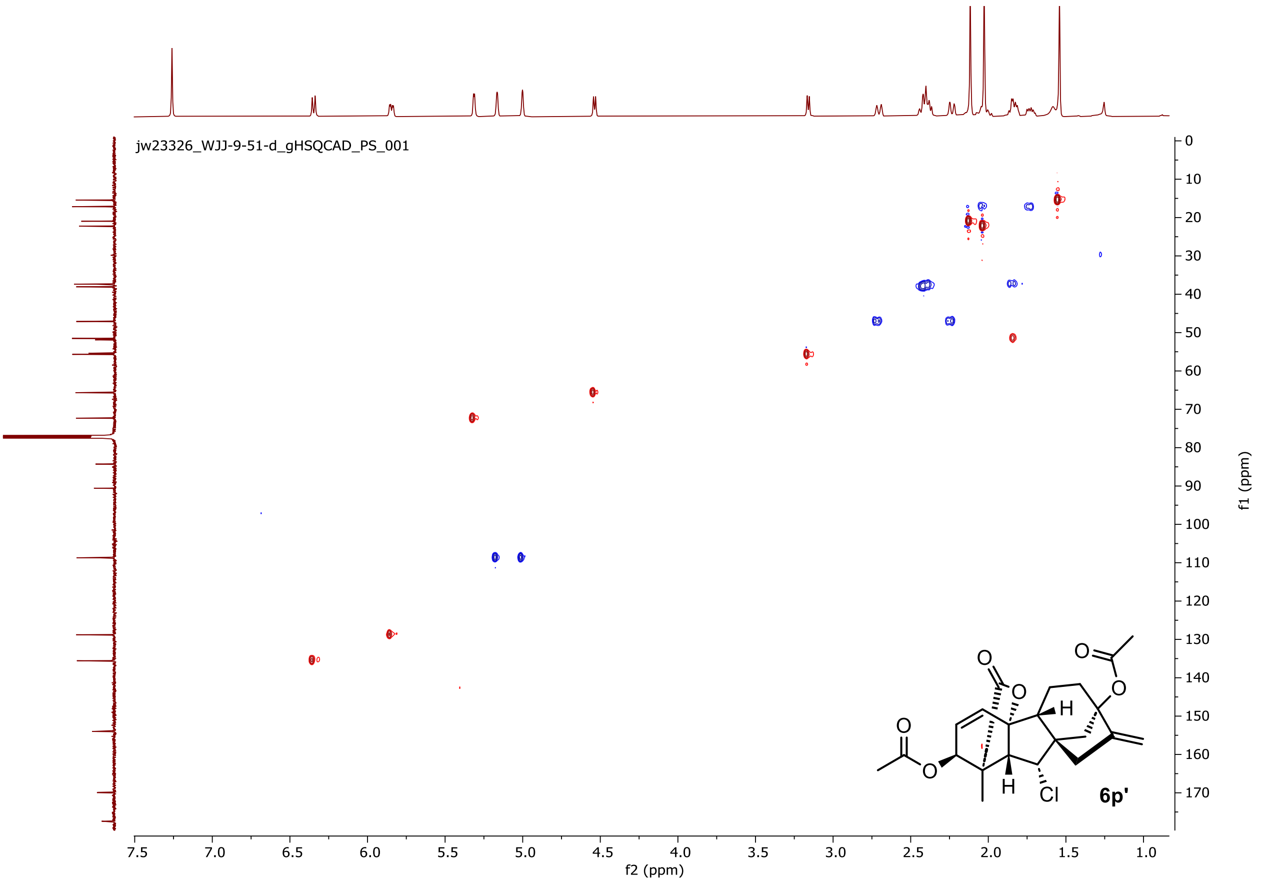

^{1}H NMR (500 MHz, $CDCl_3$) of **7a** (*see procedure*)

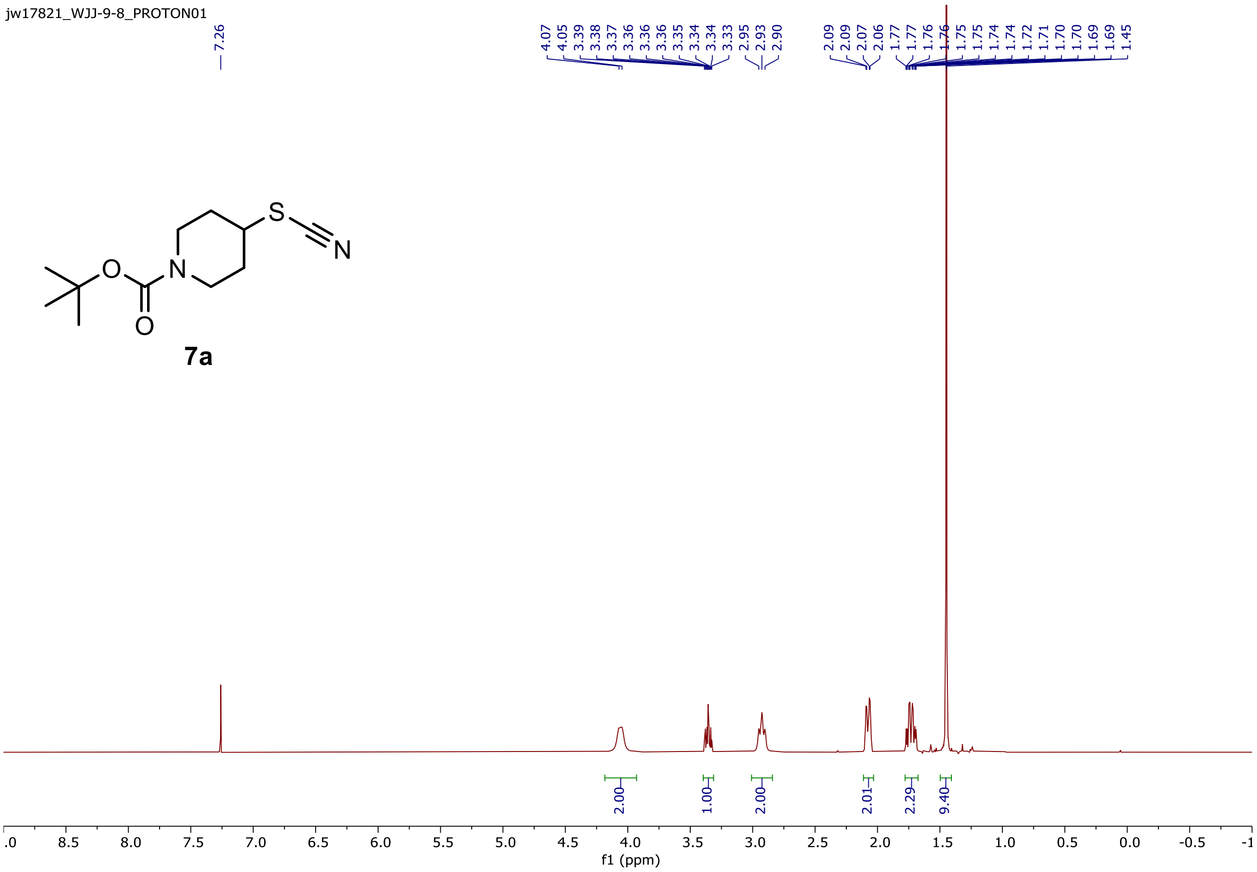

^{13}C NMR (125 MHz, $CDCl_3$) of **7a**

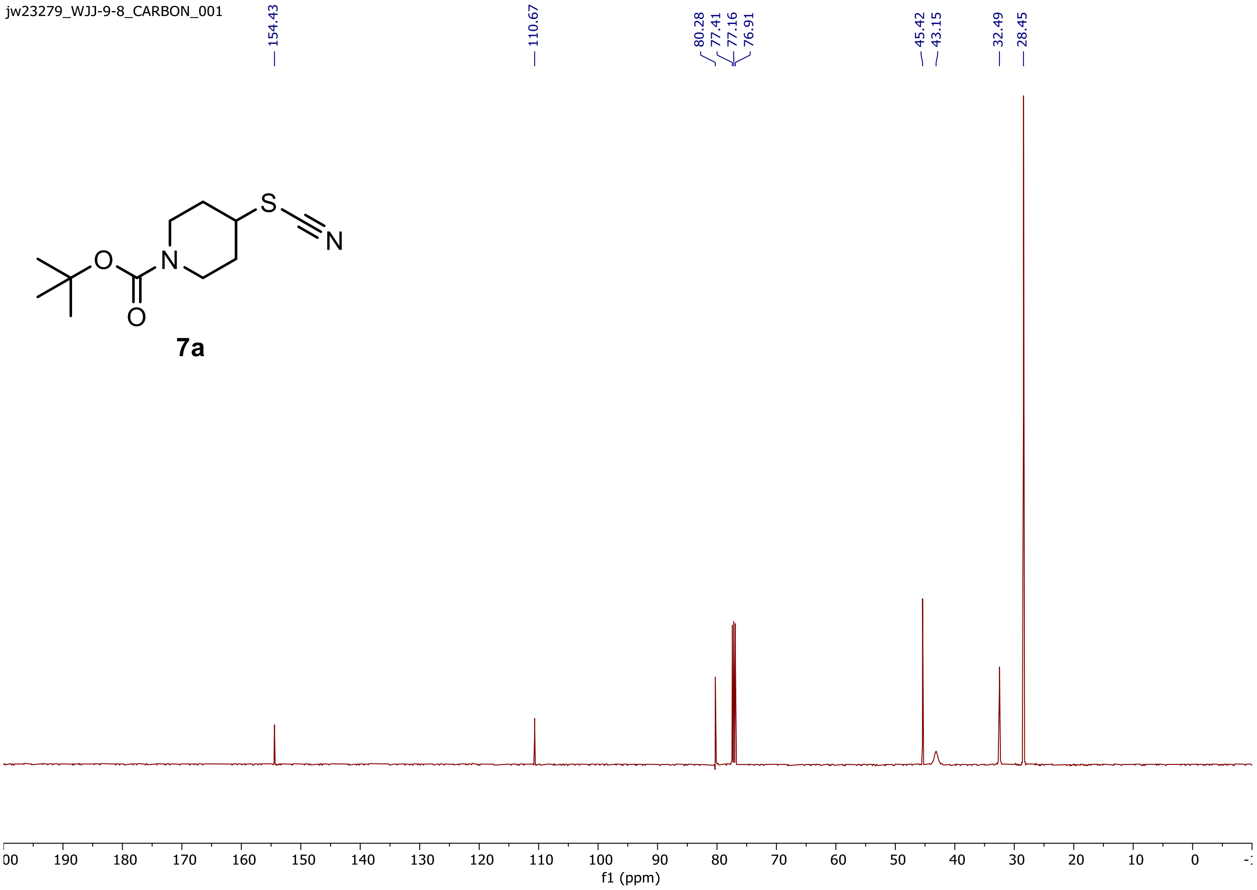

^{1}H NMR (500 MHz, $CDCl_3$) of **7b** (see procedure)

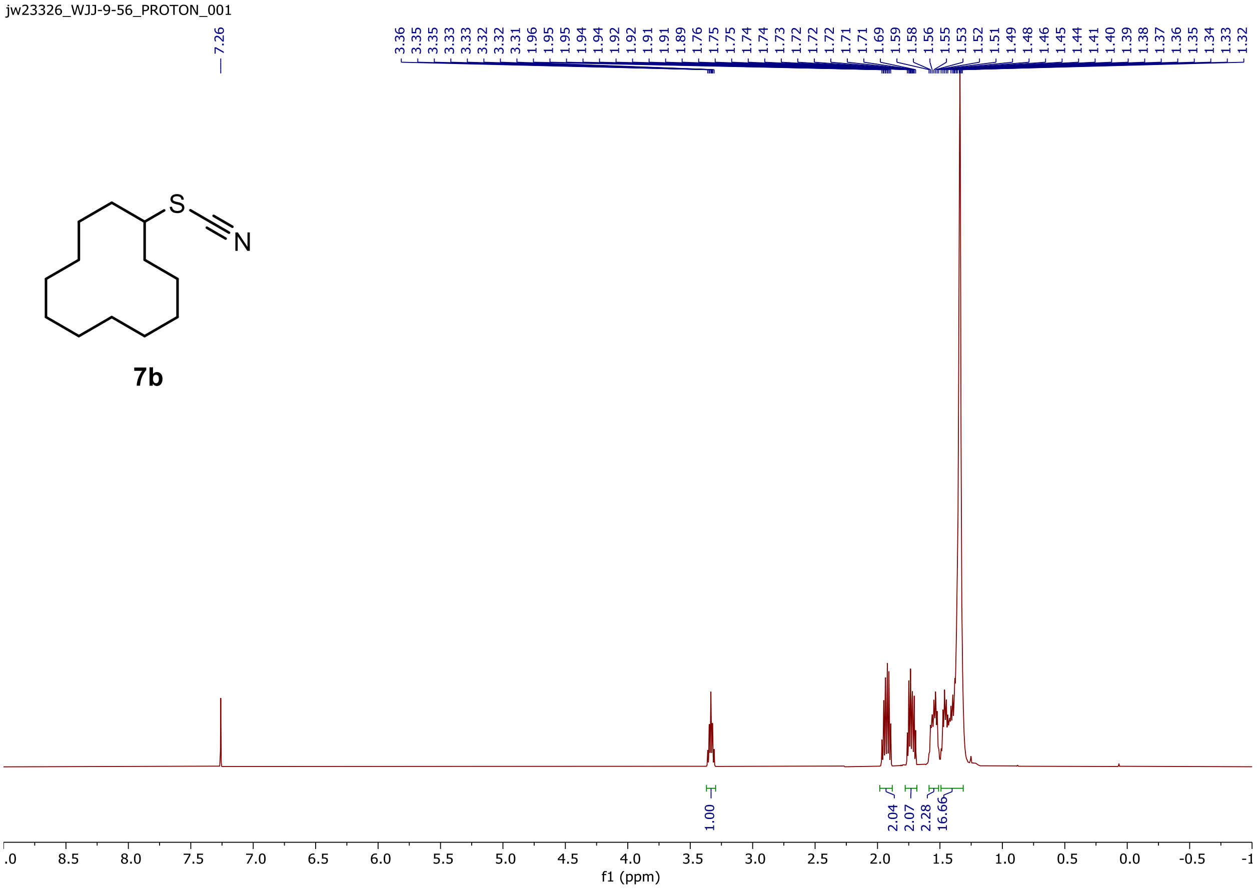

^{13}C NMR (125 MHz, $CDCl_3$) of **7b**

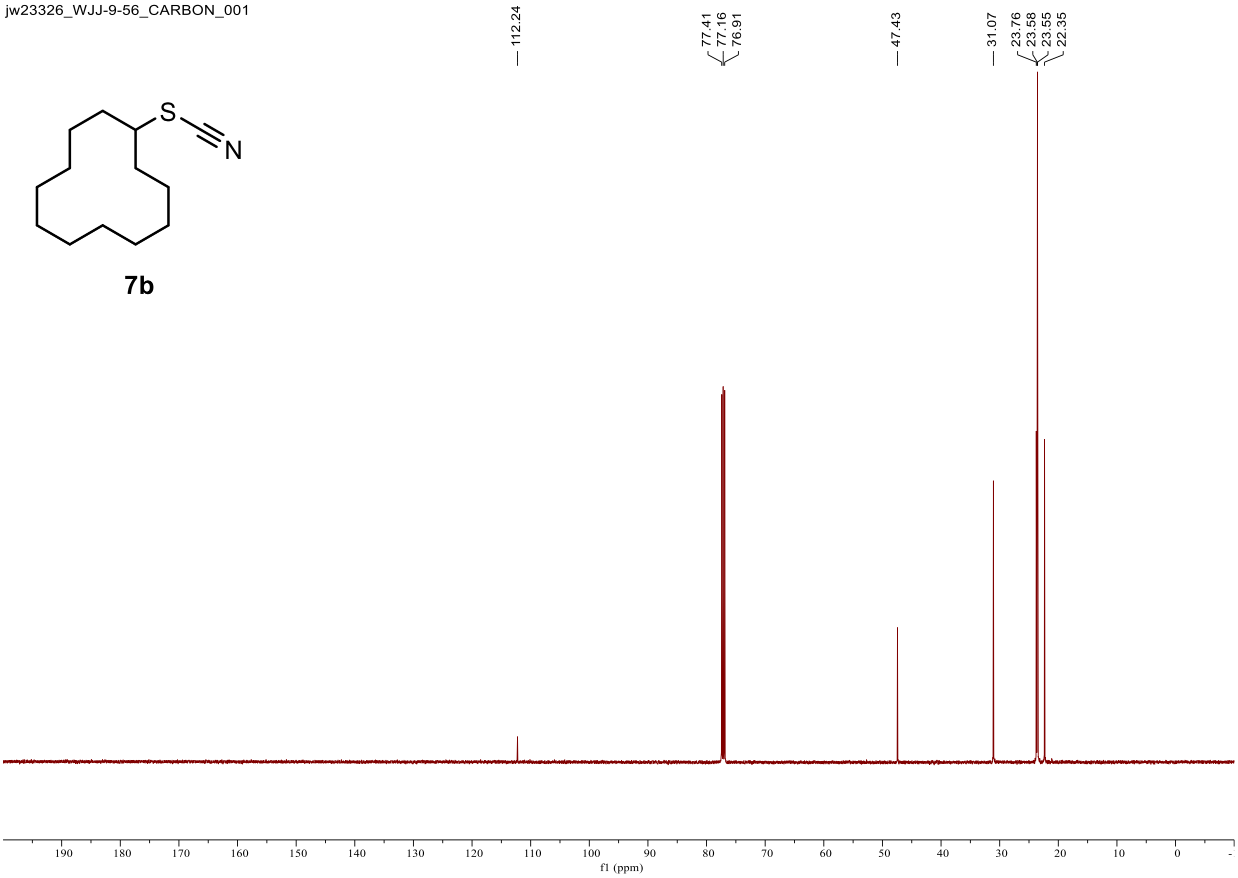

^{1}H NMR (500 MHz, $CDCl_3$) of **7c** (see procedure)

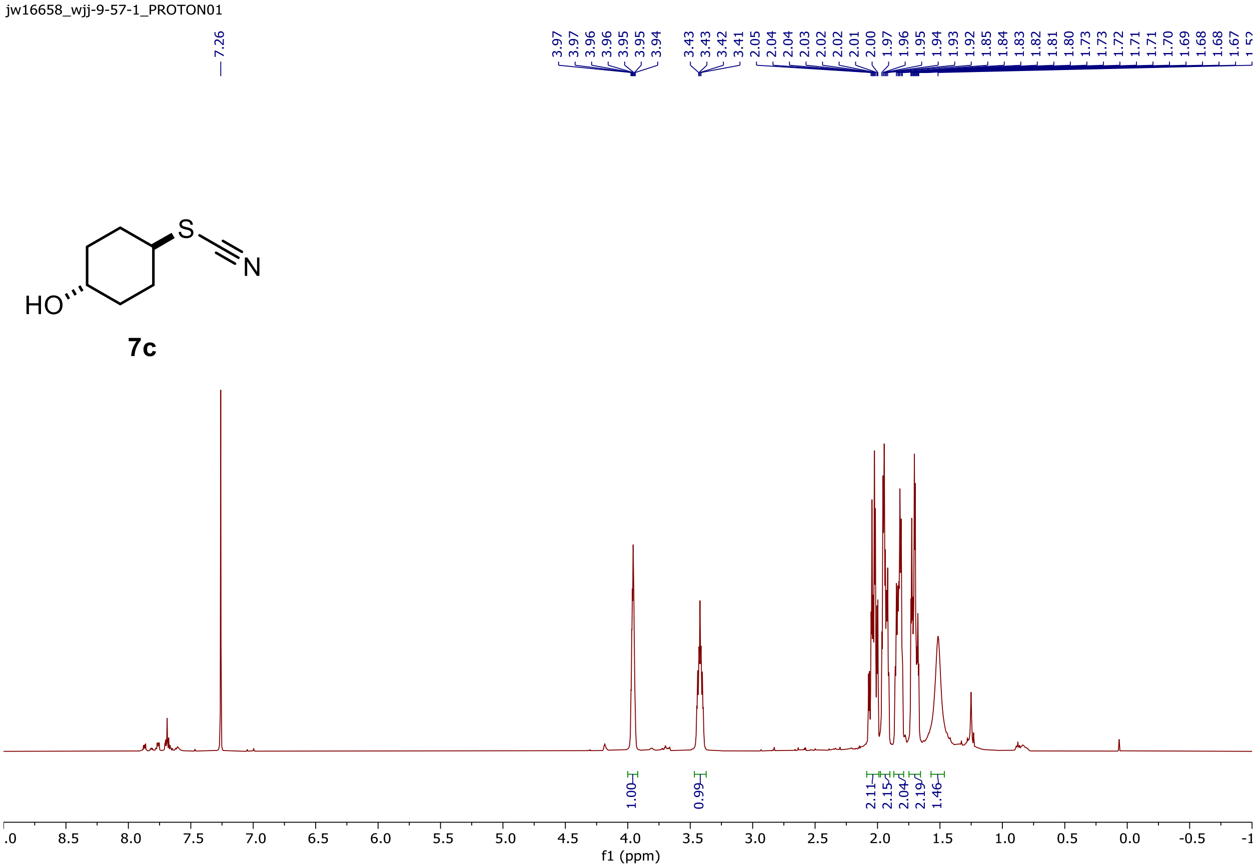

^{13}C NMR (125 MHz, $CDCl_3$) of **7c**

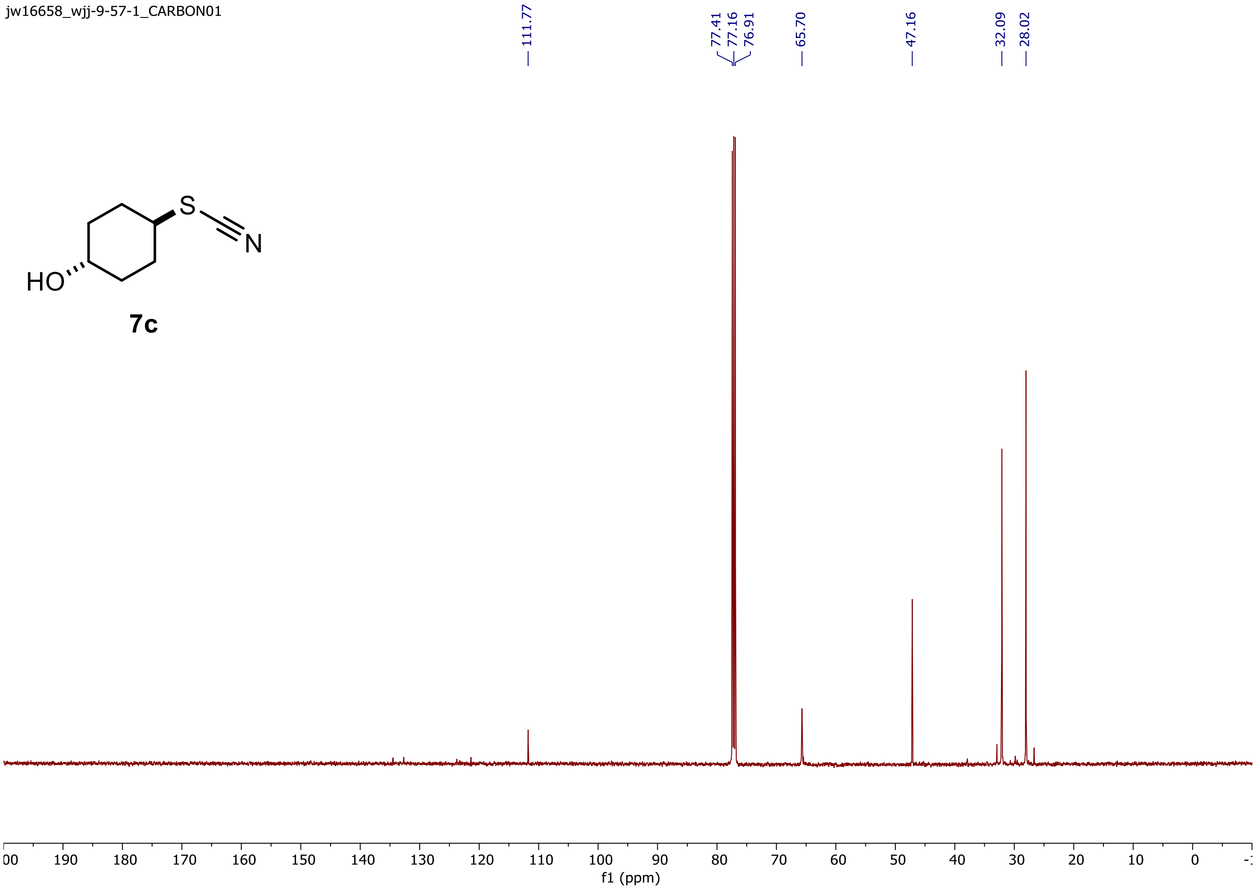

^{1}H NMR (500 MHz, $CDCl_3$) of **7c'** (*see procedure*)

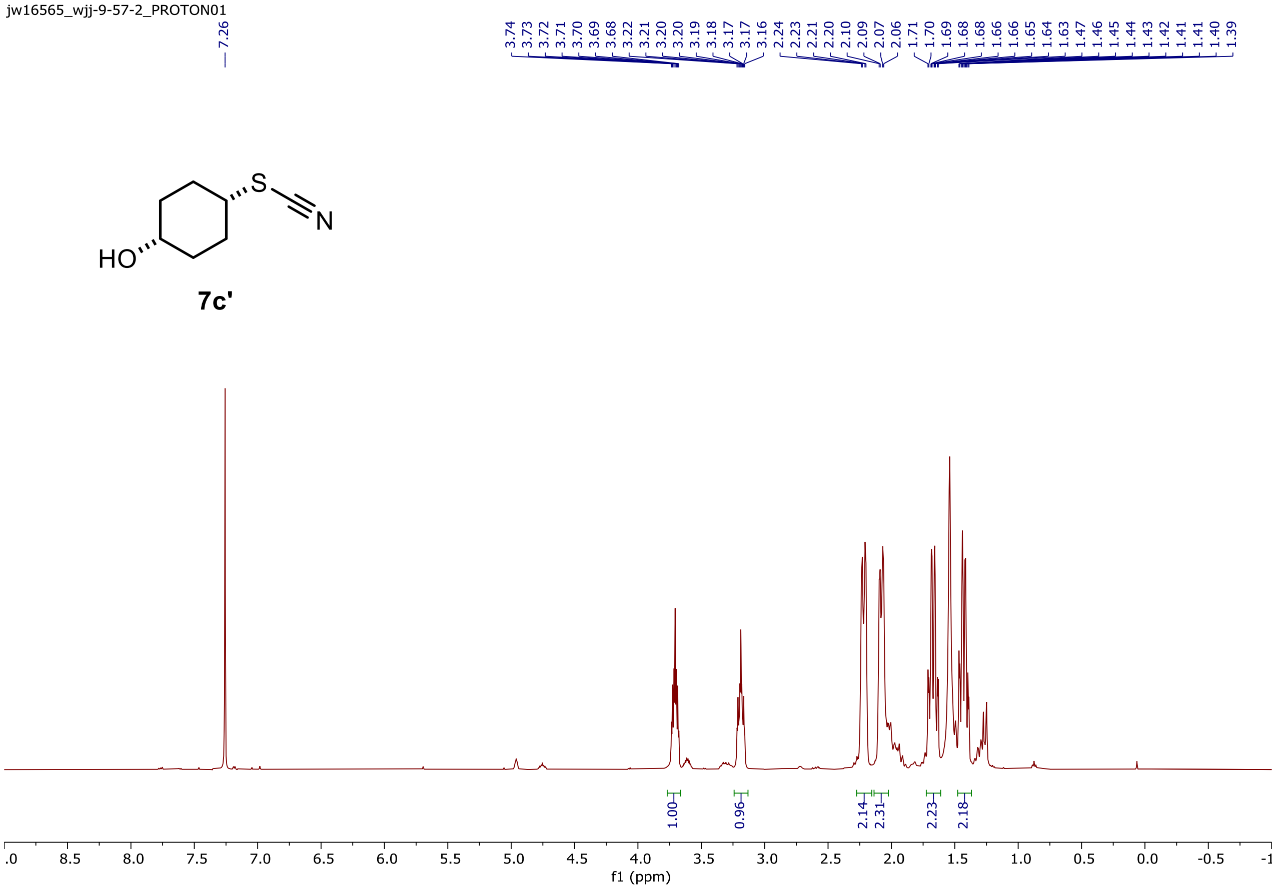

^{13}C NMR (125 MHz, $CDCl_3$) of **7c'**

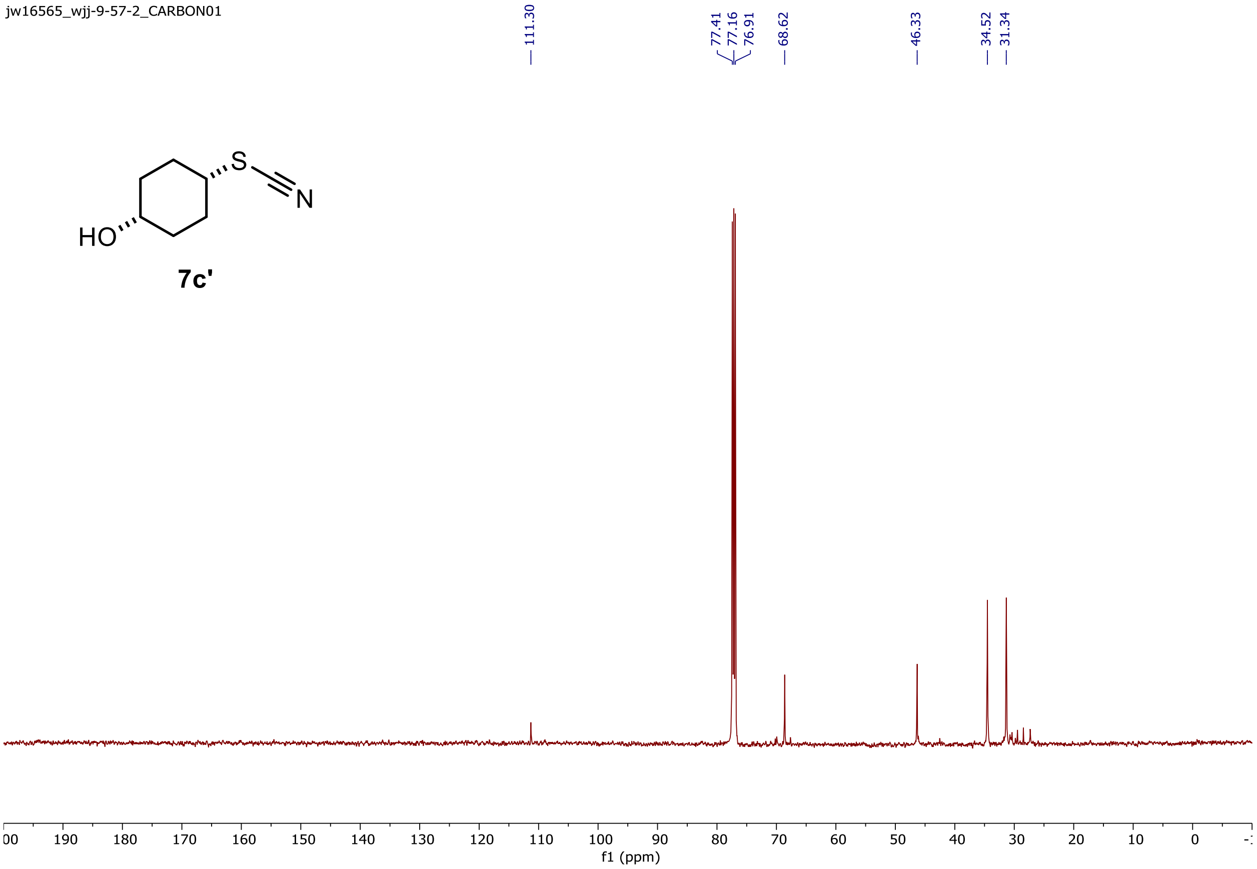

^{1}H NMR (400 MHz, $CDCl_3$) of **7h** (*see procedure*)

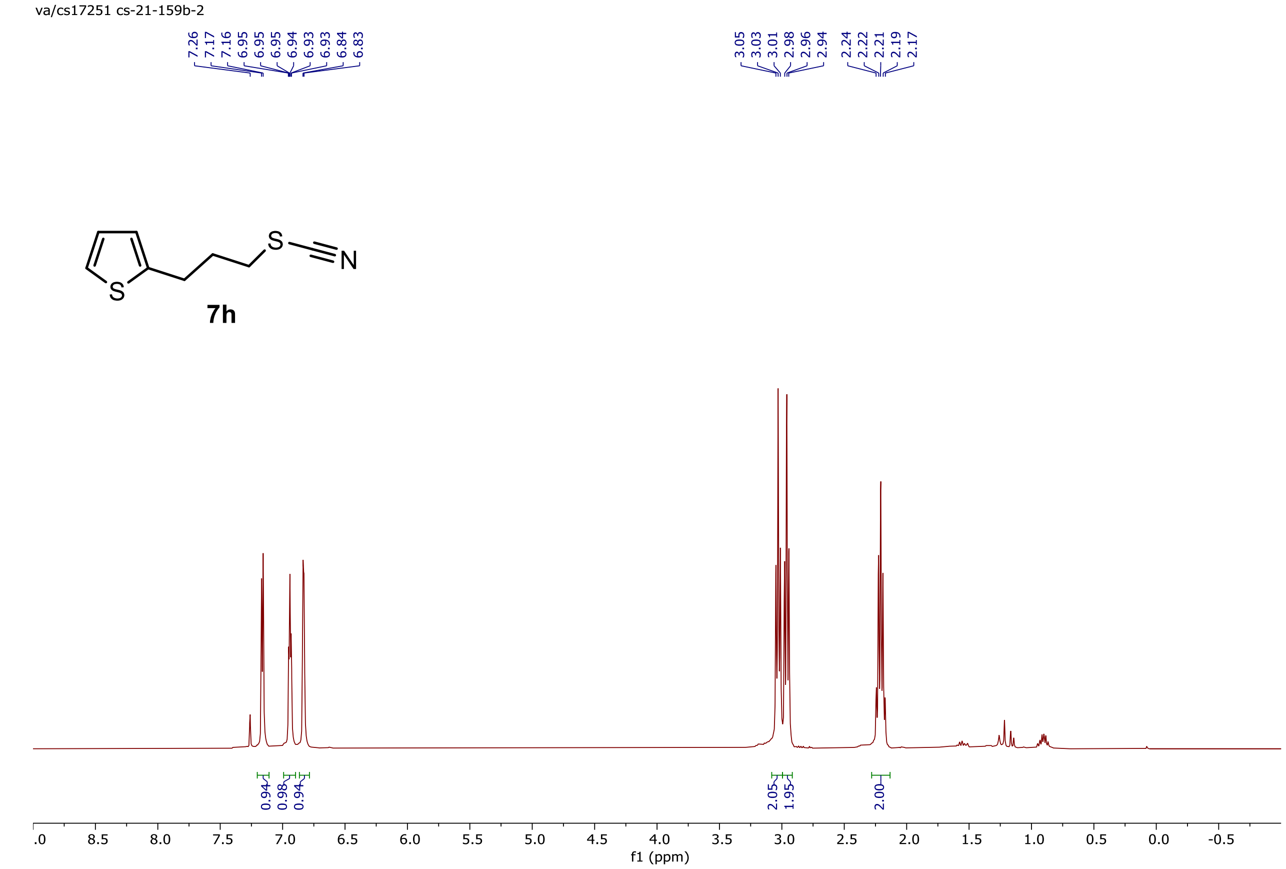

^{13}C NMR (100 MHz, $CDCl_3$) of **7h**

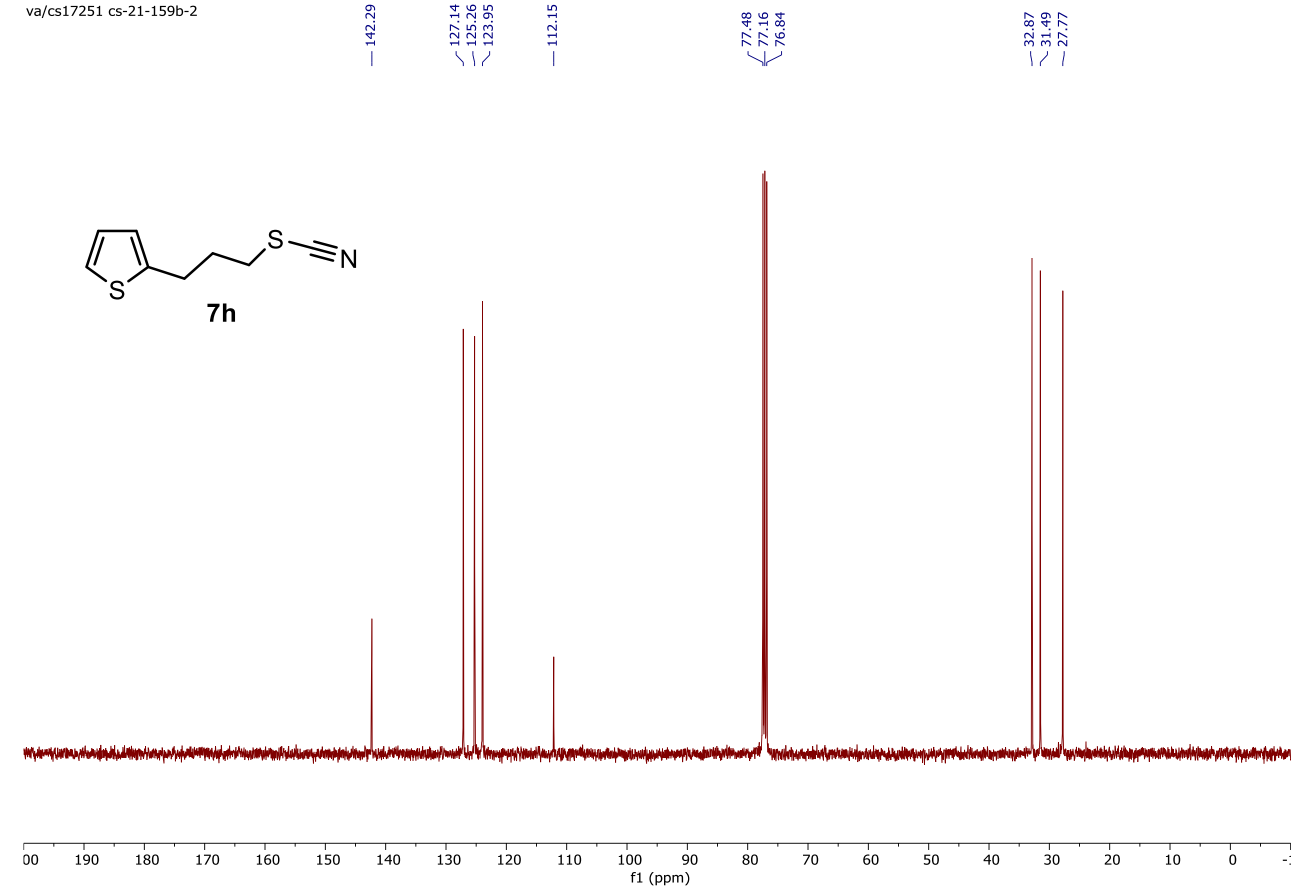

^{1}H NMR (500 MHz, $CDCl_3$) of **7j** (*see procedure*)

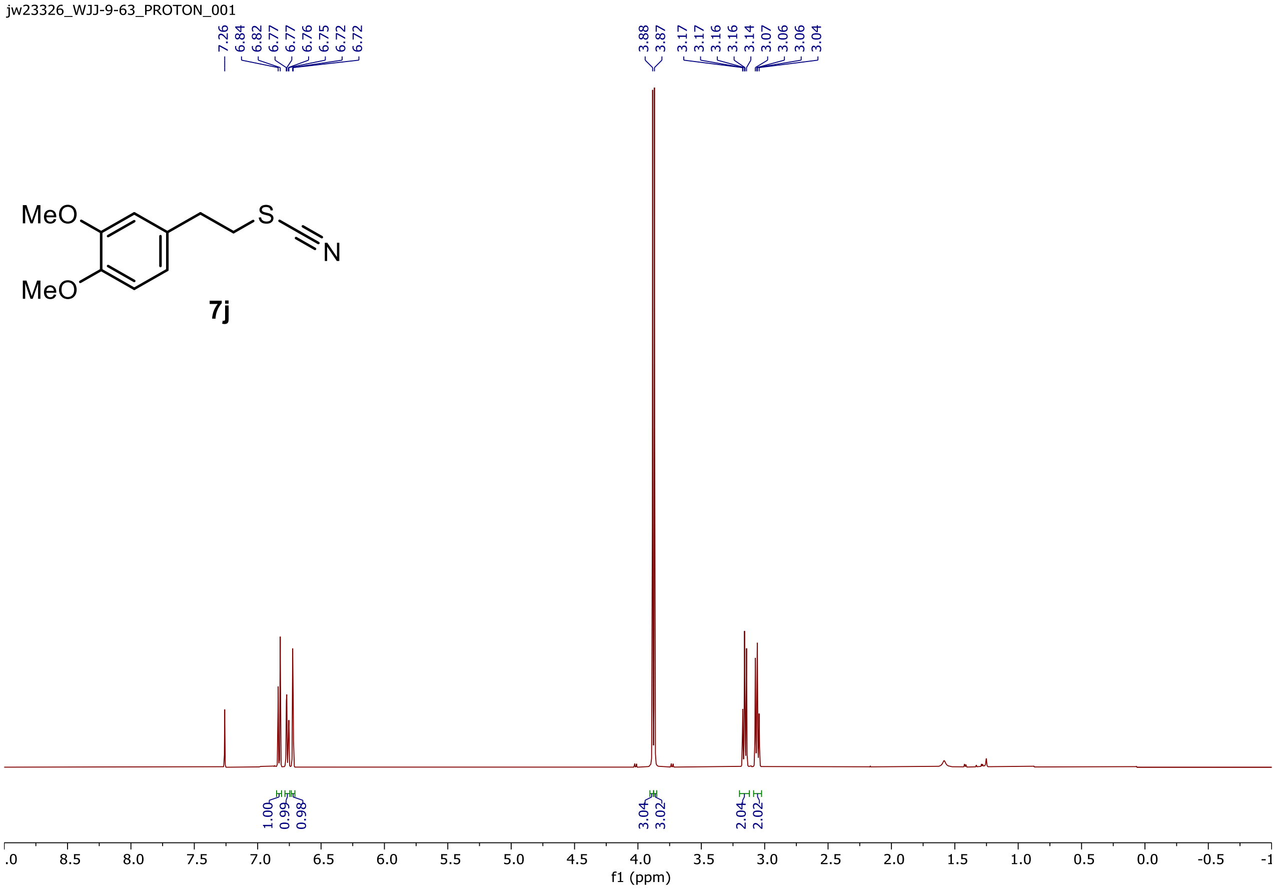

^{13}C NMR (125 MHz, $CDCl_3$) of **7j**

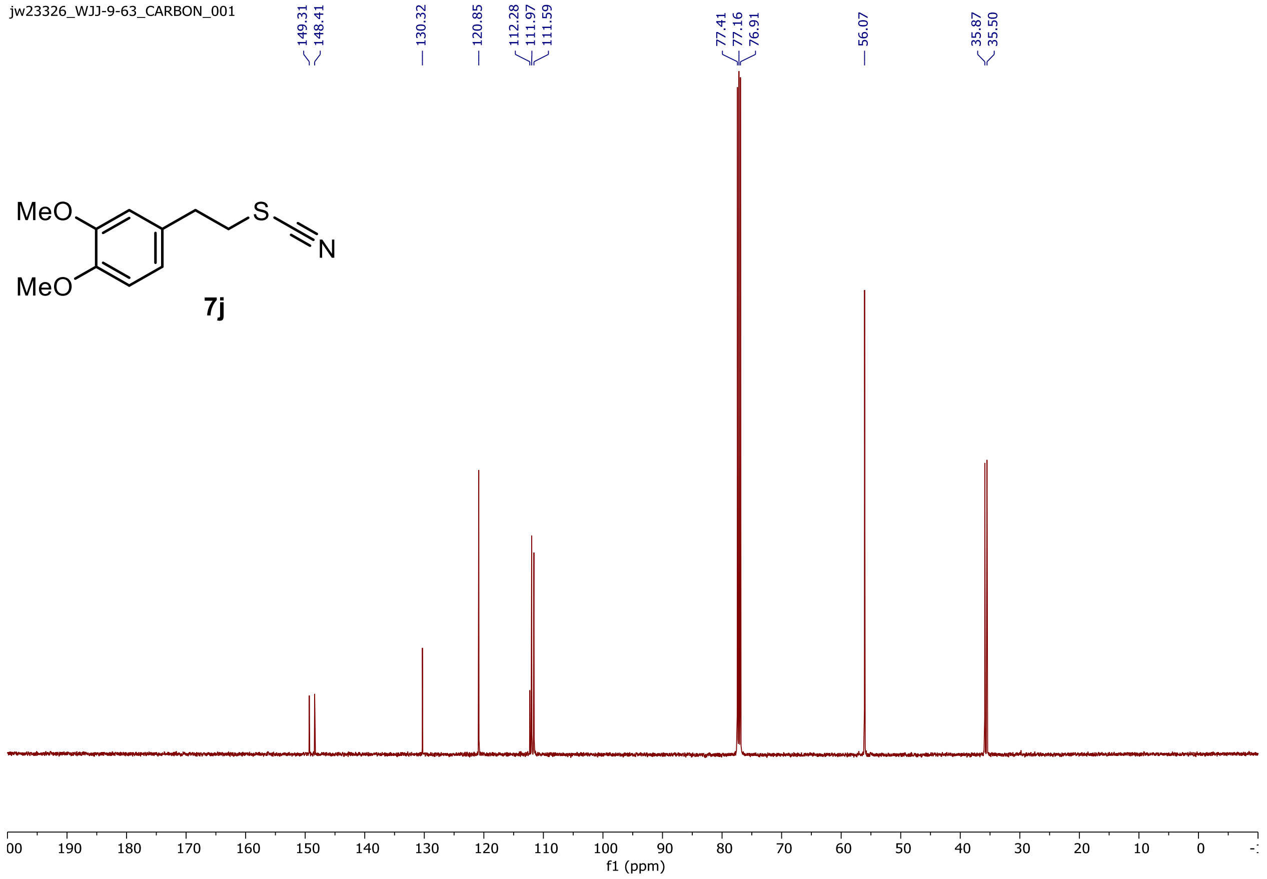

^{1}H NMR (500 MHz, $CDCl_3$) of **7j'** (*see procedure*)

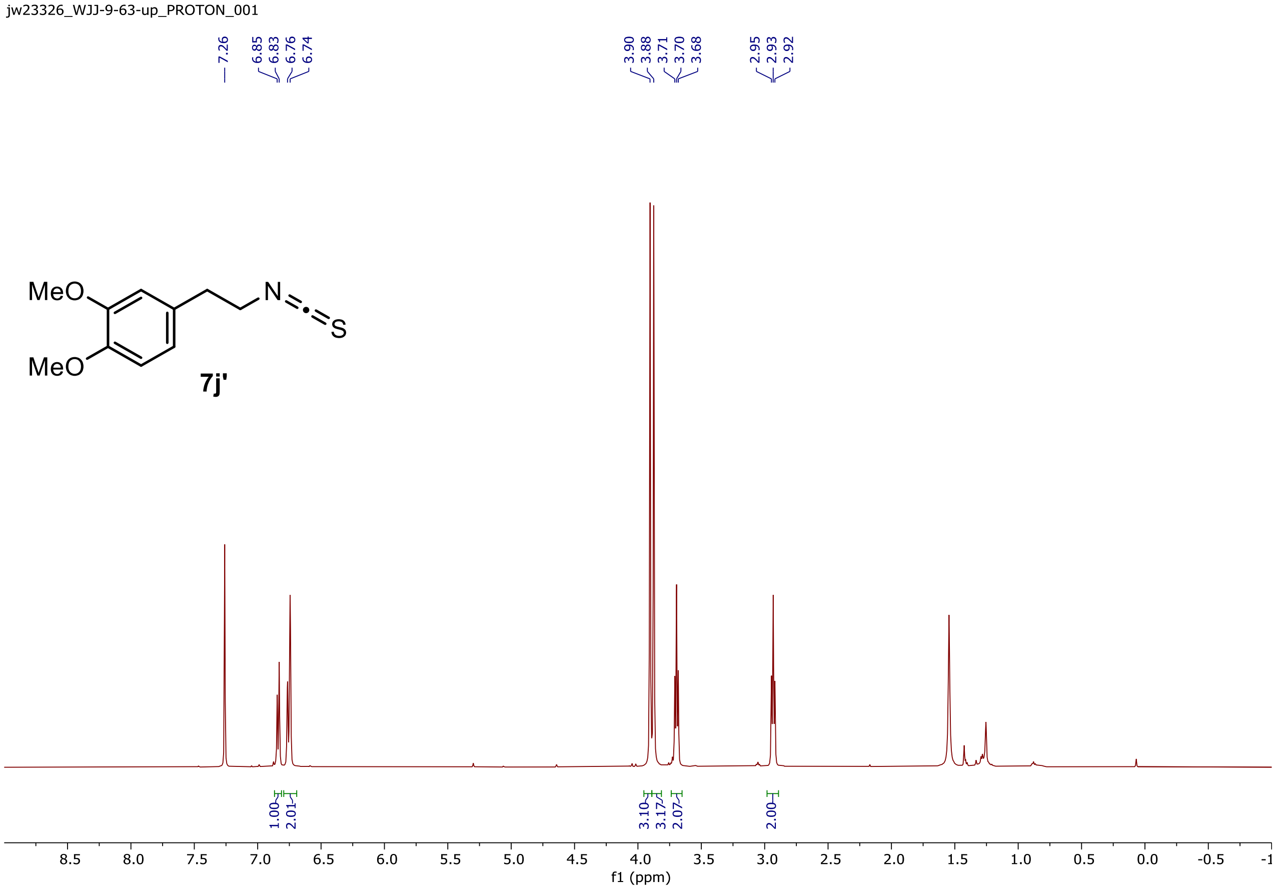

^{13}C NMR (125 MHz, $CDCl_3$) of **7j'**

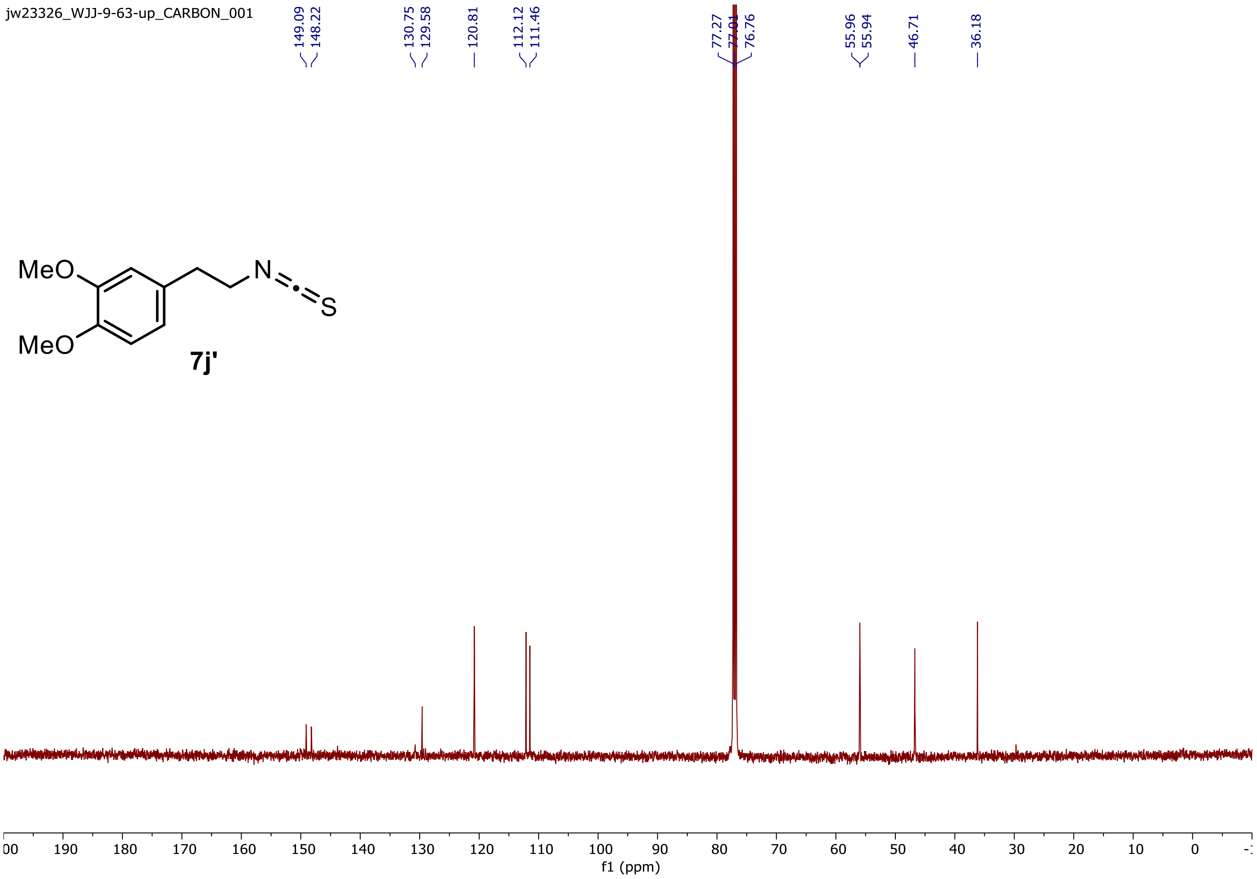

^{1}H NMR (500 MHz, $CDCl_3$) of **7k** (*see procedure*)

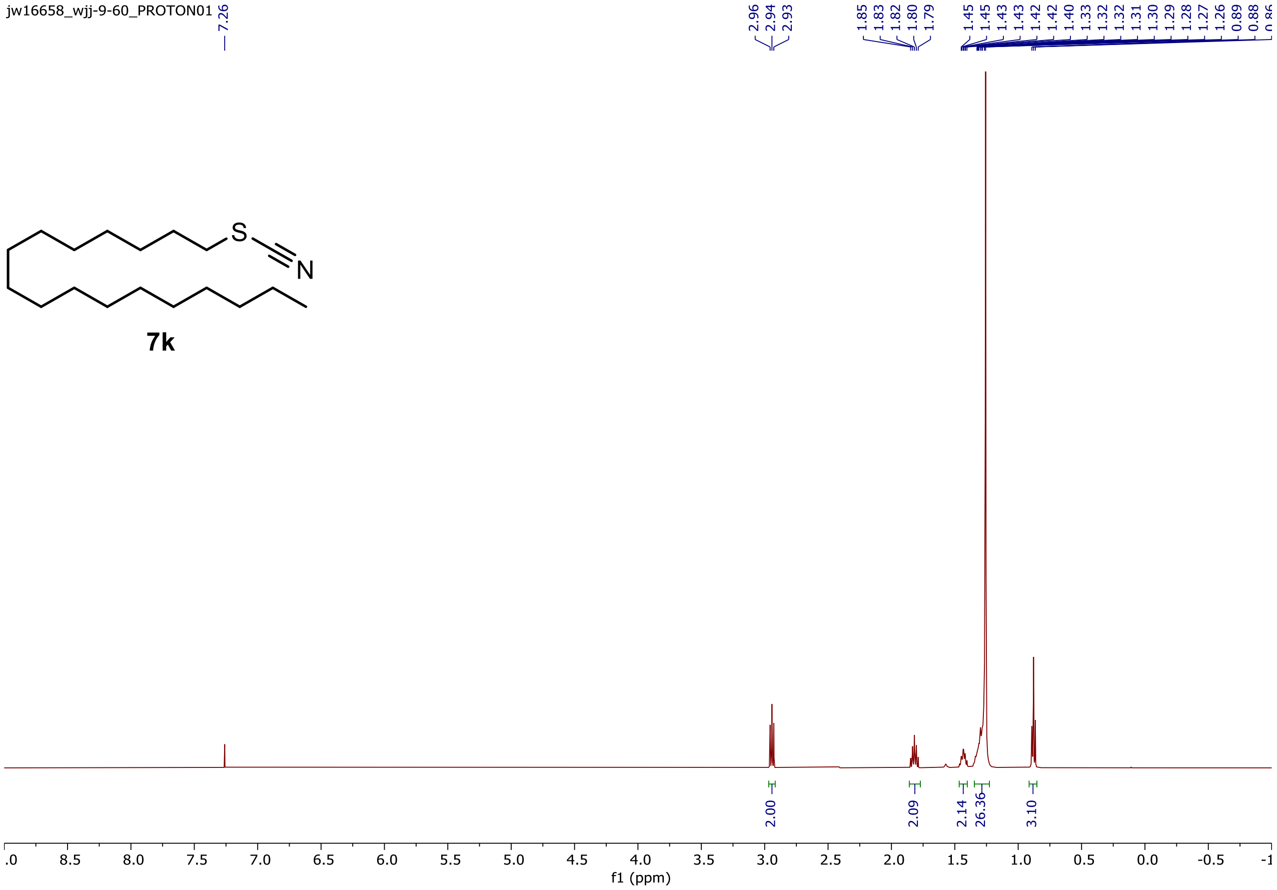

7k

^{13}C NMR (125 MHz, $CDCl_3$) of **7k**

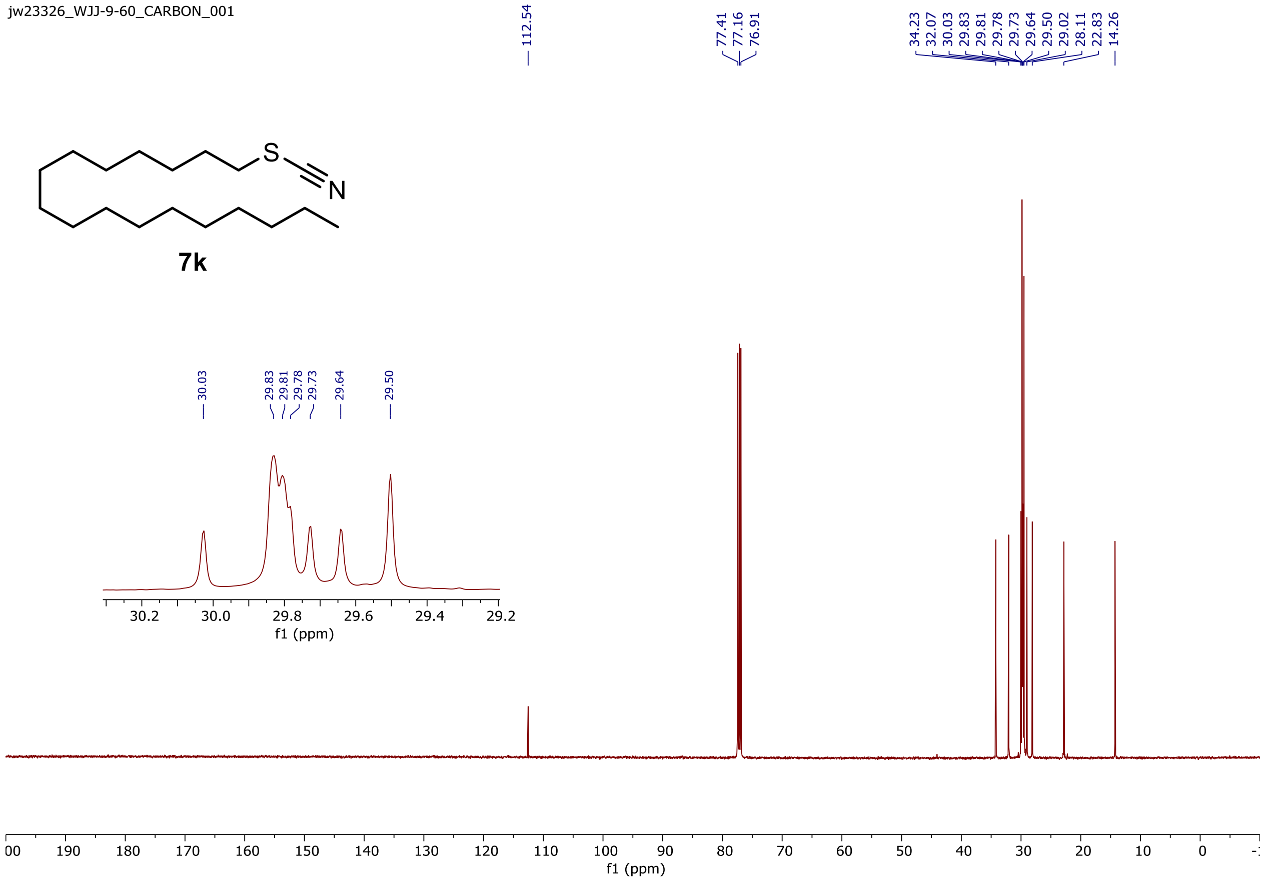

7k

^{1}H NMR (500 MHz, $CDCl_3$) of **7k'** (*see procedure*)

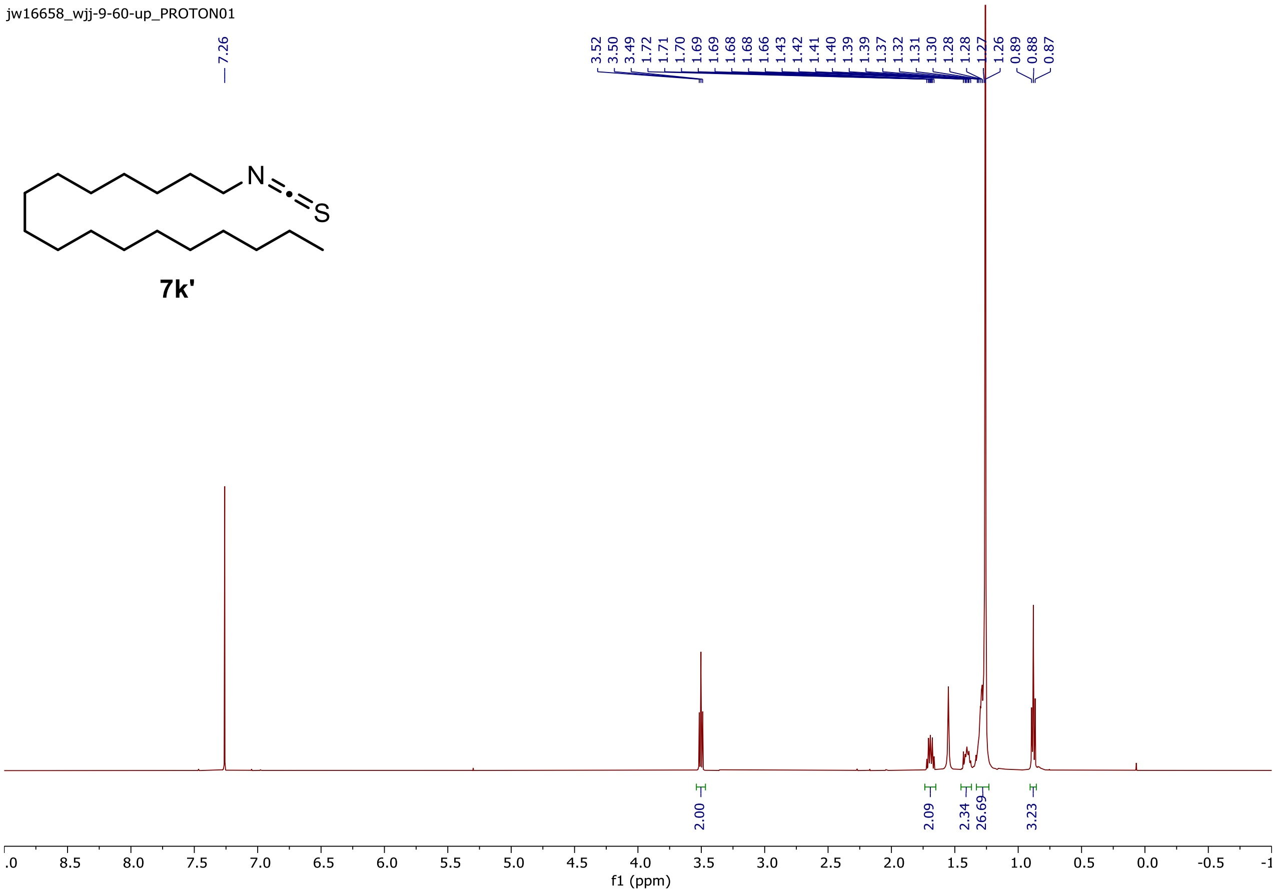

7k'

^{13}C NMR (125 MHz, $CDCl_3$) of **7k'**

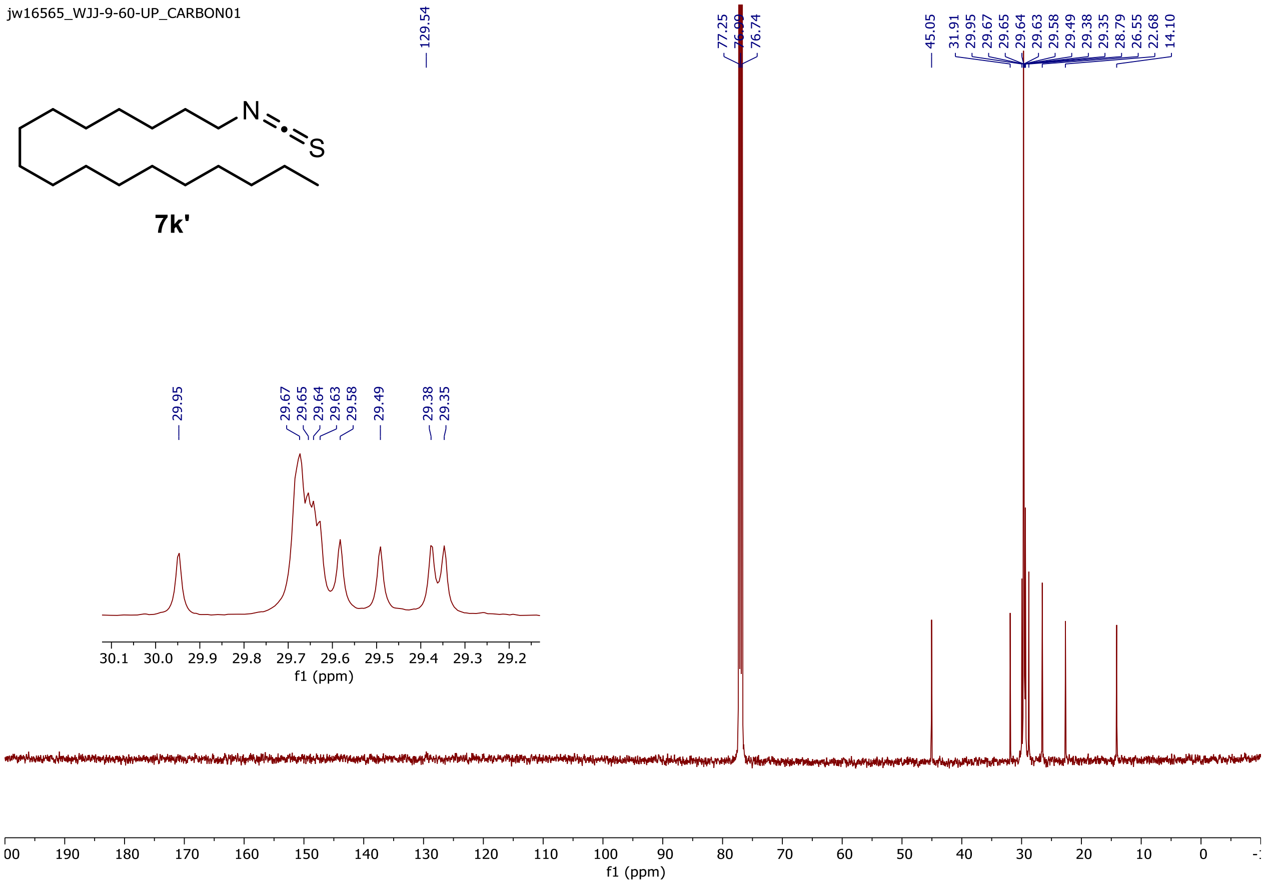

7k'

^{1}H NMR (500 MHz, $CDCl_3$) of **7l** (<u>*see procedure*</u>)

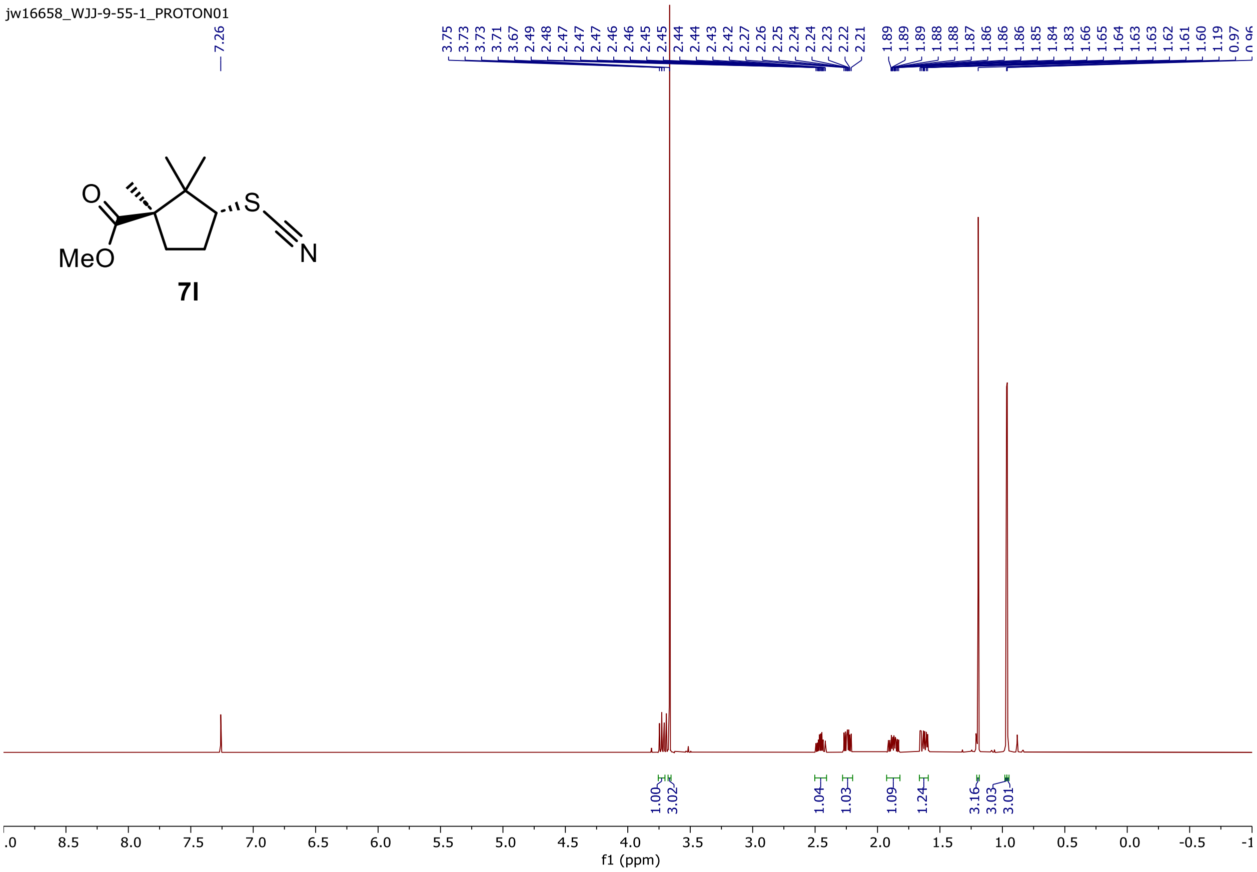

^{13}C NMR (125 MHz, $CDCl_3$) of **7l**

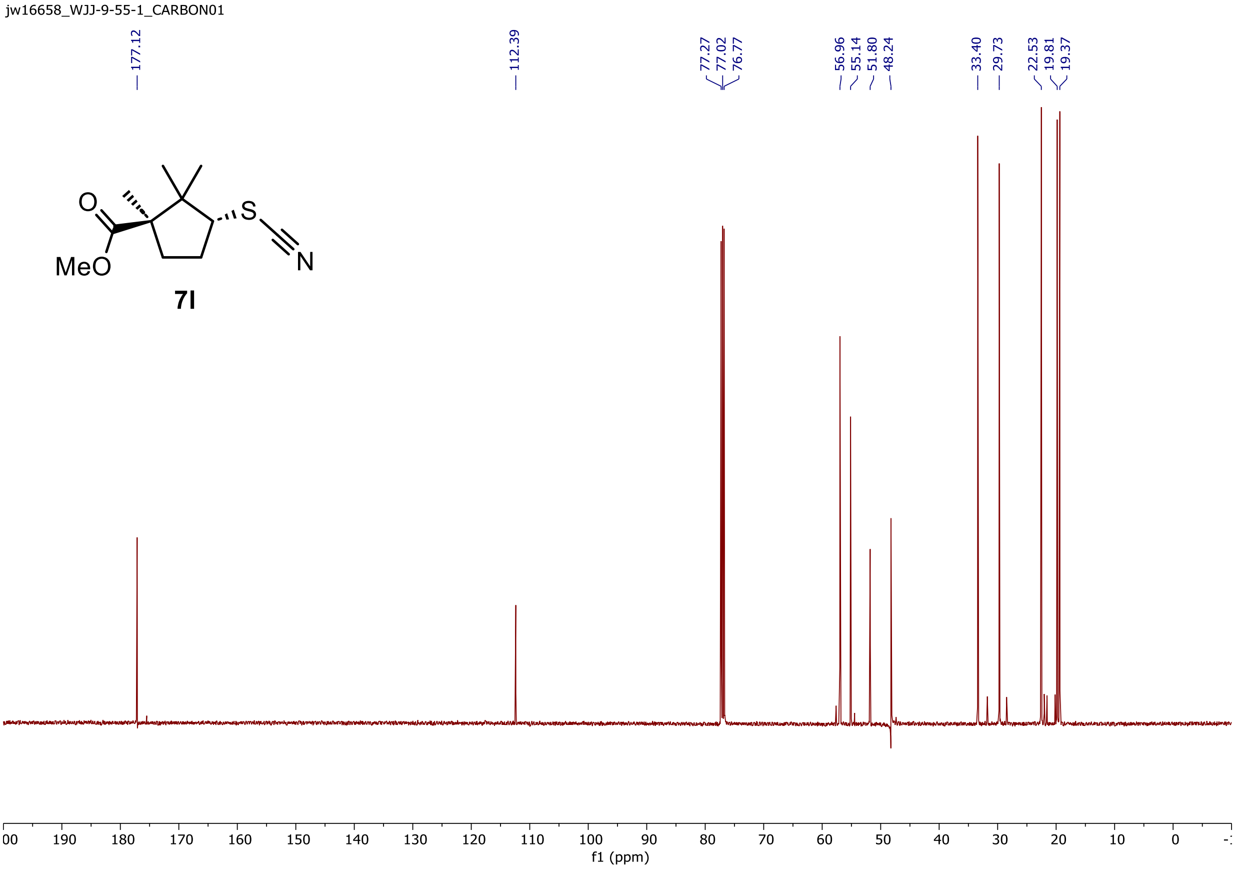

^{1}H NMR (500 MHz, $CDCl_3$) of **7l'** (*see procedure*)

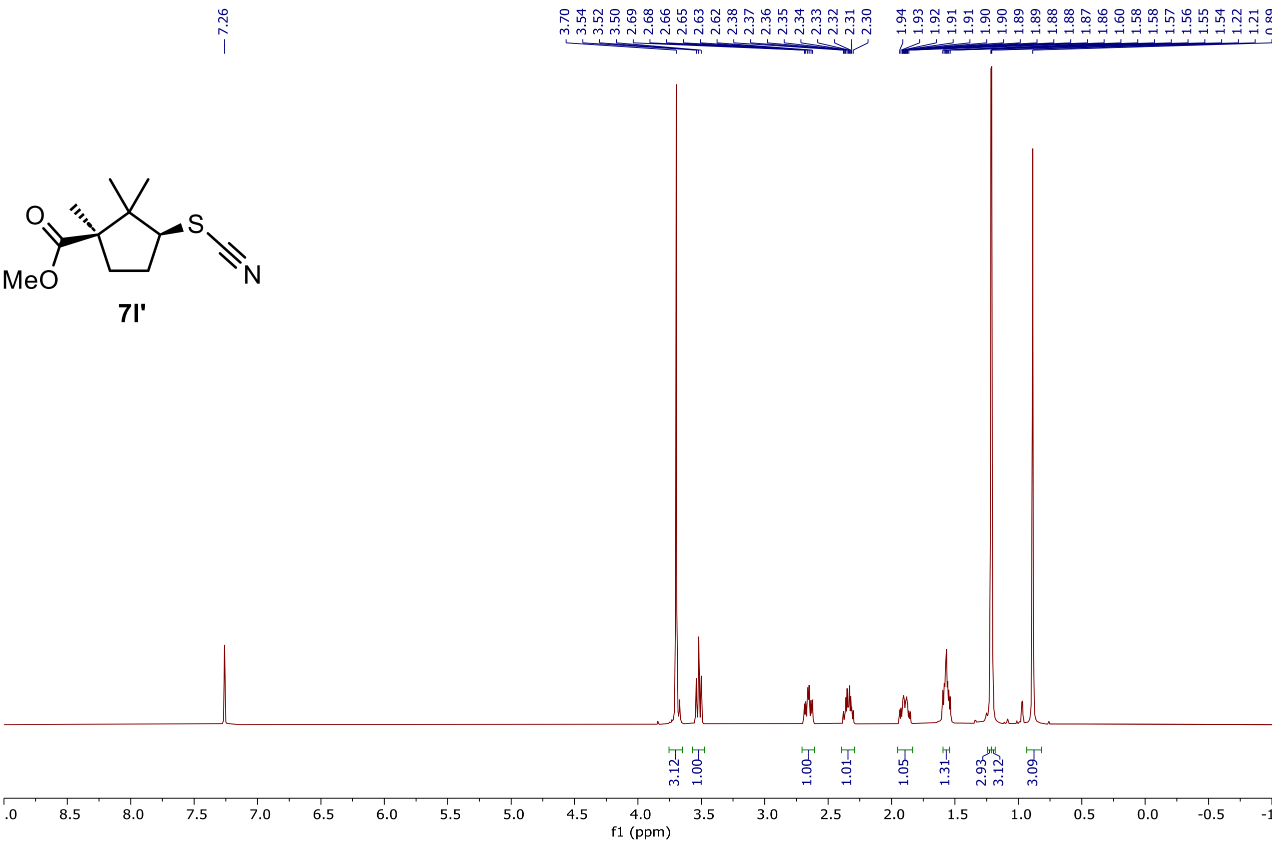

^{13}C NMR (125 MHz, $CDCl_3$) of **7l'**

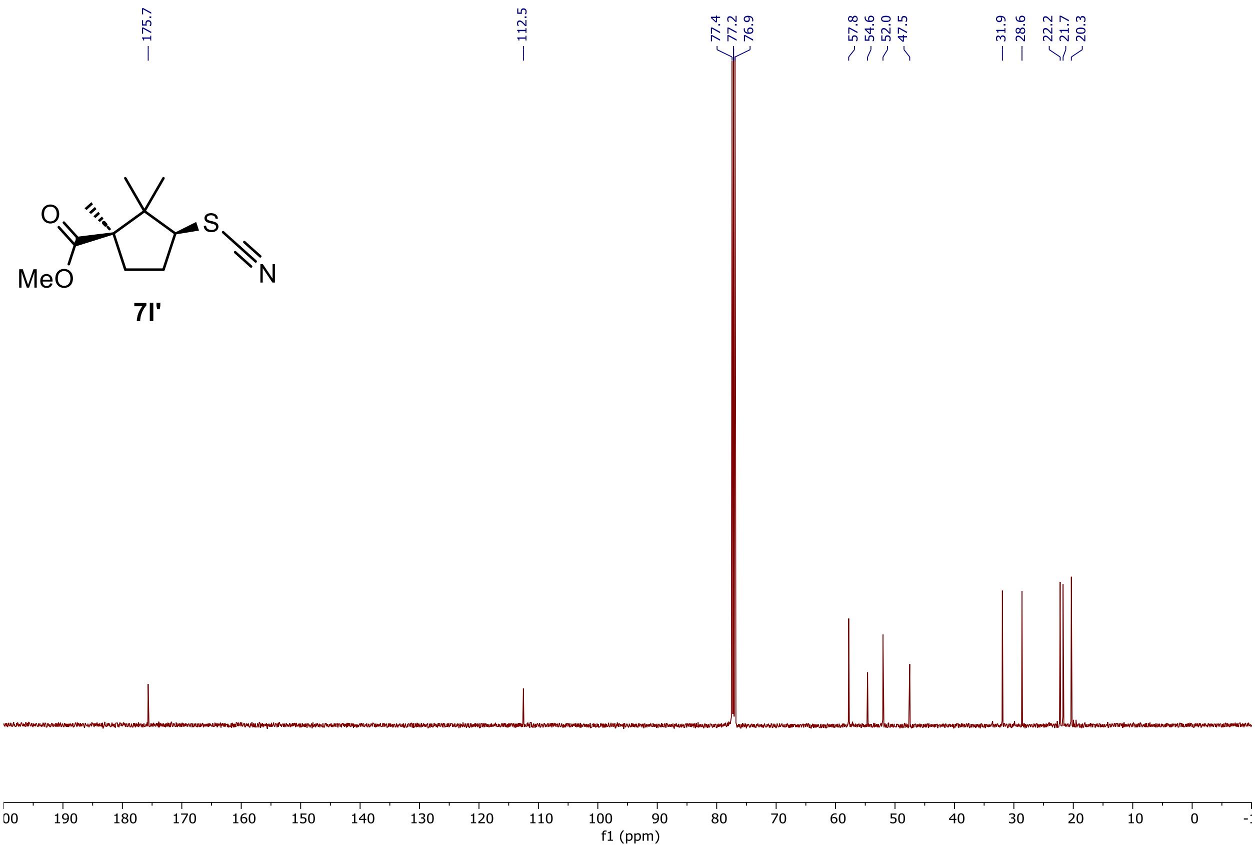

^{1}H NMR (500 MHz, $CDCl_3$) of **7m** (*see procedure*)

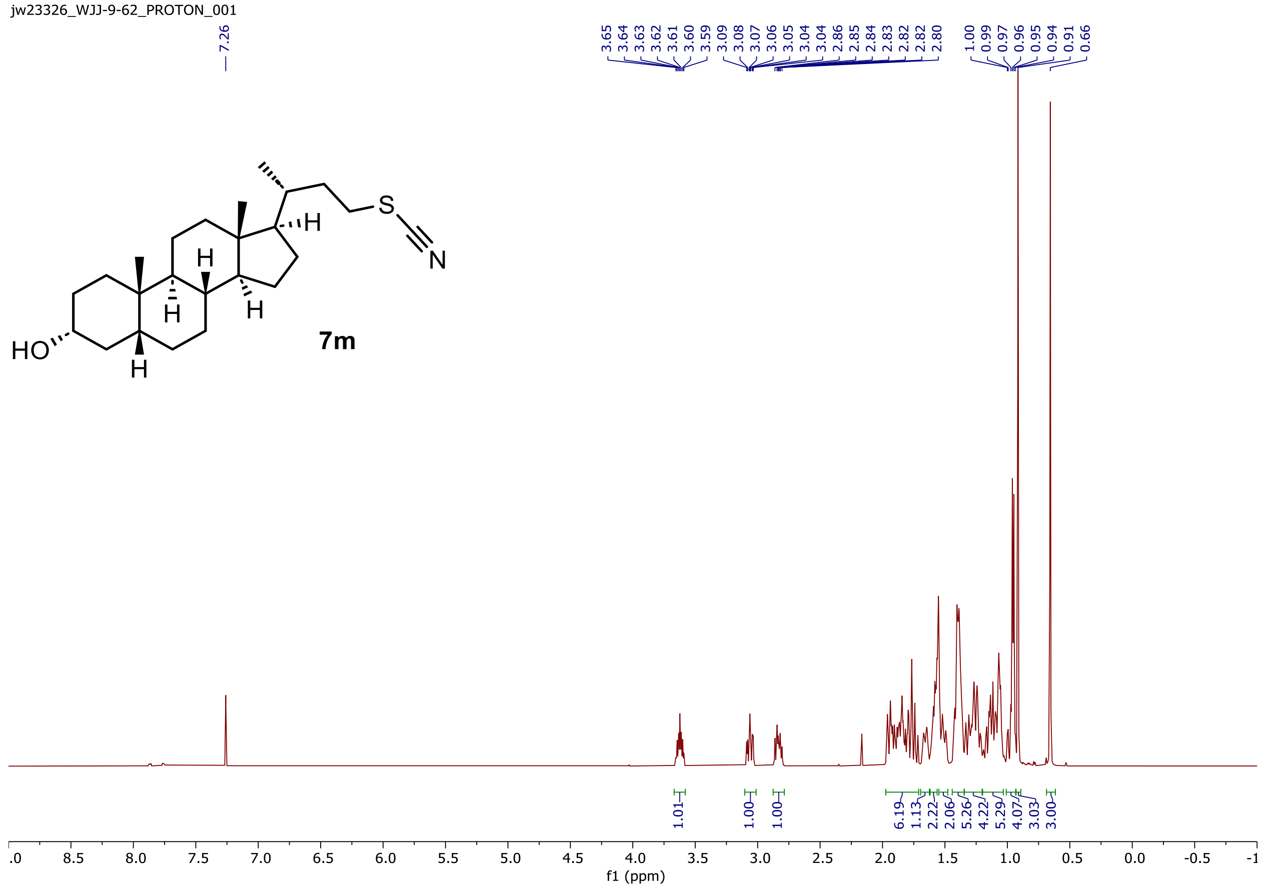

^{13}C NMR (125 MHz, $CDCl_3$) of **7m**

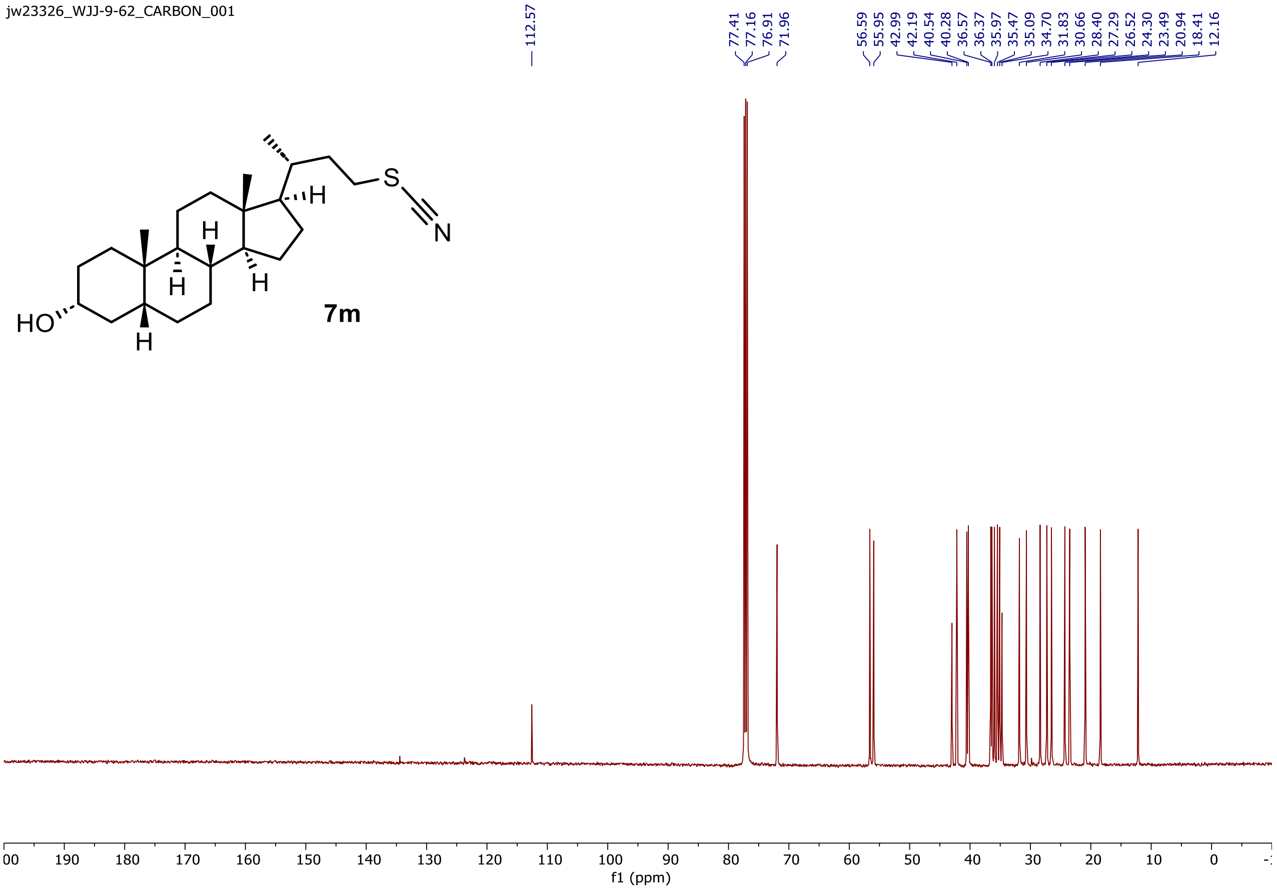

^{1}H–^{1}H COSY (CDCl$_3$) of **7m**

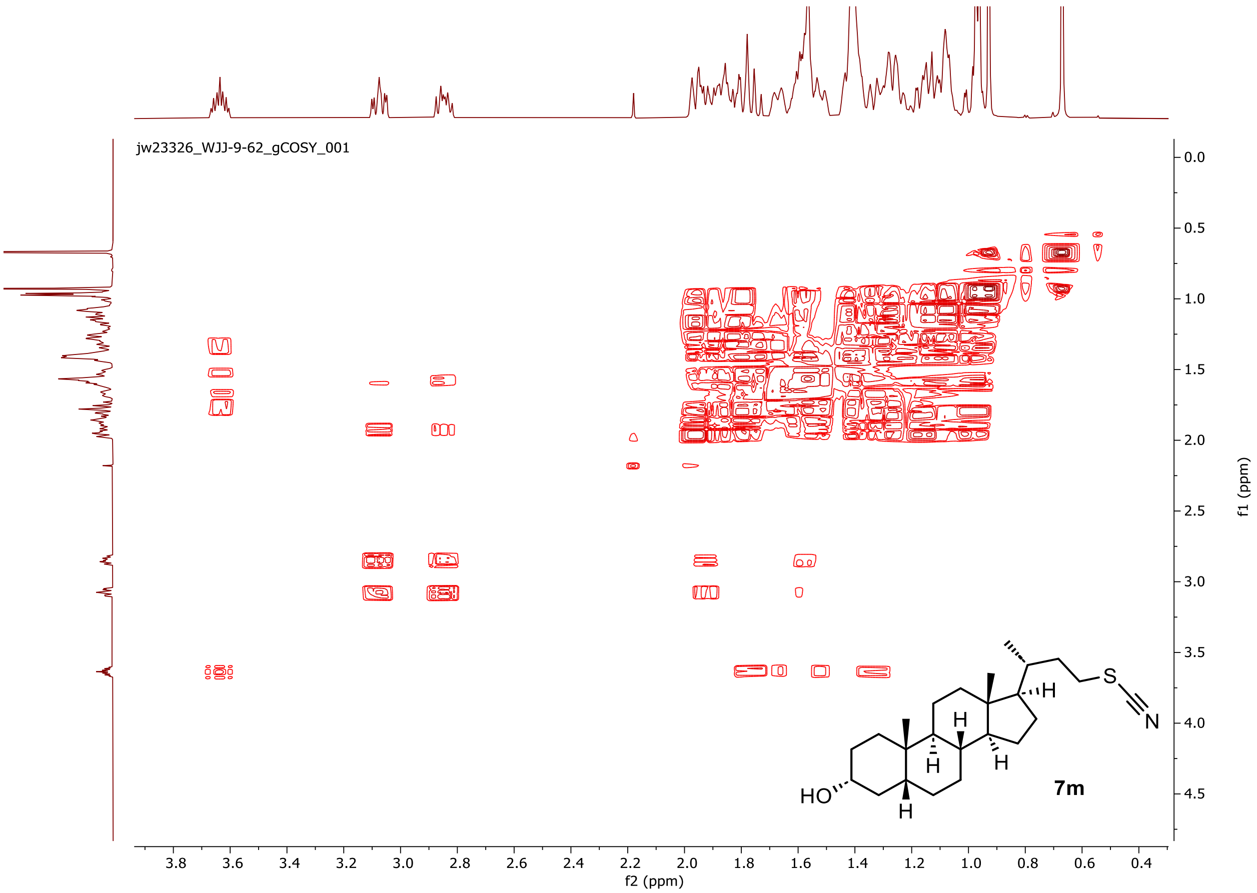

^{1}H–^{13}C HSQC (CDCl$_3$) of **7m**

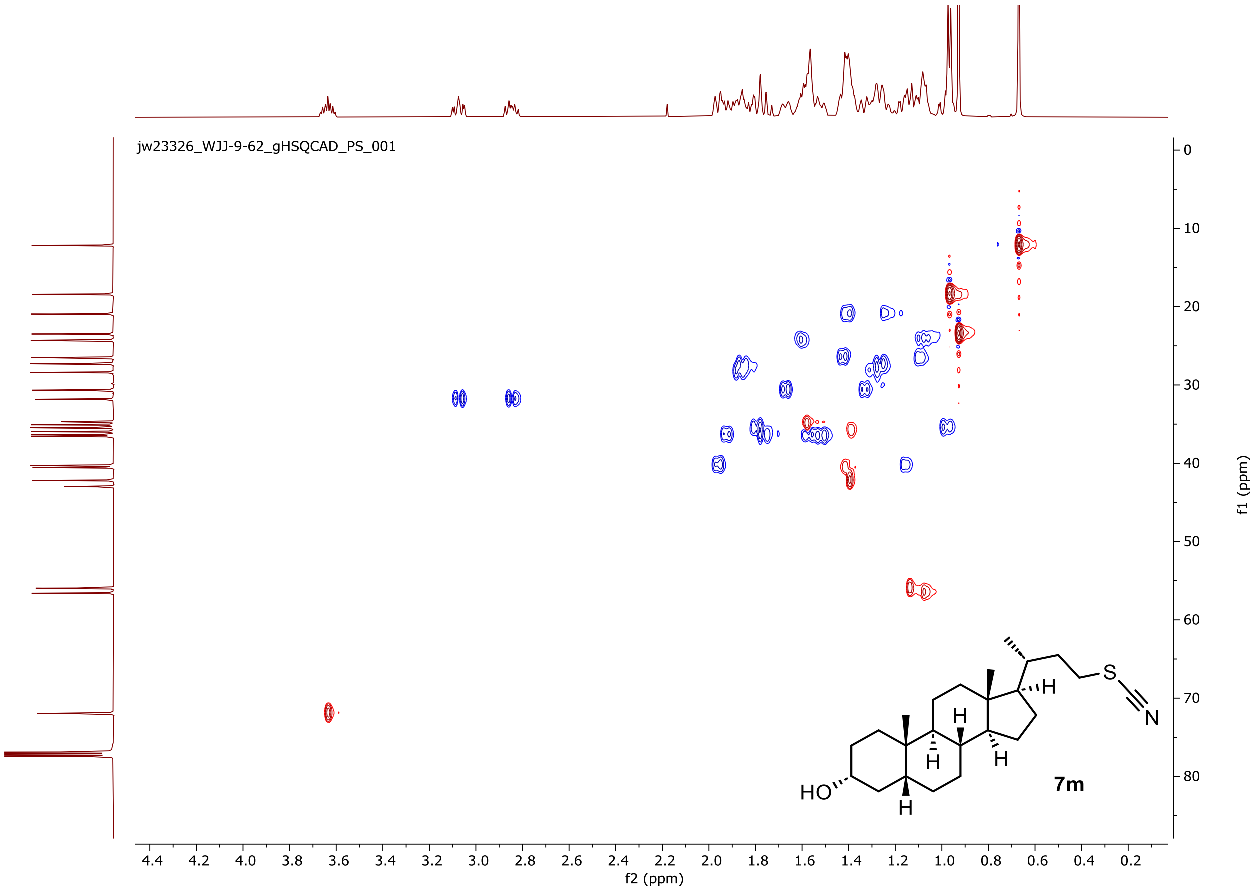

^{1}H NMR (500 MHz, $CDCl_3$) of **7m'** (*see procedure*)

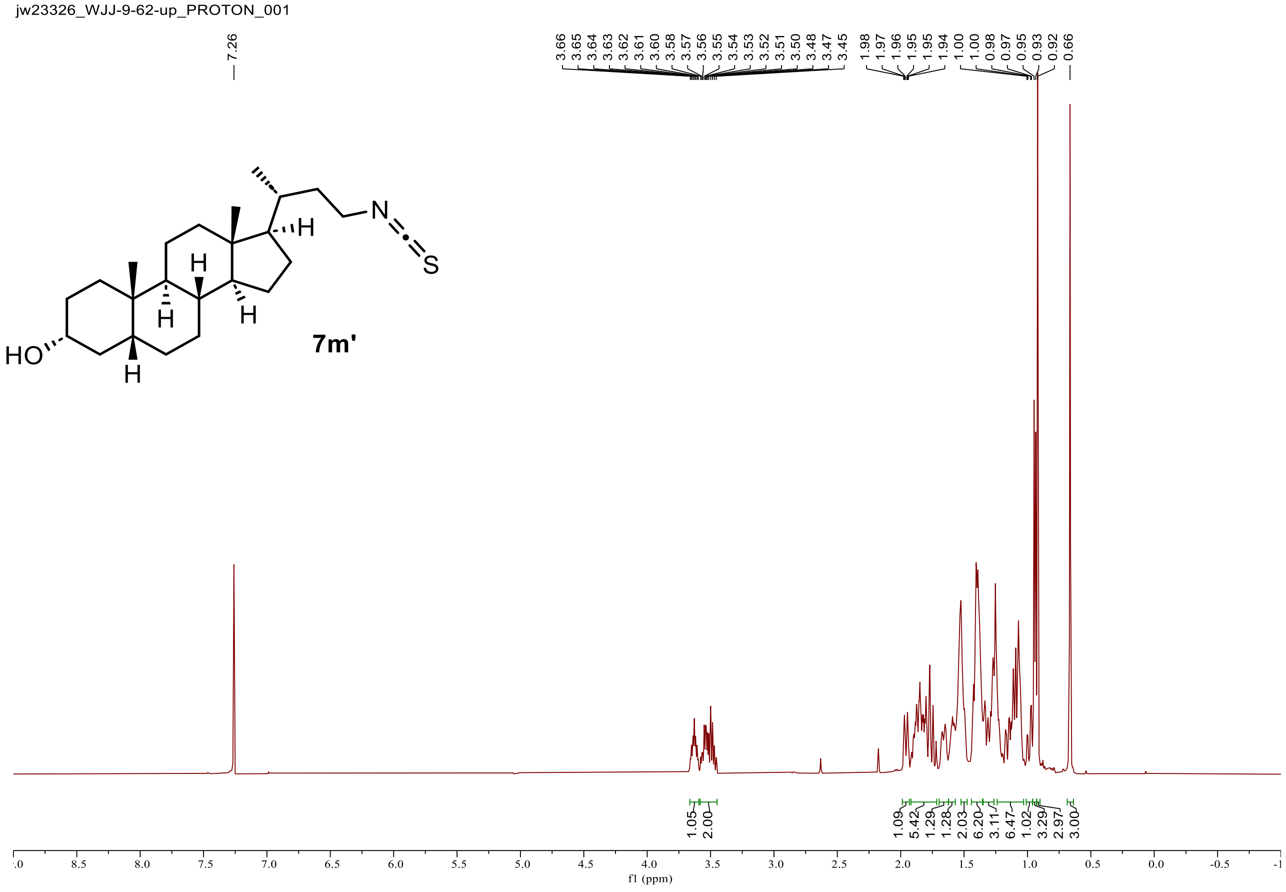

^{13}C NMR (125 MHz, $CDCl_3$) of **7m'**

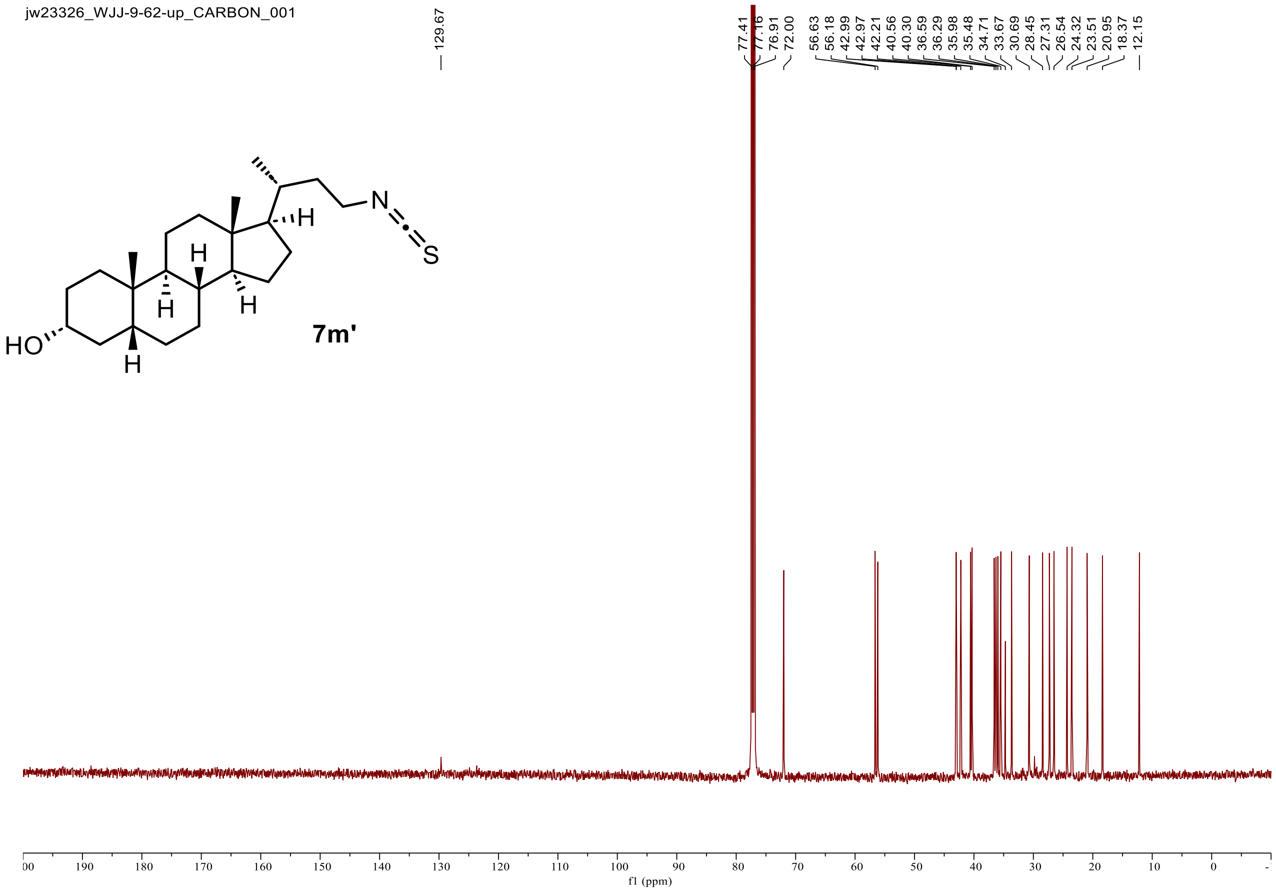

^{1}H–^{13}C HSQC ($CDCl_3$) of **7m'**

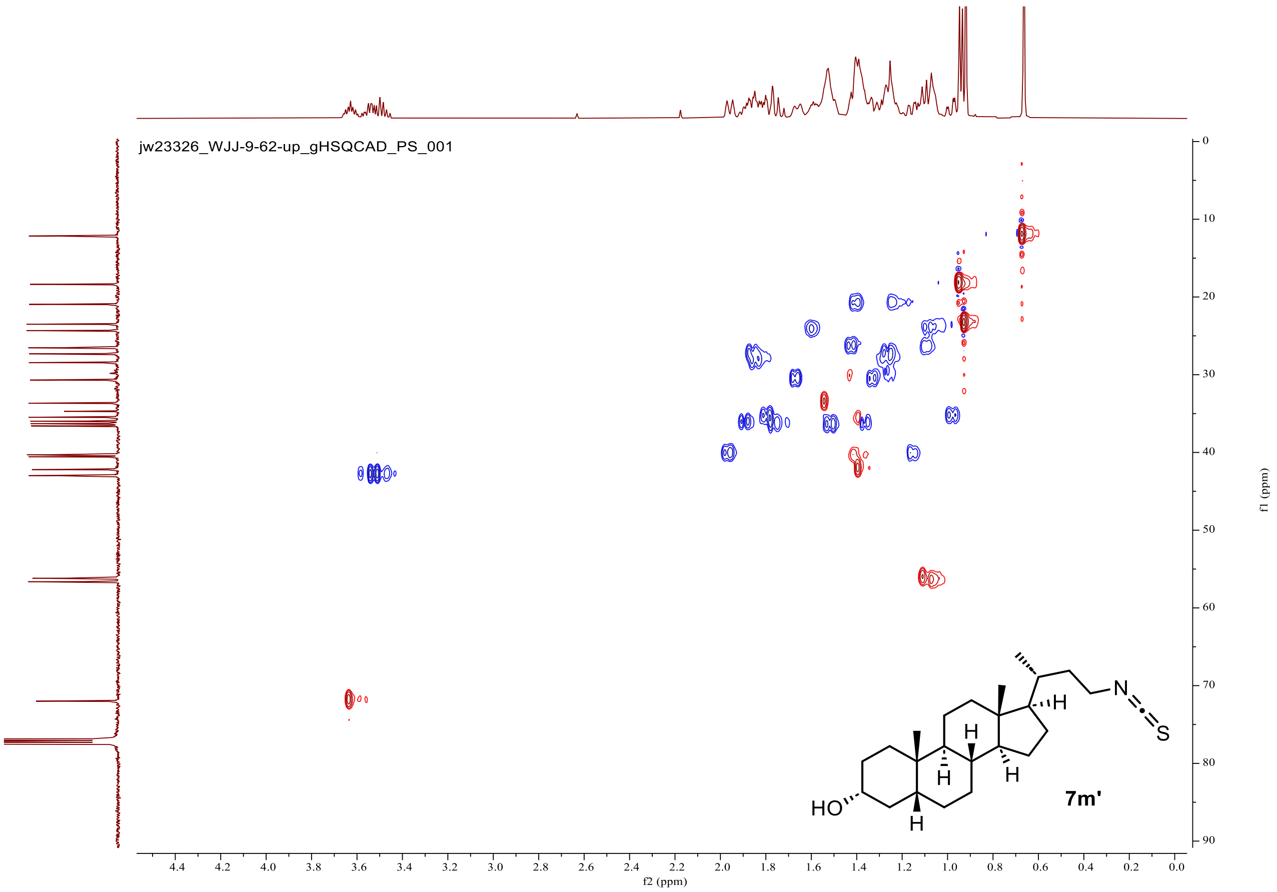

^{1}H NMR (500 MHz, $CDCl_3$) of **7n** (*see procedure*)

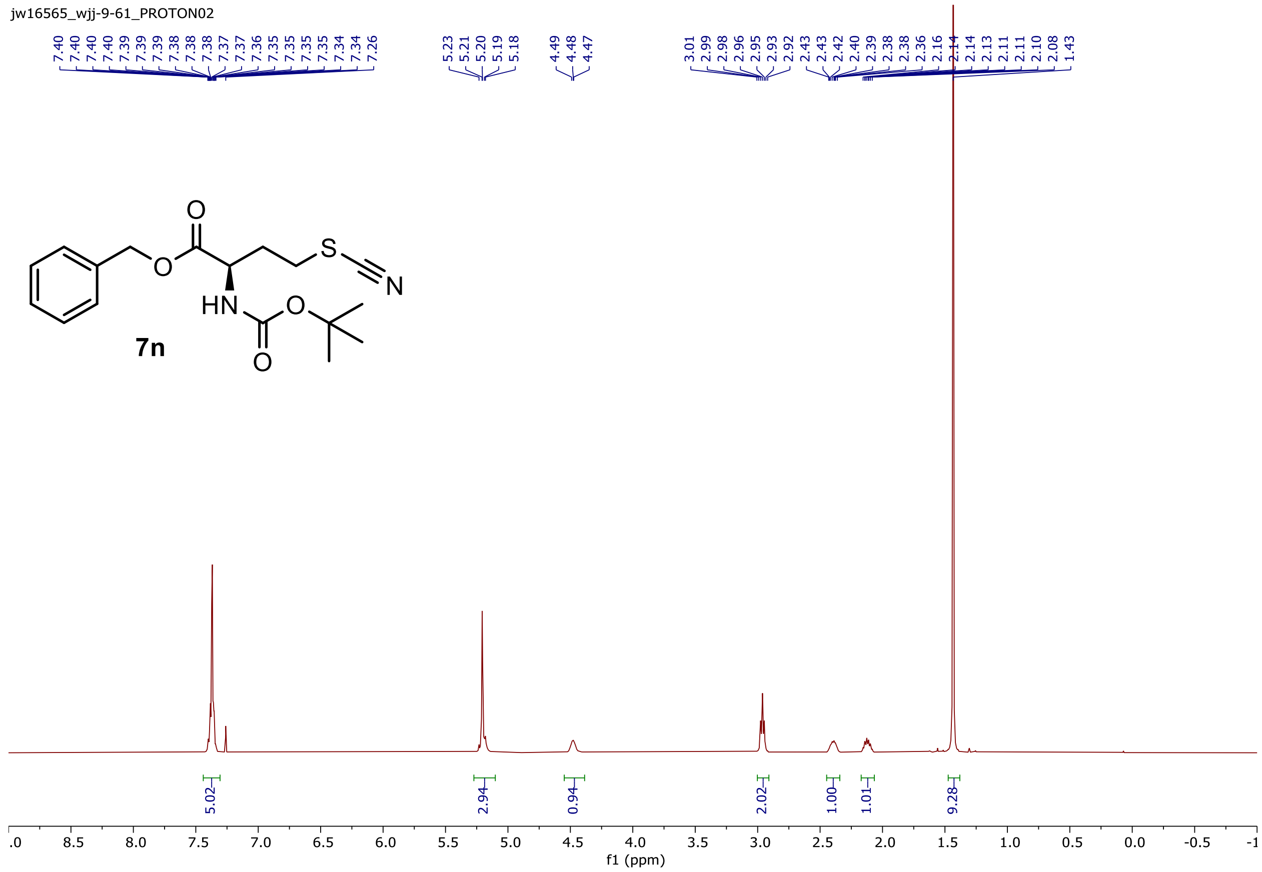

^{13}C NMR (125 MHz, $CDCl_3$) of **7n**

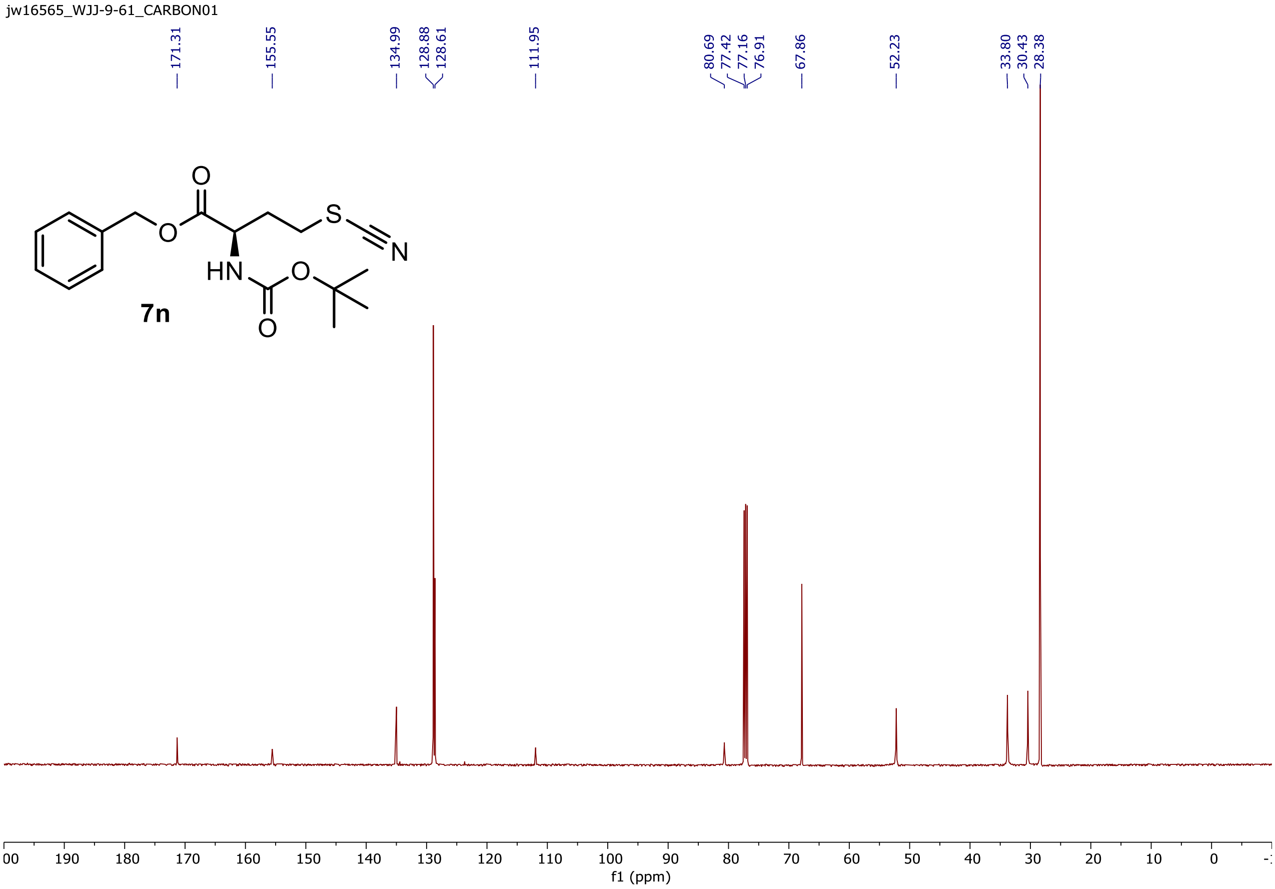

^{1}H NMR (500 MHz, $CDCl_3$) of **8n** (*see procedure*)

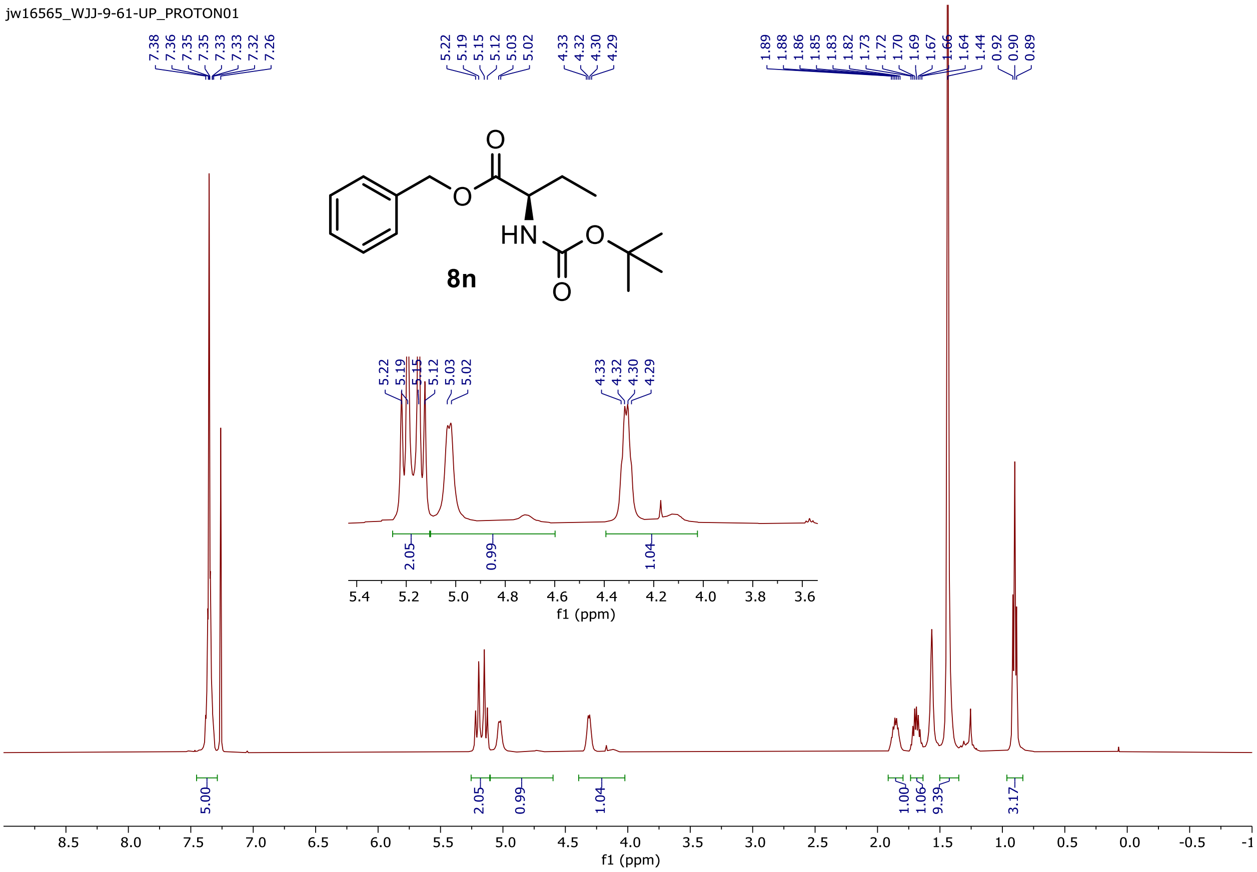

^{13}C NMR (125 MHz, $CDCl_3$) of **8n**

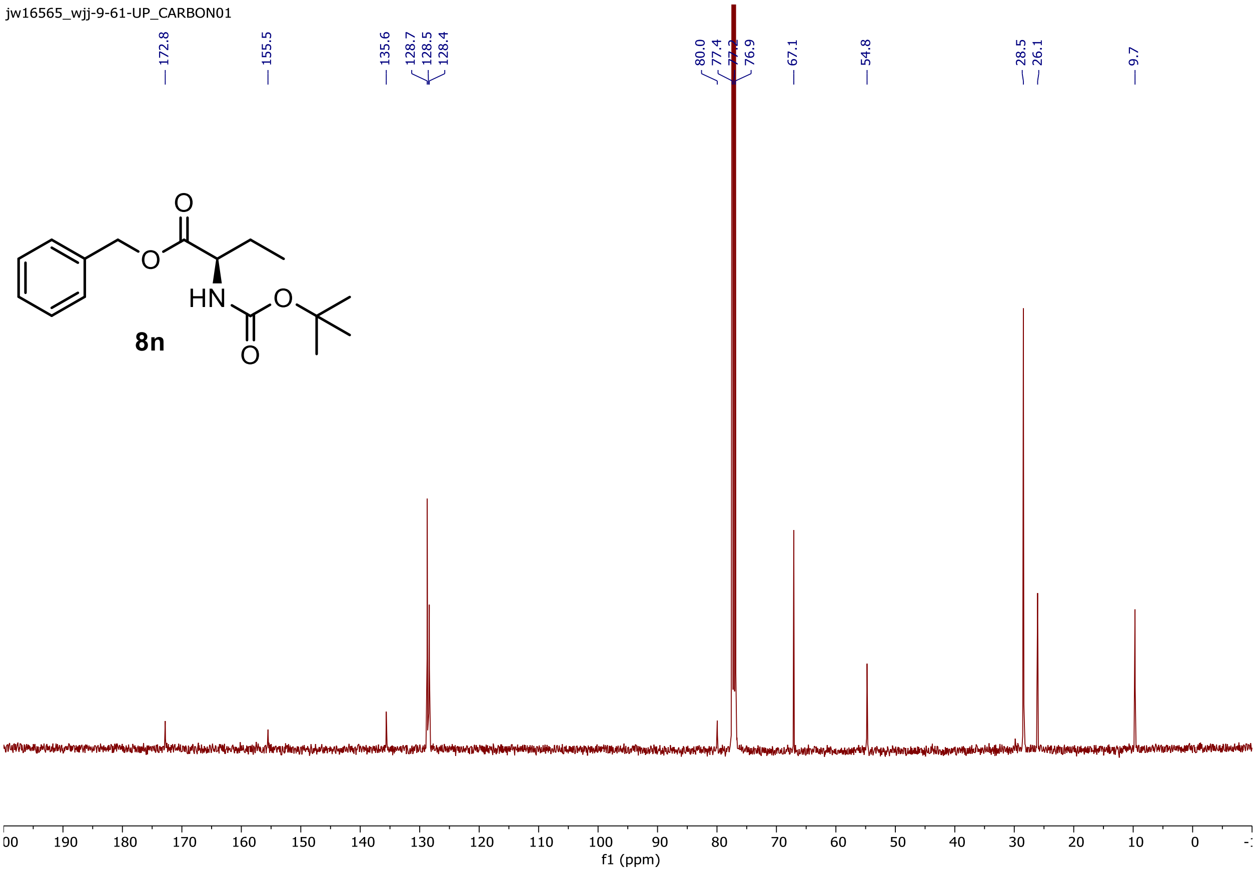

^{1}H NMR (500 MHz, CD_3CN) of **7o** (*see procedure*)

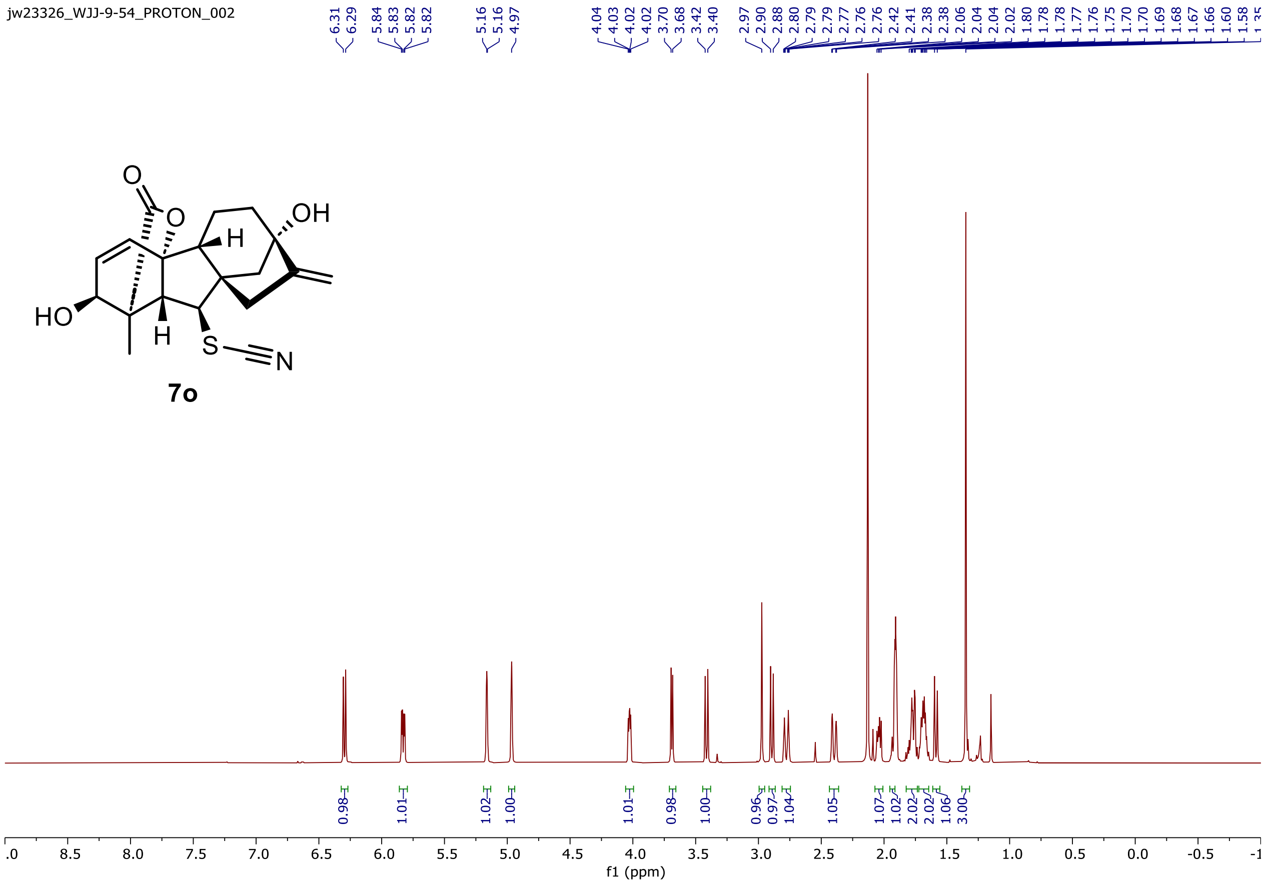

^{13}C NMR (125 MHz, CD_3CN) of **7o**

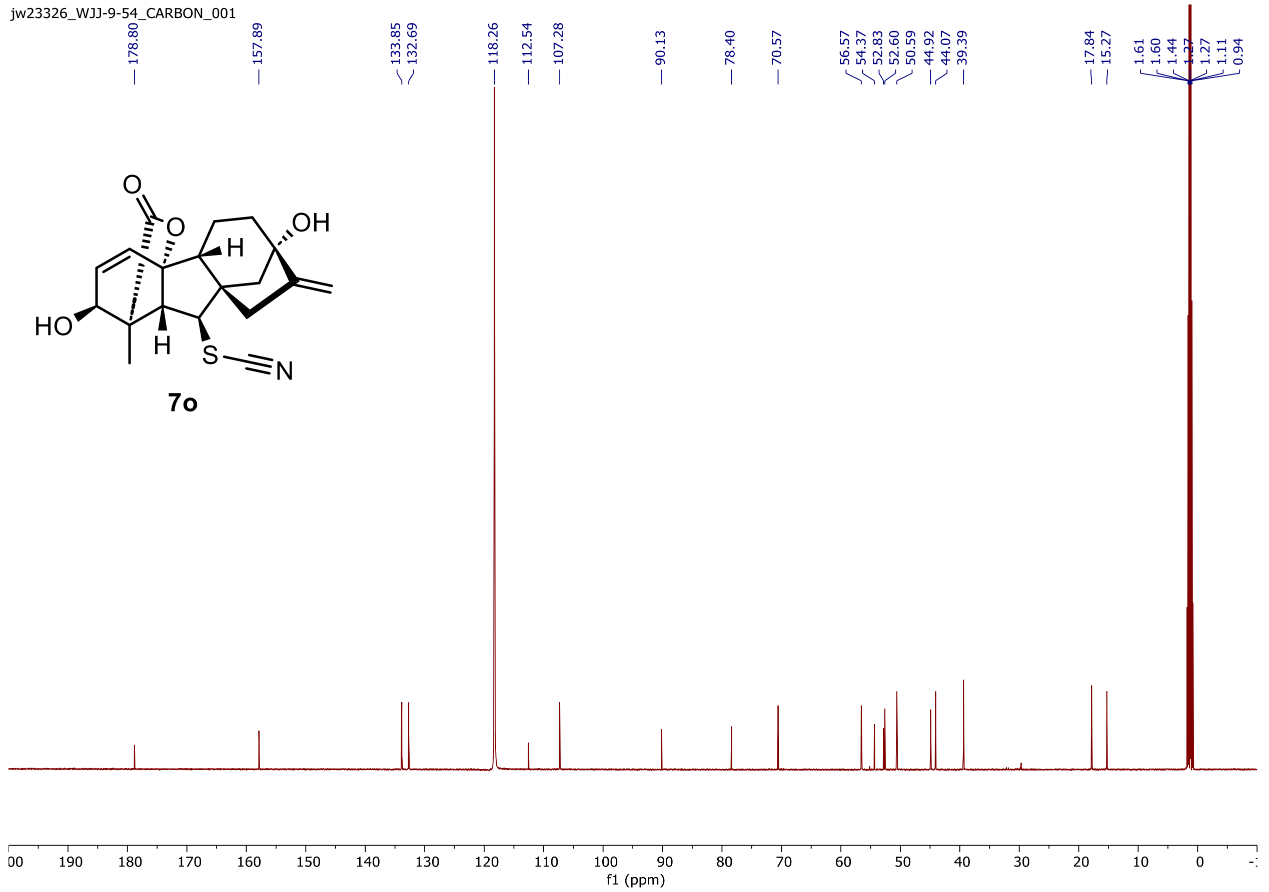

^1H–^1H COSY (CD_3CN) of **7o**

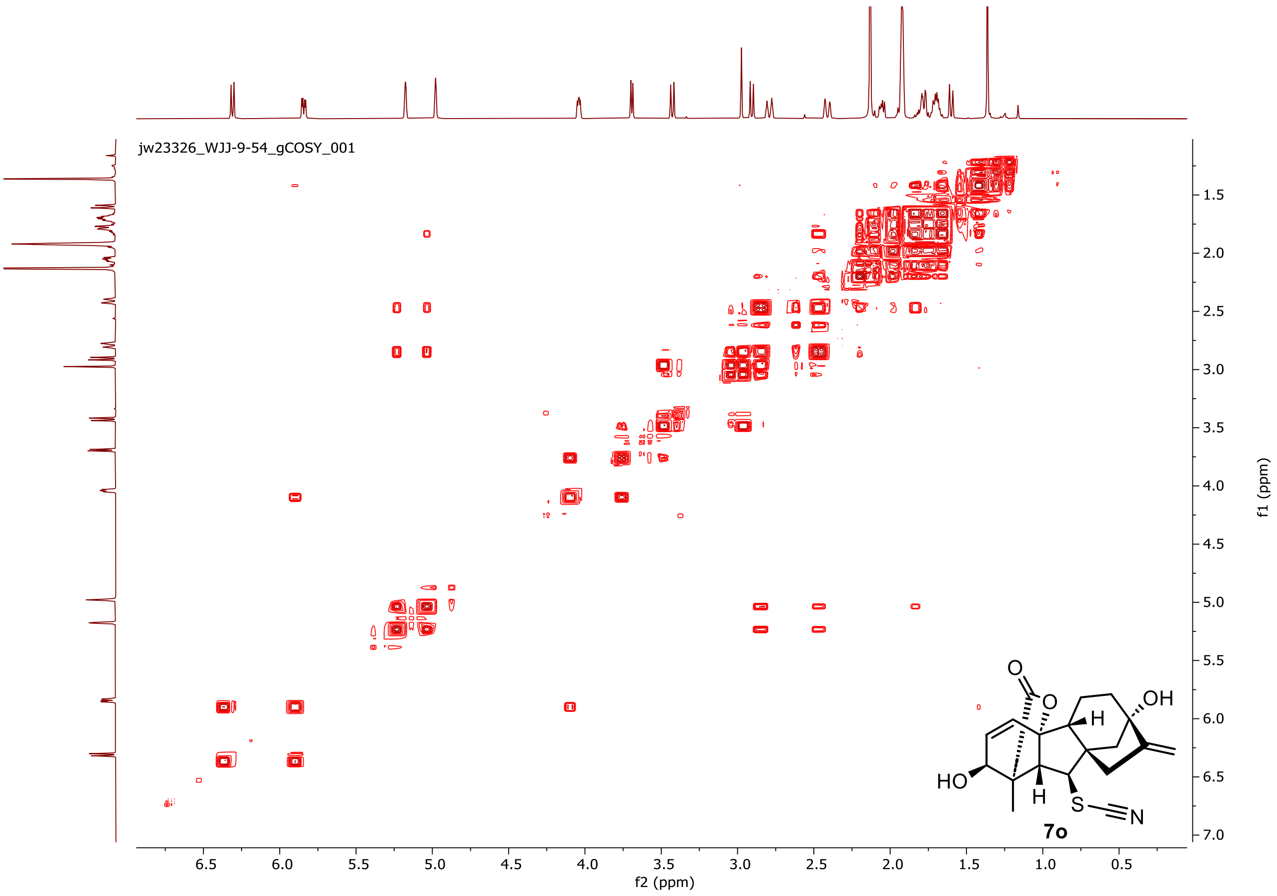

^1H–^{13}C HSQC (CD_3CN) of **7o**

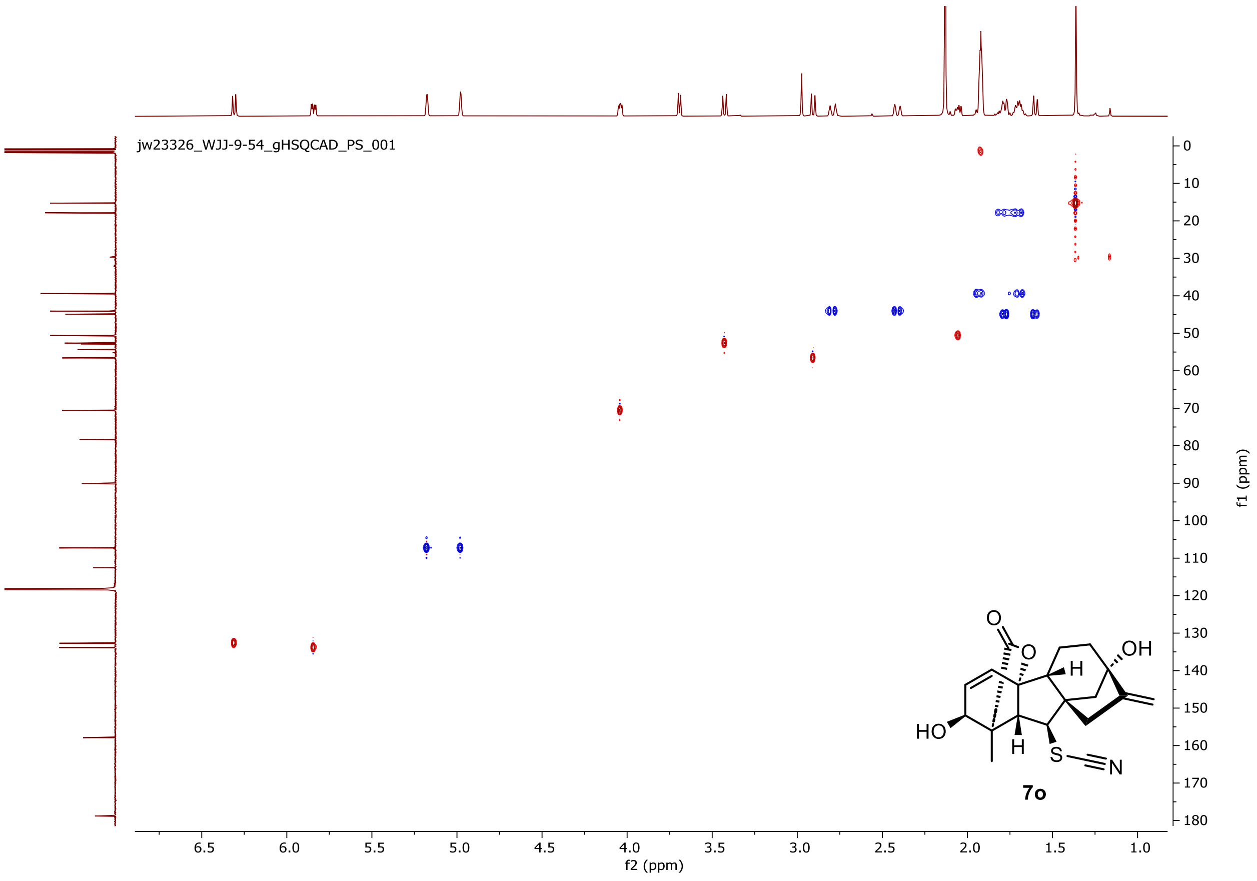

^{1}H NMR (500 MHz, $CDCl_3$) of **7p** (*see procedure*)

jw16658_WJJ-9-53_PROTON01

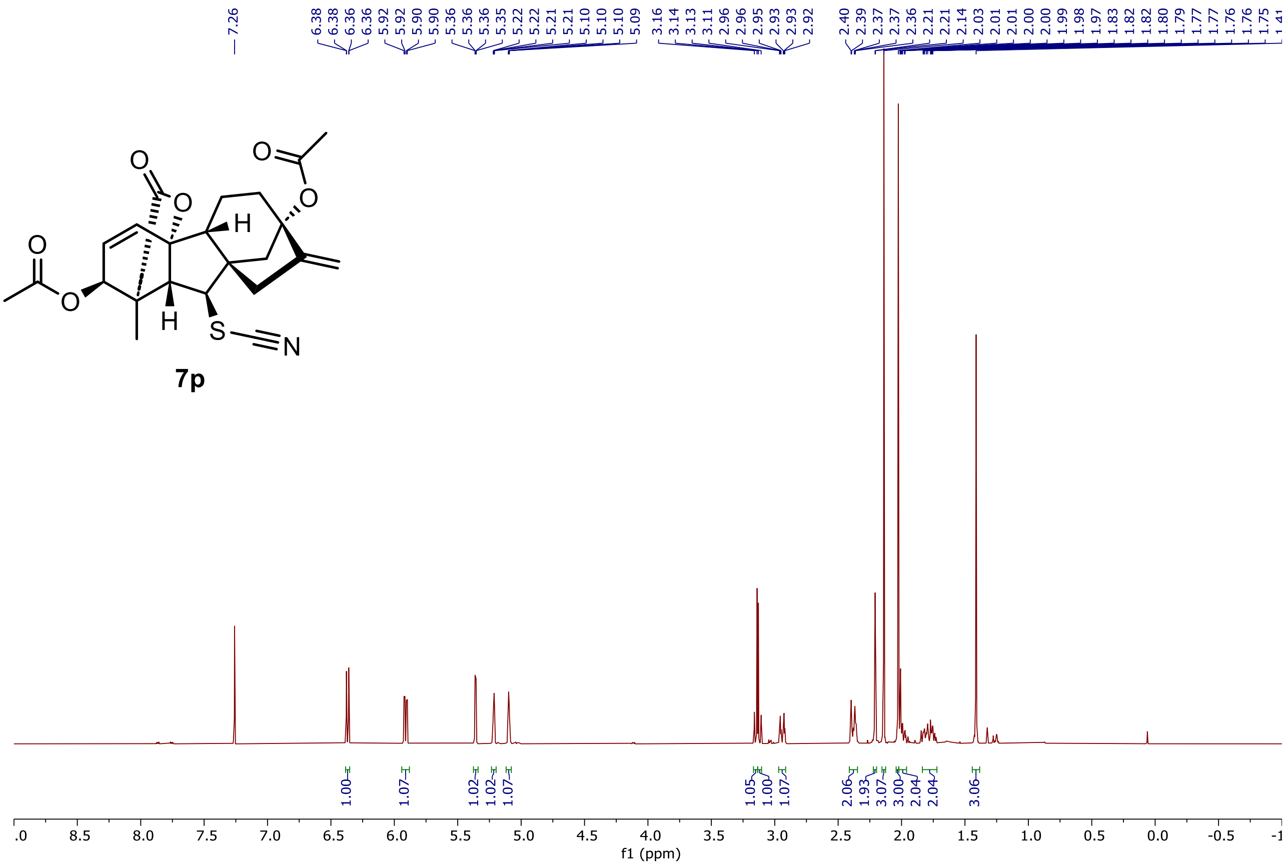

^{13}C NMR (125 MHz, $CDCl_3$) of **7p**

jw16658_WJJ-9-53_CARBON01

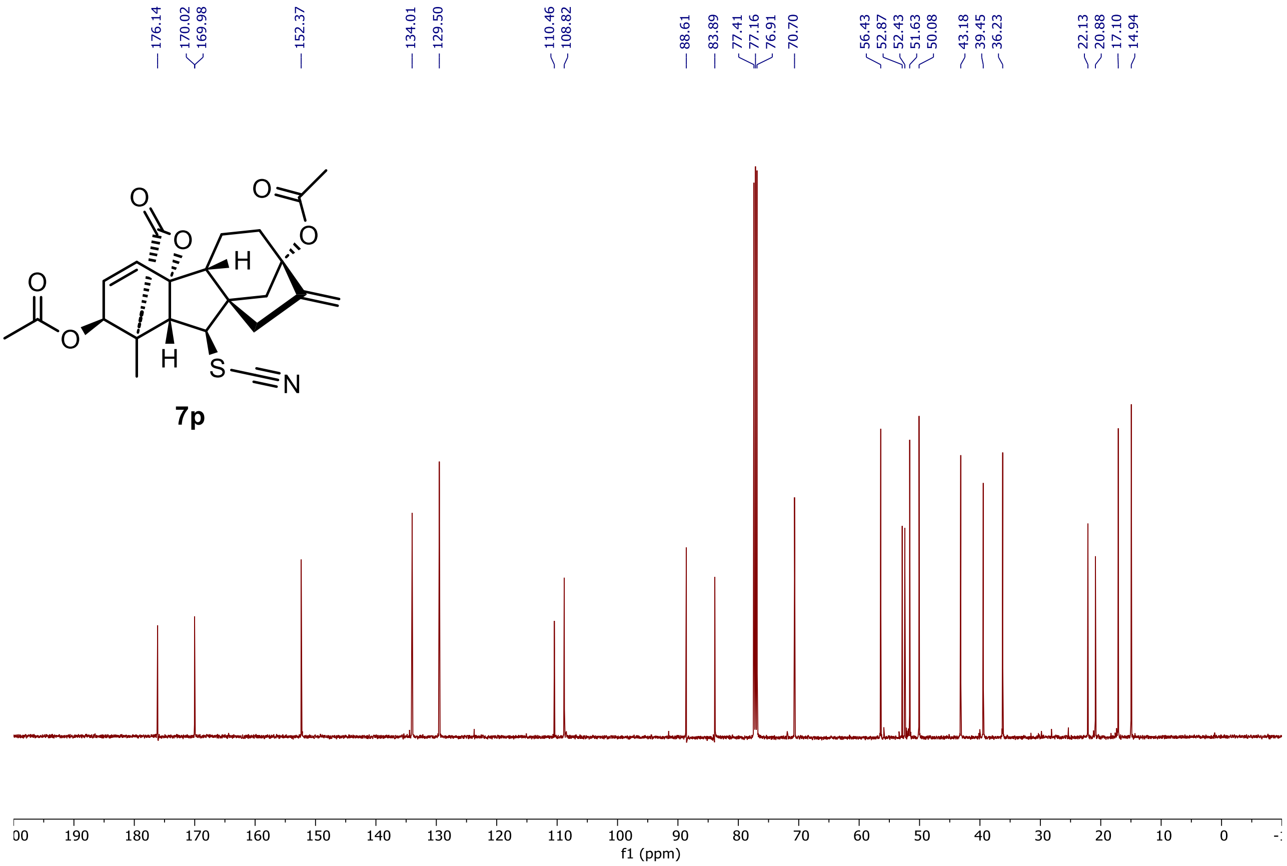

^{1}H–^{1}H COSY ($CDCl_3$) of **7p**

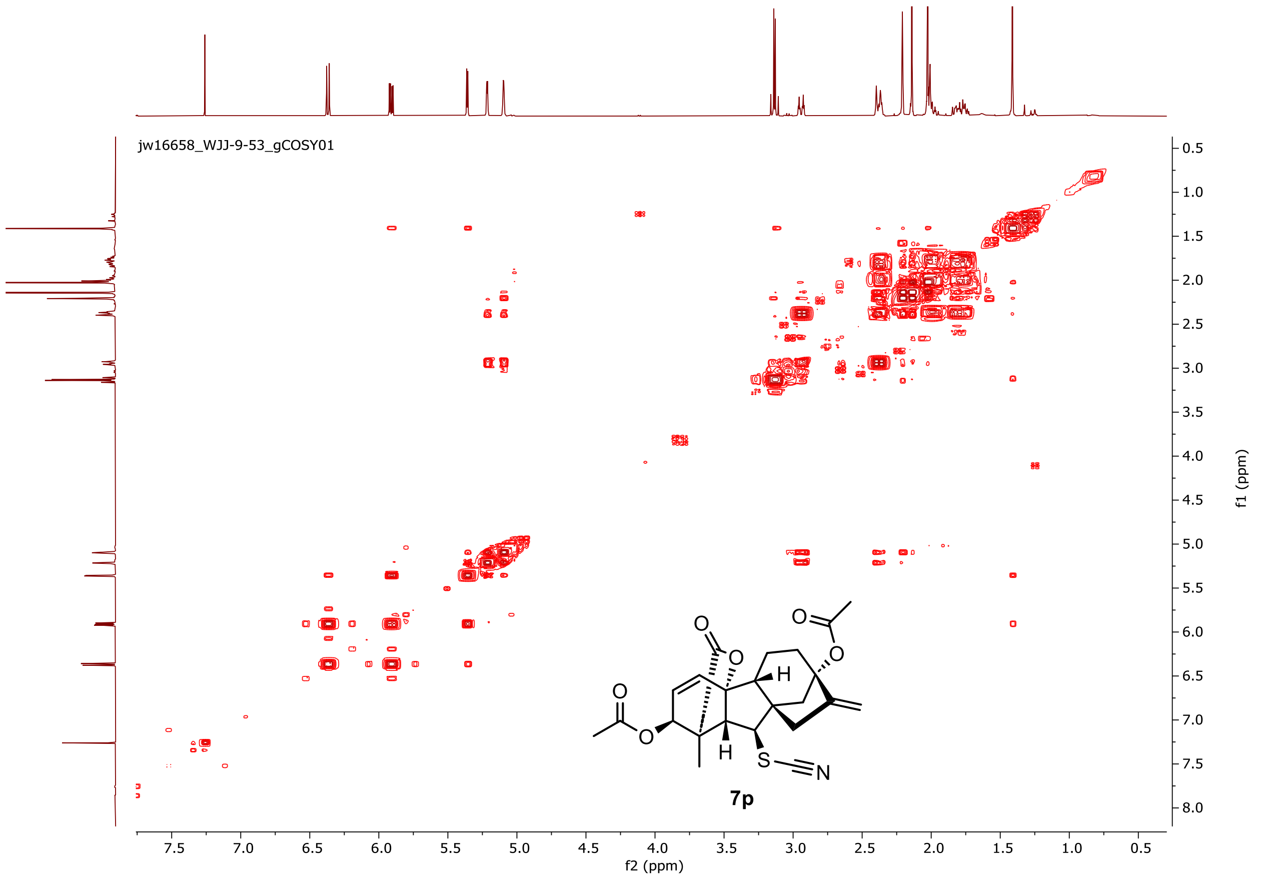

^{1}H–^{13}C HSQC ($CDCl_3$) of **7p**

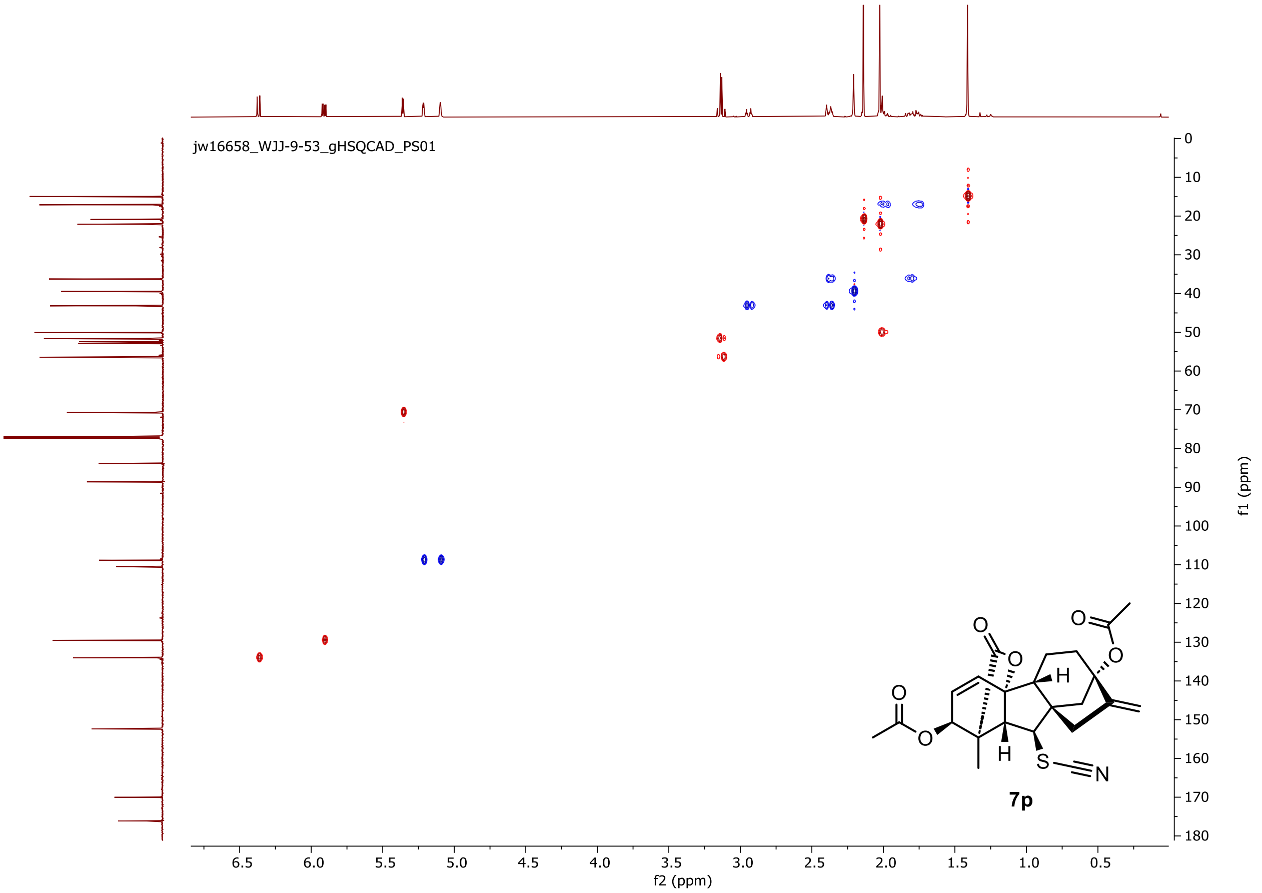

^{1}H NMR (500 MHz, $CDCl_3$) of **7q** (*see procedure*)

jw17821_WJJ-9-17_PROTON01

7q

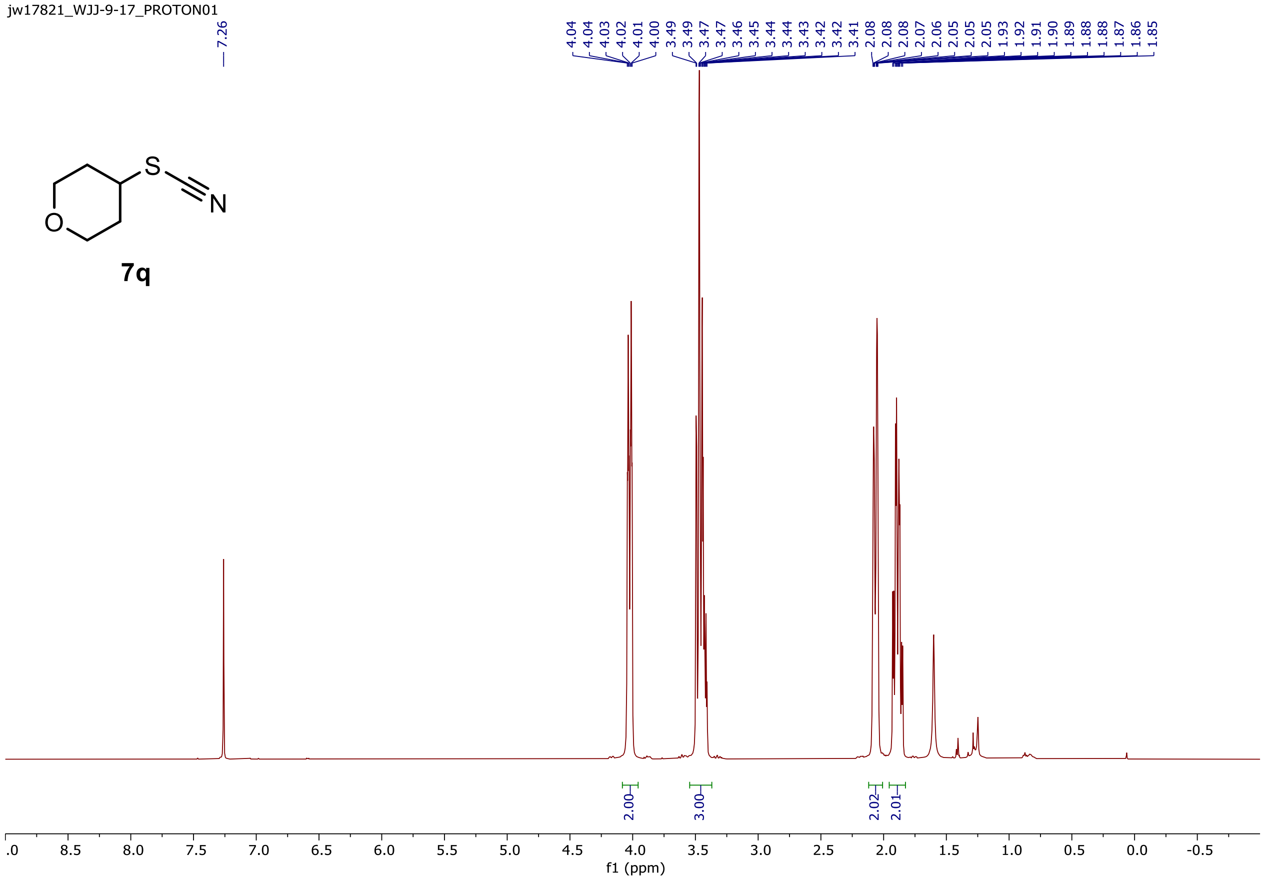

^{13}C NMR (125 MHz, $CDCl_3$) of **7q**

jw17821_WJJ-9-17_CARBON01

7q

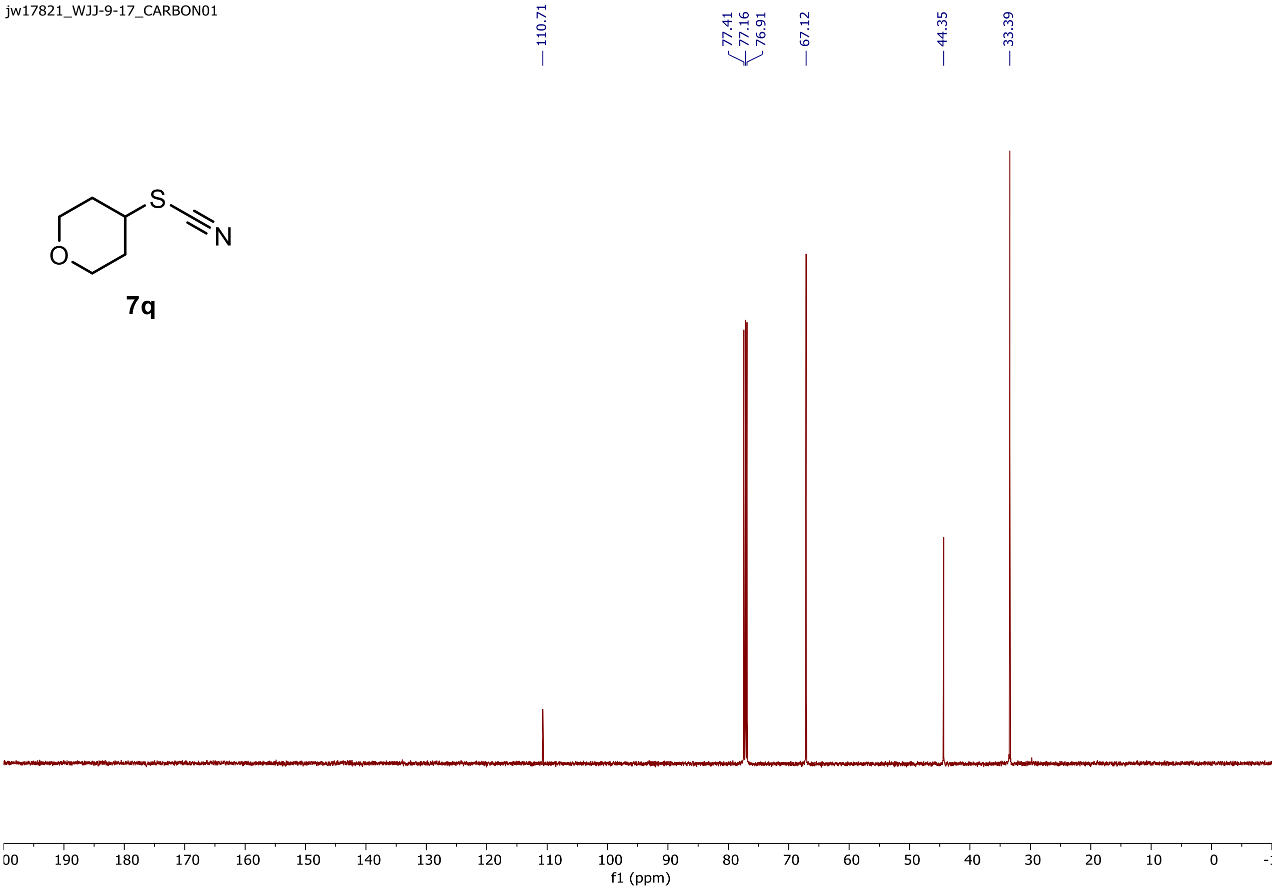

^{1}H NMR (500 MHz, $CDCl_3$) of **7r** (*see procedure*)

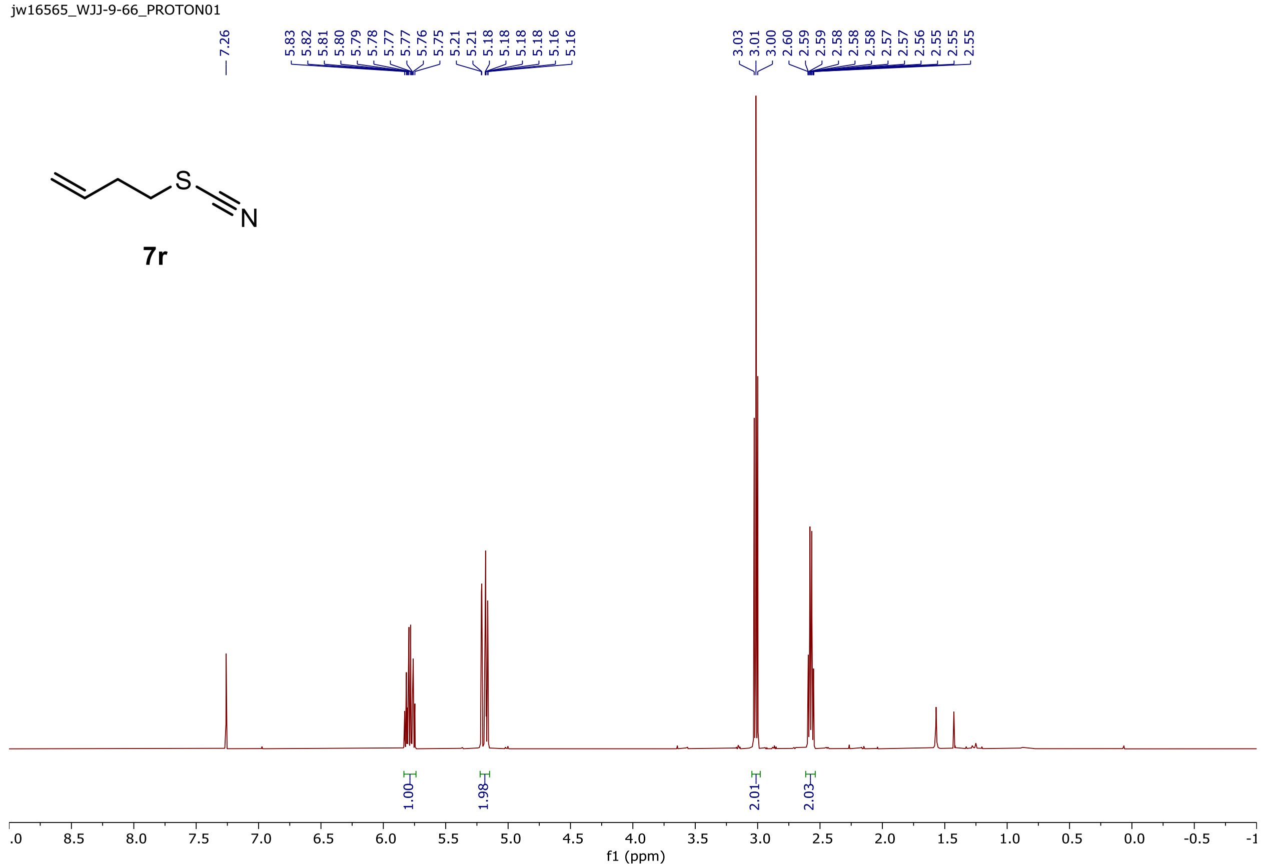

^{13}C NMR (125 MHz, $CDCl_3$) of **7r**

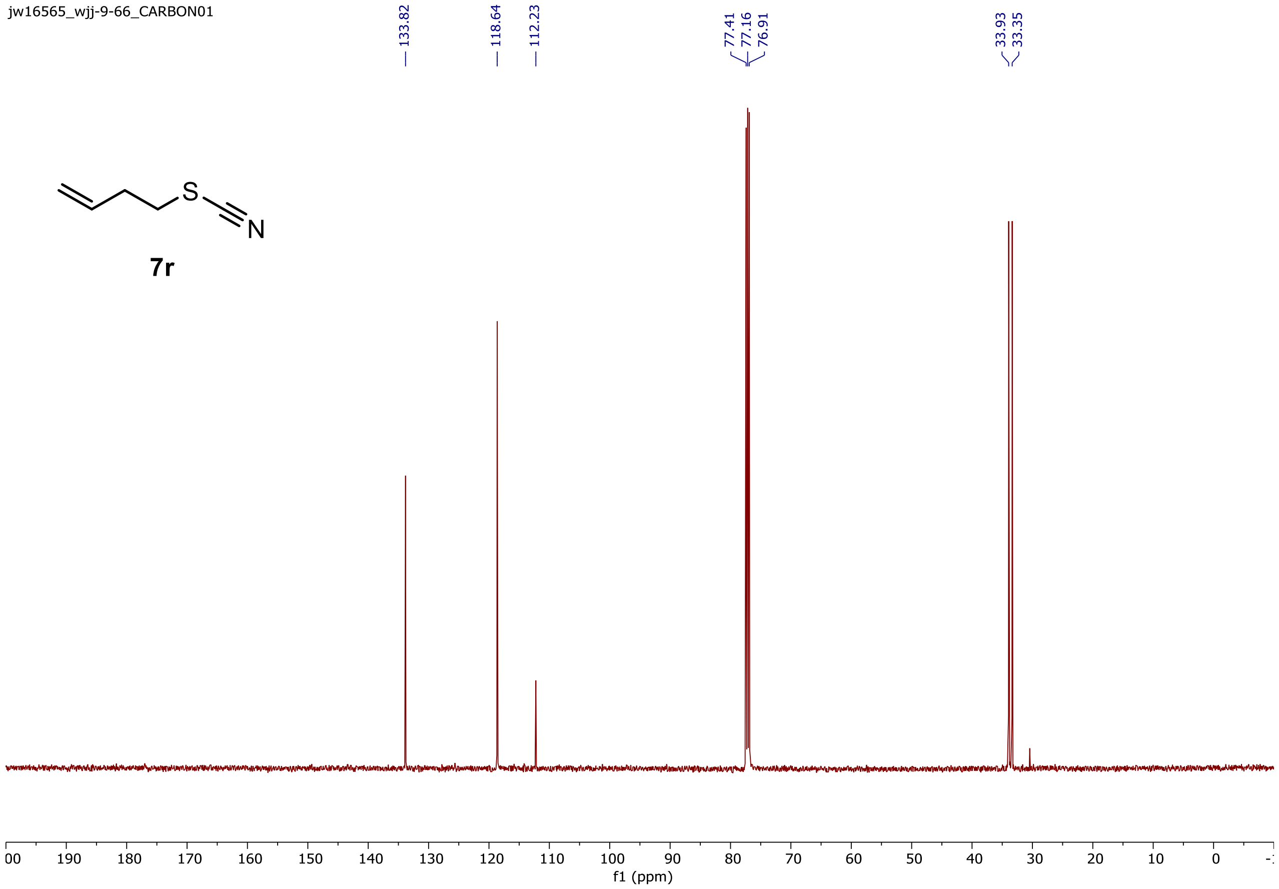

5. REFERENCES

1) A. Fawcett, J. Pradeilles, Y. Wang, T. Mutsuga, E. L. Myers, V. K. Aggarwal, *Science* **2017**, *357*, 283.

2) W. Zhao, R. P. Wurz, J. C. Peters, G. C. Fu, *J. Am. Chem. Soc.* **2017**, *139*, 12153–12156.

3) T. C. Sherwood, H.-Y. Xiao, R. G. Bhaskar, E. M. Simmons, S. Zaretsky, M. P. Rauch, R. R. Knowles, T. G. M. Dhar, *J. Org. Chem.* **2019**, *84*, 8360-8379.

4) K. Kulbitski, G. Nisnevich, M. Gandelman, *Adv. Synth. Catal.* **2011**, *353*, 1438–1442.

5) C. Andersen, V. Ferey, M. Daumas, P. Bernardelli, A. Guérinot, J. Cossy, *Org. Lett.* **2020**, *22*, 6021–6025.

6) E. Masson, F. Leroux, *Helv. Chim. Acta* **2005**, *88*, 1375–1386.

7) Y. Guindon, M. Therien, Y. Girard, C. Yoakim, *J. Org. Chem.* **1987**, *52*, 1680–1686.

8) W.-M. Cheng, R. Shang, B. Zhao, W.-L. Xing, Y. Fu, *Org. Lett.* **2017**, *19*, 4291–4294.

9) A. Watanabe, K. Koyamada, K. Miyamoto, J. Kanazawa, M. Uchiyama, *Org. Process Res. Dev.* **2020**, *24*, 1328–1334.

10) E. J. Ko, G. P. Savage, C. M. Williams, J. Tsanaktsidis, *Org. Lett.* **2011**, *13*, 1944–1947.

11) H. A. van Kalkeren, S. H. A. M. Leenders, C. R. A. Hommersom, F. P. J. T. Rutjes, F. L. van Delft, *Chem. Eur. J.* **2011**, *17*, 11290–11295.

12) M. B. Maquieira, A. B. Peñéñory, R. A. Rossi, *J. Org. Chem.* **2002**, *67*, 1012–1015.

13) D.-Z. Xu, Y. Liu, S. Shi, Y. Wang, *Tetrahedron: Asymmetry* **2010**, *21*, 2530–2534.

14) T. Iwasaki, R. Imanishi, R. Shimizu, H. Kuniyasu, J. Terao, N. Kambe, *J. Org. Chem.* **2014**, *79*, 8522–8532.

15) X. Fan, H. Zhao, J. Yu, X. Bao, C. Zhu, *Org. Chem. Front.* **2016**, *3*, 227–232.

16) S. Shahane, F. Louafi, J. Moreau, J.-P. Hurvois, J.-L. Renaud, P. van de Weghe, T. Roisnel, *Eur. J. Org. Chem.* **2008**, *2008*, 4622–4631.

17) T. Onoda, Y. Takikawa, T. Fujimoto, Y. Yasui, K. Suzuki, T. Matsumoto, *Synlett* **2009**, *2009*, 1041–1046.

18) L. Candish, E. A. Standley, A. Gómez-Suárez, S. Mukherjee, F. Glorius, *Chem. Eur. J.* **2016**, *22*, 9971–9974.

19) A. Villalpando, C. E. Ayala, C. B. Watson, R. Kartika, *J. Org. Chem.* **2013**, *78*, 3989–3996.

20) L. Han, J.-B. Xia, L. You, C. Chen, *Tetrahedron* **2017**, *73*, 3696–3701.

21) G.-D. Roiban, R. Agudo, M. T. Reetz, *Angew. Chem. Int. Ed.* **2014**, *53*, 8659–8663.

22) K. V. Chuang, R. Navarro, S. E. Reisman, *Chemical Science* **2011**, *2*, 1086–1089.

23) Z. Wang, L. Zhu, F. Yin, Z. Su, Z. Li, C. Li, *J. Am. Chem. Soc.* **2012**, *134*, 4258–4263.

24) F. Cros, B. Pelotier, O. Piva, *Synthesis* **2010**, *2010*, 233–238.

25) St J. Blanksby, G. B. Ellison, *Acc. Chem. Res.* **2003**, *36*, 255–263.
